# Transient Triplet Metallopnictinidenes M–Pn (M = Pd^II^, Pt^II^; Pn = P, As, Sb): Characterization and Dimerization

**DOI:** 10.1021/jacs.4c16830

**Published:** 2025-01-29

**Authors:** Marc C. Neben, Nils Wegerich, Tarek A. Al Said, Richard R. Thompson, Serhiy Demeshko, Kevin Dollberg, Igor Tkach, Gerard P. Van Trieste, Hendrik Verplancke, Carsten von Hänisch, Max C. Holthausen, David C. Powers, Alexander Schnegg, Sven Schneider

**Affiliations:** † Institut für Anorganische Chemie and International Center for Advanced Studies of Energy Conversion, Georg-August-Universität Göttingen, Tammannstr 4, 37077 Göttingen, Germany; ‡ Institut für Anorganische und Analytische Chemie Goethe-Universität, Max-von-Laue-Strasse7, 60438 Frankfurt am Main, Germany; § Helmholtz-Zentrum Berlin für Materialien und Energie GmbH, Hahn-Meitner-Platz 1, 14109 Berlin, Germany; ∥ EPR Research Group, MPI for Chemical Energy Conversion, Stiftstrasse 34−36, 45470 Mülheim Ruhr, Germany; ⊥ Department of Chemistry, University of Idaho, Moscow Campus, Moscow, Idaho 83844, United States; # Fachbereich Chemie, Philipps-Universität Marburg, Hans-Meerwein-Straße 4, 35043 Marburg, Germany; ¶ RG ESR Spectroscopy, Max Planck Institute for Multidisciplinary Sciences, Am Faßberg 11, 37077 Göttingen, Germany; ∇ Department of Chemistry, Texas A&M University, College Station, Texas 77843, United States

## Abstract

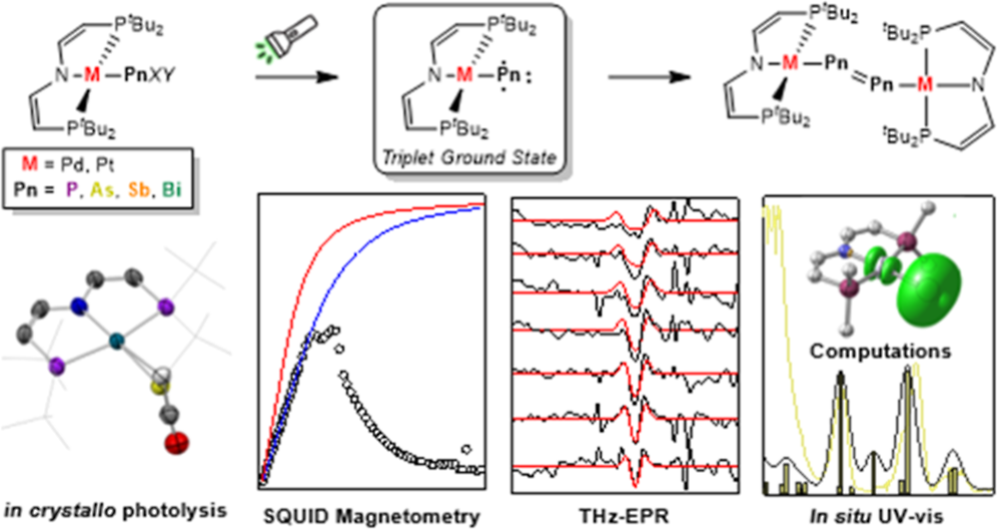

Nitrenes (R–N) have been subject to a large body of experimental and theoretical studies. The fundamental reactivity of this important class of transient intermediates has been attributed to their electronic structures, particularly the accessibility of triplet vs singlet states. In contrast, electronic structure trends along the heavier pnictinidene analogues (R–Pn; Pn = P–Bi) are much less systematically explored. We here report the synthesis of a series of metallodipnictenes, {M–Pn=Pn–M} (M = Pd^II^, Pt^II^; Pn = P, As, Sb, Bi) and the characterization of the transient metallopnictinidene intermediates, {M–Pn} for Pn = P, As, Sb. Structural, spectroscopic, and computational analysis revealed spin triplet ground states for the metallopnictinidenes with characteristic electronic structure trends along the series. In comparison to the nitrene, the heavier pnictinidenes exhibit lower-lying ground state SOMOs and singlet excited states, thus suggesting increased electrophilic reactivity. Furthermore, the splitting of the triplet magnetic microstates is beyond the phosphinidenes {M–P} dominated by heavy pnictogen atom induced spin–orbit coupling.

## Introduction

**Figure 1:**
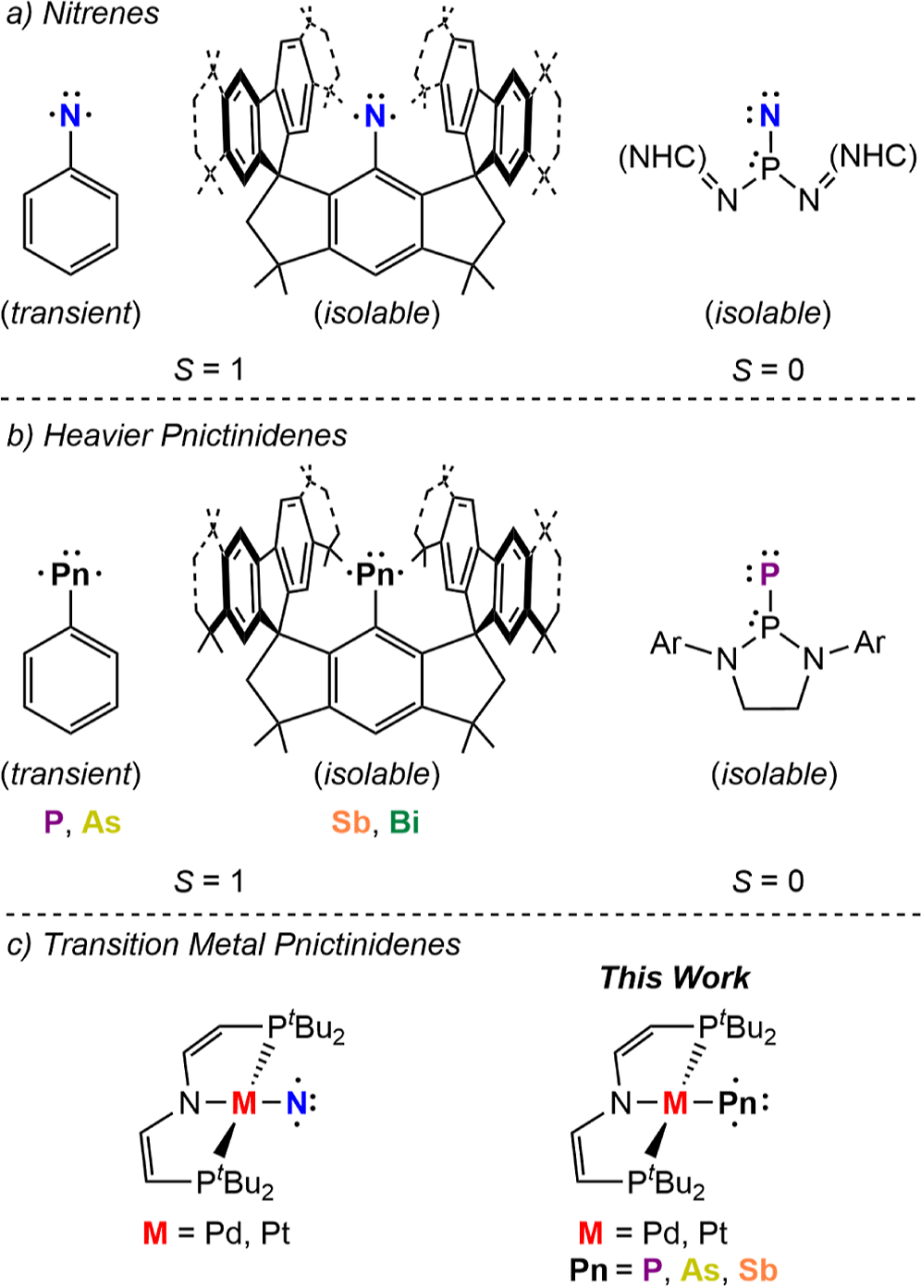
Selected examples of nitrenes, R–N (a) and heavier pnictinidenes, R–Pn (b) versus the metallopnictinidenes presented here (c).

Nitrenes (N–R), are key reactive intermediates in chemical synthesis and, as such, usually transient species.^1^ Nevertheless, a substantial body of matrix isolation, transient spectroscopy, and computational studies could connect the fundamental reactivity of these subvalent species to their spin state energetics.^2^ Parent imidogen (N–H) is a prototypical axial diradical with two electrons in two localized, degenerate, and orthogonal orbitals.^3^ In consequence, the lowest electronic configuration 1σ^2^2σ^2^3σ^2^1π^2^ gives rise to a triplet ground (^3^Σ^–^) and two singlet excited states (^1^Δ and ^1^Σ^+^).^4^ Simple alkyl- and arylnitrenes exhibit the same state ordering with a ground state that is moderately split by dipolar spin interactions (*D* ≈ 1–2 cm^–1^).^5,6^ Excessive steric shielding recently enabled the isolation of persistent triplet arylnitrenes (Figure 1a).^7^ In turn, the singlet electromer is stabilized by breaking the π_N_ orbital degeneracy,^8^ and Bertrand could isolate a singlet nitrene with a strong, unidirectional π-push substituent (Figure 1a).^9^

In contrast to nitrenes, the heavier pnictinidenes, Pn–R (Figure 1b; Pn = P, As, Sb, Bi), are much less well explored.^10^ Computational studies predicted a triplet ground state for methylphosphinidene, P–Me, but facile isomerization via the lower lying singlet states^11^ prevented its spectroscopic confirmation until very recently.^12^ EPR characterization of a triplet phoshinidene (P–Mes) was first reported in 1994;^13^ matrix isolation studies of transient arylphosphinidenes and -arsinidenes even much more recently.^14^ Sb–Ph and Bi–Ph remain elusive. Triplet Bi–Me could recently be detected in the gas-phase.^15^ The installation of bulky aryl substituents also enabled the isolation of the heaviest triplet pnictinidenes (Figure 1b),^16,17^ while the analogous phosphinidene proved unstable toward intramolecular decay.^18^ However, in analogy to nitrenes a strong π-donor substituent gave rise to an isolable singlet phosphinidene (Figure 1b).^19^

Notably, the heavier triplet pnictinidenes showcase an increasing impact of relativistic effects. For example, Cornella’s bismuthinidene (Figure 1b) exhibits a nonmagnetic triplet ground state owing to huge spin–orbit coupling (SOC) induced zero-field-splitting (ZFS) of more than +5400 cm^–1^.^16,20^ Thus, SOC-induced stabilization is of a magnitude that not only dominates the spectroscopic and magnetic properties, but might be of direct relevance for chemical reactivity. Systematic studies of these electronic structure trends are of key importance to support the current emergence of redox catalysis mediated by heavy main group elements.^21^

While azide dissociation is most common for nitrene generation, alternative strategies are required for the heavier analogues. Thermal or photochemical fragmentation of precursors, like ^*t*^Bu_2_P–P=PBr^*t*^Bu_2_, phospholenes, phosphiranes, or dibenzophosphanorbornadienes, are versatile routes to transient phosphinidenes.^10,22^ The heavier pnictinidenes are commonly synthesized upon reduction of Pn^III^ (pseudo)halides.^16,17^ Furthermore, pnictaethynolate anions (PnCO^–^, Pn = P, As)^23^ have recently emerged as versatile reagents for pnictogen atom transfer to transition metals.^24^ CO dissociation gave rise to pnictide complexes with M≡P triple bonds and dipnictene (M–Pn=Pn–M) coupling products.^25^ However, heavier authentic metallopnictinidenes (M–Pn), which are analogous to organic triplet pnictinidenes with a limiting electronic configuration that represents M–Pn single bonding, remain elusive.^26^

**Scheme 1:**
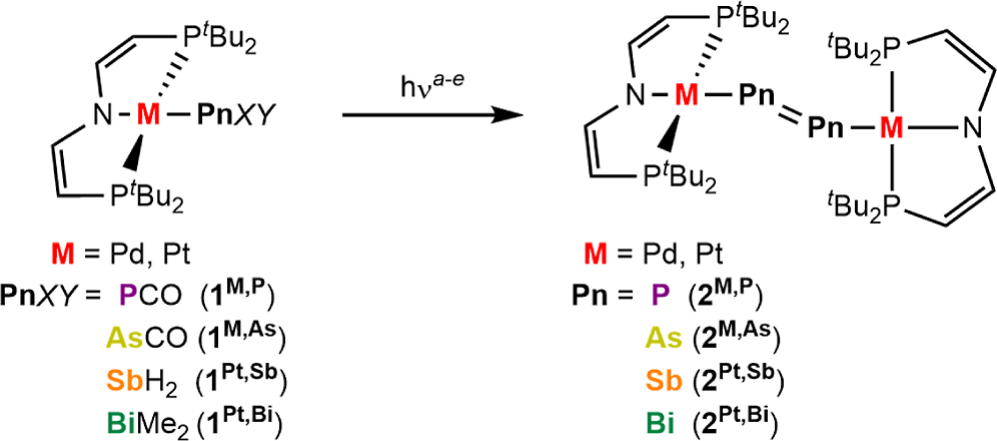
Photoinduced Pnictide Coupling; Reaction Conditions: (a) **2**^**Pd,P**^: λ = 456 nm, Benzene, 3 h, Room Temperature, 87% Yield; (b) **2**^**Pt,P**^: see ref (28); (c) 2^Pd,As^: λ = 525 nm, THF, 90 min, 0 °C, 82% Yield; (d) **2**^**Pt,As**^: λ = 525 nm, Toluene, 30 min, −30 °C; 87% Yield; (e) **2**^**Pt,Sb**^: λ = 370 nm, 2-Methyl THF, 6 h, −196 °C; 84% Yield; (f) **2**^**Pt,Bi**^: λ = 370 nm, Toluene, 16 h,–40 °C, 25% Yield

Our groups recently reported the synthesis of the formal group 10 nitrido complexes, [M(N)(PNP)] [M = Pd, Pt; PNP = N(CHCHP^*t*^Bu_2_)_2_; Figure 1c],^27^ as authentic metallonitrenes (M–N) with a univalent nitrogen diradical ligand that is singly bound to closed shell M^II^ ions. Furthermore, photolysis of the phosphaethynolate complex [Pt(PCO)(PNP)] (**1**^**Pt,P**^, Scheme 1) gave the pnictide coupling product [(P_2_){Pt(PNP)}_2_] (**2**^**Pt,P**^),^28^ suggesting a transient metallophosphinidene intermediate. We here report the synthesis of the full heavier metallodipictene series, [(Pn_2_){M(PNP)}_2_] (Pn = P–Bi) as well as the *in situ* characterization of the pnictinidene intermediates, [M(Pn)(PNP)}] (Pn = P–Sb), providing systematic electronic structure trends along the series.

## Results and Discussion

### Precursor Syntheses

In analogy to previously reported **1**^**Pt,P**^,^28^ the diamagnetic precursors [M(PnCO)(PNP)] (**1**^**M,Pn**^; M = Pd, Pt; Pn = P, As; Scheme 1) were obtained in near quantitative yields by salt metathesis from the divalent group 10 triflates with [Na(diox)_*n*_]PnCO (Pn = P, As). Structural characterization of all products by single-crystal X-ray diffraction (see Supporting Information) showed marginal variations within the M(PNP) fragments. The smaller M–Pn–C angles for Pn = As [**1**^**Pd,As**^: 95.87(15)°, **1**^**Pt,As**^: 99.0(3)°] versus P [**1**^**Pd,P**^: 99.63(3)°, **1**^**Pt,P**^: 101.74(11)°] are in line with increased pnictogen p-character in M–PnCO bonding.^29^ All complexes feature absorption bands in the visible range at around 500–600 nm, which can be assigned to electronic LMCT(PnCO → M) transitions (cf. Supporting Information for further details).

The lack of the heaviest pnictaethynolate precursors required alternative strategies for Sb and Bi.^30^ The Liddle group prepared thorium stibinidene and stibido complexes, starting from SbH_2_^–^.^31^ Motivated by this work, we synthesized the parent stibanide complex [Pt(SbH_2_)(PNP)] (**1**^**Pt,Sb**^, Scheme 1) with [K(18-crown-6)(thf)SbH_2_].^32^ Isolation of the Pd analogue was not successful due to low thermal stability. The SbH_2_ substituent of **1**^**Pt,Sb**^ exhibits a ^1^H NMR signal at δ = −0.94 ppm, comparing well with reported primary stibanes.^33^ The Sb–H stretching modes are assigned to a broad IR band at ν̃ = 1834 cm^–1^, which is blue-shifted versus the potassium salt (Δν̃ = 60 cm^–1^). **1**^**Pt,Sb**^ exhibits an absorption band in the near-UV region (λ_max_ = 318 nm) that is assigned to a π_*C*=*C*_ → π_*C*=C_^*^ transition in the pincer ligand backbone upon comparison with [Pt(N_3_)(PNP)].^34^

**Figure 2:**
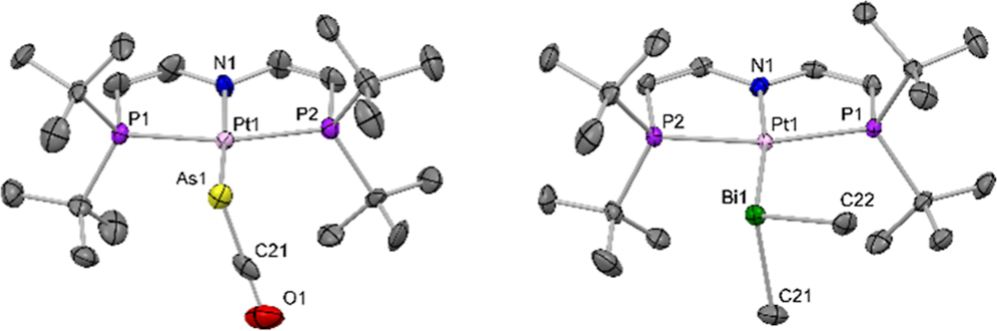
Molecular structure of **1**^**Pt,As**^ (left) and **1**^**Pt,Bi**^ (right) with thermal ellipsoids at the 50% probability level. H atoms are omitted for clarity. Selected bond lengths [Å] and angles [°]:**1**^**Pt,As**^ Pt1–As1:2.455(1); Pt1–As1–C21:99.0(3); **1**^**Pt,Bi**^: Pt1–Bi1:2.7323(3); Pt1–Bi1–C21:99.69(15), Pt1–Bi1–C22:117.53(14), C21–Bi1–C22:91.7(2).

As BiH_2_^–^ is highly unstable, we resorted to organobismuth analogues, which can be generated from triorganobismuthanes with alkali metal.^35^ Thermal Bi–C homolysis of BiMe_3_ has been reported at moderate temperatures (60–120 °C).^15^ Salt metathesis of [Pt(OTf)(PNP)] with *in situ* generated BiMe_2_^–^ gave a product in up to 90% ^31^P NMR spectroscopic yield that is assigned to [Pt(BiMe_2_)(PNP)] (**1**^**Pt,Bi**^) based on mass spectrometry and single crystal X-ray diffraction (Figure 2). The molecular structure of **1**^**Pt,Bi**^ reveals a long Pt–Bi bond [2.7323(3) Å] and almost orthogonal methyl groups [C–Bi–C: 91.7(2)°], which is in line with purely p_Bi_ orbital contributions to bonding [cf. Supporting Information for a detailed natural bond orbital (NBO) analysis]. Notably, **1**^**Pt,Bi**^ is the first structurally characterized transition metal diorganobismuthyl complex.^36^ Besides **1**^**Pt,Bi**^, a side product (10–15%) is observed. The small ^1^*J*_Pt–P_ coupling constant (469 Hz) suggests high-valent Pt, e.g., due to oxidative addition to Pt^II^.^37^ While identification and separation of the side product unfortunately remained unsuccessful, the mixture could be directly used as precursor for dibismuthene synthesis (see below).

### Photolytic Pnictide Coupling

In the absence of light, the phosphorus precursors **1**^**Pd,P**^/**1**^**Pt,P**^ are stable in solution at room temperature over several days. The other precursors slowly decay at these conditions giving either the respective pnictide coupling products (see below) as main products or lead to undefined decomposition in case of **1**^**Pt,Sb**^. Photolysis was carried out in solution, guided by the thermal and spectroscopic properties of the respective precursors (Scheme 1). For **1**^**Pd,P**^**/1**^**Pd,As**^ and **1**^**Pt,P**^**/1**^**Pt,As**^, decay of the characteristic ν(PnCO) IR band confirmed the conversion of the precursors over 1–3 h with concomitant growth of new electronic absorption features in the visible range. As previously reported for **1**^**Pt,P**^,^28^ the Pd^II^ and Pt^II^ metallodipnictene products [(μ-Pn_2_){M(PNP)}_2_] (**2**^**M,Pn**^; M = Pd, Pt; Pn = P–Sb) could be isolated in yields beyond 80%, while significantly smaller yields (∼25%) were obtained for **2**^**Pt,Bi**^. All coupling products proved thermally stable at room temperature in solution and in the solid state. Note that the analogous diazenido complex [(μ-N_2_){Pt(PNP)}_2_] readily loses N_2_ at low temperatures.^38^ Heavier dipnictenes, R–Pn=Pn–R, with organic, main group, and transition metal substituents are well-known for Pn = P–Sb.^25a,28,33,39^ In contrast, only few dibismuthenes (R–Bi=Bi–R) have been reported,^16a,39,40^ as well some metal compounds of the Bi_2_^2–^ and Bi_2_^3–^ radical anions.^41,42^

**Figure 3:**
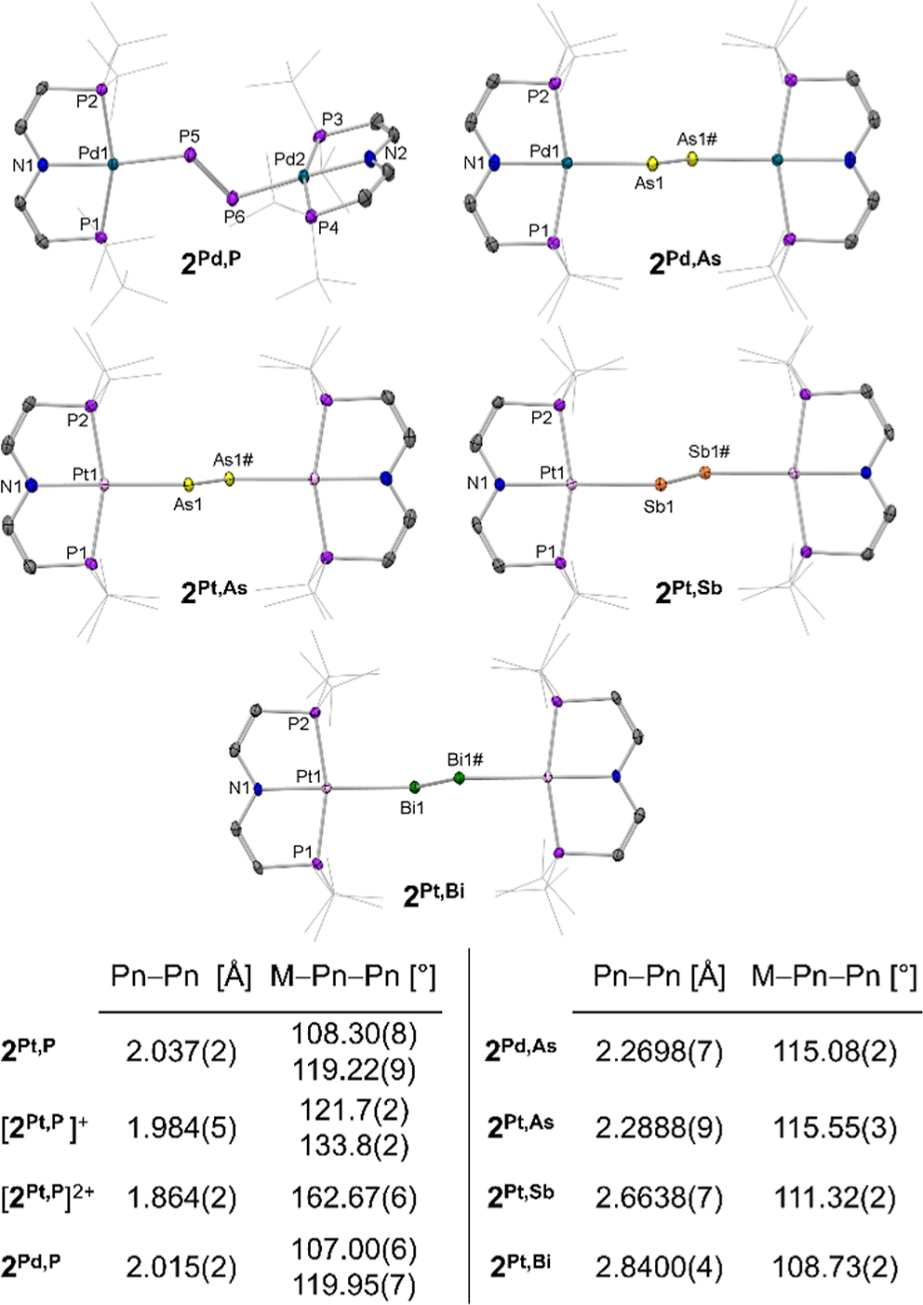
Molecular structures and selected structural parameters of the dipnictenes with thermal ellipsoids at the 50% probability level. H atoms and disordered atoms are omitted and ^*t*^Bu-groups represented as sticks for clarity. Structural parameters of **2**^**Pt,P**^, **[2**^**Pt,P**^**]**^**+**^, and **[2**^**Pt,P**^**]**^**2+**^ taken from ref (28).

The broad ^31^P NMR signal at δ_P_ = +784 ppm is assigned to the P_2_-bridged Pd complex **2**^**Pd,P**^, supporting P=P double bond character, as for previously reported **2**^**Pt,P**^ (δ_P_ = +707 ppm).^28^ NMR spectroscopic characterization of **2**^**Pt,Bi**^ was unfortunately impeded by the extremely low solubility in common organic solvents. All coupling products were characterized by single-crystal X-ray diffraction (Figure 3). Comparison with Pyykkö’s covalent bond radii^43^ and computed Wiberg bond indices support Pn=Pn double bonding. The elongation of the Pn=Pn bonds from {P_2_}^2–^ to {Bi_2_}^2–^ is in line with the increasing valence orbital expansion (see Supporting Information for detailed results of NBO analysis).^29,44^ Accordingly, the As=As stretching vibrations [ν̃_exp_ = 308 (**2**^**Pd,As**^) and 302 cm^–1^ (**2**^**Pt,As**^)] are red-shifted vs P=P [ν̃_exp_ = 585 (**2**^**Pd,P**^) and 582 cm^–1^ (**2**^**Pt,P**^)]. While the Sb=Sb and Bi=Bi stretching modes could not be reliably assigned from experiment, DFT computations confirmed the trend for the full series [ν̃_DFT_/cm^–1^ = 561 (**2**^**Pt,P**^), 314 (**2**^**Pt,As**^), 204 (**2**^**Pt,Sb**^), 149 (**2**^**Pt,Bi**^)]. This picture is complemented by comparison of the dipole allowed electronic π(Pn_2_) → π^*^(Pn_2_) transitions (**2**^**Pt,P**^: 22,400, **2**^**Pt,As**^: 21,400, **2**^**Pt,Sb**^: 20,100, **2**^**Pt,Bi**^: 16,800 cm^–1^; see Supporting Information for Pd complexes), which were assigned upon comparison with TD-DFT calculations.

**Figure 4:**
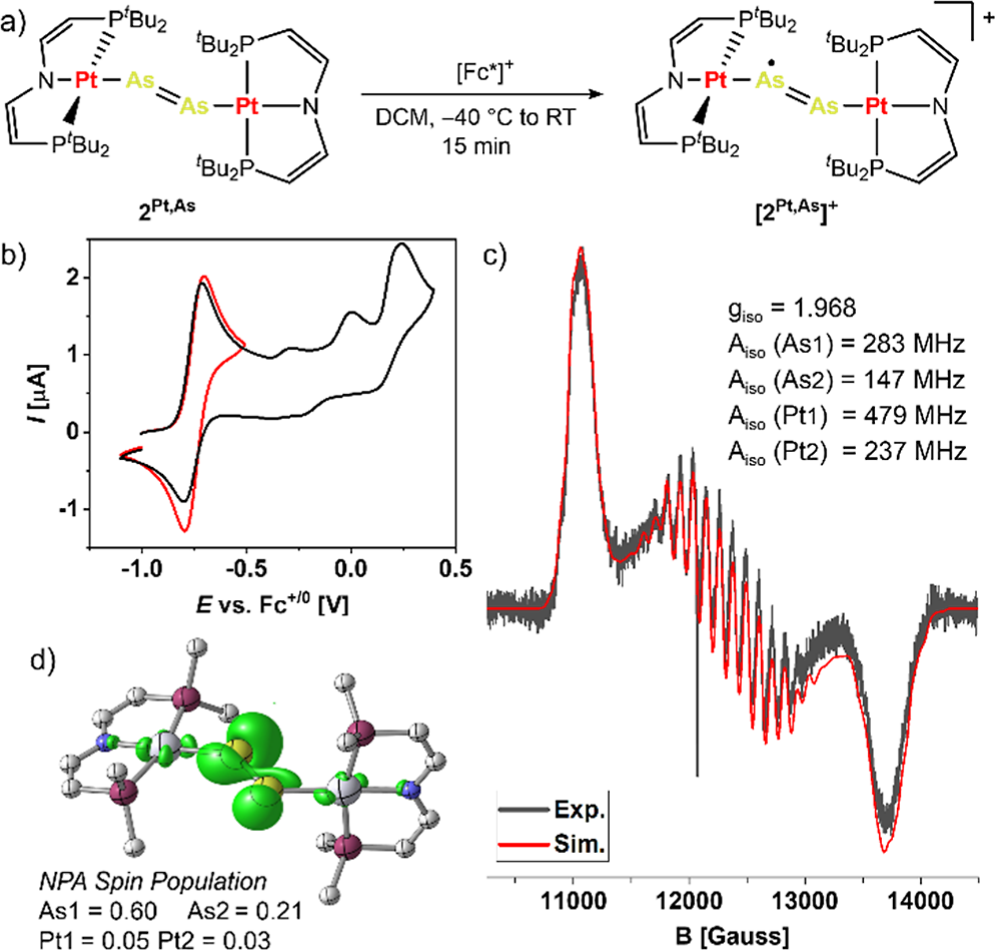
(a) Synthesis of radical complex **[2**^**Pt,As**^**]**^**+**^. (b) Cyclic voltammogram of **2**^**Pt,As**^ in PhF. (c) Continuous wave *Q*-band EPR spectrum (black) and simulation (red with simulation parameters) of **[2**^**Pt,As**^**]**^**+**^ in MeTHF at 40 K. (d) NPA spin population of **[2**^**Pt,As**^**]**^**+**^.

The cyclic voltammogram of **2**^**Pt,As**^ (Figure 4b) features a reversible oxidation, which is anodically shifted by Δ*E*^0^ = 0.15 V with respect to P-analogue **2**^**Pt,P**^,^28^ supporting a higher lying highest occupied molecular orbital (HOMO) of the diarsene complex. In contrast to **2**^**Pt,P**^, further oxidations are fully irreversible at scan rates up to 4 V·s^–1^, preventing synthetic access to a stable dication with a neutral As≡As bridge. However, chemical oxidation of **2**^**Pt,As**^ with decamethylferrocenium enabled the isolation of the monocationic radical complex [(μ-As_2_){Pt(PNP)}_2_]^+^ (**[2**^**Pt,As**^**]**^**+**^) in 91% yield (Figure 4a). The *Q*-band EPR spectrum of **[2**^**Pt,As**^**]**^**+**^ in frozen methyltetrahydrofuran (Figure 4c) shows a rhombic signal with large *g*-anistropy (2.183, 1.959, 1.764), which reflects that of [(μ-P_2_){Pt(PNP)}_2_]^+^ with a bridging {P_2_}˙^–^ radical ligand.^28^ The notion of an As_2_-centered π-radical is further supported by large, anisotropic ^75^As hyperfine interaction, as well as quantum-chemical spin population analyses, which assign less than 10% of the spin density to the Pt atoms. Accordingly, removal of an electron from **2**^**Pt,As**^ [HOMO: π* (As_2_)] results in a hypsochromic shift of the As=As stretching vibration (Δν̃_exp_ = +38 cm^–1^) similar to the reported diphosphenyl radical (Δν̃_exp_ = +44 cm^–1^).^28^ Electrochemical characterization of the heavier metallodipnictenes **2**^**Pt,Sb**^ and **2**^**Pt,Bi**^ was prevented by their poor solubility. Nevertheless, the spectroscopic data of **[2**^**Pt,As**^**]**^**+**^ fully supports the electronic structure model obtained for the pnictide coupling products.

### Structural Characterization of the Metallopnictinidenes

Having established light-induced pnictide coupling, the putative metallopnictinidene intermediates were examined. The spectroscopic observation of CO (P, As) and H_2_ (Sb) upon irradiation of the precursor complexes supports the primary formation of metallopnictinidenes. In contrast, no ethane was detected upon photolysis of **1**^**Pt,Bi**^. Thus, direct reductive elimination can be ruled out. BiMe_3_ exhibits a propensity for radical reactions upon Me_2_Bi–Me homolysis due to a small Bi–C bond dissociation energy around 50 kcal·mol^–1^.^15^ However, the mechanism that leads to dibismuthene **2**^**Pt,Bi**^ ultimately remains unknown. The bismuth precursor was therefore not included in further examinations of transient metallopnictinidenes.

**Figure 5:**
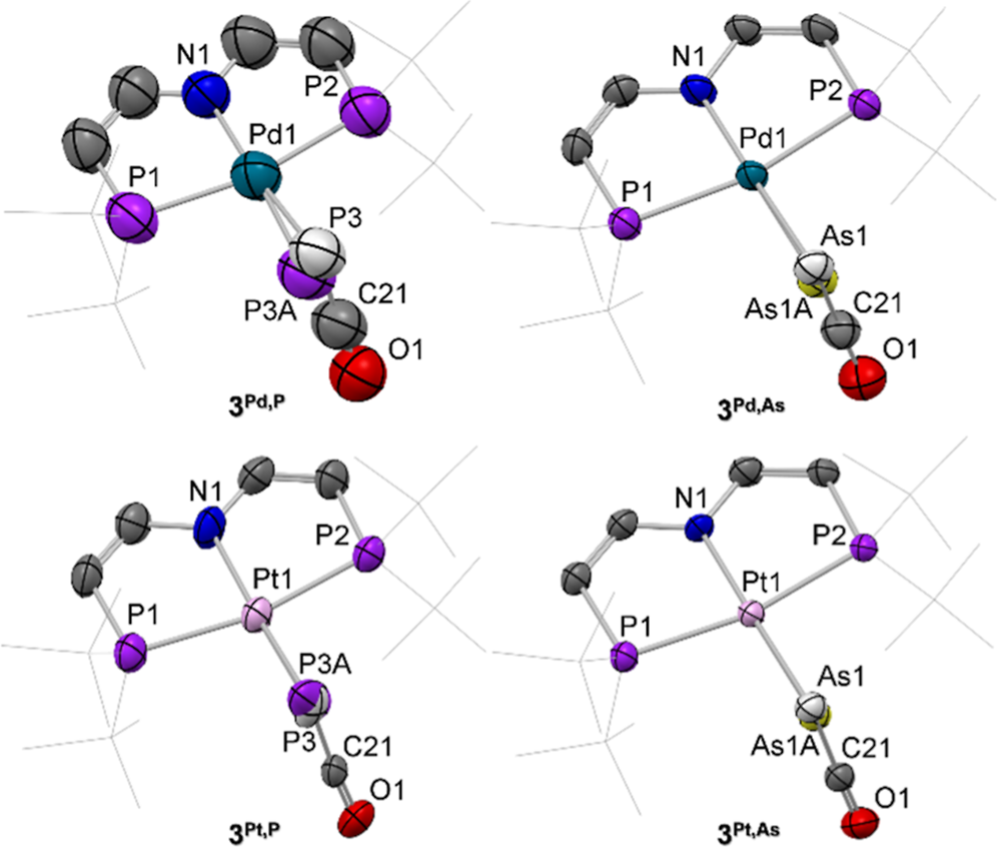
Overlays of the molecular structures of the precursor complexes and the respective metallopnictinidenes from crystal-to-crystal transformation experiments; thermal ellipsoids at the 50% probability level. H atoms are omitted and ^*t*^Bu-groups drawn as sticks for clarity. Selected bond lengths [Å]: **3**^**Pd,As**^ Pd1–As1A, 2.349(13); **3**^**Pt,P**^ Pt1–P3A, 2.25(4); **3**^**Pt,As**^ Pt1–As1A 2.36(3).

We first pursued *in crystallo* photolysis studies, which has proven effective for the structural characterization of related transient species.^27,45^ Site isolation within the crystal lattice prevents bimolecular decay as observed during solution-phase photolysis. Mounted single crystals of **1**^**Pd,P**^, **1**^**Pd,As**^, **1**^**Pt,P**^, and **1**^**Pt,As**^, respectively, were photolyzed at 100 K with an light-emitting diode (λ = 365 nm) and the diffraction data acquired with synchrotron radiation (λ = 0.41328 Å). Refinement of the data for **1**^**Pd,P**^ and **1**^**Pt,P**^ indicated about 40% photoconversion to the phosphinidenes [M(P)(PNP)] [M = Pd (**3**^**Pd,P**^), Pt (**3**^**Pt,P**^)], while the arsinidenes [M(As)(PNP)] [M = Pd (**3**^**Pd,As**^), Pt (**3**^**Pt,As**^)] were obtained in lower yields around 15% (Figure 5). Further irradiation led to sample degradation and loss of crystallinity. Due to significantly larger experimental errors, the bond metrics of **3**^**Pd,P**^ will not be discussed. All attempts to obtain suitable single crystals of the antimonide complex **1**^**Pt,Sb**^ were unfortunately unsuccessful, excluding the stibinidene from crystallographic characterization. However, only minor structural perturbation can be expected to result from H_2_ elimination, anyway.

Light-induced CO elimination is accompanied by significant contraction of the Pt–P bond length (Δ*d* = −0.11 Å) from 2.3574(16) Å (**1**^**Pt,P**^) to 2.25(4) Å (**3**^**Pt,P**^). This effect is also observed for both arsenide analogues **3**^**Pd,As**^ (Δ*d* = −0.12 Å) and **3**^**Pt,As**^ (Δ*d* = −0.21 Å). Based on Pyykkö’s covalent radii, the M–Pn bonds lengths are in the range between single and double bonds.^43^ This is consistent with our earlier reports on the analogous triplet metallonitrenes [*d*_Pd–N_(**3**^**Pd,N**^) = 1.92(2) Å, *d*_Pt–N_(**3**^**Pt,N**^) = 1.874(11) Å], which were ultimately determined to exhibit M–N single bond character.^27^ DFT optimized geometries reproduced all M–Pn bond lengths within experimental errors (Table S13).

### Electronic Structure Characterization of the Metallopnictinidenes

**Figure 6:**
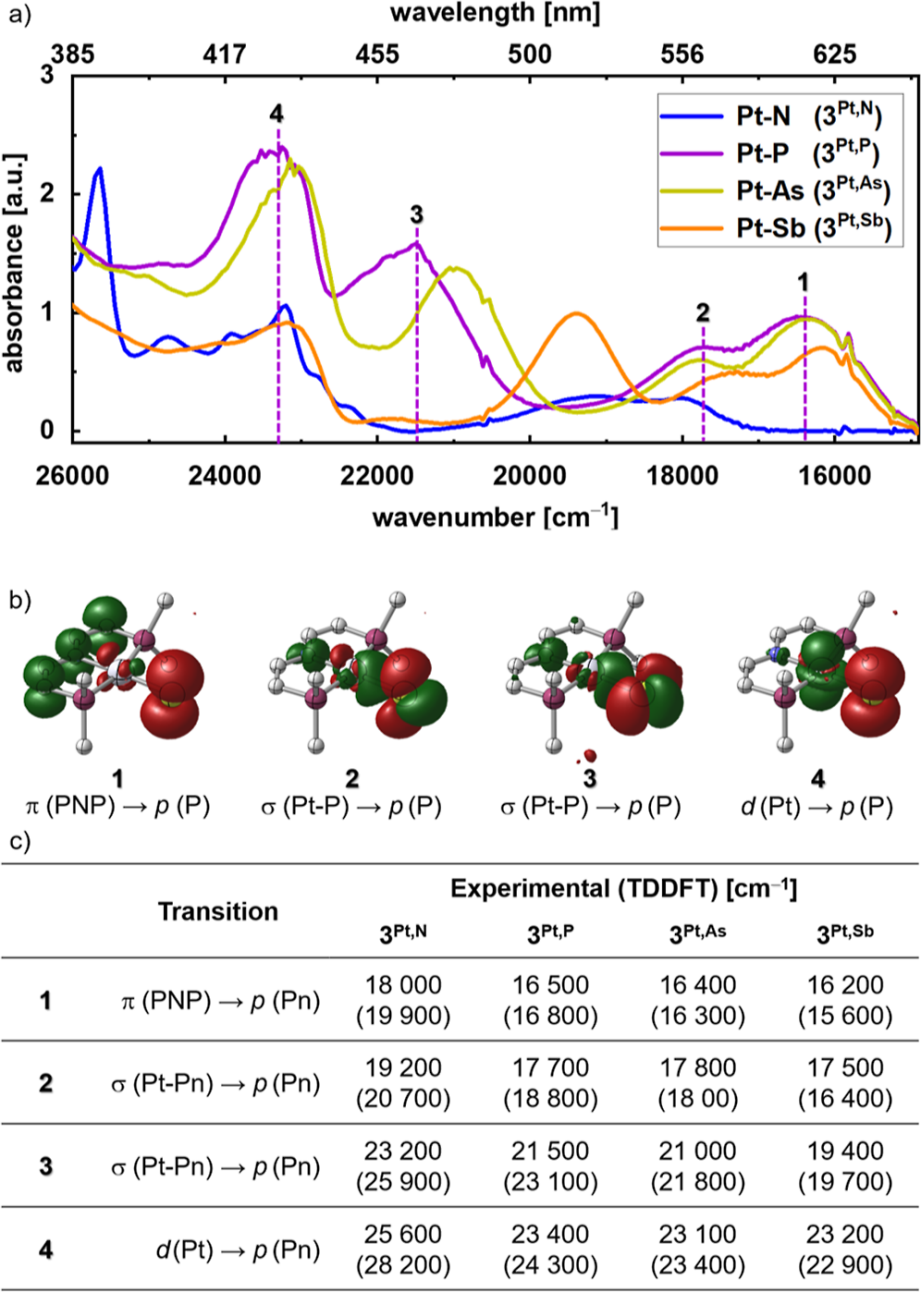
(a) UV/vis spectra of the metallopnictinidenes **3**^**Pt,Pn**^ after photolysis of the precursors **1**^**Pt,Pn**^ in frozen MeTHF at 77 K. (b) DFT computed transition difference densities (green: electron depopulation, red: electron population) of the respective transitions of **3**^**Pt,P**^. (c) Assignment of the experimental data for the series **3**^**Pt,Pn**^ (Pn = N–Sb) with TD-DFT computed data in parentheses.

To identify systematic trends in electronic structure, we first turn to electronic absorption spectroscopy. We limit the discussion in the following to the platinum pnictinidenes; all data obtained for the palladium congeners are presented as Supporting Information. The precursor complexes were photolyzed to the corresponding pnictinidenes **3**^**Pt,Pn**^ in frozen 2-methyltetrahydrofuran glass at 77 K to suppress bimolecular coupling. In all cases, irradiation results in the evolution of qualitatively similar spectra with four new absorption features in the visible region that exhibit characteristic shifts along the pnictide series (Figure 6). Upon thawing, these spectroscopic signatures rapidly decay due to Pn–Pn coupling, which is highly exergonic (cf. DFT results in Table S7). Notably, significantly higher thermal stability in solution was reported for the nitrene analogue. Decay of the UV/vis and NMR signatures of **3**^**Pt,N**^ was observed at temperatures above approximately 203 K.^27,38^ We tentatively attribute the higher solution lifetime of the nitrene to the significantly shorter Pt–N bond [1.874(11) Å] compared with the heavier homologues [e.g., Pt–P: 2.25(4) Å). In consequence, diffusion-controlled coupling is more efficiently shielded by the ^*t*^Bu groups around the nitrene.

The similarity of the electronic absorption spectra along the pnictinidene series (Pn = N–Sb) suggests analogous electronic structures. Indeed, the quantum chemical analysis indicates far reaching similarities. CASSCF(18,11)/NEVPT2 computations revealed triplet ground states with negligible multireference character for all pnictinidenes (|p_*x*_^1^p_*y*_^1^⟩ determinantal representation in the pnictogen p_π_ atomic orbital space with *z*-axis alignment of the M–Pn bond). The lowest singlet states exhibit pronounced multireference character [|p_*x*_^2^p_*y*_^0^⟩ – λ|p_*x*_^0^p_*y*_^2^⟩] and are energetically well separated from the ground state, yet with significantly smaller vertical singlet/triplet gaps (=Δ*E*_S_ – Δ*E*_T_) for the heavier pnictinidenes M–Pn (Pn = P–Bi: 12–14 kcal·mol^–1^) than for the nitrene analogues (21–23 kcal·mol^–1^, cf. Table S17). In all cases, the second exited state, with |p_*x*_^↑^p_*y*_^↓^⟩ – |p_*x*_^↓^p_*y*_^↑^⟩ singlet character throughout, is only 2–3 kcal·mol^–1^ higher in energy. By and large, state ordering of the metallopnictinidenes thus reflects that of imidogen (^3^Σ^–^, ^1^Δ, and ^1^Σ^+^), or simple pnictinidenes like Me–Pn (^3^A_2_, ^1^E, and ^1^A_1_),^5,46^ which is in line with the Salem-Rowland bonding model for heterosymmetric diradicals.^3a^

**Figure 7:**
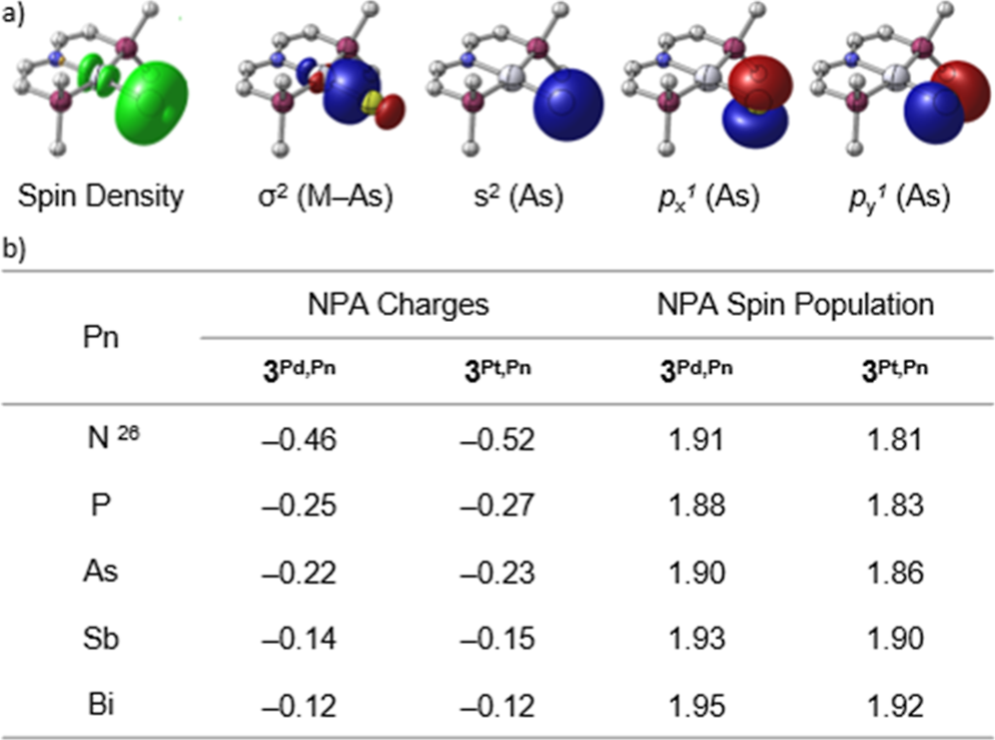
(a) DFT computed spin density of **3**^**Pt,As**^ and selected NLMOs (cf. Supporting Information for other pnictinidenes). The doubly occupied NLMOs are obtained by averaging over the α and β spin orbitals. (b) NPA charges and spin populations of the pnictide ligands of **3**^**M,Pn**^.

NBO analyses of the triplet ground states revealed M–Pn single bonding and predominant localization of the unpaired electrons in two orthogonal atomic p_Pn_ orbitals (Figure 7a). In consequence, the spin populations show toroidal distributions around the pnictogen atoms with little delocalization onto the metal ions (5–14%). In fact, spin localization at the Pn atom further increases along the pnictide series with almost complete localization in case of Sb and Bi (Figure 7b). Natural population analysis (NPA) also revealed significantly less negative NPA charges for the heavier pnictide ligands (Figure 7b), reflecting Allen’s spectroscopic electronegativity scale along the pnictogen series [configuration energies in Pauling units: 3.07 (N), 2.25 (P), 2.21 (As), 1.98 (Sb)].^47^

More detailed interpretation of the electronic absorption spectra was carried out by aid of TD-DFT simulations for the triplet pnictinidenes. The experimental data was nicely reproduced, using the B3LYP functional and a ZORA scalar-relativistic Hamiltonian along with the corresponding relativistically recontracted basis sets (Figure 6, cf. Supporting Information for details). Notably, the character of the four main transitions in the visible region does not change along the series, allowing for direct comparison. The lowest excitation with dominant π(PNP) → p(Pn) ligand-to-ligand charge transfer character (Figure 6b, **1**) proved particularly instructive, as the pincer-based donor orbital energy is system independent. Thus, the red-shift of this transition for all heavier pnictide complexes with respect to the nitrene (Δν̃ ≈ 1700 cm^–1^) directly indicates a significant lowering of their SOMO energies.

### Magnetic Characterization of the Metallopnictinidenes

**Figure 8:**
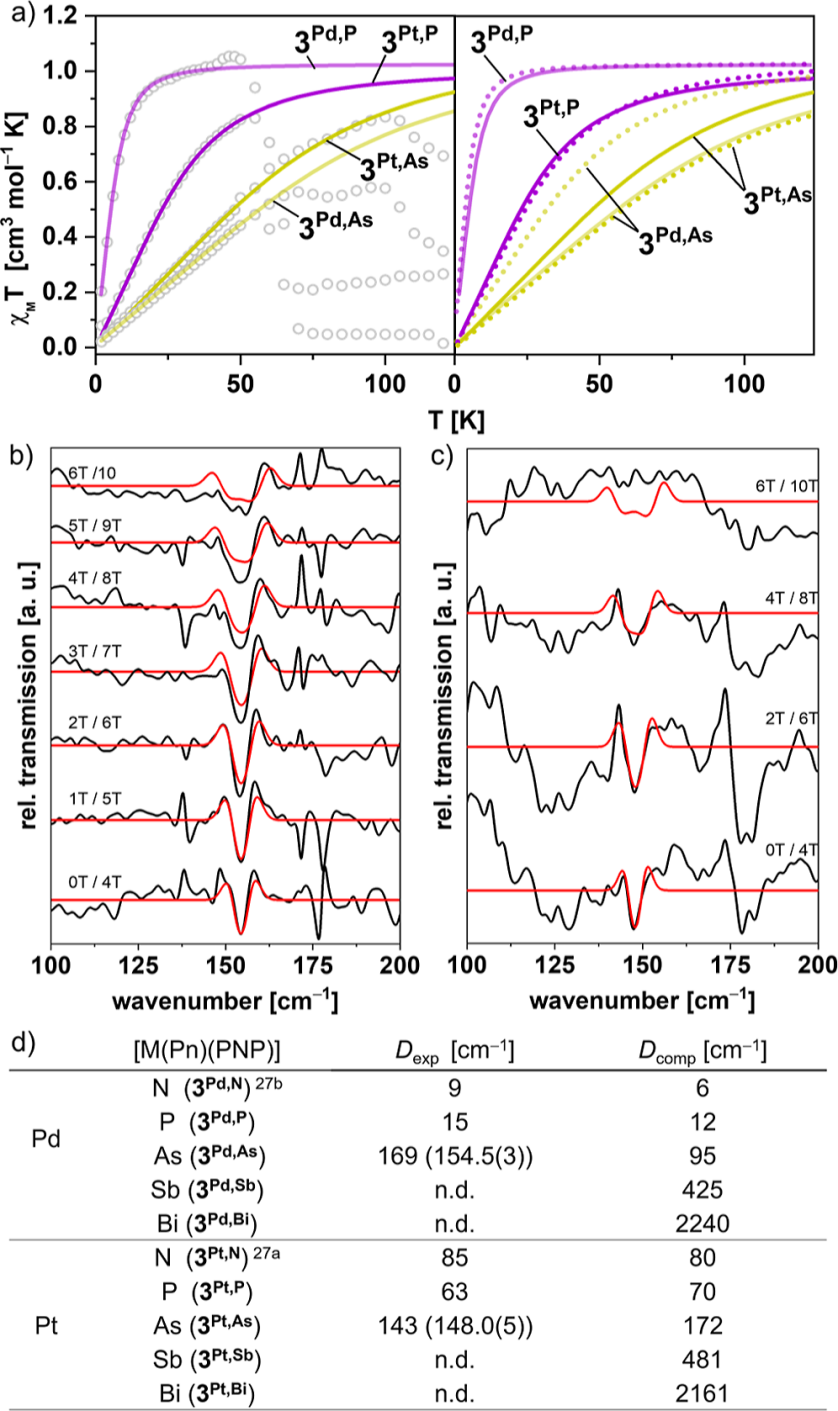
(a) SQUID magnetometric characterization of *in situ* formed **3**^**Pd,P**^, **3**^**Pd,As**^ and **3**^**Pt,P**^, **3**^**Pt,As**^; left: experimental (circles) and simulated (colored lines) χ_m_*T* vs *T* data; right: comparison of simulated (colored lines) and *ab initio* computed (dotted lines) χ_m_*T* vs *T* data. (b) THz-EPR data (black lines) and numerical simulations (red lines; cf. Supporting Information for details) of **3**^**Pd,As**^. (c) THz-EPR data (black lines) and numerical simulations (red lines; cf. Supporting Information for details) of **3**^**Pt,As**^. (d) Comparison of axial zero-field splitting parameters *D* from SQUID magnetometry and THz-EPR (in paratheses) with *ab initio* computed values (cf. Supporting Information for details).

Relativistic effects were evaluated by examination of the magnetic properties with *in situ* SQUID magnetometry. Continuous photolysis of solid **1**^**Pd,P**^, **1**^**Pd,As**^, **1**^**Pt,P**^, and **1**^**Pt,As**^ at 5 K results in constant rise of the magnetization (Figures S52–S59) due to formation of the respective paramagnetic pnictinidene photoproducts. The χ_m_*T* products of **3**^**Pd,P**^, **3**^**Pd,As**^, **3**^**Pt,P**^, and **3**^**Pt,As**^ (Figure 8a) rise with temperature and sharply drop beyond 50–100 K, indicating sample decay. The apparently higher sample stability during single crystal photolysis experiments (100 K), might be caused by lower CO gas mobility within the larger crystals, but ultimately remains unknown. In consequence, the accessible temperature ranges for magnetic characterization were markedly smaller than for the nitrogen analogues. Nevertheless, pronounced deviation from purely temperature independent paramagnetism allowed for reliably fitting the conversion-normalized magnetic data of **3**^**Pd,P**^, **3**^**Pt,P**^, and **3**^**Pt,As**^ to a ZFS spin-Hamilitonian (sH) for spin triplet ground states (*S* = 1) and isotropic *g*-factors fixed to *g* = 2 (Figure 8a). The low magnetic moment of the sample after photolysis of **1**^**Pt,Sb**^ prohibited meaningful simulation of the magnetic data.

Due to the limited temperature range of the magnetic data, variable field (0–10 T) *in situ* THz-EPR spectroscopy was used to determine the ZFS of *in situ* photogenerated **3**^**Pd,As**^ and **3**^**Pt,As**^ (λ_exc_ = 532 nm) directly from the transitions between the magnetic sublevels at 5 K. Notably, fitting of the field-dependent data to a ZFS-sH (Figure 8b,c) gave axial ZFS parameters *D* that are in nice agreement with the SQUID simulations (Figure 8d). Furthermore, the data confirmed *g* values close to 2 and negligible rhombicity in the ZFS.

The axial ZFS parameters of both metallophoshinidenes are close to those of the respective nitrene analogues but vary significantly with the nature of the metal substituent. Furthermore, the values are markedly higher than the ZFS that was calculated by Michl and co-workers for simple organic nitrenes and phosphinidenes, like Me–Pn [Pn = N, P; *D*/cm^–1^ = 1.8 (N), 3.5 (P)].^6b^ This finding supports the notion that magnetic sublevel splitting of the lighter pnictinidenes (N, P) is dominated by metal-induced spin–orbit coupling (SOC) of the ground and excited states. Notably, the ZFS slightly drops from **3**^**Pt,N**^ to the heavier **3**^**Pt,P**^ (Figure 8d), albeit the excited states of the phosphinidene are significantly lower in energy (see above). We tentatively attribute this observation to overcompensation from decreased Pt–P orbital overlap and, thus, lower effective SOC.

In turn, the arsinidenes exhibit substantially higher ZFS parameters, while the difference between the two transition metal platforms almost vanishes. We note in passing, that the ZFS parameters of the metalloarsinidenes are closer to the value computed for Me–As (*D*/cm^–1^ = 86.5),^6b^ as compared with the phosphorus analogues. Quantum-chemical expansion of the magnetic data toward the heaviest pnictinidenes (Sb, Bi), which were so far not accessible experimentally, predicts further rising *D* values and ultimately negligible differences between the two transition metal platforms for **3**^**Pd,Bi**^ and **3**^**Pt,Bi**^ (Figure 8d). Note that the axial ZFS computed for the metallobismuthinidenes is of similar magnitude as the energetic separation that Neese and Cornella found for the nonmagnetic ground state of their isolable triplet bismuthinidene (*D* > 5400 cm^–1^; Figure 1b). Thus, for the arsinidene and beyond the splitting of the magnetic microstates is dominated by pnictogen-induced SOC effects. This interpretation is in line with exclusive localization of the spin orbitals at the pnictide ligand, as supported by the NBO analysis.

## Conclusions

In continuation of our report about the meatllonitrenes [M(N)(PNP)] (M = Pd, Pt),^27^ we have characterized the heavier metallopnictinidenes [M(Pn)(PNP)] (Pn = P–Sb) as transient precursors to bimolecular pnictide coupling. In addition to the common pnictaenthynolate precursors for the introduction of atomic P and As ligands, the parent antimonide, SbH_2_^–^, proved effective for stibinidene generation upon UV-induced H_2_ elimination. In analogy to the metallonitrenes, the spectroscopic, magnetic, and computational analysis confirmed predominantly pnictide-centered diradicals with triplet ground states. Beyond N and P, excited state admixture through spin–orbit coupling is predominantly induced by the heavy pnictide atom.

Importantly, the electronic structure examinations revealed some trends of the heavier pnictides versus their nitrogen analogues with potential relevance for chemical reactivity. First, the lowest singlet excited states are all at significantly lower energy. Thus, reactions that require crossover onto the singlet surface might proceed with markedly smaller electronic reorganization energies. Note that Bertrand’s group showed that singlet phosphinidenes exhibit distinctly electrophilic reactivity.^19b^ Furthermore, the lowest SOMOs of the heavier pnictinidenes feature lower energies than those of the nitrenes. This observation also suggests increased electrophilicity in radical reactions, which contrasts with the distinct nucleophilicity of the metallonitrenes.^28,38^ Future studies will focus on the exploration of these predictions for chemical synthesis.

## Supporting Information Available

Experimental procedures, spectroscopic data and computational details for the discussed structures (PDF)
Cartesian coordinates (TXT)
