# Supplementary material for: Transient Triplet Metallopnictinidenes M–Pn (M = PdII, PtII; Pn = P, As, Sb): Characterization and Dimerization
Source: J Am Chem Soc. 2025 Jan 29;147(6):5330–9. doi: 10.1021/jacs.4c16830 (PMC11826990; doi:10.1021/jacs.4c16830)
Supplement: Supplementary file 1 — ja4c16830_si_001.pdf [file ja4c16830_si_001.pdf]

Supporting Information for

Transient Triplet Metallopnictinidenes M–Pn (M = Pd^{II}, Pt^{II}; Pn = P, As, Sb): Characterization and Dimerization

Marc C. Neben,[a] Nils Wegerich,[b] Tarek A. Al Said,[c,d] Richard R. Thompson,[e] Serhiy Demeshko,[a] Kevin Dollberg,[f] Igor Tkach,[g] Gerard P. Van Trieste III,[h] Hendrik Verplancke,[b] Carsten von Hänisch,*[f] Max C. Holthausen,*[b] David C. Powers,*[h] Alexander Schnegg,*[d] and Sven Schneider*[a]

[a] Institut für Anorganische Chemie and International Center for Advanced Studies of Energy Conversion, Georg-August-Universität Göttingen, Tammannstr. 4, 37077 Göttingen (Germany).

[b] Institut für Anorganische und Analytische Chemie Goethe-Universität, Max-von-Laue-Strasse7, 60438 Frankfurt am Main (Germany).

[c] Helmholtz-Zentrum Berlin für Materialien und Energie GmbH, Hahn-Meitner-Platz 1, 14109 Berlin (Germany).

[d] EPR Research Group, MPI for Chemical Energy Conversion, Stiftstrasse 34–36, 45470 Mülheim Ruhr (Germany).

[e] Department of Chemistry, University of Idaho – Moscow Campus, Moscow, ID 83844 (USA)

[f] Fachbereich Chemie, Philipps-Universität Marburg Hans-Meerwein-Straße 4, 35043 Marburg (Germany).

[g] RG ESR Spectroscopy, Max Planck Institute for Multidisciplinary Sciences, Am Faßberg 11, 37077 Göttingen (Germany).

[h] Department of Chemistry,Texas A&M University, College Station,TX 77843 (USA).

Table of Content

Experimental Procedures 3

Materials and Methods 3

Syntheses 3

[(PNP)Pd-PCO] ($1^{Pd,P}$) 3

[(PNP)Pd-AsCO] ($1^{Pd,As}$) 3

[(PNP)Pt-AsCO] ($1^{Pt,As}$) 4

[(PNP)Pt-SbH$_2$] ($1^{Pt,Sb}$) 4

[(PNP)Pt-BiMe$_2$] ($1^{Pt,Bi}$) 4

[(μ-P$_2$){(PNP)Pd}$_2$] ($2^{Pd,P}$) 4

[(μ-As$_2$){(PNP)Pd}$_2$] ($2^{Pd,As}$) 5

[(μ-As$_2$){(PNP)Pt}$_2$] ($2^{Pt,As}$) 5

[(μ-Sb$_2$){(PNP)Pt}$_2$] ($2^{Pt,Sb}$) 5

[(μ-Bi$_2$){(PNP)Pt}$_2$] ($2^{Pt,Bi}$) 5

[(μ-As$_2$){(PNP)Pt}$_2$]BArF$_{24}$ ([$2^{Pt,As}$]$^+$) 5

Spectroscopic Characterization of [(PNP)Pd-PCO] ($1^{Pd,P}$) 6

Spectroscopic Characterization of [(PNP)Pd-AsCO] ($1^{Pd,As}$) 8

Spectroscopic Characterization of [(PNP)Pt-AsCO] ($1^{Pt,As}$) 11

Spectroscopic Characterization of [(PNP)Pt-SbH$_2$] ($1^{Pt,Sb}$) 14

Spectroscopic Characterization of [(PNP)Pt-BiMe$_2$] ($1^{Pt,Bi}$) 17

Spectroscopic Characterization of [(*μ*-P$_2$){(PNP)Pd}$_2$] ($2^{Pd,P}$) 18

Spectroscopic Characterization of [(*μ*-As$_2$){(PNP)Pd}$_2$] ($2^{Pd,As}$) 20

(Spectroscopic) Characterization of [(*μ*-As$_2$){(PNP)Pt}$_2$] ($2^{Pt,As}$) 22

Spectroscopic Characterization of [(*μ*-Sb$_2$){(PNP)Pt}$_2$] ($2^{Pt,Sb}$) 26

Spectroscopic Characterization of [(*μ*-Bi$_2$){(PNP)Pt}$_2$] ($2^{Pt,Bi}$) 29

Spectroscopic Characterization of [(*μ*-As$_2$){(PNP)Pt}$_2$]BArF$_{24}$ ([$2^{Pt,As}$]$^+$) 29

***In situ* SQUID measurements** 33

THz-EPR Spectroscopy 37

Sample preparation and measurement conditions 37

Mid-IR reaction monitoring of the photoreactions 38

THz-EPR measurements 39

In situ UV-vis studies 41

Computational Details 44

Electronic structures of the precursors $1^{M,Pn}$ 45

Computed UV-vis spectra of Precursors $1^{M,Pn}$ 46

Electronic structures of dipnictenes $2^{M,Pn}$ 48

Computed UV-vis spectra of Dipnictenes $2^{M,Pn}$ 51

Computed UV-vis spectra of Diaryldipnictenes $2^{Ar,Pn}$ (Ar: C$_6$H$_3$-2,6-Mes$_2$; Pn: P, As, Sb, Bi) 53

Electronic Structure of Dipnictenyl radical cations [$2^{M,Pn}$]$^+$ 54

Electronic Structure of Metallopnictinidenes $3^{M,Pn}$ 55

NEVPT2/CASSCF Computations on Metallopnictinidenes $3^{M,Pn}$ 56

Computed UV-vis spectra of Metallopnictinidenes $3^{M,Pn}$ 59
Detailed computational results 62
Crystallographic Details 63
X-ray Single-Crystal Structure Analysis of [Pd(PCO)(PNP)] ($1^{Pd,P}$) 64
X-ray Single-Crystal Structure Analysis of [Pd(AsCO)(PNP)] ($1^{Pd,As}$) 67
X-ray Single-Crystal Structure Analysis of [Pt(AsCO)(PNP)] ($1^{Pt,As}$) 70
X-ray Single-Crystal Structure Analysis of [Pt(BiMe$_2$)(PNP)] ($1^{Pt,Bi}$) 73
X-ray Single-Crystal Structure Analysis of [(μ-P$_2$){Pd(PNP)}$_2$] ($2^{Pd,P}$) 75
X-ray Single-Crystal Structure Analysis of [(μ-As$_2$){Pd(PNP)}$_2$] ($2^{Pd,As}$) 79
X-ray Single-Crystal Structure Analysis of [(μ-As$_2$){Pt(PNP)}$_2$] ($2^{Pt,As}$) 82
X-ray Single-Crystal Structure Analysis of [(μ-Sb$_2$){Pt(PNP)}$_2$] ($2^{Pt,Sb}$) 85
X-ray Single-Crystal Structure Analysis of [(μ-Bi$_2$){Pt(PNP)}$_2$] ($2^{Pt,Bi}$) 88
X-ray Single-Crystal Structure Analysis of [Pd(P)(PNP)] ($3^{Pd,P}$) 90
X-ray Single-Crystal Structure Analysis of [Pd(As)(PNP)] ($3^{Pd,As}$) 93
X-ray Single-Crystal Structure Analysis of [Pt(P)(PNP)] ($3^{Pt,P}$) 96
X-ray Single-Crystal Structure Analysis of [Pt(As)(PNP)] ($3^{Pt,As}$) 99
References 102

Experimental Procedures

Materials and Methods

All experiments and measurements were carried out under inert conditions (argon atmosphere) using glovebox or standard Schlenk techniques. PR160(L) LED lamps (370, 456 and 525 nm) from Kessil (DiCon Fiberoptics & DiCon Lighting, Richmond, CA, USA) were used for all photolysis experiments. Bulk photolysis was carried out upon irradiation of samples that were immersed in ^{i}PrOH baths cooled by cryogenic circulation system. Solvents were purchased from Sigma-Aldrich in HPLC quality and pre-dried using a Solvent Purification System (SPS) purchased from MBRAUN. All solvents were further dried prior to use over Na/K alloy or CaH_2 and distilled by *trap-to-trap* transfer. Deuterated solvents (C_6D_6, toluene-d_8) were purchased from Euriso-top GmbH, dried over Na/K alloy, distilled by *trap-to-trap* transfer, and degassed by two *freeze-pump-thaw* cycles. [(PNP)Pd-OTf],[1] [(PNP)Pd-N_3],[1] [(PNP)Pt-OTf],[2] [(PNP)Pt-PCO],[3] [Na(diox)$_n$][PCO],[4] [Na(diox)$_n$][AsCO],[5] Fc*[BArF$_{24}$],[6] and $BiMe_3$[7] were prepared according to literature procedures. [K(THF)(18crown-6)]SbH_2 was synthesized according to the published procedure.[8] All other chemicals used were purchased from chemical vendors and used without further purification.

NMR spectra were recorded with Bruker Avance III HD 300, Avance III HD 400, Avance Neo 400, or Avance III HD 500 spectrometers and referenced to residual solvent signals (C_6D_6: δ_H = 7.16 ppm, δ_C = 128.4 ppm; toluene-d_8: δ_H = 2.09 ppm, δ_C = 20.4 ppm; THF-d$_8$: δ_H = 3.58 ppm, δ_C = 67.2 ppm). Spectra were recorded at room temperature unless otherwise stated. Signal multiplicity abbreviations: s (singlet), d (doublet), t (triplet), q (quartet), quint (quintet), m (multiplet), dd (doublet of doublets), dt (doublet of triplets), td (triplet of doublets), vt (virtual triplet), br (broad). All samples were prepared under inert conditions in an argon glovebox and recorded in *J-Young* NMR tubes. Elemental analyses were carried out by the analytical laboratories at the Georg-August University using an Elementar Vario El 3 analyzer. LIFDI mass spectra were obtained with a Joel AccuTOF spectrometer under inert conditions by the Zentrale Massenabteilung of the Georg-August-University. IR spectra were recorded with a Bruker ALPHA FT-IR spectrometer featuring a Platinum ATR module. UV-vis samples were prepared under inert conditions in an argon glovebox in air-tight 1cm quartz cuvettes with attached *J-Young* screw caps and recorded with Agilent Cary 8454 or Cary 60 spectrophotometers, respectively. Low-temperature UV-vis spectra were obtained with a USP-203 Series variable temperature unit from Unisoku (+100 to −195 °C). For measurements below −180 °C, the samples were prepared in septum capped reinforced quartz cuvettes (Hellma GmbH, Müllheim, Germany). Cyclic voltammograms were measured using a GAMRY 600 reference potentiostat in 0.1 M [NBu_4]BArF$_{24}$ electrolyte solutions under N_2 atmosphere. The data was obtained with Ag/Ag$^+$ reference, glassy carbon working, and Pt wire counter electrodes and internally referenced against the Fc$^+$/Fc redox couple. Solution magnetic moments at room temperature were determined by Evan's method, as modified by Sur, and corrected for diamagnetic contributions.[9,10] Experimental procedures for *in situ* SQUID measurements are presented in the respective chapter. Q-Band continuous wave EPR measurements were performed on a Bruker ElexSys E580 EPR spectrometer equipped with a Bruker ER5106QT TE$_{011}$-mode cylindrical resonator (3 mm O.D. sample tubes) in a CF935 helium gas flow cryostat (Oxford Instruments, Abingdon, UK) at 40 K. For EPR simulations the *pepper* function of the EasySpin software toolbox[11] was used. Euler angles are used in the zy'z'' convention and describe a subsequent rotation of the frame: first by α about the z axis, yielding the frame x'y'z', then by β about the y' axis, yielding the frame x''y''z'', and last by γ about the z'' axis yielding the final frame. Positive angles refer to counterclockwise rotation. Experimental procedures for *in situ* THz EPR measurements are presented in the respective chapter. Resonance raman spectra were measured using a Horiba Jobin Yvon LabRAM HR 800 Vis with 633 nm excitation wavelength.

Syntheses

[(PNP)Pd-PCO] ($1^{Pd,P}$)

[(PNP)Pd-OTf] (130 mg, 212 µmol, 1.00 eq.) and [Na(diox)$_{2.37}$][PCO] (123 mg, 425 µmol, 2.00 eq.) are dissolved in THF at −40 °C and stirred for 5 min under the exclusion of light. The mixture is allowed to warm to room temperature and filtered. After removal of the solvent *in vacuo*, the residue is extracted with hexane (3 x 1 mL). Subsequent evaporation of the solvent yields a reddish purple solid (105 mg, 202 µmol, 95%) in analytical purity.
^{1}H NMR (C_6D_6, 400 MHz, [ppm]): δ = 6.85 (ABXX'A'B', N = $|^3J_{HP} + {}^4J_{HP}|$ = 43.3 Hz $^3J_{HH}$ = 5.0 Hz, 2H, NC*H*), 3.84 (ABXX'A'B', N = $|^2J_{HP}+{}^4J_{HP}|$ = 9.4 Hz $^3J_{HH}$ = 5.2 Hz, 2H, C*H*P), 1.35 (A$_{18}$BXX'A'$_{18}$B', N = $|^3J_{HP} + {}^5J_{HP}|$ = 14.3 Hz, 36H, P(C(CH_3)$_3$)$_2$). **^{13}C{^{1}H} NMR** (C_6D_6, 100 MHz, [ppm]): δ = 167.4 (d, $^1J_{PC}$ = 93.5 Hz, 1C P*C*O), 160.0 (t, $^2J_{PC}$ = 8.7 Hz, 2C, N*C*H), 82.2 (td, $^1J_{PC}$ = 17.9 Hz, $^3J_{PC}$ = 2.1 Hz, 2C, *C*HP), 36.5 (t, $^1J_{PC}$ = 9.9 Hz, 4C, P(*C*(CH$_3$)$_3$)$_2$), 29.2 (q, $^2J_{PC}$ = 3.0 Hz, 12C, P(C(*C*H$_3$)$_3$)$_2$). **^{31}P{^{1}H} NMR** (C_6D_6, 162 MHz, [ppm]): δ = 74.9 (d, $^2J_{PP}$ = 22.4 Hz, 2P, *P*C(CH$_3$)$_3$)$_2$), – 384.3 (t, $^2J_{PP}$ = 22.4 Hz, 1P, *P*CO). **IR (ATR):** 1863 (PC=O), 1533 (C=C) cm^{-1}. **UV-Vis (THF):** λ_{max} (ε/M^{-1} cm^{-1}) = 260 (67690), 319 (26596), 533 (2518) nm. **LIFDI-MS**: m/z = 521.6 (100). **Elem. Anal.:** Calcd. $C_{21}H_{40}NOP_3Pd$ (521.9) C, 48.33; H 7.73; N, 2.68. Found: C, 48.43; H, 7.71; N, 2.65.

[(PNP)Pd-AsCO] ($1^{Pd,As}$)

[(PNP)PdOTf] (120 mg, 196 µmol, 1.00 eq.) and [Na(diox)$_{3.50}$][AsCO] (162 mg, 373 µmol, 1.90 eq.) are stirred in diethylether at −40 °C for 16 hours under the exclusion of light. Workup is carried out at −40 °C by filtration and solvent removal *in vacuo*. Extraction with cold pentane (−40 °C, 4 x 0.5 mL) and subsequent evaporation of the solvent yields a dark purple solid (104 mg, 184 µmol, 94 %) with analytical purity.

^{1}H NMR (Tol-d_8, –35 °C, 500 MHz, [ppm]): δ = 6.80 (ABXX'A'B', N = |$^3J_{HP}$ + $^4J_{HP}$| = 42.9 Hz, $^3J_{HH}$ = 5.2 Hz, 2H, NC*H*), 3.81 (ABXX'A'B', N = |$^2J_{HP}$+$^4J_{HP}$| = 9.5 Hz, $^3J_{HH}$ = 5.2 Hz, 2H, C*H*P), 1.35 (A_{18}BXX'A'$_{18}$B', N = |$^3J_{HP}$ + $^5J_{HP}$| = 14.2 Hz, 36H, P(C(C*H*$_3$)$_3$)$_2$). **^{13}C{^{1}H} NMR** (Tol-d_8, –35 °C, 125 MHz, [ppm]): δ = 175.6 (br, 1C, As*C*O), 160.2 (t, $^2J_{PC}$ = 8.7 Hz, 2C, N*C*H), 82.4 (t, $^1J_{PC}$ = 18.1 Hz, 2C, *C*HP), 36.8 (t, $^1J_{PC}$ = 10.0 Hz, 4C, P(*C*(CH$_3$)$_3$)$_2$), 29.4 (t, $^2J_{PC}$ = 3.1 Hz, 12C, P(C(*C*H$_3$)$_3$)$_2$). **^{31}P{^{1}H} NMR** (Tol-d_8, –35 °C, 202 MHz, [ppm]): δ = 73.4 (s, 2P, *P*C(CH$_3$)$_3$)$_2$). **IR (ATR):** 1850 (AsC=O), 1533 (C=C) cm^{-1}. **UV-Vis (THF):** λ_{max} (ε/M^{-1} cm^{-1}) = 264 (66049), 324 (28859), 570 (1608) nm. **LIFDI-MS:** m/z = 565.3 (100). **Elem. Anal.:** Calcd. $C_{21}H_{40}AsNOP_2Pd$ (565.8) C, 44.58; H, 7.13; N, 2.48. Found: C, 45.05; H, 7.29; N, 2.41.

[(PNP)Pt-AsCO] (1^{Pt,As})

[(PNP)Pt-OTf] (75.0 mg, 107 µmol, 1.00 eq.) and [Na(dioxane)$_{4.16}$][AsCO] (59.0 mg, 192 µmol, 1.8 eq.) are stirred in diethylether (3 mL) for 16 hours at –40 °C under the exclusion of light. The resulting reddish solution is filtered at –40 °C and the solvent is removed *in vacuo*. Extraction with cold pentane (4 x 1 mL) under exclusion of light into a brown flask resulted in a light and temperature sensitive red to pink solid (61.2mg, 93.5 µmol, 87%) of sufficient purity for further use.
^{1}H NMR (Tol-d_8, –35 °C, 600 MHz, [ppm]): δ = 6.78 (ABXX'A'B', N = |$^3J_{HP}$ + $^4J_{HP}$| = 38.0 Hz $^3J_{HH}$ = 5.4 Hz, $^3J_{PtH}$ = 77.5 Hz, 2H, NC*H*), 3.98 (ABXX'A'B', N = |$^2J_{HP}$+$^4J_{HP}$| = 9.1 Hz, $^3J_{HH}$ = 5.4 Hz, $^3J_{PtH}$ = 24.6 Hz, 2H, C*H*P), 1.36 (A_{18}BXX'A'$_{18}$B', N = |$^3J_{HP}$ + $^5J_{HP}$| = 14.2 Hz, 36H, P(C(C*H*$_3$)$_3$)$_2$). **^{13}C{^{1}H} NMR** (Tol-d_8, –35 °C, 150 MHz, [ppm]): δ = 185.4 (t, $^3J_{PC}$ = 2.1 Hz, 1C, As*C*O), 161.4 (t, $^2J_{PC}$ = 7.4 Hz, $^2J_{PtC}$ = 73.6 Hz, 2C, N*C*H), 84.1(t, $^1J_{PC}$ = 22.5 Hz, 2C, *C*HP), 36.9 (t, $^1J_{PC}$ = 13.4 Hz, 4C, P(*C*(CH$_3$)$_3$)$_2$), 30.0 (t, $^2J_{PC}$ = 2.4 Hz, $^3J_{PtC}$ = 13.6 Hz, 12C, P(C(*C*H$_3$)$_3$)$_2$). **^{31}P{^{1}H} NMR** (Tol-d_8, –35 °C, 243 MHz, [ppm]): δ = 61.3 (s, $^1J_{PtP}$ = 2596.2 Hz, 2P, *P*C(CH$_3$)$_3$)$_2$). **^{195}Pt{^{1}H} NMR** (Tol-d_8, , –35 °C, 107 MHz, [ppm]): δ = – 4644.3 (t, $^1J_{PPt}$ = 2604.2 Hz). **IR (ATR, cm^{-1}):** 1859 (AsC=O), 1533 (C=C). **UV-Vis (THF):** λ_{max} (ε, M^{-1} cm^{-1}) = 235 (57649), 318 (37341), 517 (603) nm. **LIFDI-MS:** m/z = 654.2 (100). **Elem. Anal.:** Calcd. $C_{21}H_{40}AsNOP_2Pt$ (654.5) C, 38.54; H, 6.16; N, 2.14. Found: C, 39.23; H, 6.25; N, 2.09.

[(PNP)Pt-SbH$_2$] (1^{Pt,Sb})

[(PNP)Pt-OTf] (150 mg, 214 µmol, 1.00 eq.) and [K(THF)(18crown-6)]SbH$_2$ (320 mg, 624 µmol, 3.00 eq.) are stirred in diethylether (10 mL) for 16 hours at –40 °C under the exclusion of light. The suspension is filtered and the solvent removed *in vacuo*. Extraction with cold pentane (3 x 4 mL), removal of the solvent and subsequent washing with cold hexamethyldisiloxane (2 x 1 mL) resulted in an off-white solid (114 mg, 169 µmol, 79 %). Traces of residual 18crown-6 can be removed by additional washing with cold hexamethyldisiloxane which leads to a significant decrease in yield.
^{1}H NMR (C_6D_6, 500 MHz, [ppm]): δ = 7.01 (ABXX'A'B', N = |$^3J_{HP}$ + $^4J_{HP}$| = 30.6 Hz, $^3J_{HH}$ = 5.5 Hz, $^3J_{PtH}$ = 61.3 Hz, 2H, NC*H*), 3.81 (ABXX'A'B', N = |$^2J_{HP}$+$^4J_{HP}$| = 9.3 Hz $^3J_{HH}$ = 5.4 Hz, $^3J_{PtH}$ = 21.8 Hz, 2H, C*H*P), 1.35 (A_{18}BXX'A'$_{18}$B', N = |$^3J_{HP}$ + $^5J_{HP}$| = 14.2 Hz, 36H, P(C(C*H*$_3$)$_3$)$_2$), – 0.94 (t, $^3J_{HP}$ = 8.4 Hz, $^3J_{HPt}$ = 60.3 Hz, 2H, Sb*H*$_2$). **^{13}C{^{1}H} NMR** (C_6D_6, 125 MHz, [ppm]): δ = 159.9 (t, $^3J_{PC}$ = 7.4 Hz, $^2J_{PtC}$ = 63.4 Hz, 2C, N*C*H), 82.4 (t, $^1J_{PC}$ = 9.6 Hz, $^2J_{PtC}$ = 45.8 Hz, *C*HP), 36.5 (t, $^1J_{PC}$ = 13.5 Hz, $^2J_{PtC}$ = 48.4 Hz, 4C, P(*C*(CH$_3$)$_3$)$_2$), 29.4 (t, $^2J_{PC}$ = 2.9 Hz, $^3J_{PtC}$ = 14.1 Hz, 12C, P(C(*C*H$_3$)$_3$)$_2$). **^{31}P{^{1}H} NMR** (C_6D_6, 202 MHz, [ppm]): δ = 67.7 (s, $^1J_{PtP}$ = 2640.1 Hz, 2P, *P*C(CH$_3$)$_3$)$_2$). **^{195}Pt{^{1}H} NMR** (C_6D_6, 107 MHz, [ppm]): δ = – 4980.4 (t, $^1J_{PPt}$ = 2637.6 Hz). **IR (ATR):** (ATR): 1834 (Sb-H), 1530 (C=C) cm^{-1}. **UV-Vis (THF):** (THF): λ_{max} (ε, M^{-1} cm^{-1}) = 318 (1958) nm. **LIFDI-MS**: m/z = 675.2 (100). **Elem. Anal.:** Calcd. $C_{20}H_{42}NP_2PtSb$ (675.4) C, 35.57; H, 6.27; N, 2.07. Found: C, 36.05; H, 6.29; N, 1.99.

[(PNP)Pt-BiMe$_2$] (1^{Pt,Bi})

BiMe$_3$ (15.5 µL, 141 µmol, 3.90 eq) is added to a mixture of potassium (10.0mg, 256 µmol, 7.14 eq) and 18-cr-6 (67.6 mg, 256 µmol, 7.14 eq.) in THF at −40 ◦C and stirred for overnight. [(PNP)Pt-OTf] (25 mg, 35.7 µmol, 1.00 eq.) is added to the resulting dark brown solution and stirred over night at −40 °C. The solvent is removed *in vacuo* and the residue extracted with cold pentane. Solvent evaporation results in a dark yellow oil. Crystals suitable for XRD measurements are obtained from a concentrated pentane solution. NMR spectroscopic characterization shows small amounts of unknown impurities and residual 18-cr-6.
^{1}H NMR (C_6D_6, 400 MHz, [ppm]): δ = 7.08 (ABXX'A'B', N = |$^3J_{HP}$ + $^4J_{HP}$| = 35.8 Hz, $^3J_{HH}$ = 5.4 Hz, $^3J_{PtH}$ = 53.8 Hz, 2H, NC*H*), 4.09 (ABXX'A'B', N = |$^2J_{HP}$+$^4J_{HP}$| = 9.6 Hz $^3J_{HH}$ = 5.3 Hz, $^3J_{PtH}$ = 25.2 Hz, 2H, C*H*P),), 1.97 (s, $^3J_{PtH}$ = 28.6 Hz , 3H, Bi(C*H*$_3$)$_2$),1.31 (A_{18}BXX'A'$_{18}$B', N = |$^3J_{HP}$ + $^5J_{HP}$| = 14.2 Hz, 36H, P(C(C*H*$_3$)$_3$)$_2$). **^{31}P{^{1}H} NMR** (C_6D_6, 162MHz, [ppm]): δ = 66.25 (d, $^2J_{PP}$ = 4.9 Hz, $^1J_{PtP}$ = 2856.7 Hz, 1P, *P*C(CH$_3$)$_3$)$_2$), 66.21 (d, $^2J_{PP}$ = 4.9 Hz, $^1J_{PtP}$ = 2856.7 Hz, 1P, *P*C(CH$_3$)$_3$)$_2$). **LIFDI-MS:** 790.2 (100)

[(µ-P$_2$){(PNP)Pd}$_2$] (2^{Pd,P})

[(PNP)Pd-PCO] (**1^{Pd,P}**) (17.2 mg, 33.0 µmol) is dissolved in benzene (0.5 mL) and photolyzed with an LED (456 nm) for 3 h at room temperature. The solvent is removed and the residue is washed with cold pentane (2 x 0.1 ml). After lyophilization with benzene, a dark purple solid of analytical purity is obtained (14.1 mg, 14.3 µmol, 87 %).
^{1}H NMR (C_6D_6, 400 MHz, [ppm]): δ = 6.85 (ABXX'A'B', N = |$^3J_{HP}$ + $^4J_{HP}$| = 35.9 Hz $^3J_{HH}$ = 5.4 Hz, 4H, NC*H*), 4.01 (ABXX'A'B', N = |$^2J_{HP}$+$^4J_{HP}$| = 9.9 Hz $^3J_{HH}$ = 5.5 Hz, 4H, C*H*P), 1.46 (A_{36}BXX'A'$_{36}$B', N = |$^3J_{HP}$ + $^5J_{HP}$| = 13.8 Hz, 72H, P(C(C*H*$_3$)$_3$)$_2$). **^{13}C{^{1}H} NMR** (C_6D_6, 100 MHz, [ppm]): δ = 159.6 (t, $^2J_{PC}$ = 8.4 Hz, 4C, N*C*H), 82.6 (t, $^1J_{PC}$ = 19.0 Hz, 4C, *C*HP), 36.7 (t, $^1J_{PC}$ = 8.5 Hz, 8C, P(*C*(CH$_3$)$_3$)$_2$), 30.2 (m, $^2J_{PC}$ = 3.0 Hz, 24C, P(C(CH$_3$)$_3$)$_2$). **^{31}P{^{1}H} NMR** (C_6D_6, 162 MHz, [ppm]): δ = 784.1 (br, 2P, Pd-*P*$_2$-Pd), 59.9 (br, 4P, *P*C(CH$_3$)$_3$)$_2$).

Raman: 585 cm^{-1} (P=P). **UV-Vis (THF)**: λ_{max} (ε, M^{-1} cm^{-1}) = 241 (96441), 320 (78037), 549 (23822) nm. **LIFDI-MS**: m/z = 988.1 (100). **Elem. Anal.:** Calcd. $C_{40}H_{80}N_2P_6Pd_2$ (987.8) C, 48.64; H, 8.16; N, 2.84. Found: C, 48.34; H, 7.70; N, 2.48.

[(μ-As$_2$){(PNP)Pd}$_2$] (2^{Pd,As})

[(PNP)PdAsCO] (**1^{Pd,As}**) (15 mg, 33.0 μmol, 1.00 eq.) is dissolved in THF photolyzed with an LED (525 nm, 25% intensity) for 90 min at 0 °C. A color change to deep red is observed with concomitant precipitation of the product. The solvent is removed in vacuo and the product washed with pentane (4 x 1 mL) and cold dichloromethane (2 x 1 mL), yielding analytically pure product as a dark red solid (11.7 mg, 10.9 μmol, 82.0 %).
^{1}H NMR (THF-d_8, 400 MHz, [ppm]): δ = 7.31 (ABXX'A'B', N = |$^3J_{HP}$ + $^4J_{HP}$| = 37.2 Hz, $^3J_{HH}$ = 5.4 Hz, 4H, NC*H*), 3.96 (ABXX'A'B', N = |$^2J_{HP}$+$^4J_{HP}$| = 10.1 Hz $^3J_{HH}$ = 5.4 Hz, 4H, C*H*P), 1.32 (A_{36}BXX'A'$_{36}$B', N = |$^3J_{HP}$ + $^5J_{HP}$| = 13.8 Hz, 72H, P(C(C*H*$_3$)$_3$)$_2$). **^{13}C{^{1}H} NMR** (THF-d_8, 125 MHz, [ppm]): δ = 159.5 (t, $^2J_{PC}$ = 8.2 Hz, 4C, N*C*H), 82.7 (t, $^1J_{PC}$ = 19.2 Hz, 4C *C*HP), 37.3 (t, $^1J_{PC}$ = 8.7 Hz, 8C, P(*C*(CH$_3$)$_3$)$_2$), 30.5 (t, $^2J_{PC}$ = 3.1 Hz, 24C, P(C(*C*H$_3$)$_3$)$_2$). **^{31}P{^{1}H} NMR** (THF-d_8, 162 MHz, [ppm]): δ = 66.0 (s, 4P, *P*C(CH$_3$)$_3$)$_2$). **Raman:** 308 cm^{-1} (As=As). **UV-Vis (THF):** λ_{max} (ε, M^{-1} cm^{-1}) = 246 (45662), 321 (29669), 411 (7687), 528 (5677) nm. **LIFDI-MS**: m/z = 1076.0 (100). **Elem. Anal.:** Calcd. $C_{40}H_{80}As_2N_2P_4Pd_2$ (1075.7) C, 44.66 H, 7.50; N, 2.60. Found: C, 44.63; H, 7.37; N, 2.51.

[(μ-As$_2$){(PNP)Pt}$_2$] (2^{Pt,As})

[(PNP)Pt-AsCO] (**1^{Pt,As}**) (10.0 mg, 15.3 μmol, 1.00 eq.) is dissolved in toluene (2 mL) and irradiated with an LED (525 nm, 25% Intensity) at –30 °C for 30min. A color change to yellow and precipitation of the product are observed. The solvent is removed *in vacuo* and the residue is washed with pentane (2 x 1 mL). Extraction with benzene (4 mL) and subsequent lyophilization results in a yellow, analytically pure solid (8.3 mg, 7.3 μmol, 87 %).
^{1}H NMR (C_6D_6, 400 MHz, [ppm]): δ = 7.26 (ABXX'A'B', N = |$^3J_{HP}$ + $^4J_{HP}$| = 35.2 Hz $^3J_{HH}$ = 5.4 Hz, 4H, NC*H*), 4.17 (ABXX'A'B', N = |$^2J_{HP}$+$^4J_{HP}$| = 8.8 Hz, $^3J_{HH}$ = 5.3 Hz, 4H, C*H*P), 1.45 (A_{36}BXX'A'$_{36}$B', N = |$^3J_{HP}$ + $^5J_{HP}$| = 14.1 Hz, 72H, P(C(C*H*$_3$)$_3$)$_2$). **^{13}C{^{1}H} NMR** (C_6D_6, 125 MHz, [ppm]): δ = 160.2 (t, $^2J_{PC}$ = 7.1 Hz, 4C, N*C*H), 84.4 (t, $^1J_{PC}$ = 23.3 Hz, 4C, *C*HP), 37.2 (t, $^1J_{PC}$ = 13.2 Hz, 8C, P(*C*(CH$_3$)$_3$)$_2$), 30.4 (t, $^2J_{PC}$ = 2.5 Hz, $^3J_{PtC}$ = 13.6 Hz, 24C, P(C(*C*H$_3$)$_3$)$_2$). **^{31}P{^{1}H} NMR** (C_6D_6, 162 MHz, [ppm]): δ = 60.3 (s, $^1J_{PtP}$ = 2921.7 Hz, 4P, *P*C(CH$_3$)$_3$)$_2$). **^{195}Pt{^{1}H} NMR** (C_6D_6, 107 MHz, [ppm]): δ = – 3491.1 (t, $^1J_{PPt}$ = 2908.4 Hz). **IR (ATR, cm^{-1}):** 1546 (C=C). **Raman:** 302 cm^{-1} (As=As). **UV-Vis (THF):** λ_{max} (ε, M^{-1} cm^{-1}) = 318 (28602), 416 (5617), 467 (4168), 696 (29) nm. **LIFDI-MS:** m/z = 1252.1 (100) **Elem. Anal.:** Calcd. $C_{40}H_{80}As_2N_2P_4Pt_2$ (1253.0) C, 38.34; H, 6.44; N, 2.24. Found: C, 38.32; H, 6.43; N, 2.15

[(μ-Sb$_2$){(PNP)Pt}$_2$] (2^{Pt,Sb})

[(PNP)Pt-SbH$_2$] (**1^{Pt,Sb}**) (10.0 mg, 14.8 μmol, 1.00 eq.) is dissolved in 2-methytetrahydrofuran (2 mL) in a J-Young flask and photolyzed with an LED (370 nm) at -196 °C for 6h, resulting in color change of the frozen solution to deep purple. After thawing, immediate color change to orange and precipitation of the product is observed. The solvent is removed *in vacuo*. Washing the residue with pentane (2 x 1 mL) and cold dichloromethane (2 x 1 mL) results in a bright orange solid (8.4 mg, 6.2 μmol, 84 %). Poor solubility prevented full NMR spectroscopic characterization. See chapter Spectroscopic Characterization for further details.
^{31}P{^{1}H} NMR (THF-d_8, 162 MHz, [ppm]): δ = 63.9 (s, 4P, *P*(C(CH$_3$)$_3$)$_2$, ^{195}Pt satellites could not be found due to the low solubility). **IR (ATR):** 1538 (C=C) cm^{-1}. **UV-Vis (THF):** λ_{max} (ε, M^{-1} cm^{-1}) = 262 (44414), 312 (35698), 384 (13056), 497 (8363) nm. **LIFDI-MS**: m/z = 1346.0 (100). **Elem. Anal.:** Calcd. $C_{40}H_{80}P_4N_2Sb_2Pt_2$ (1346.7) C, 35.68; H, 5.99; N, 2.08. Found C, 35.56, H, 6.06, N, 2.01.

[(μ-Bi$_2$){(PNP)Pt}$_2$] (2^{Pt,Bi})

A toluene solution of [(PNP)Pt-BiMe$_2$] (**1^{Pt,Bi}**) (5.10 mg, 6.45 μmol, 1.00 eq.) is photolyzed with an LED (370 nm) at -40 °C overnight. A color change to green is accompanied by product precipitation. The solvent is removed *in vacuo*. Washing with hexane (2 x 1 mL) and diethylether (2 x 1 mL) results in a green solid (2.40 mg, 1.58 μmol, 25 %). Single crystals suitable for X-ray diffraction are obtained from a saturated toluene solution. Poor solubility prevented NMR spectroscopic characterization.
UV-Vis (THF): λ_{max} (ε, M^{-1} cm^{-1}) = 322 (184155), 391 (21203), 468 (11862), 595 (3962) nm. **LIFDI-MS**: m/z = 1520.1 (100). **Elem. Anal.:** Calcd. $C_{40}H_{80}P_4N_2Bi_2Pt_2$ (1521.1) C, 31.58; H, 5.30; N, 1.84. Found C, 31.94, H, 5.44, N, 1.88.

[(μ-As$_2$){(PNP)Pt}$_2$]BArF$_{24}$ ([2^{Pt,As}]$^+$)

[(μ-As$_2$){(PNP)Pt}$_2$] (**2^{Pt,As}**) (6.40 mg, 5.11 μmol, 1.00 eq.) is dissolved in dichloromethane (2 mL) at –38 °C. [Fe(C_5Me_5)$_2$]BArF$_{24}$ (5.70 mg, 4.79 μmol, 0.94 eq.) is added and the mixture is stirred and while warming to room temperature. The color of the solution changes from yellow to deep red. After removal of the solvent, the remaining solid is washed with pentane (3 x 1.5 mL) and extracted with dichloromethane (2 x 1 mL). Layering with pentane (10 mL) affords the product over several days at -38 °C. Decantation of the solvent and washing with pentane gives the product as a dark red solid (9.80 mg, 4.63 μmol, 90 %) after drying *in vacuo*.
^{1}H NMR (THF-d_8, 400 MHz, [ppm]): δ = 59.3 (br, 2H), 7.81 (br, 8H, BArF$_{24}$), 7.58 (br, 4H, BArF$_{24}$), 7.11 (br, 4H), 4.76 (br, 72H, P(C(C*H*$_3$)$_3$)$_2$), –36.9 (br, 2H). **^{11}B{^{1}H} NMR** (THF-d_8, 96 MHz, [ppm]): δ = –6.48 (br, *B*ArF$_{24}$). **^{19}F{^{1}H} NMR** (THF-d_8, 282 MHz, [ppm]):

δ = –63.3 (br, BArF_{24}). **Raman:** 340 cm^{-1} (As=As). **UV-Vis (THF):** λ_{max} (ε, M^{-1} cm^{-1}) = 310 (47542), 540 (5731), 952 (861) nm. **LIFDI-MS**: m/z = 1251.1 (100). μ_{eff}(THF): 1.5(2) μ_B. **Elem. Anal.:** Calcd. $C_{72}H_{92}As_2BF_{24}N_2P_4Pt_2$ (3471.4) C, 40.86; H, 4.38; N, 1.32. Found: C, 40.51; H, 4.19; N, 1.29.

Spectroscopic Characterization of [(PNP)Pd-PCO] ($1^{Pd,P}$)

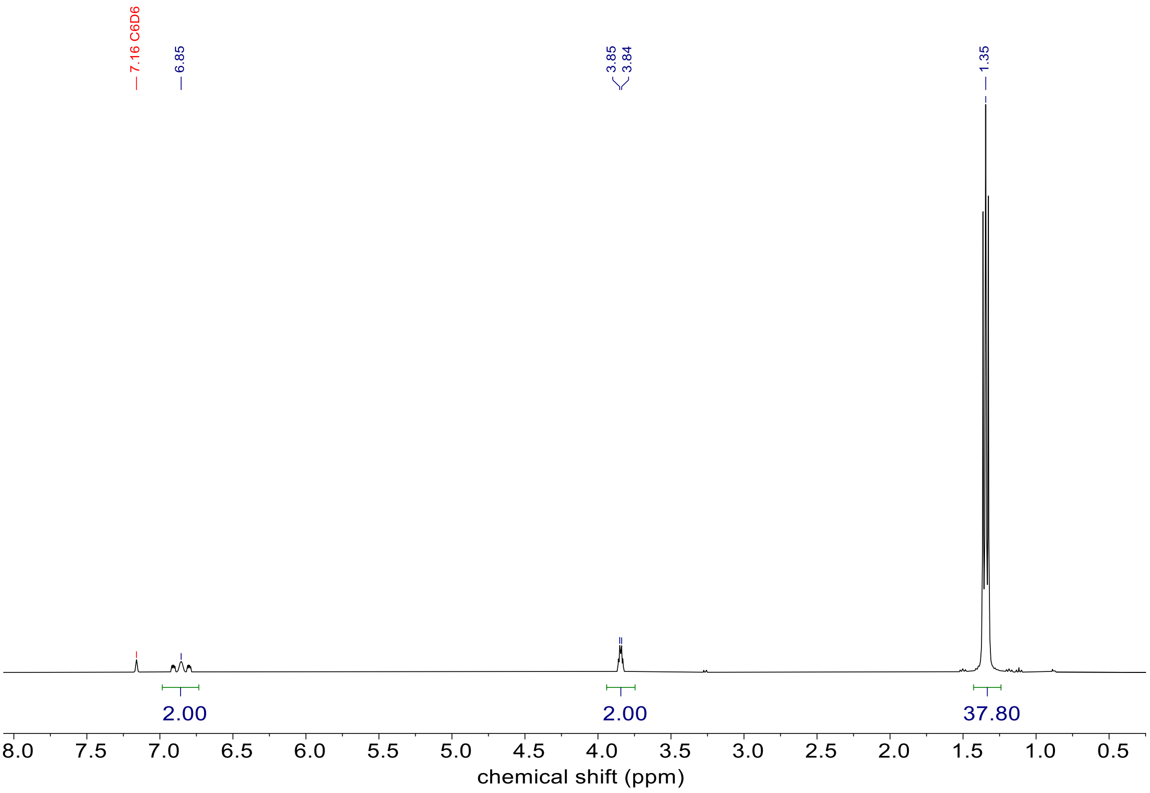

Figure S1: ^{1}H-NMR spectrum of [Pd(PCO)(PNP)] ($\mathbf{1}^{\mathbf{Pd,P}}$) in C_6D_6 at room temperature.

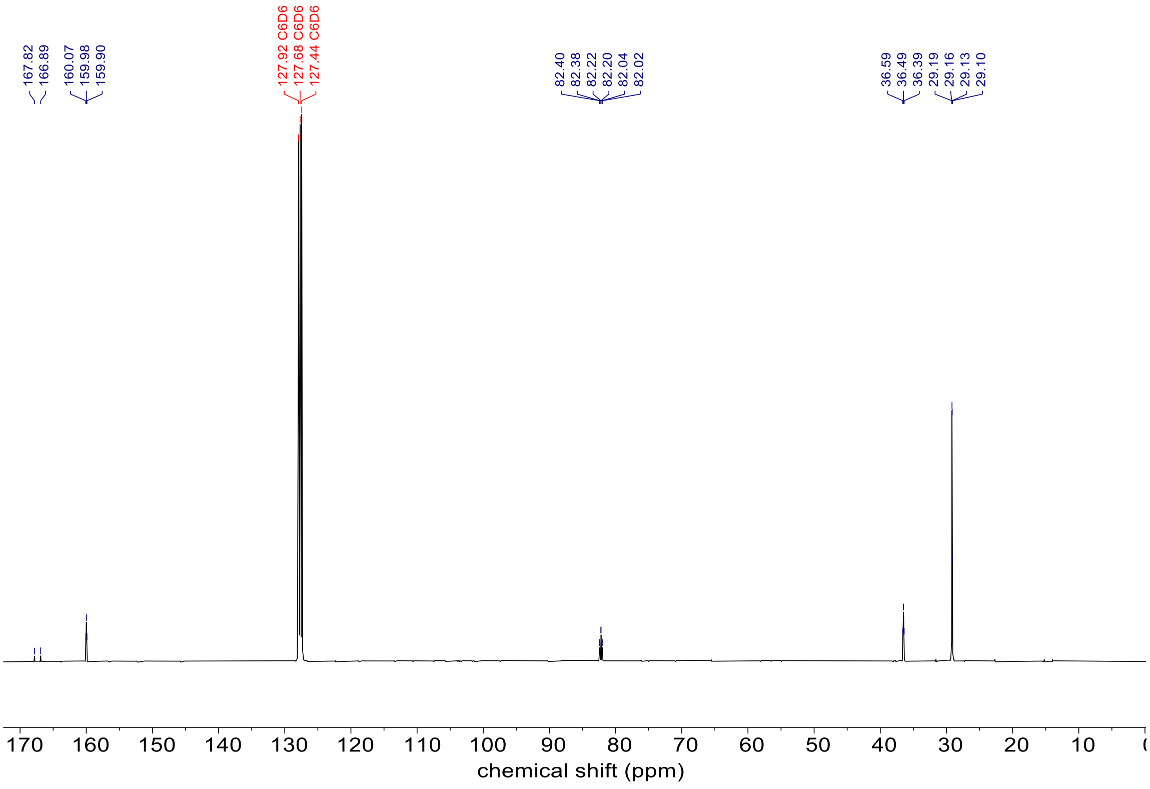

Figure S2: ^{13}C{^{1}H}-NMR spectrum of [Pd*(*PCO*)*(PNP)] ($\mathbf{1}^{\mathbf{Pd,P}}$) in C_6D_6 at room temperature.

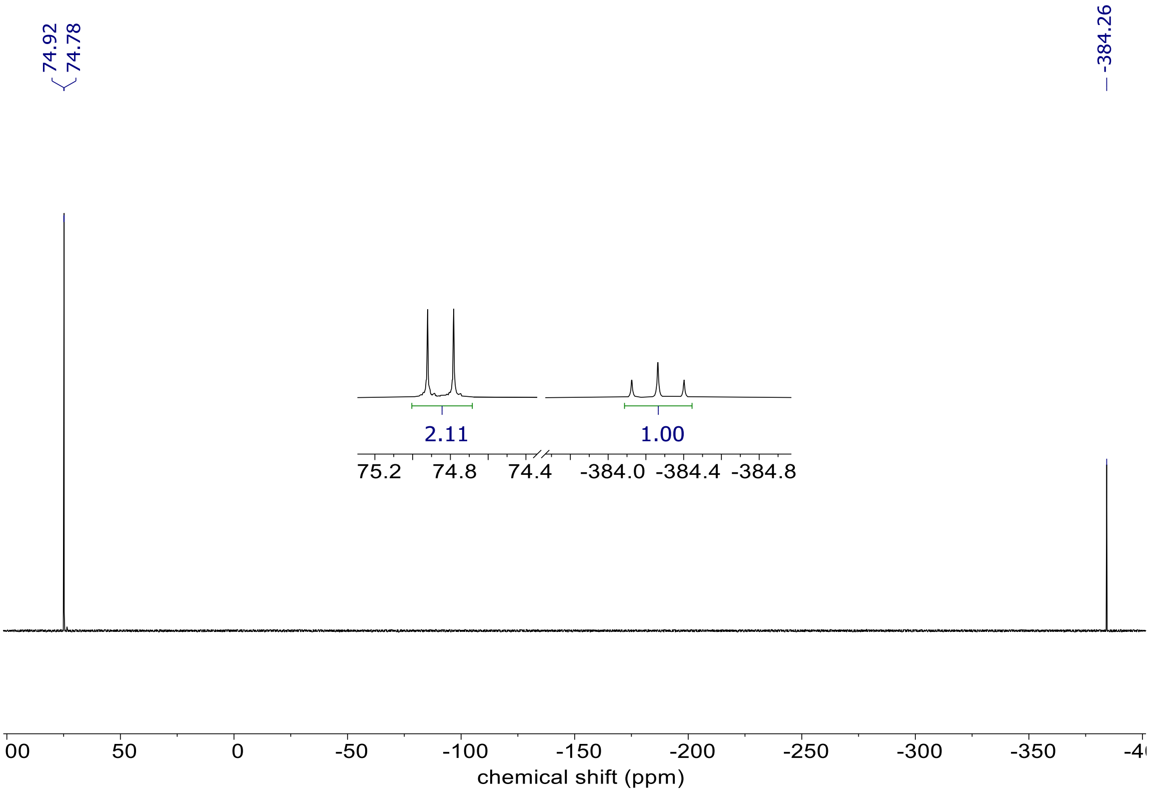

Figure S3: $^{31}P\{^{1}H\}$-NMR spectrum of [Pd(PCO)(PNP)] ($\mathbf{1}^{\mathbf{Pd,P}}$) in C_6D_6 at room temperature.

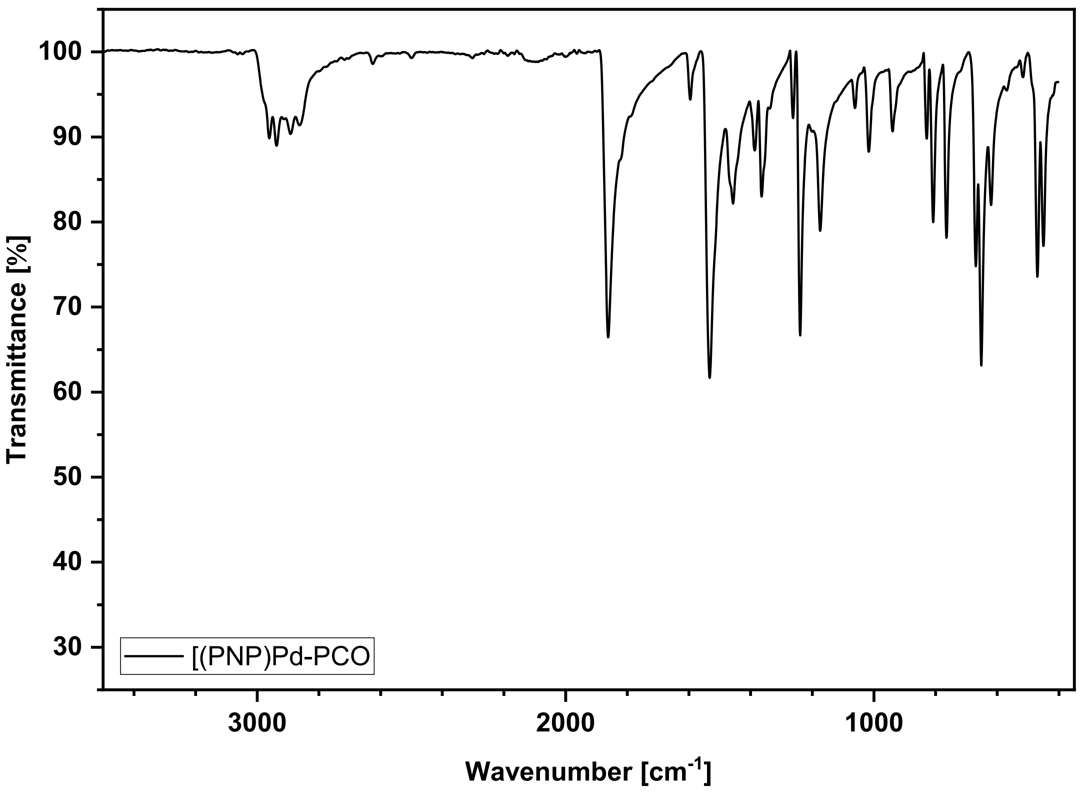

Figure S4: AT-IR spectrum of [Pd(PCO)(PNP)] ($\mathbf{1}^{\mathbf{Pd,P}}$).

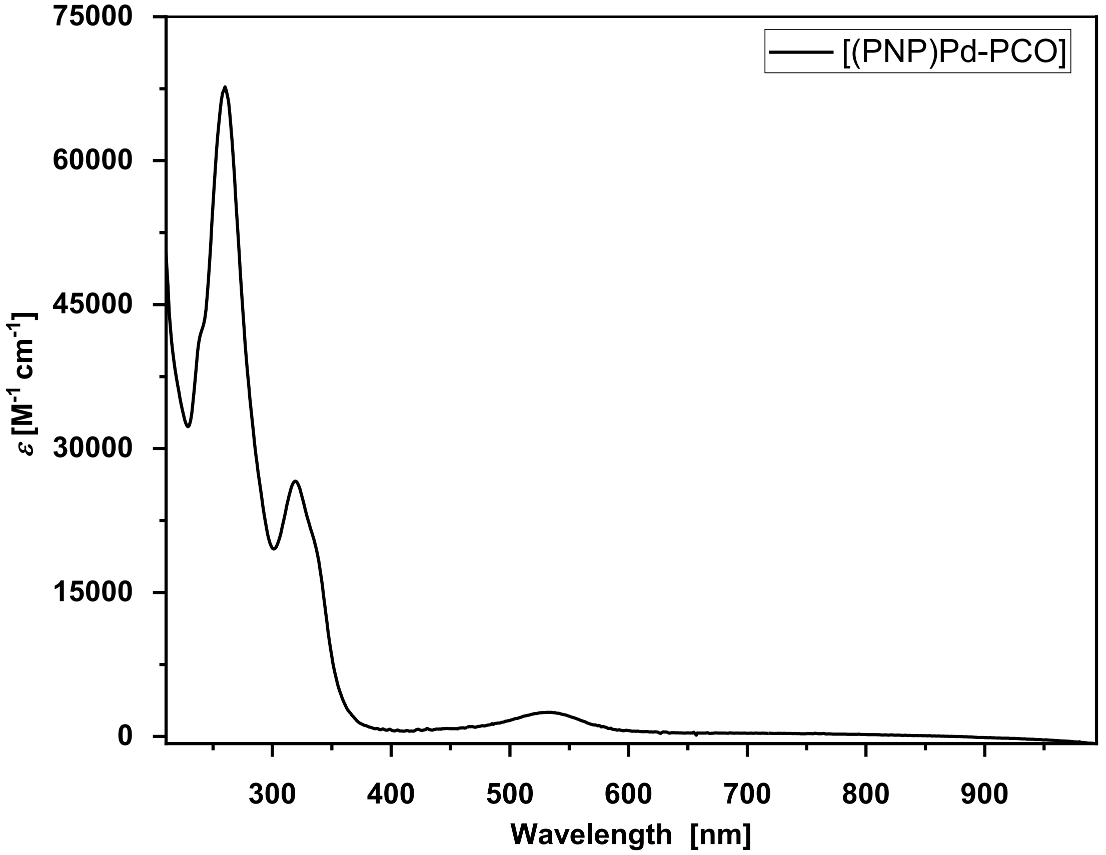

Figure S5: UV-Vis spectrum of [Pd(PCO)(PNP)] ($\mathbf{1}^{\mathrm{Pd,P}}$) in THF at room temperature.

Spectroscopic Characterization of [(PNP)Pd-AsCO] ($\mathbf{1}^{\mathrm{Pd,As}}$)

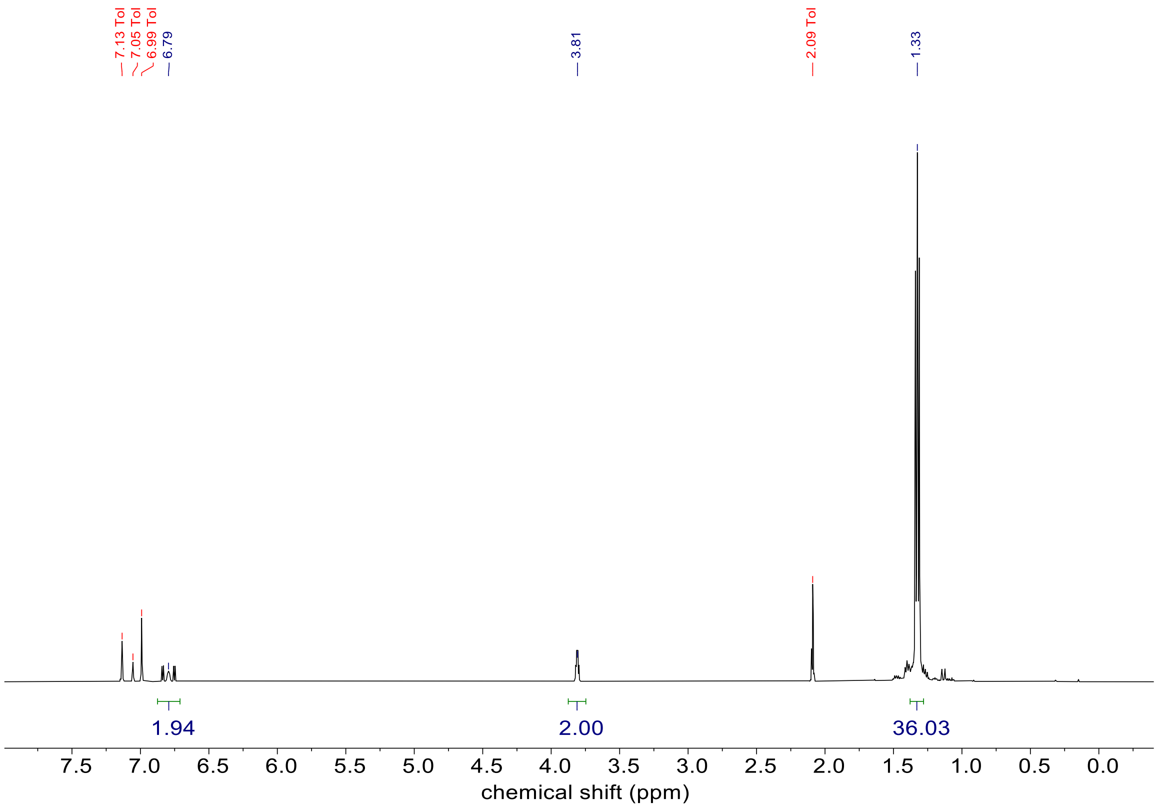

Figure S6: ^{1}H-NMR spectrum of [Pd(AsCO)(PNP)] ($\mathbf{1}^{\mathrm{Pd,As}}$) in Tol-$d_8$ at –35 °C.

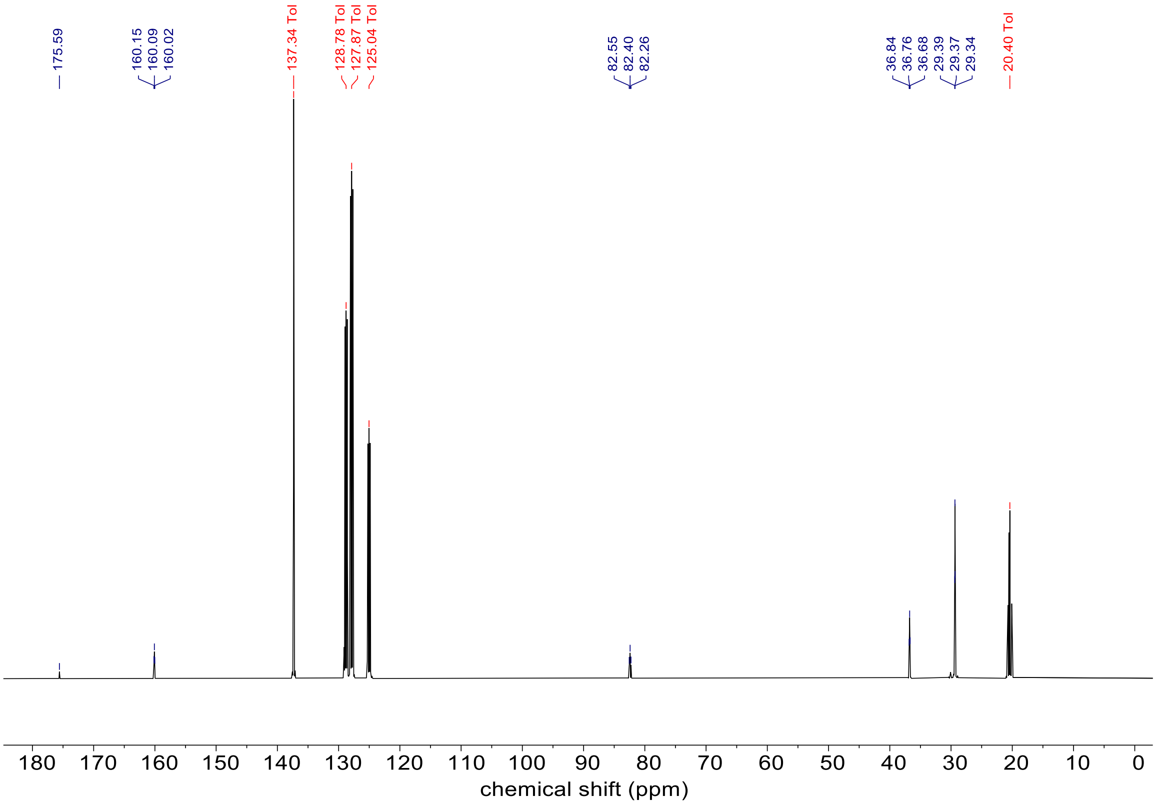

Figure S7: $^{13}C\{^1H\}$-NMR spectrum of [Pd(AsCO)(PNP)] ($\mathbf{1}^{\mathbf{Pd,As}}$) in Tol-$d_8$ at –35 °C.

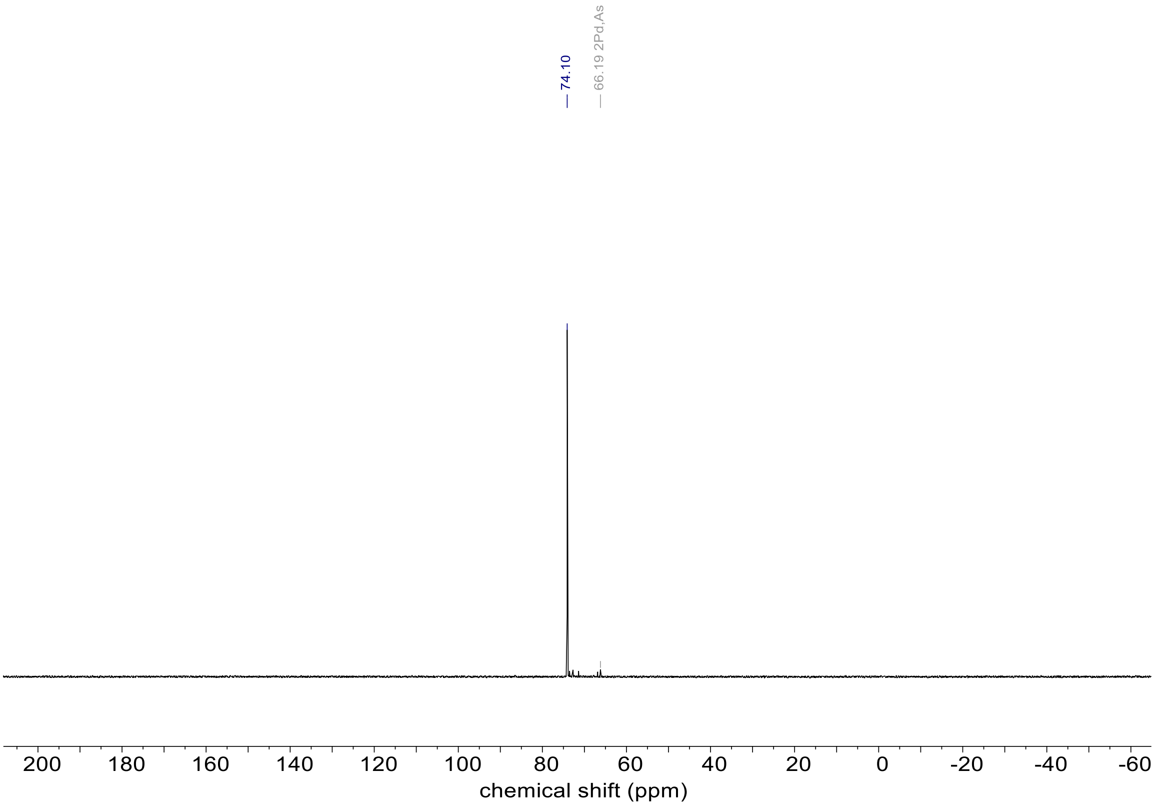

Figure S8: $^{31}P\{^1H\}$-NMR spectrum of [Pd(AsCO)(PNP)] ($\mathbf{1}^{\mathbf{Pd,As}}$) in C_6D_6 at room temperature.

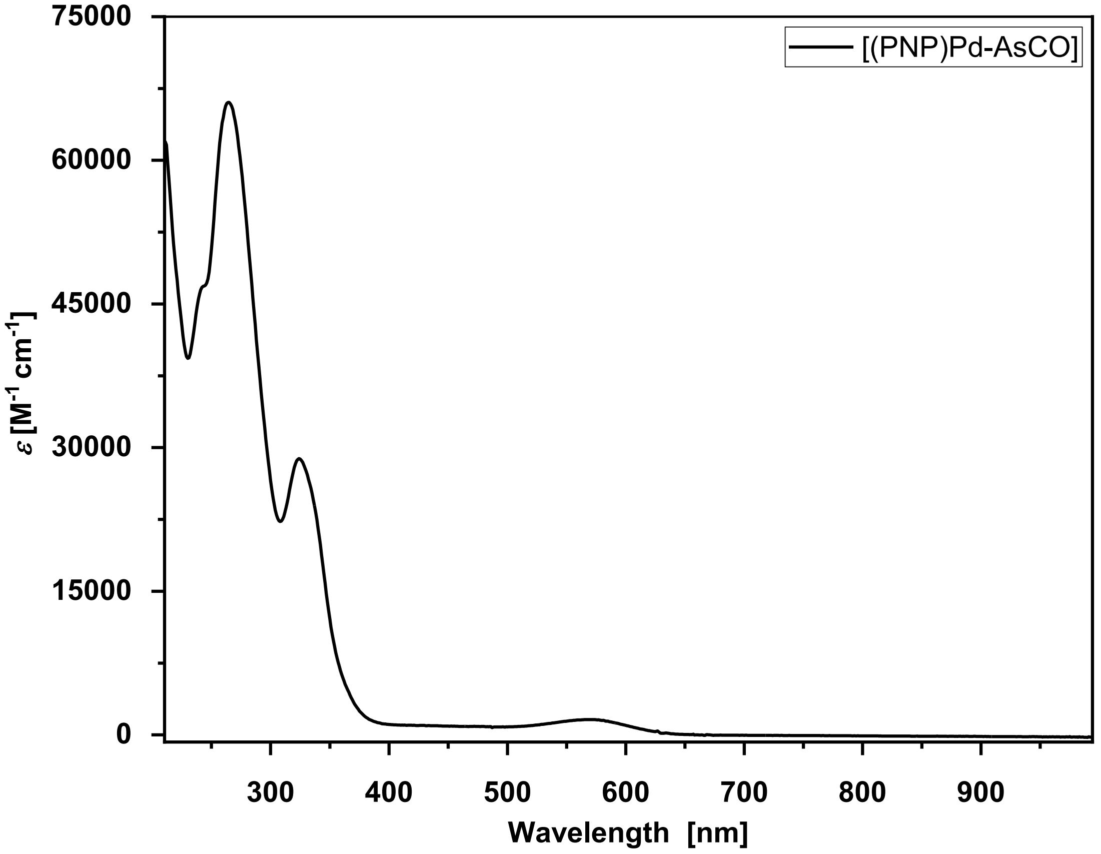

Figure S9: UV-vis spectrum of [Pd(AsCO)(PNP)] (**1**[Pd,As]) in THF at room temperature.

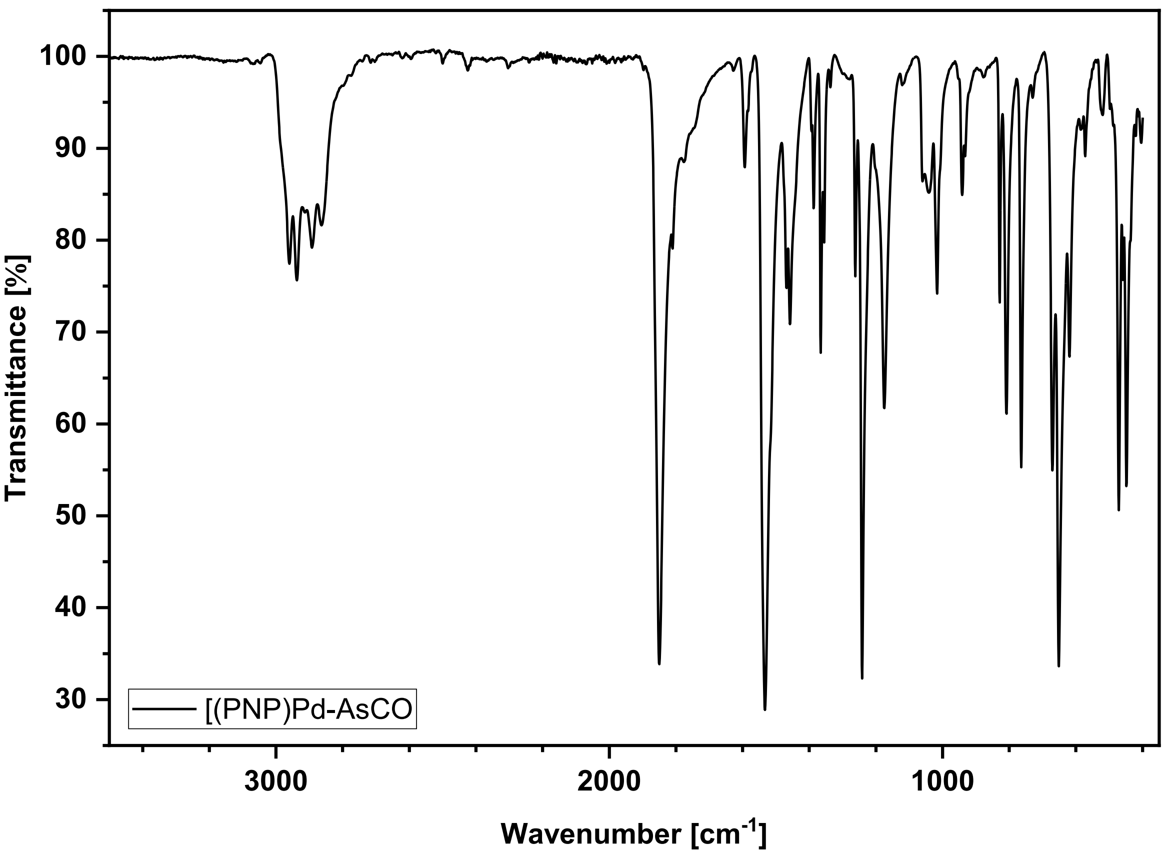

Figure S10: ATR-IR spectrum of [Pd(AsCO)(PNP)] (**1**[Pd,As]).

Spectroscopic Characterization of [(PNP)Pt-AsCO] ($1^{Pt,As}$)

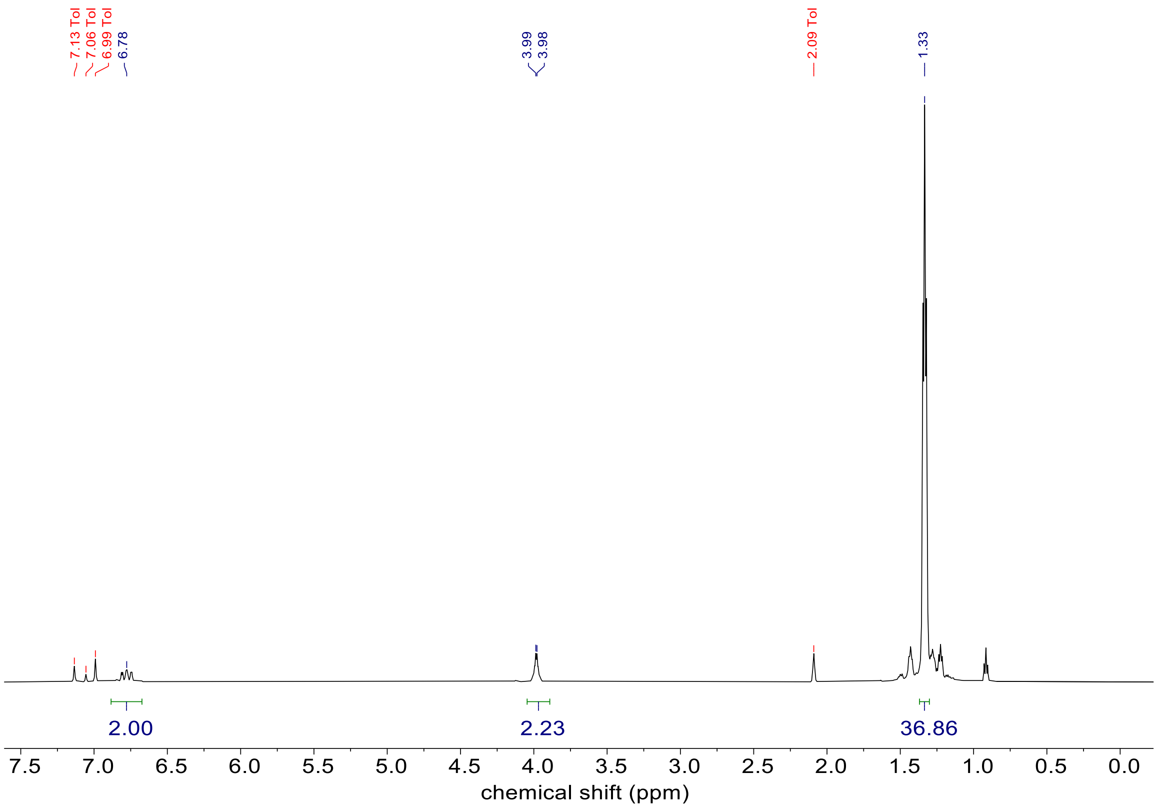

Figure S11: ^{1}H-NMR spectrum of [Pt(AsCO)(PNP)] ($\mathbf{1}^{\mathbf{Pt,As}}$) in Tol-$d_8$ at – 35 °C.

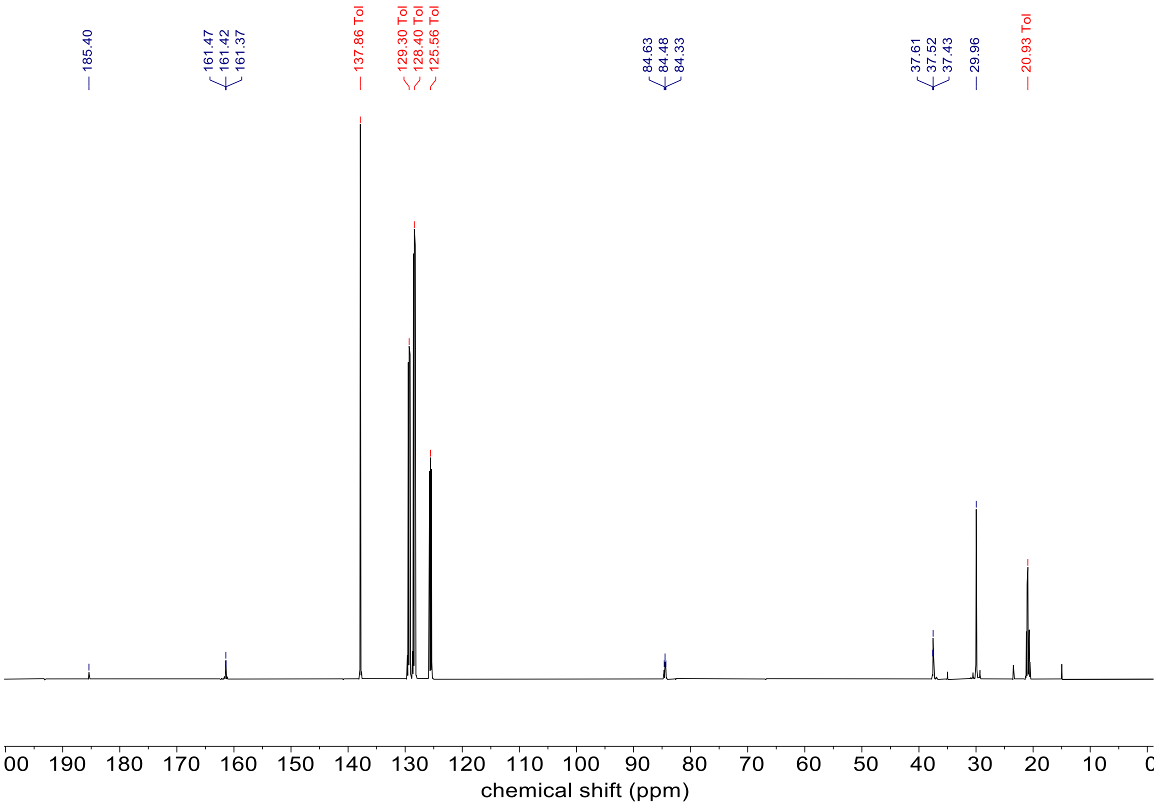

Figure S12: ^{13}C{^{1}H}-NMR spectrum of [Pt(AsCO)(PNP)] ($\mathbf{1}^{\mathbf{Pt,As}}$) in Tol-$d_8$ at – 35 °C.

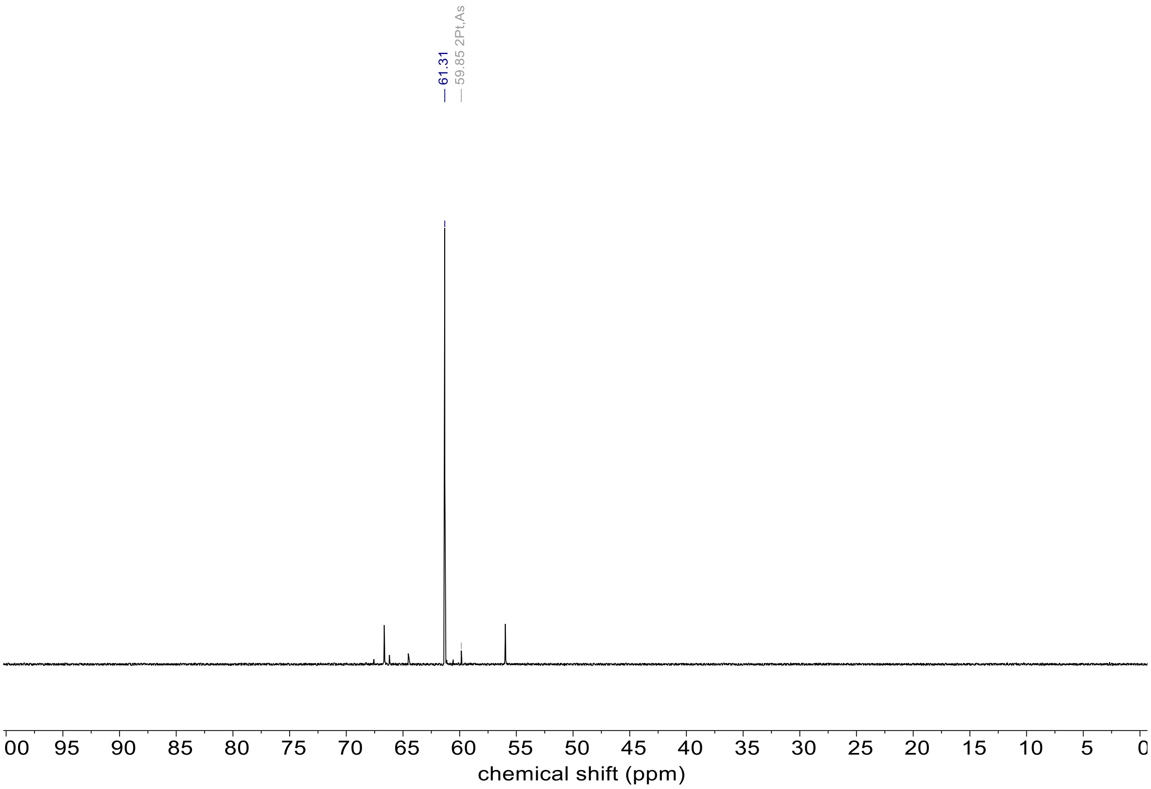

Figure S13: $^{31}P\{^{1}H\}$-NMR spectrum of [Pt(AsCO)(PNP)] (**1**$^{\mathbf{Pt,As}}$) in Tol-d_8 at – 35 °C.

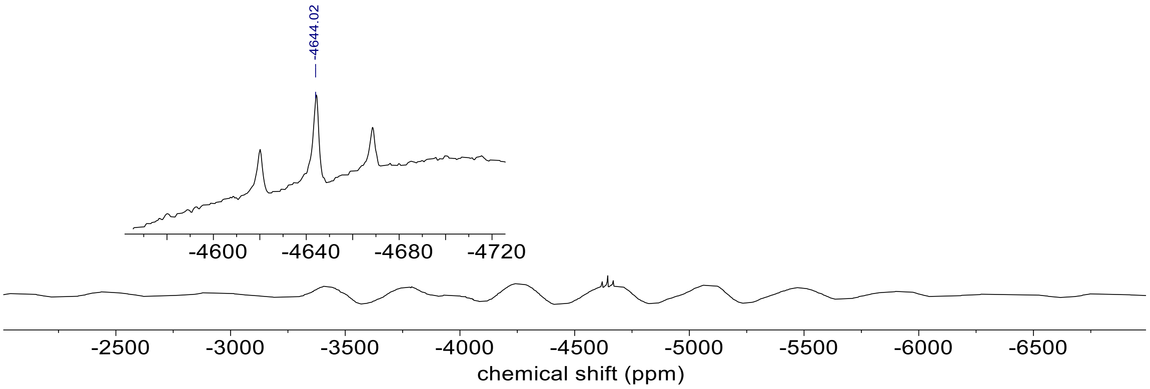

Figure S14: $^{195}Pt\{^{1}H\}$-NMR spectrum of [Pt(AsCO)(PNP)] (**1**$^{\mathbf{Pt,As}}$) in Tol-d_8 at room temperature.

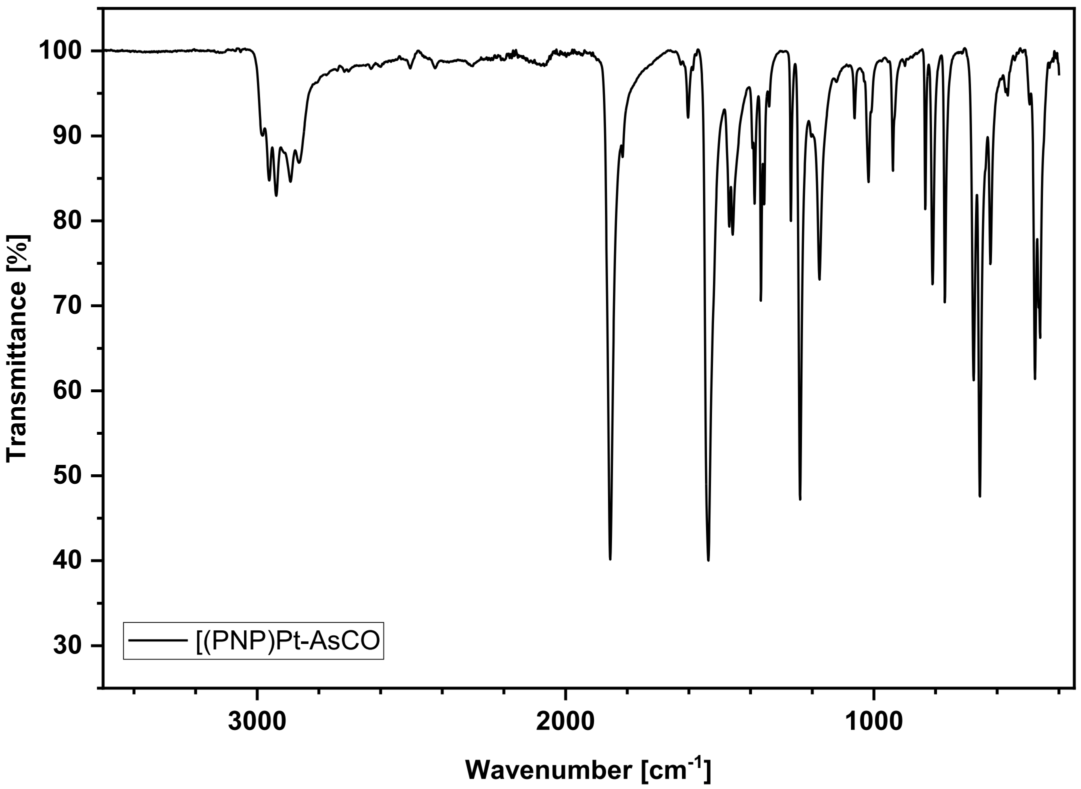

Figure S15: ATR-IR spectrum of [Pt(AsCO)(PNP)] (**1**[Pt,As]).

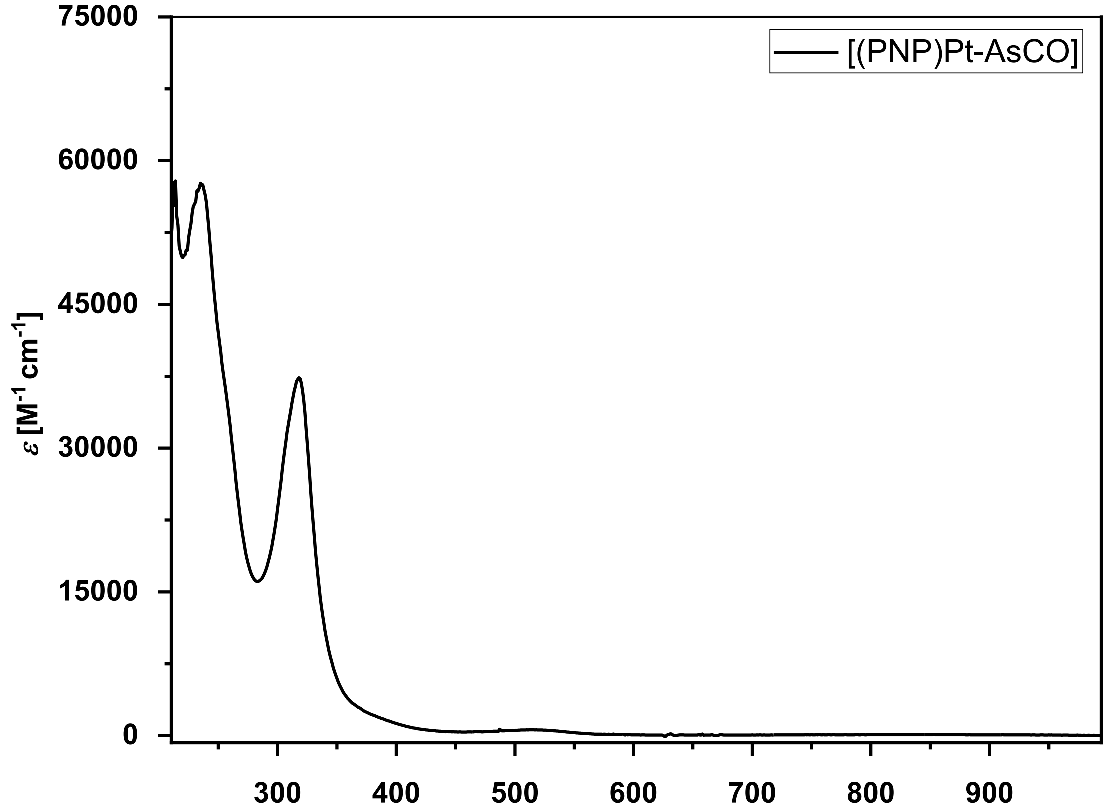

Figure S16: UV-vis spectrum of [Pt(AsCO)(PNP)] (**1**[Pt,As]) in THF at room temperature.

Spectroscopic Characterization of [(PNP)Pt-SbH$_2$] (1^{Pt,Sb})

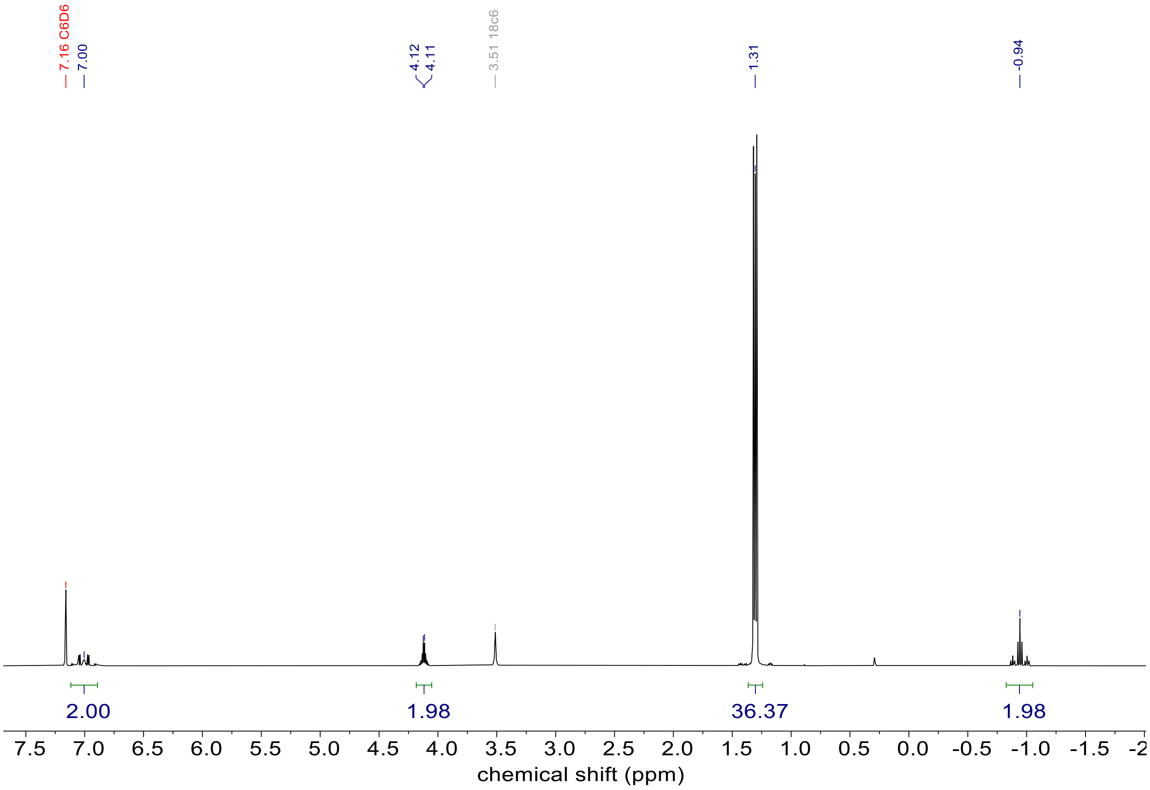

Figure S17: ^{1}H-NMR spectrum of [Pt(SbH$_2$)(PNP)] (**1^{Pt,Sb}**) in C_6D_6 at room temperature.

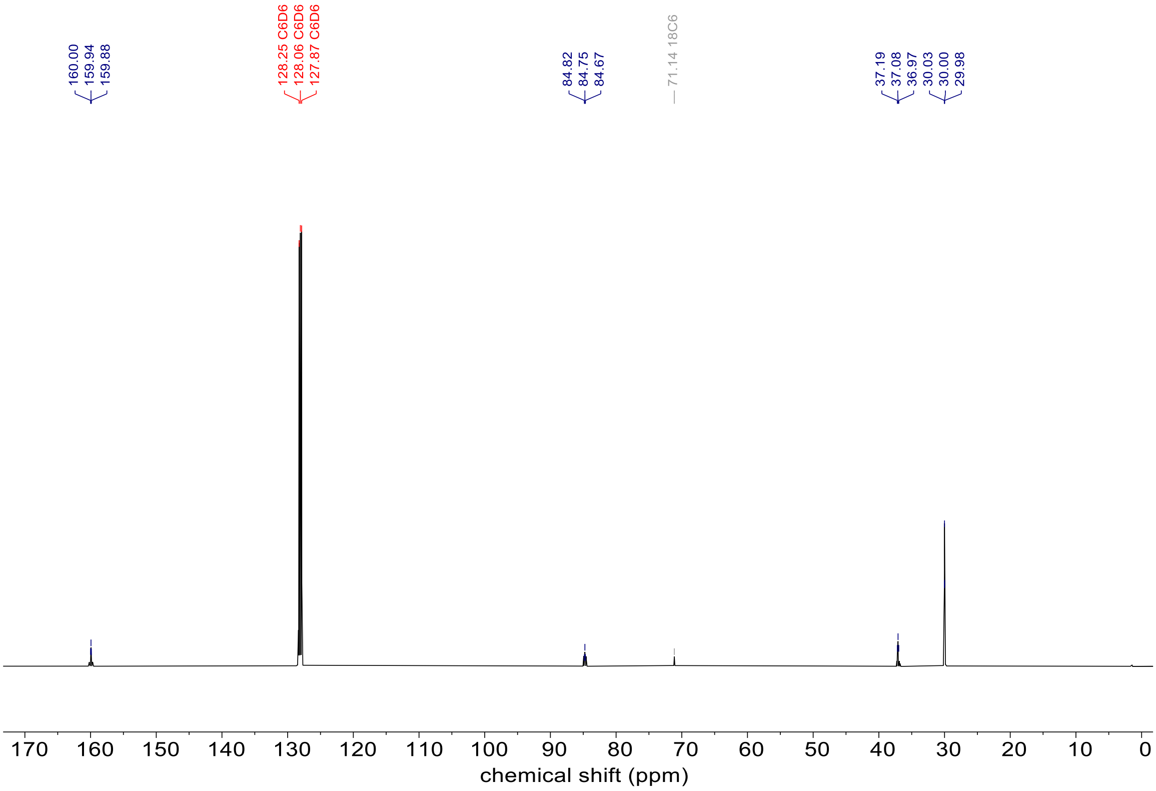

Figure S18: $^{13}C\{^{1}H\}$-NMR spectrum of [Pt(SbH$_2$)(PNP)] (**1^{Pt,Sb}**) in C_6D_6 at room temperature.

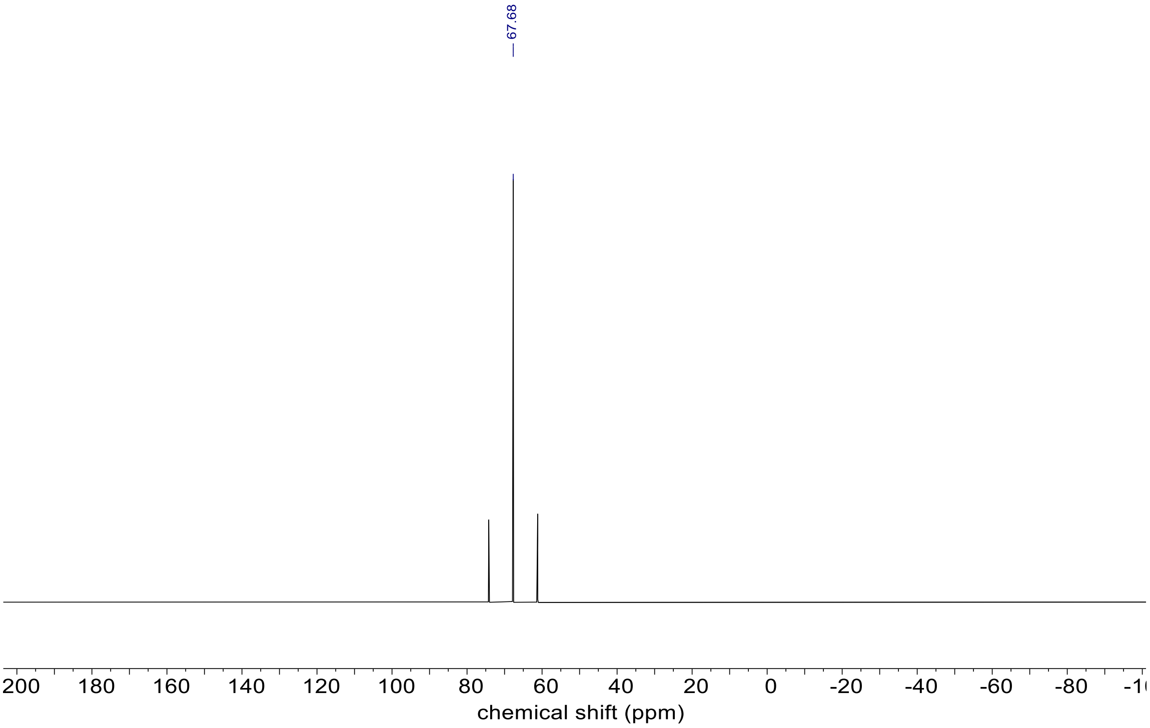

Figure S19: $^{31}P\{^{1}H\}$-NMR spectrum of [Pt(SbH$_2$)(PNP)] (**1^{Pt,Sb}**) in C_6D_6 at room temperature.

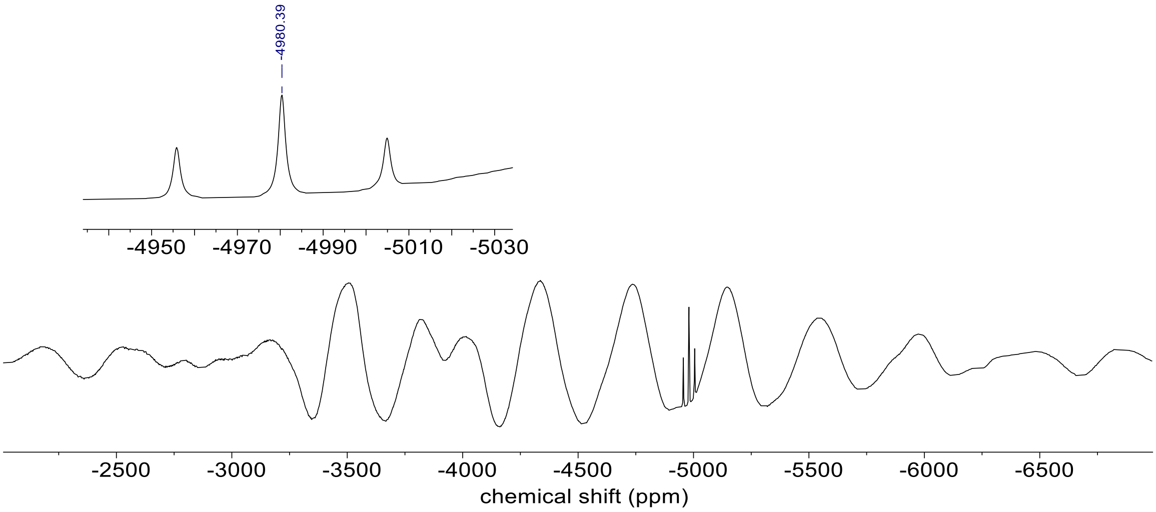

Figure S20: $^{195}Pt\{^{1}H\}$-NMR spectrum of [Pt(SbH$_2$)(PNP)] (**1^{Pt,Sb}**) in C_6D_6 at room temperature.

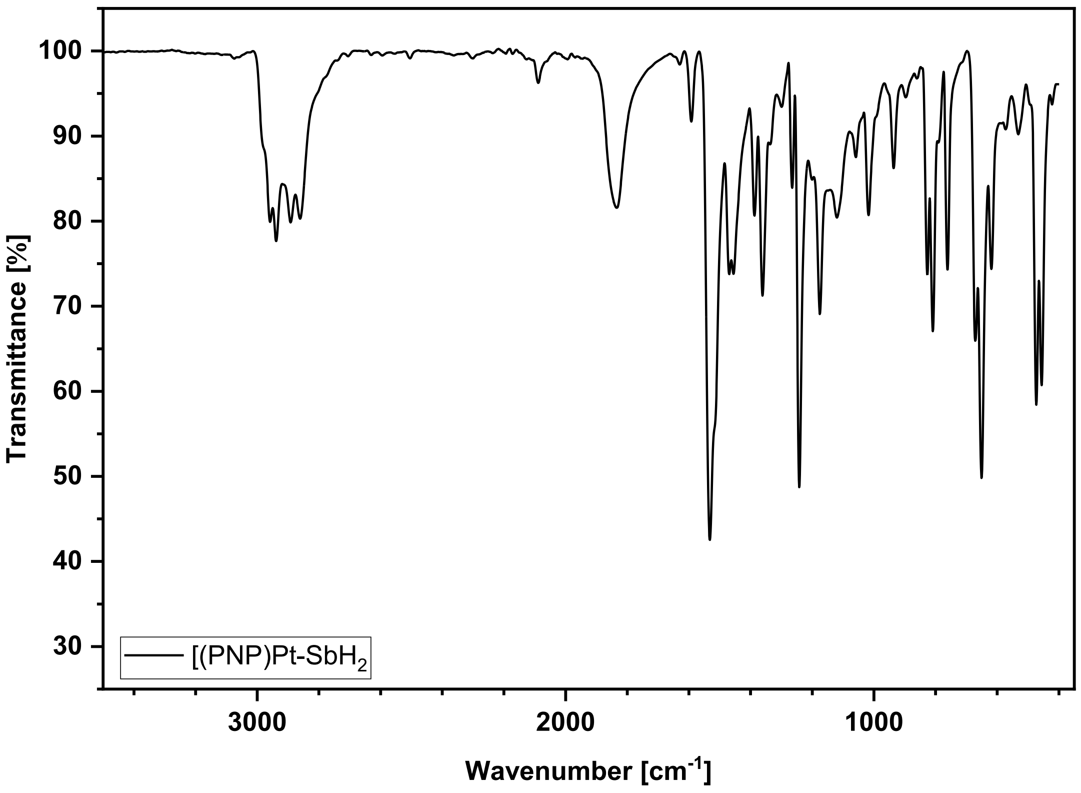

Figure S21: ATR-IR spectrum of [Pt(SbH$_2$)(PNP)] (**1**^{Pt,Sb}).

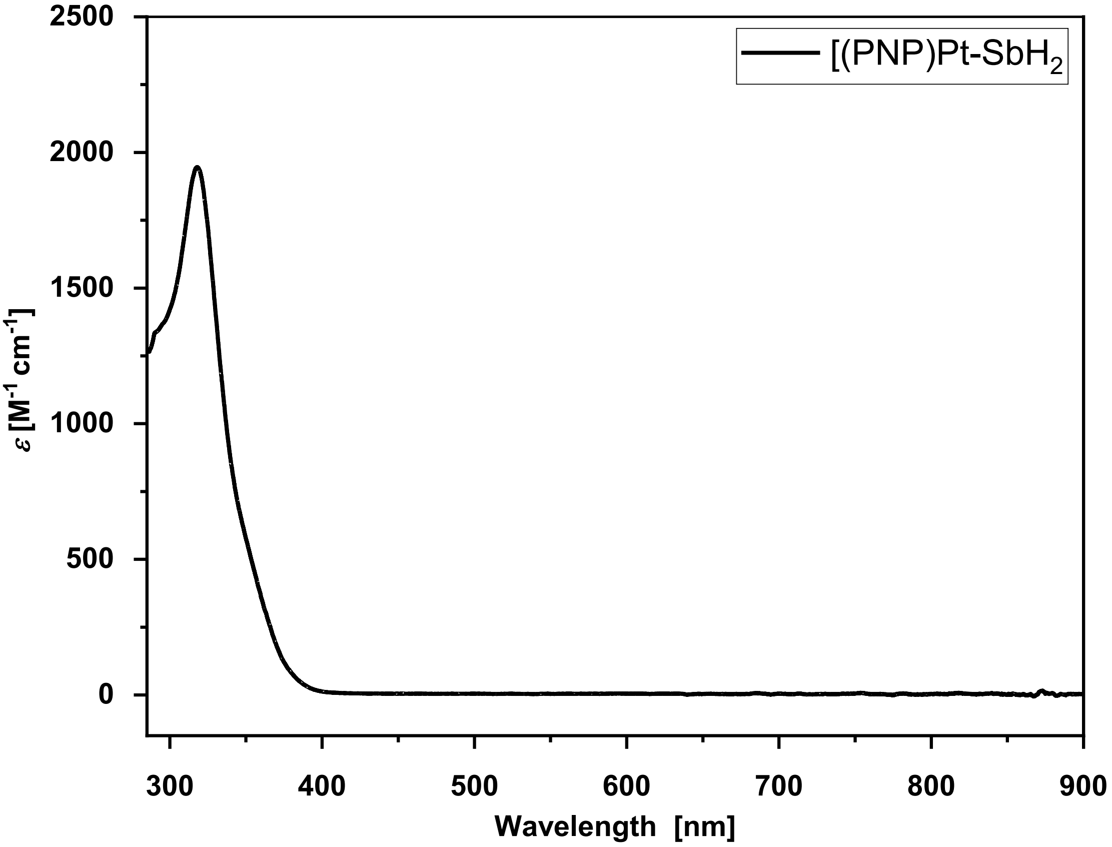

Figure S22: UV-vis spectrum of [Pt(SbH$_2$)(PNP)] (**1**^{Pt,Sb}) in benzene at room temperature.

Spectroscopic Characterization of [(PNP)Pt-BiMe$_2$] (1^{Pt,Bi})

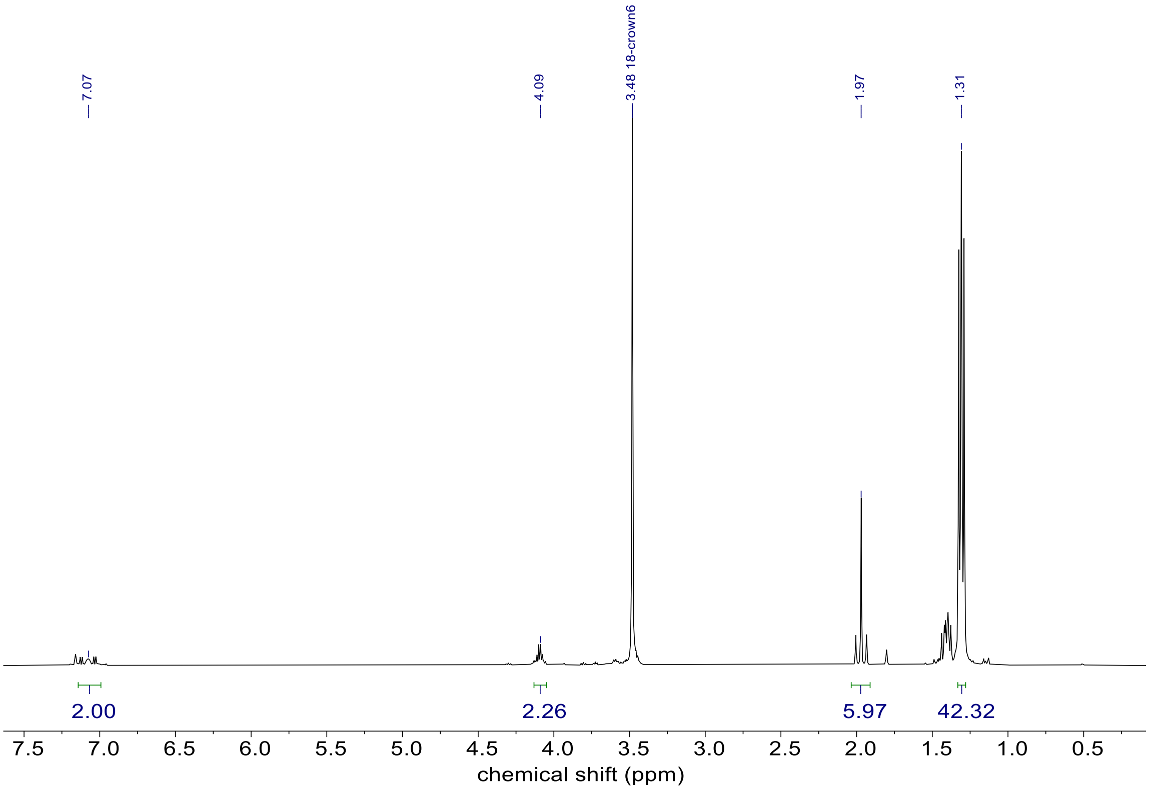

Figure S23: ^{1}H-NMR spectrum of [Pt(BiMe$_2$)(PNP)] (**1^{Pt,Bi}**) in C_6D_6 at room temperature.

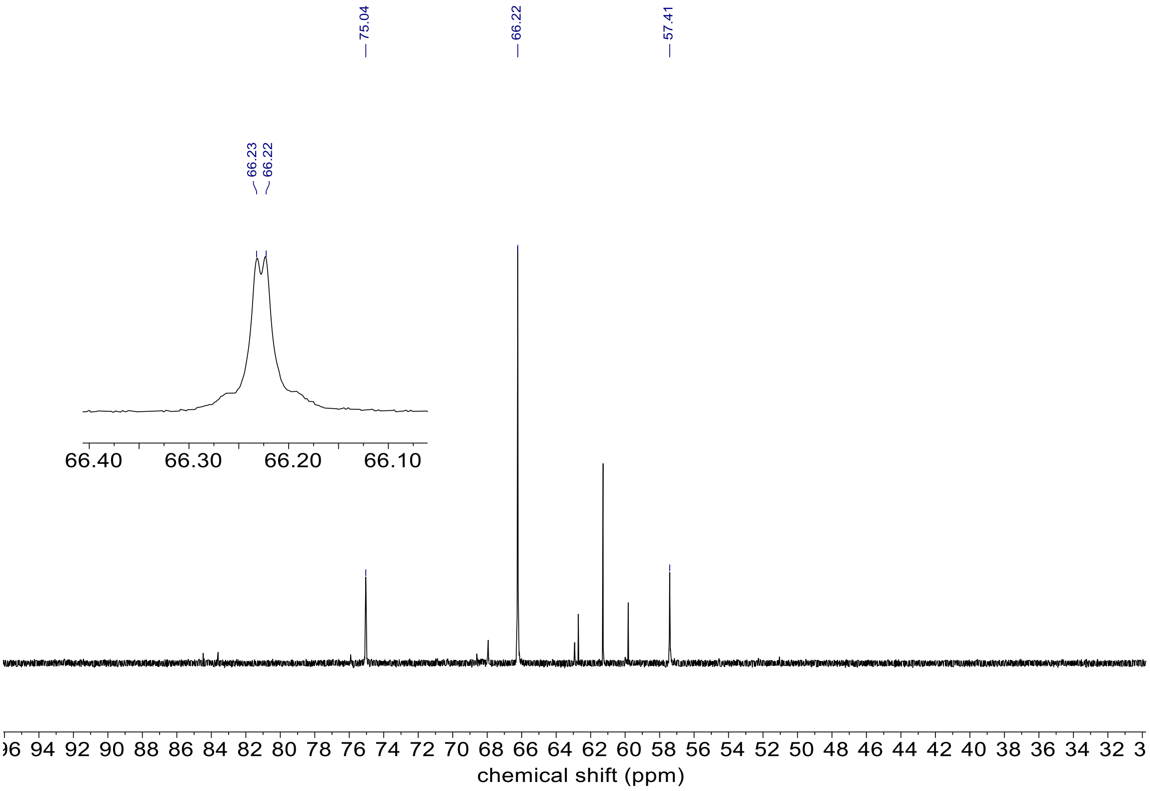

Figure S24: ^{31}P{^{1}H}-NMR spectrum of [Pt(BiMe$_2$)(PNP)] (**1^{Pt,Bi}**) in C_6D_6 at room temperature.

Spectroscopic Characterization of [(μ-P_2){(PNP)Pd}$_2$] ($2^{Pd,P}$)

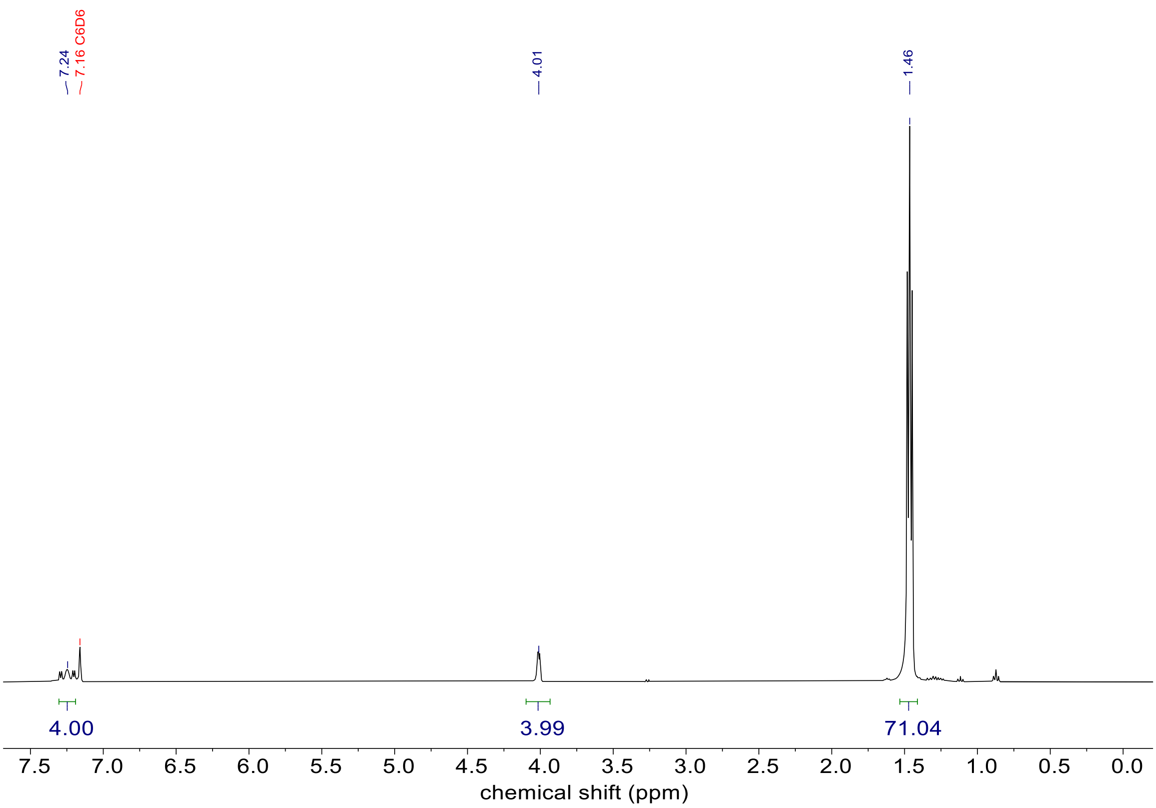

Figure S25: ^{1}H-NMR spectrum of [(μ-P_2){Pd(PNP)}$_2$] (**$2^{Pd,P}$**) in C_6D_6 at room temperature.

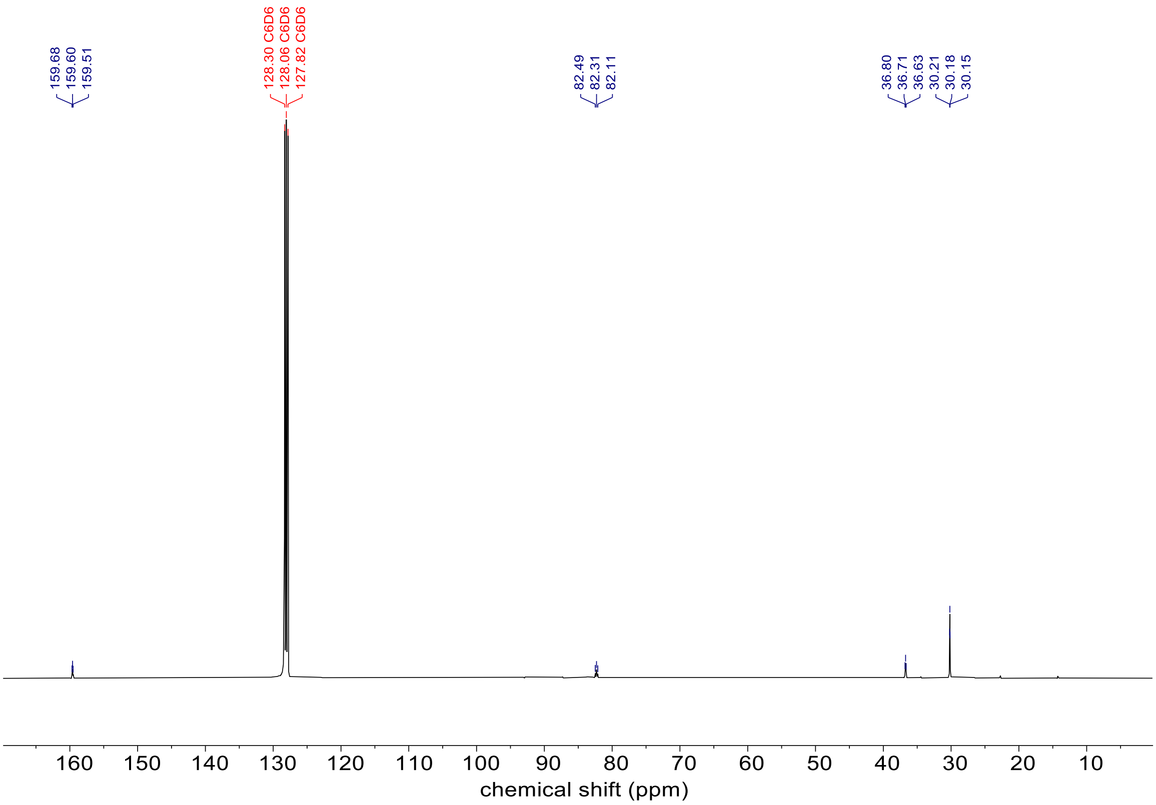

Figure S26: ^{13}C{^{1}H}-NMR spectrum of [(μ-P_2){Pd(PNP)}$_2$] (**$2^{Pd,P}$**) in C_6D_6 at room temperature.

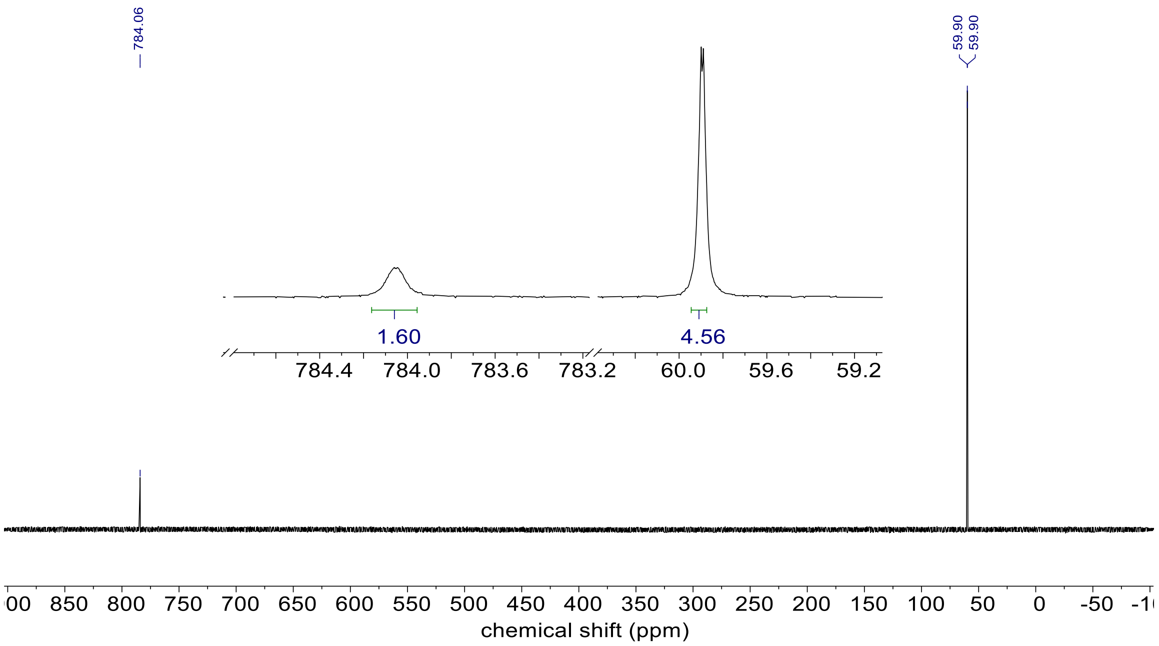

Figure S27: $^{31}P\{^{1}H\}$-NMR spectrum of [(μ-P_2){Pd(PNP)}$_2$] ($2^{Pd,P}$) in C_6D_6 at room temperature.

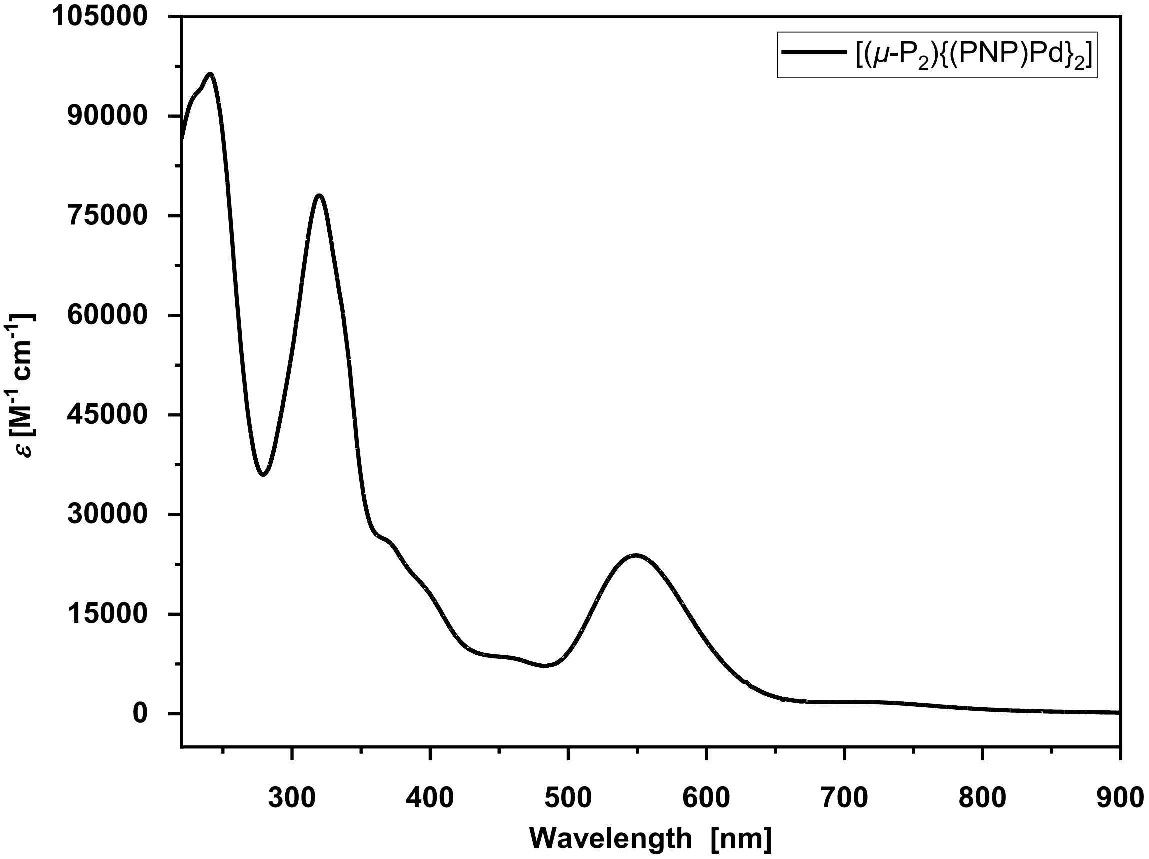

Figure S28: UV-vis spectrum of [(μ-P_2){Pd(PNP)}$_2$] ($2^{Pd,P}$) in THF at room temperature.

Spectroscopic Characterization of [(μ-As_2){(PNP)Pd}$_2$] ($2^{Pd,As}$)

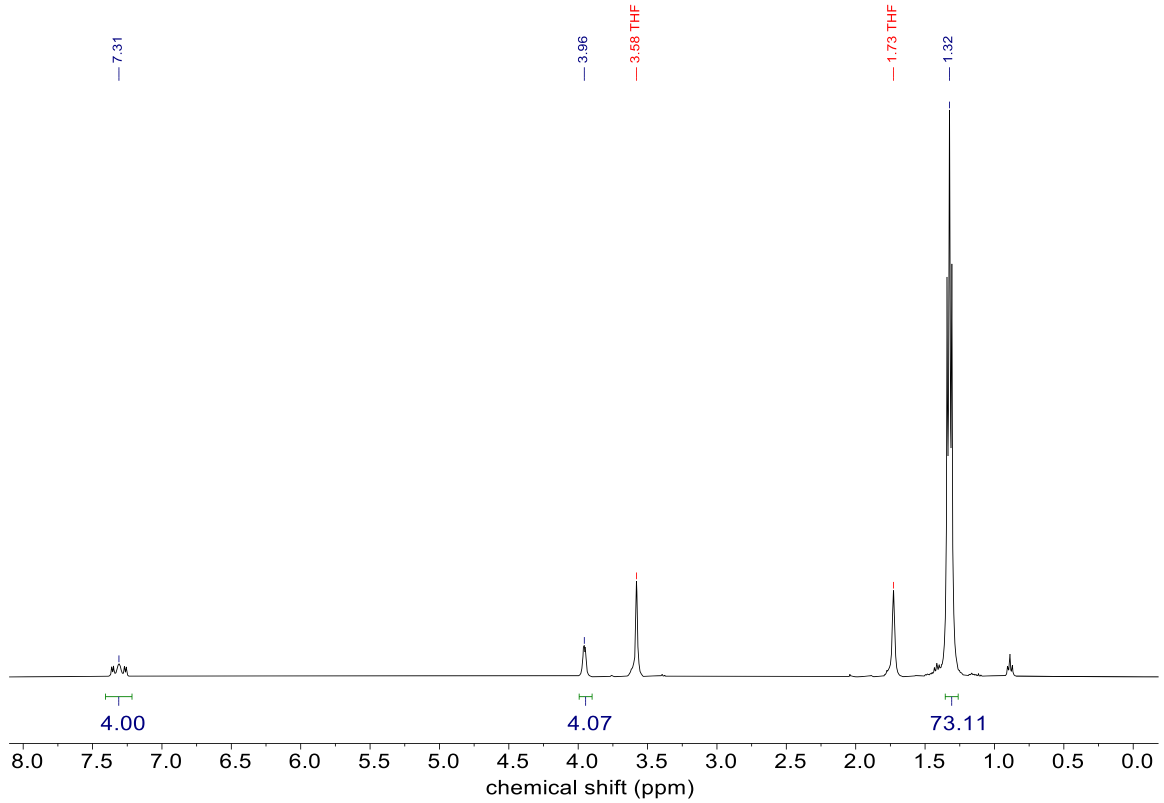

Figure S29: ^{1}H-NMR spectrum of [(μ-As_2){Pd(PNP)}$_2$] ($\mathbf{2}^{\mathbf{Pd,As}}$) in THF-$d_8$ at room temperature.

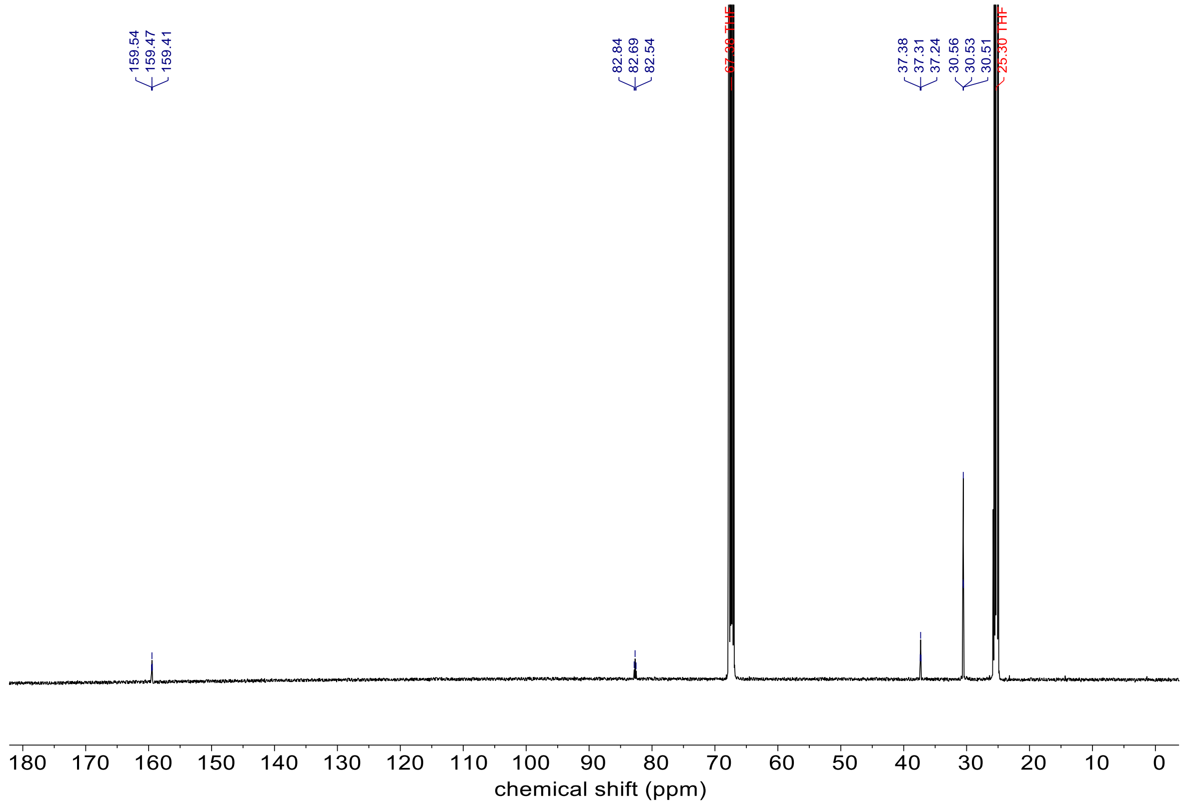

Figure S30: $^{13}C\{^1H\}$-NMR spectrum of [(μ-As_2){Pd(PNP)}$_2$] ($\mathbf{2}^{\mathbf{Pd,As}}$) in THF-$d_8$ at room temperature.

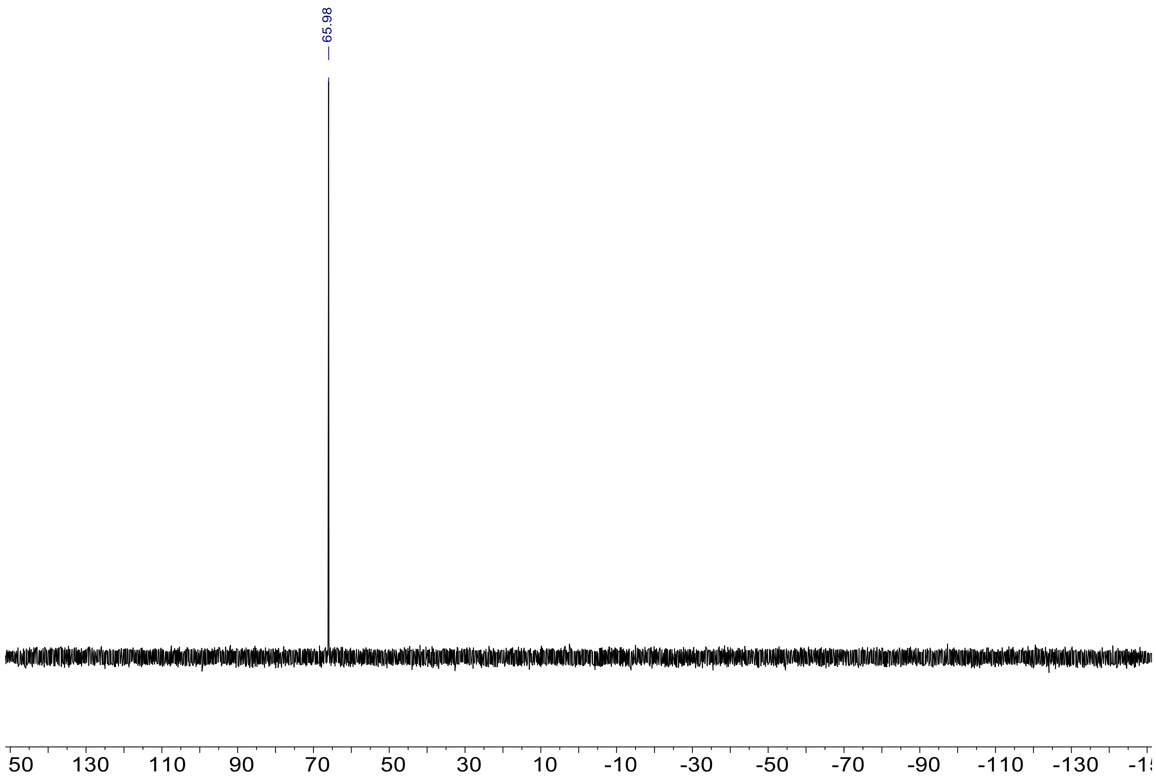

Figure S31: $^{31}P\{^{1}H\}$-NMR spectrum of [(μ-As_2){Pd(PNP)}$_2$] ($2^{Pd,As}$) in THF-d_8 at room temperature

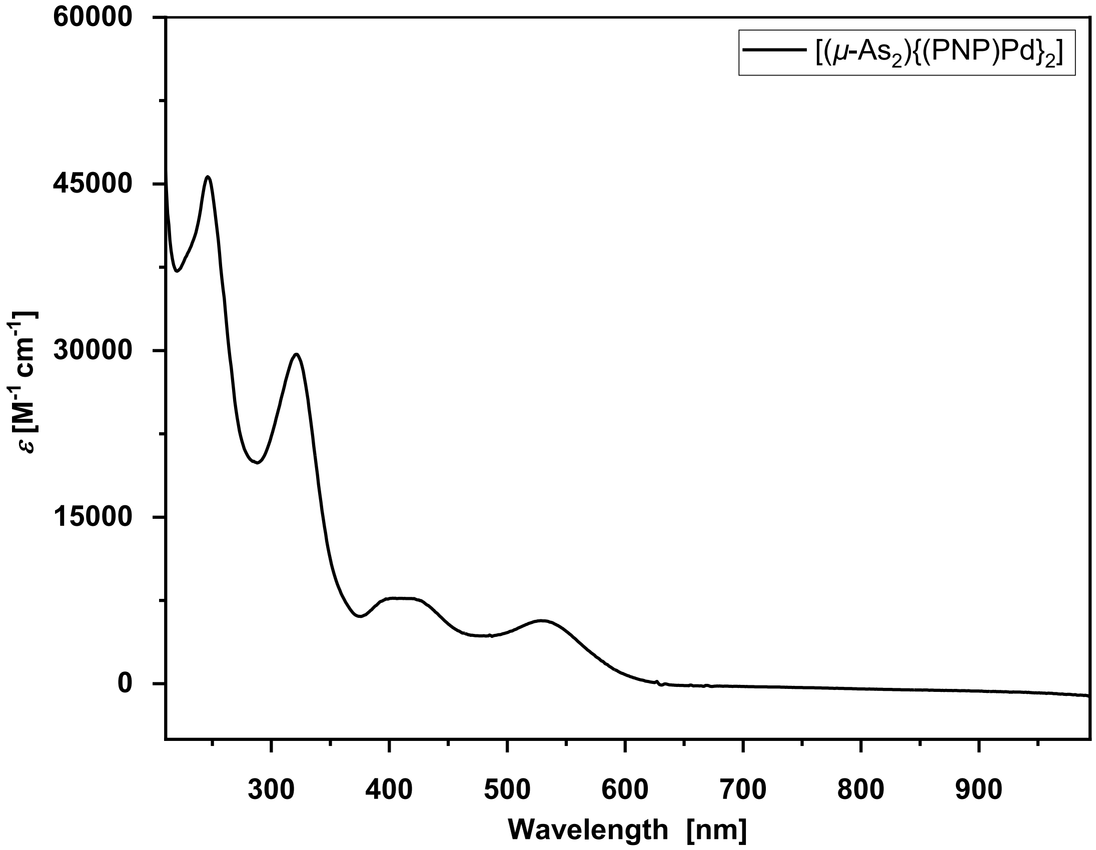

Figure S32: UV-vis spectrum of [(μ-As_2){Pd(PNP)}$_2$] ($2^{Pd,As}$) in THF at room temperature.

(Spectroscopic) Characterization of [(μ-As$_2$){(PNP)Pt}$_2$] (2^{Pt,As})

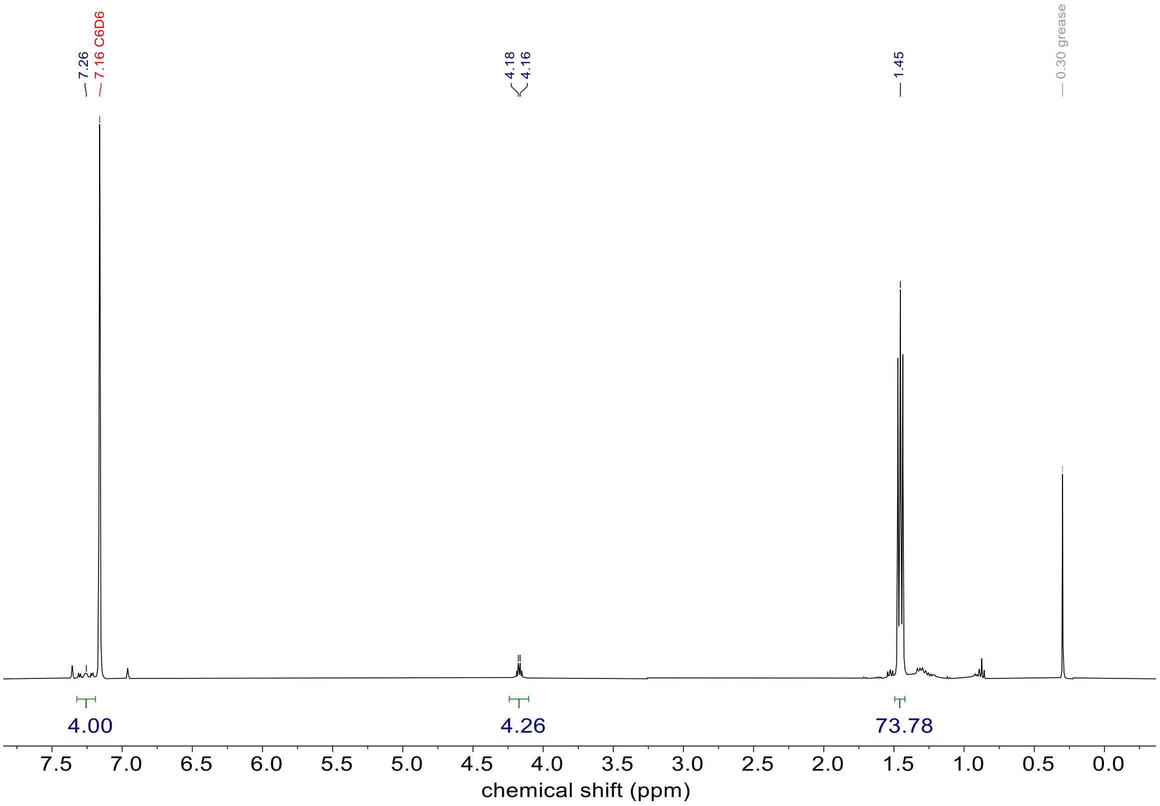

Figure S33: ^{1}H-NMR spectrum of [(μ-As$_2$){Pt(PNP)}$_2$] (**2^{Pt,As}**) in C_6D_6 at room temperature.

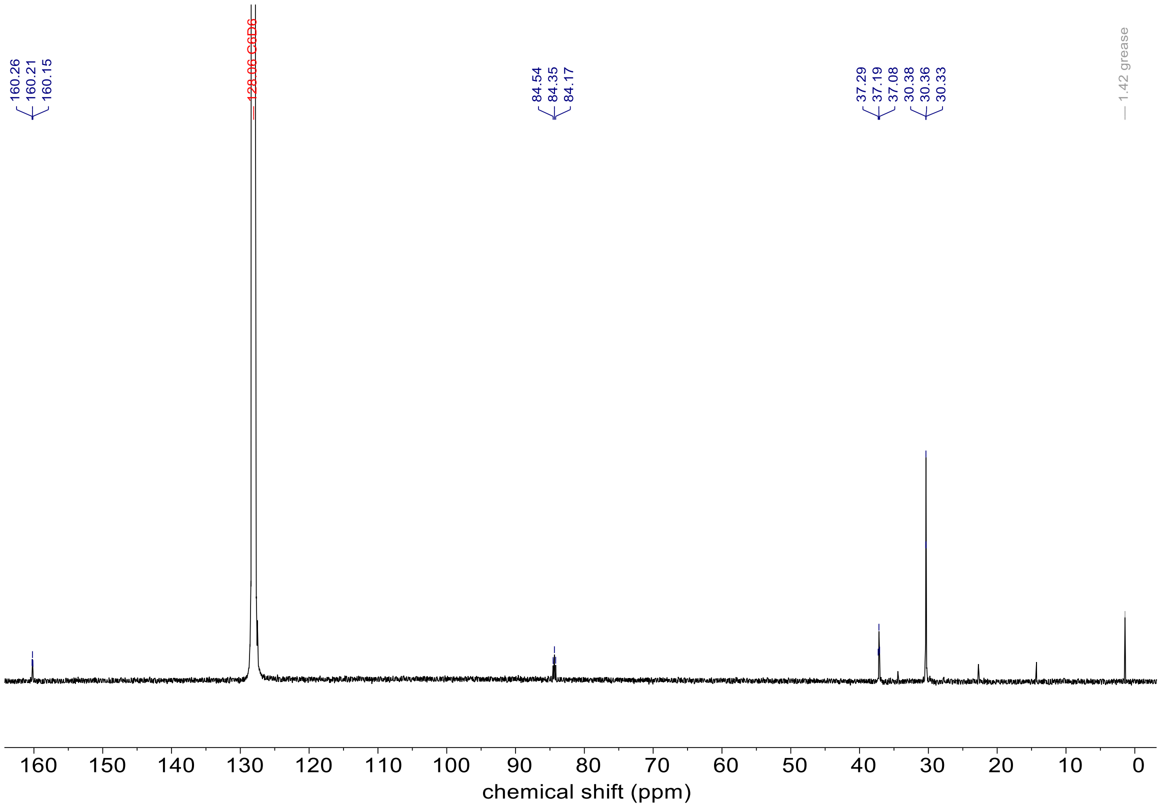

Figure S34: ^{13}C{^{1}H}-NMR spectrum of [(μ-As$_2$){Pt(PNP)}$_2$] (**2^{Pt,As}**) in C_6D_6 at room temperature.

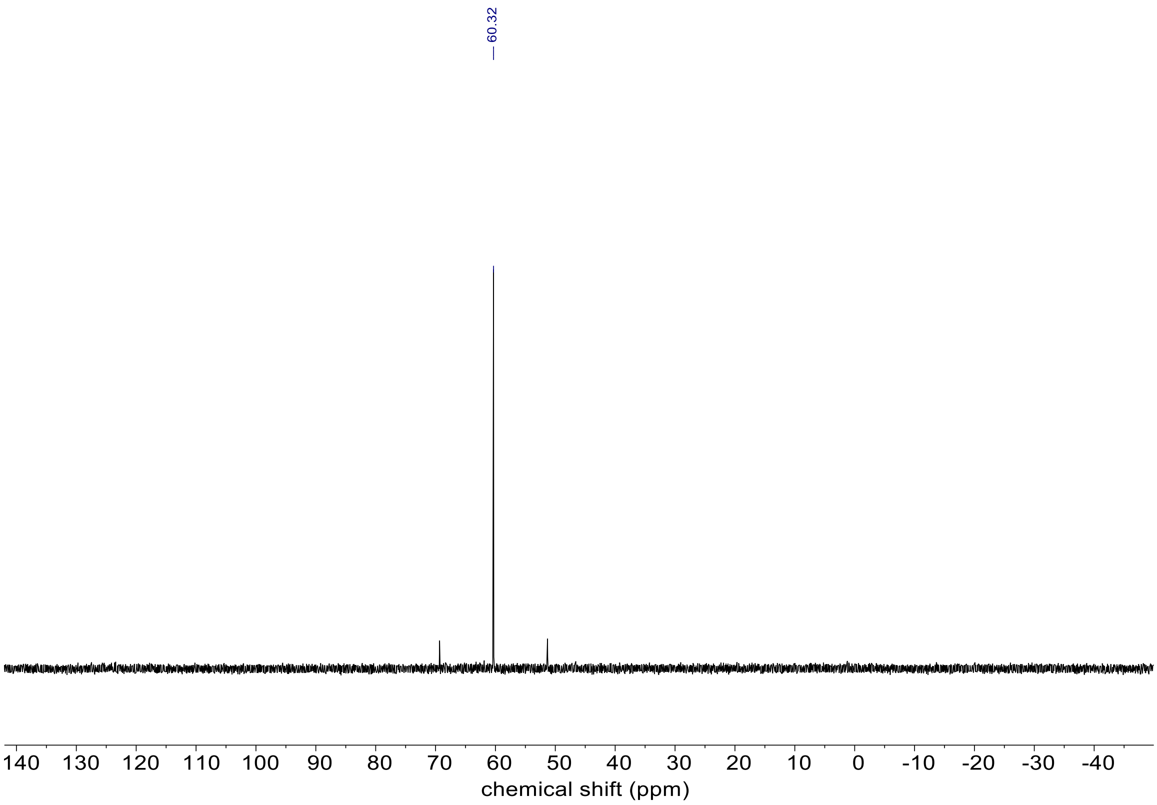

Figure S35: $^{31}P\{^{1}H\}$-NMR spectrum of [(μ-As_2){Pt(PNP)}$_2$] ($\mathbf{2^{Pt,As}}$) in C_6D_6 at room temperature.

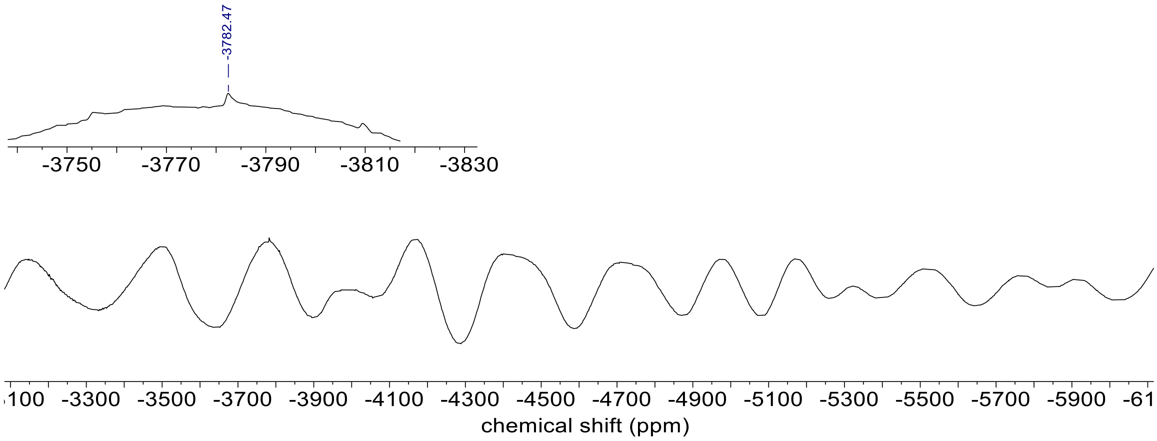

Figure S36: $^{195}Pt\{^1H\}$-NMR spectrum of [(μ-As_2){Pt(PNP)}$_2$] ($\mathbf{2^{Pt,As}}$) in C_6D_6 at room temperature.

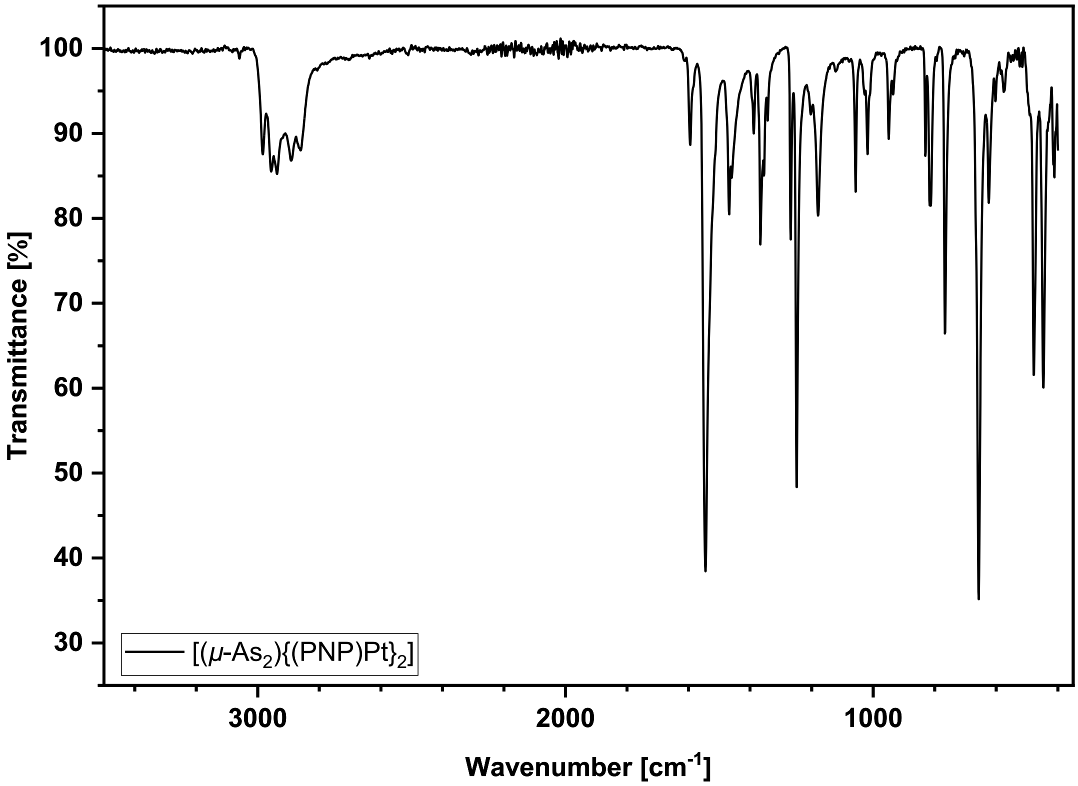

Figure S37: ATR-IR spectrum of [(μ-As_2){Pt(PNP)}$_2$] ($\mathbf{2^{Pt,As}}$).

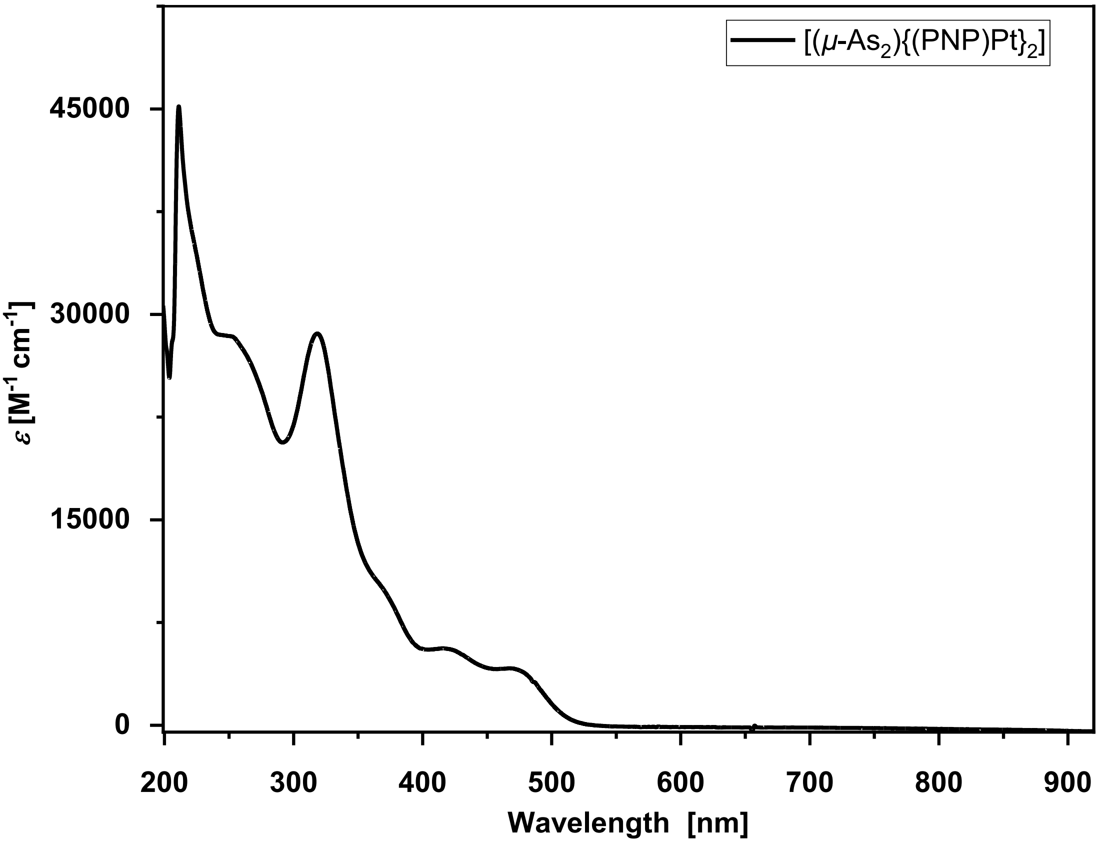

Figure S38: UV-vis spectra of [(μ-As$_2$){Pt(PNP)}$_2$] (**2**$^{\mathbf{Pt,As}}$) in THF at room temperature.

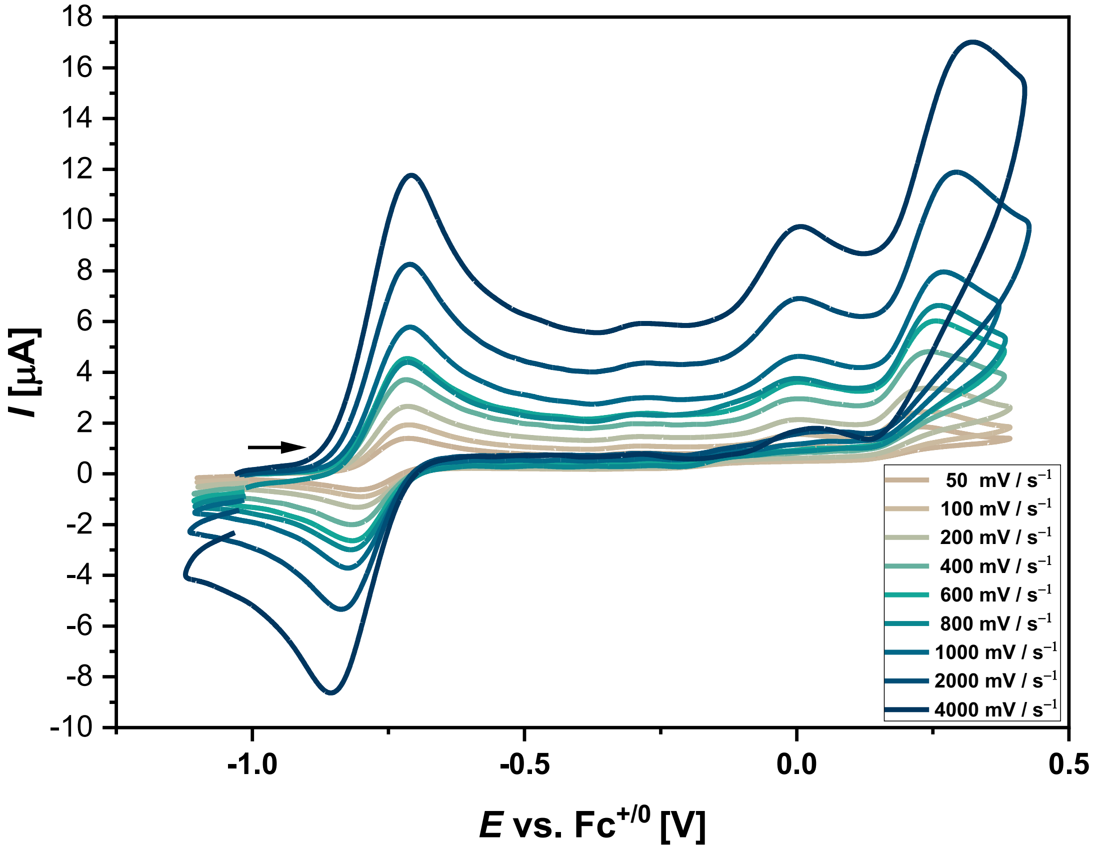

Figure S39: Scan-rate dependent cyclocloltammograms of [(μ-As$_2$){Pt(PNP)}$_2$] (**2**$^{\mathbf{Pt,As}}$) in PhF (c = 1 mM, I = 0.1 M [nBu$_4$N][BArF$_{24}$]) under Ar atmosphere.

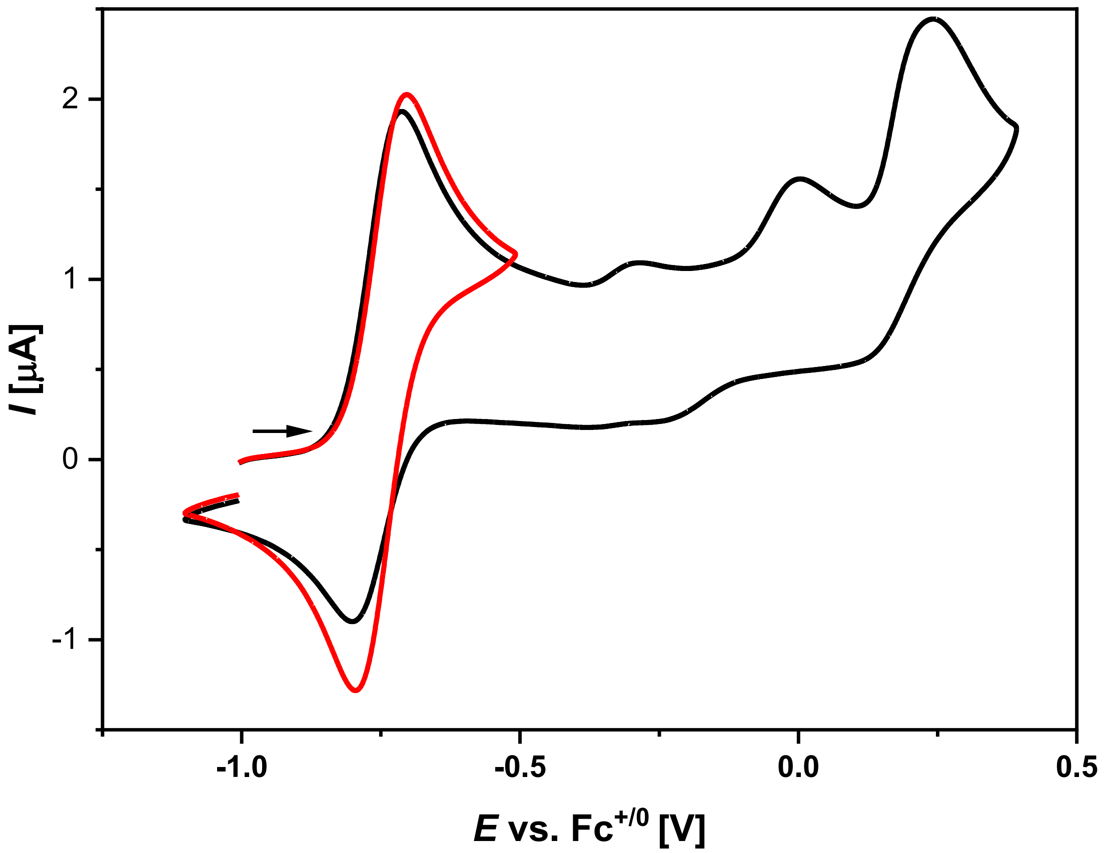

Figure S40: Cyclovoltammogram (v = 100 mVs^{-1}) of [(μ-As_2){Pt(PNP)}$_2$] ($\mathbf{2}^{\mathbf{Pt,As}}$) in PhF (c = 1 mM, I = 0.1 M [nBu_4N][$BArF_{24}$]) under Ar atmosphere.

Spectroscopic Characterization of [(μ-Sb_2){(PNP)Pt}$_2$] ($2^{Pt,Sb}$)

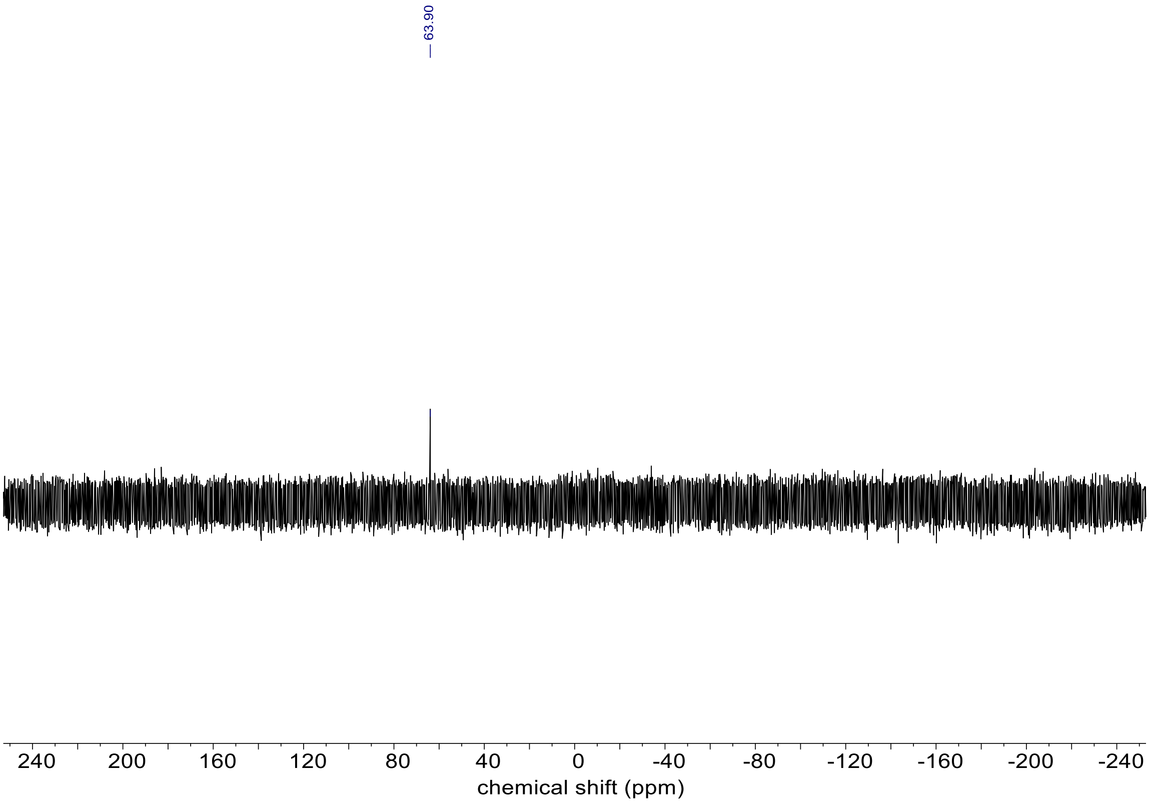

Figure S41: $^{31}P\{^1H\}$-NMR spectrum of a saturated solution of [(μ-Sb_2){Pt(PNP)}$_2$] ($\mathbf{2}^{\mathbf{Pt,Sb}}$) in THF-$d_8$ at room temperature.

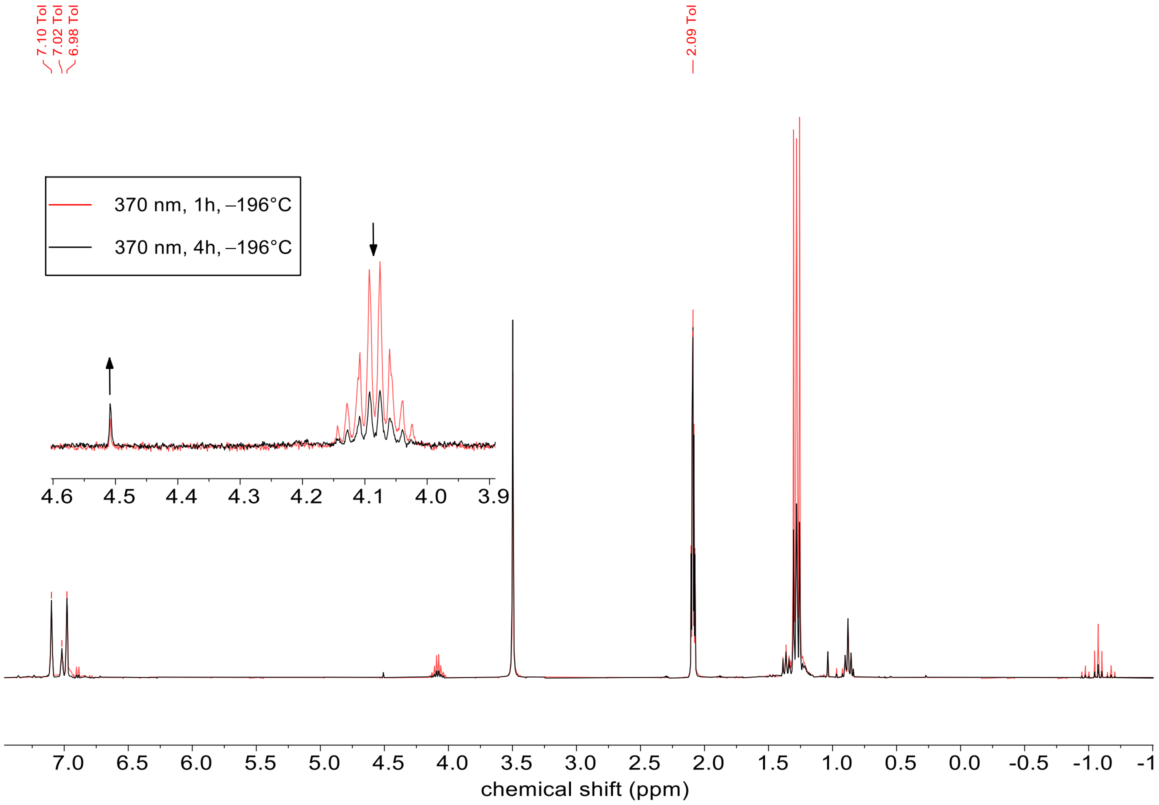

Figure S42: ^{1}H-NMR spectrum after photolysis of a frozen solution of [Pt(SbH$_2$)(PNP)] (**2^{Pt,Sb}**) in Tol-d$_8$ at room temperature.

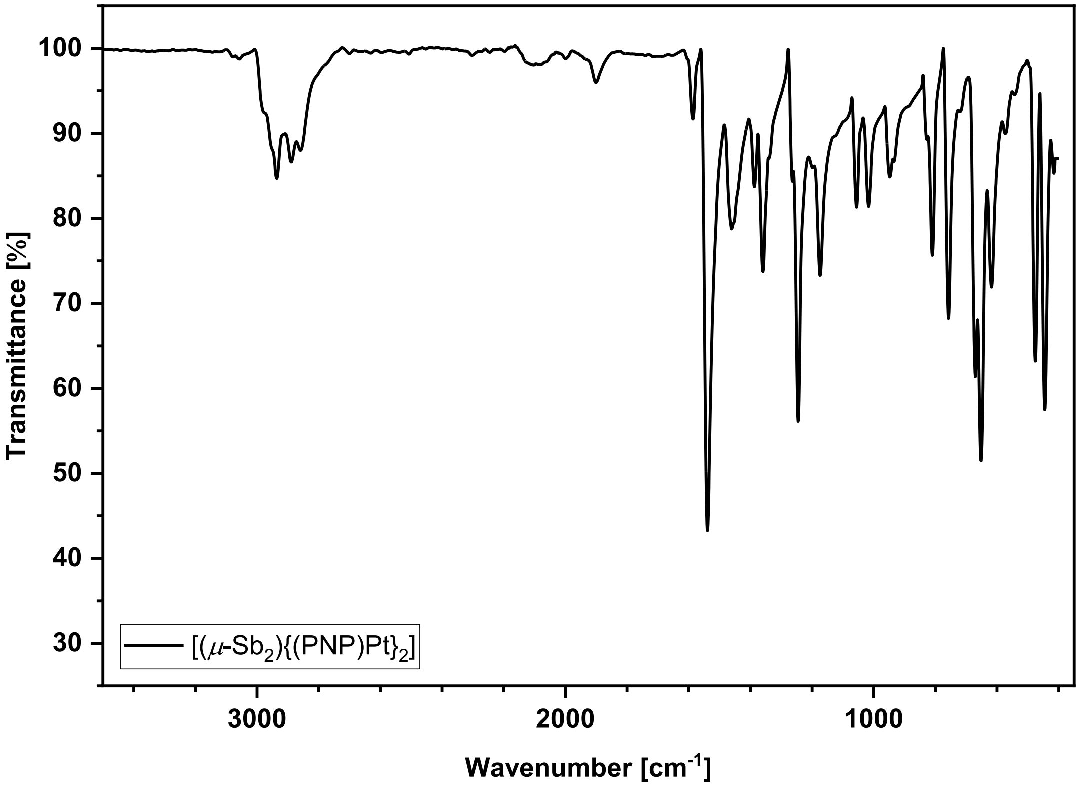

Figure S43: ATR-IR spectrum of [(μ-Sb$_2$){Pt(PNP)}$_2$ (**2^{Pt,Sb}**).

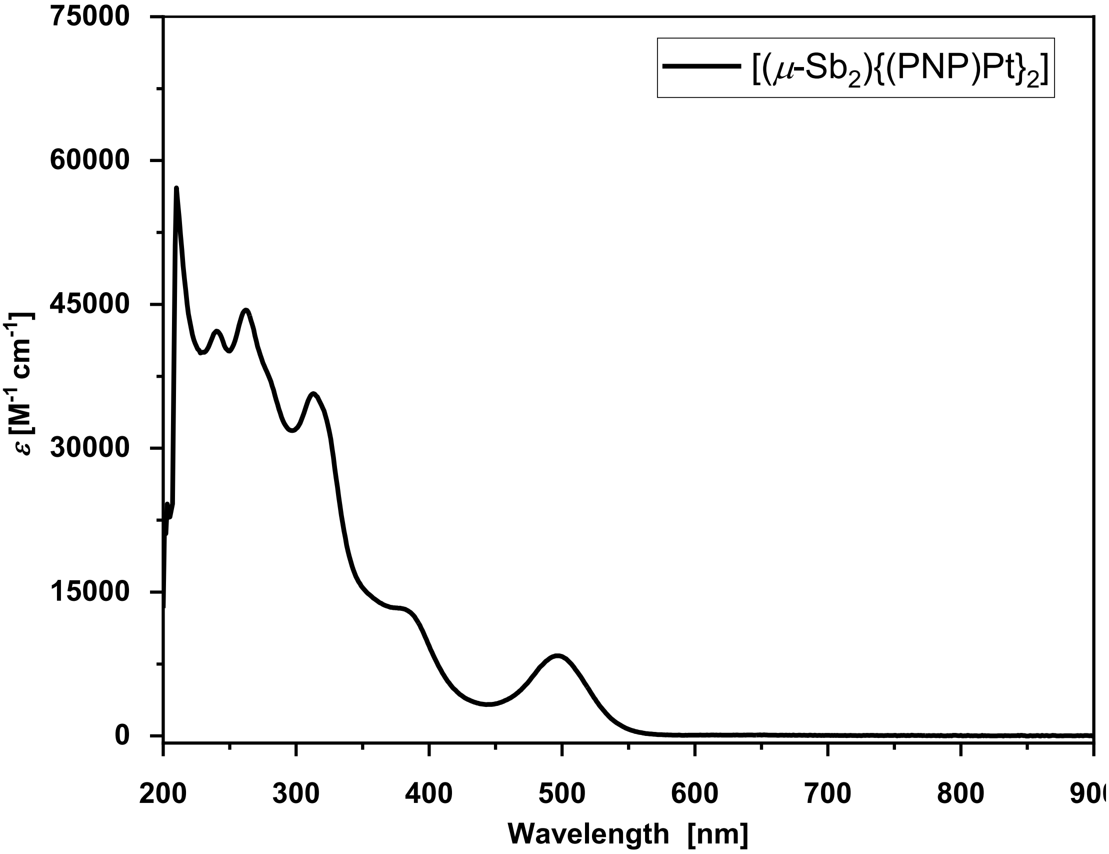

Figure S44: UV-vis spectrum of [(μ-Sb$_2$){Pt(PNP)}$_2$] ($\mathbf{2}^{\mathbf{Pt,Sb}}$) in THF at room temperature.

Spectroscopic Characterization of [(μ-Bi$_2$){(PNP)Pt}$_2$] (2^{Pt,Bi})

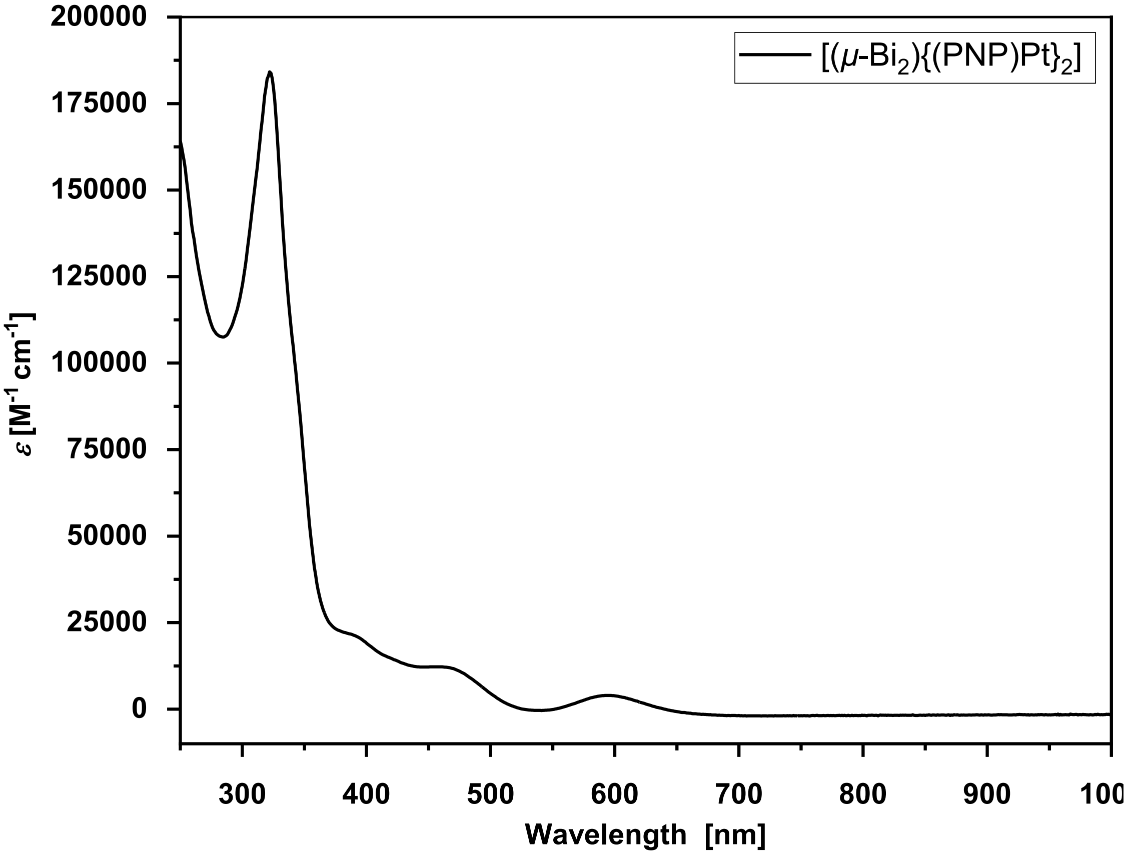

Figure S45: UV-vis spectrum of [(μ-Bi$_2$){Pt(PNP)}$_2$] (**2^{Pt,Bi}**) in MeTHF at room temperature.

Spectroscopic Characterization of [(μ-As$_2$){(PNP)Pt}$_2$]BArF$_{24}$ ([2^{Pt,As}]$^+$)

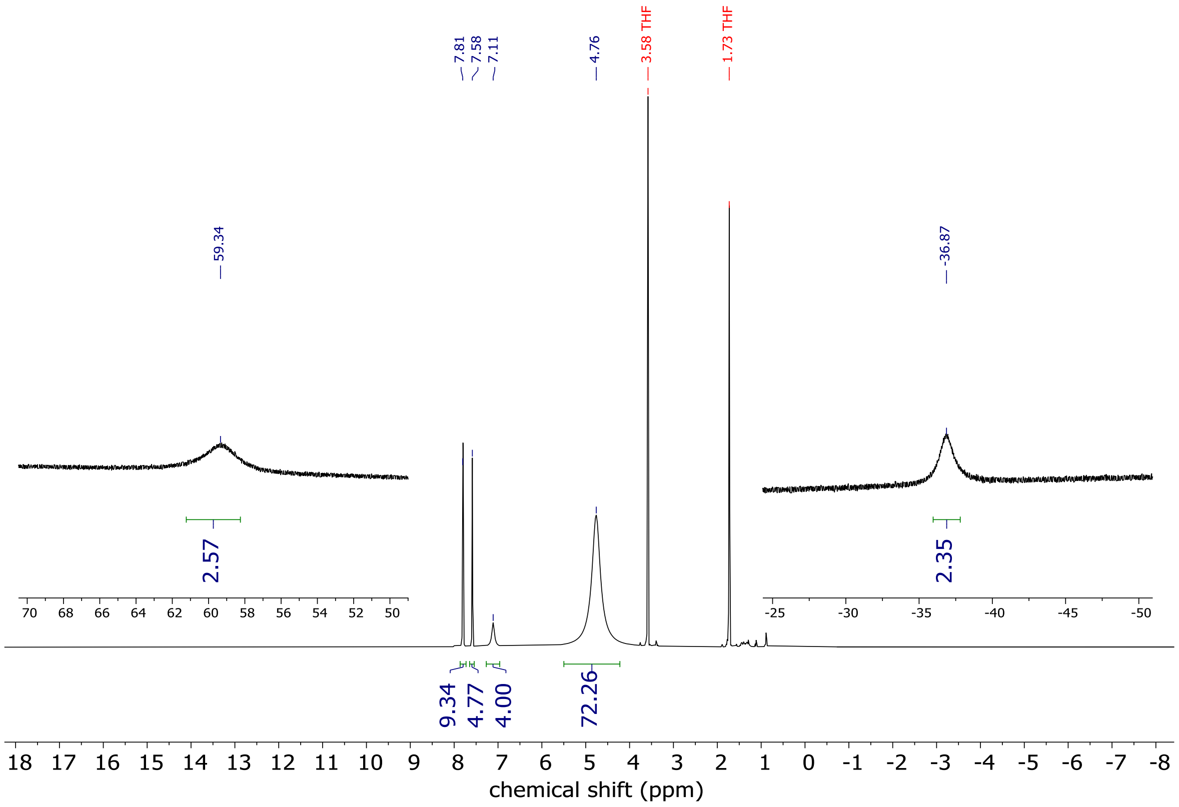

Figure S46: ^{1}H-NMR spectrum of [(μ-As$_2$){Pt(PNP)}$_2$]BArF$_{24}$ (**[2^{Pt,As}]$^+$**) in THF-d$_8$ at room temperature.

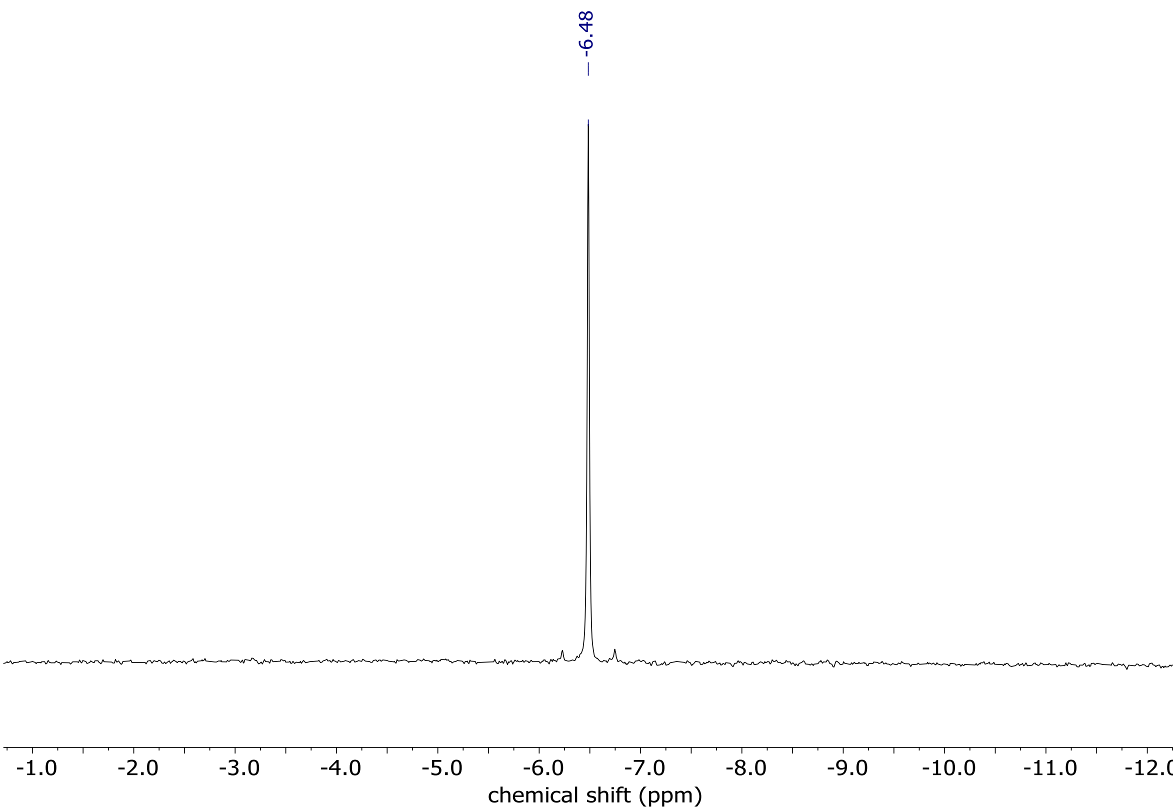

Figure S47: $^{11}B\{^{1}H\}$-NMR spectrum of [(μ-As_2){Pt(PNP)}$_2$]BArF$_{24}$ (**[2^{Pt,As}]$^+$**) in THF-d$_8$ at room temperature.

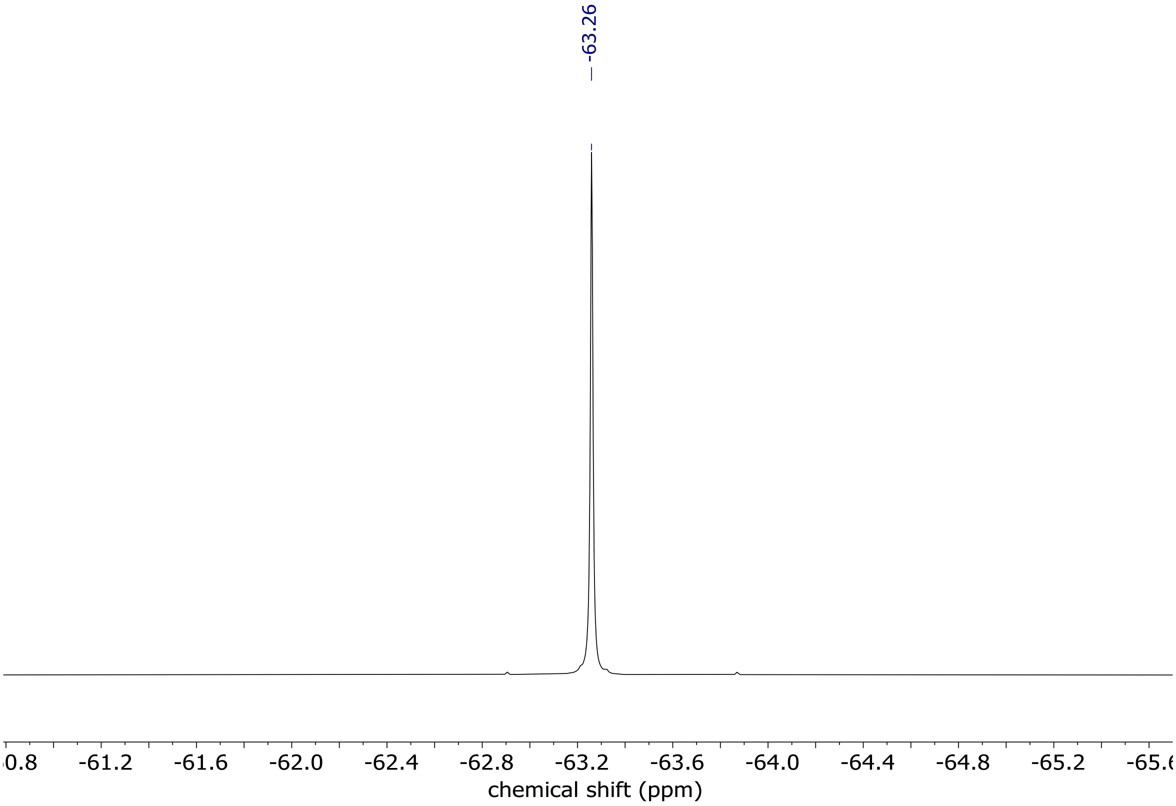

Figure S48: : $^{19}F\{^{1}H\}$-NMR spectrum of [(μ-As_2){Pt(PNP)}$_2$]BArF$_{24}$ (**[2^{Pt,As}]$^+$**) in THF-d$_8$ at room temperature.

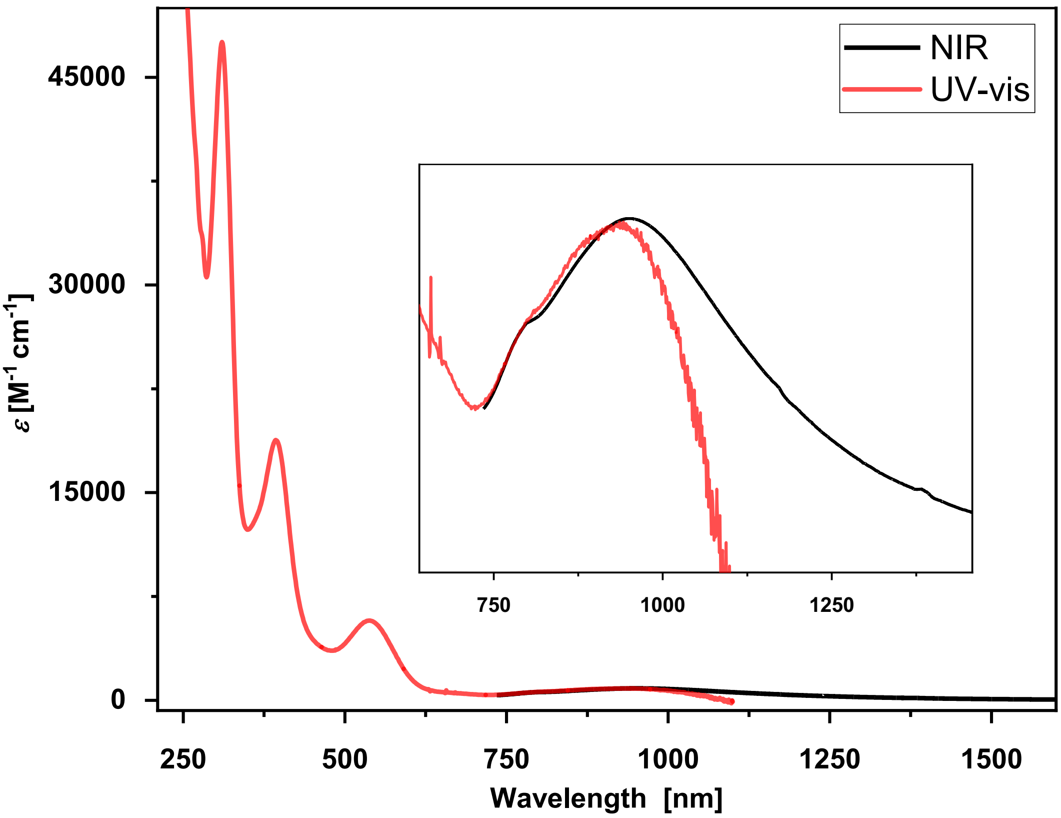

Figure S49: UV-vis (red line) and NIR (black line) spectra of [(μ-As$_2$){Pt(PNP)}$_2$]BArF$_{24}$ (**[2^{Pt,As}]$^+$**) in THF at room temperature.

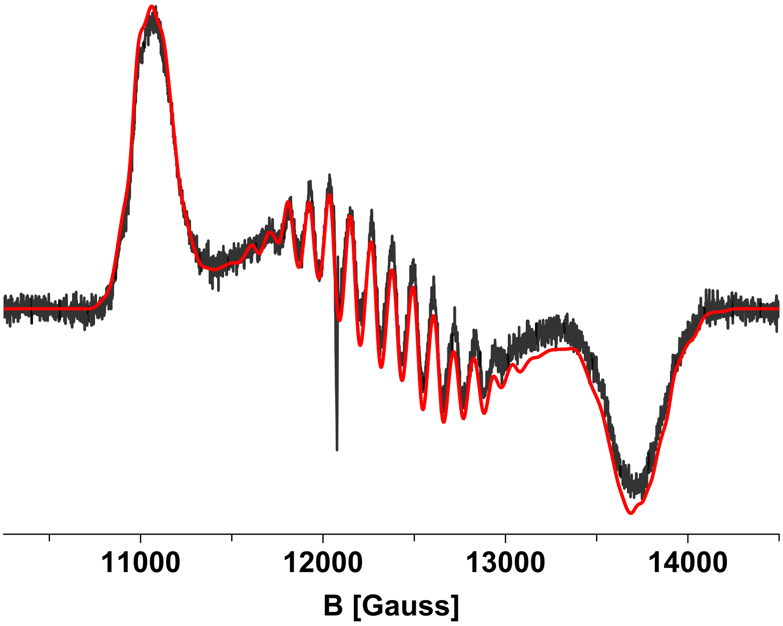

Figure S50: Experimental (black) and simulated (red) continuous wave Q-band EPR spectrum of [(μ-As$_2$){Pt(PNP)}$_2$]BArF$_{24}$ (**[2^{Pt,As}]$^+$**) in frozen MeTHF at 40 K. Experimental parameters: microwave frequency: 33.86 GHz; microwave power: 0.6 mW; field modulation amplitude: 20 G.

Table S1: Q-band EPR simulation parameters for [(μ-As$_2$){Pt(PNP)}$_2$]BArF$_{24}$ (**[2^{Pt,As}]$^+$**) (g = 2.1828, 1.9589, 1.7642); line width: 5.6 G.

Nuclei	$A = [A_x, A_y, A_z]$ / MHz	A_{iso} / MHz	Euler angles (α, β, γ) / °
^{75}As	75, 655, 119	283	-10.6, -95,7, -12.0
^{75}As	33, 315, 94	147	1.0, 10.2, 6.4
^{195}Pt	446, 1199, 685	479	53.6, -23.2, -27.7
^{195}Pt	347, 1015, 45	237	59.9, 116, -54.3

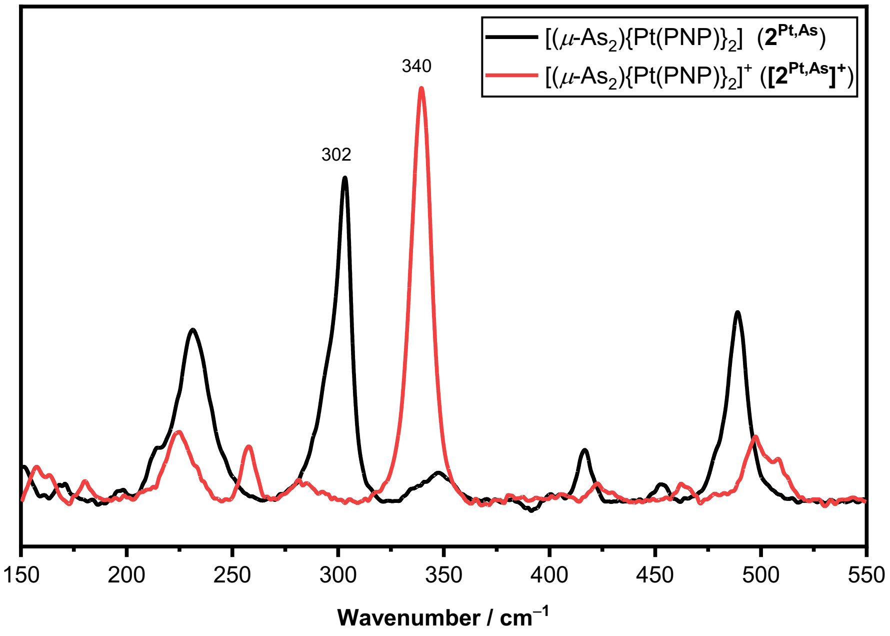

Figure S51: Resonance raman spectra (λ_{exc} = 633 nm) of [(μ-As_2){Pt(PNP)}$_2$] (**[2^{Pt,As}]$^+$**), black line) and [(μ-As_2){Pt(PNP)}$_2$]BArF$_{24}$ ((**[2^{Pt,As}]$^+$**), red line)

In situ SQUID measurements

SQUID measurements were carried out using a Quantum Design MPMS3 SQUID magnetometer. The metallopnictinidene complexes [M(Pn)(PNP)] (**3**^{M,Pn}) (M =Pd, Pt; Pn = P, As) were formed by photolysis of the corresponding pnictaethynolate complexes [M(PnCO)(PNP)] (**1**^{M,Pn}) (M =Pd, Pt; Pn = P, As) with a TLS120Xe xenon light source (456 nm (**1**^{Pd,P}), 570nm (**1**^{Pd,As}), 482 nm (**1**^{Pt,P}), 517 nm (**1**^{Pt,As})), in combination with a fiber optical sample holder (FOSH) to get optical access to the sample. The sample was placed in the FOSH and centered in the magnetometer coils, cooled to 2 K and measured to 295 K at a magnetic field of 500 mT to obtain a background measurement prior to photoproduct formation. After that, the sample was cooled to 5K followed by a temperature stabilization period of 30 minutes and subsequent photolysis at the respective wavelength. The photochemical progress was monitored by following the rise in the DC moment over the course of the irradiation until saturation was observed (approx. after 180 min).

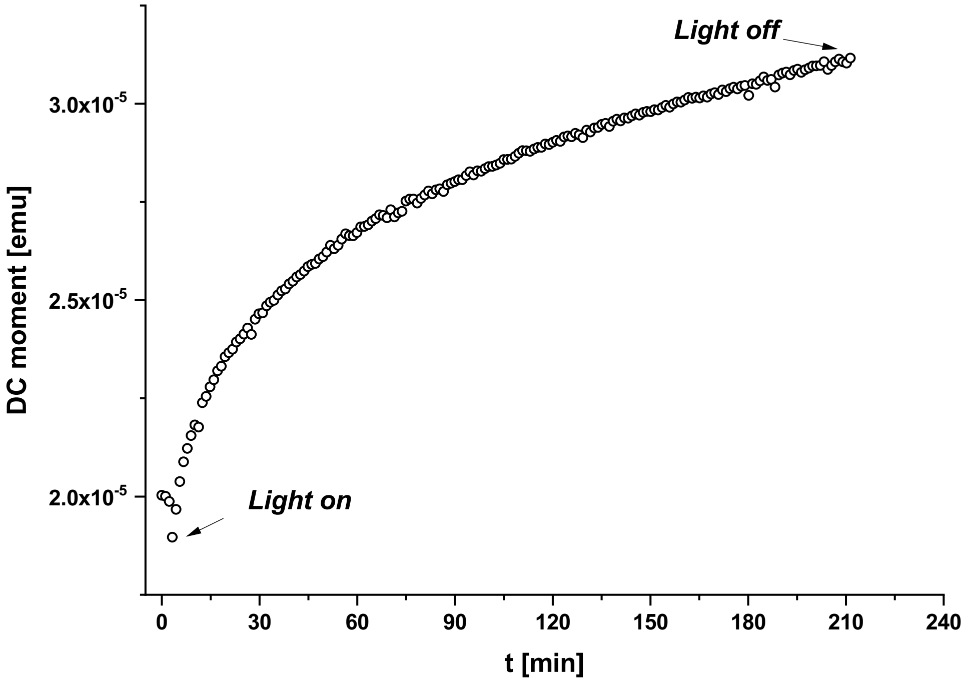

Figure S52: Increase of the DC moment upon photolysis of [Pd(PCO)(PNP)] (**1**^{Pd,P}) at λ_{exc} = 456 nm and *T* = 5 K under an applied magnetic field of 500 mT.

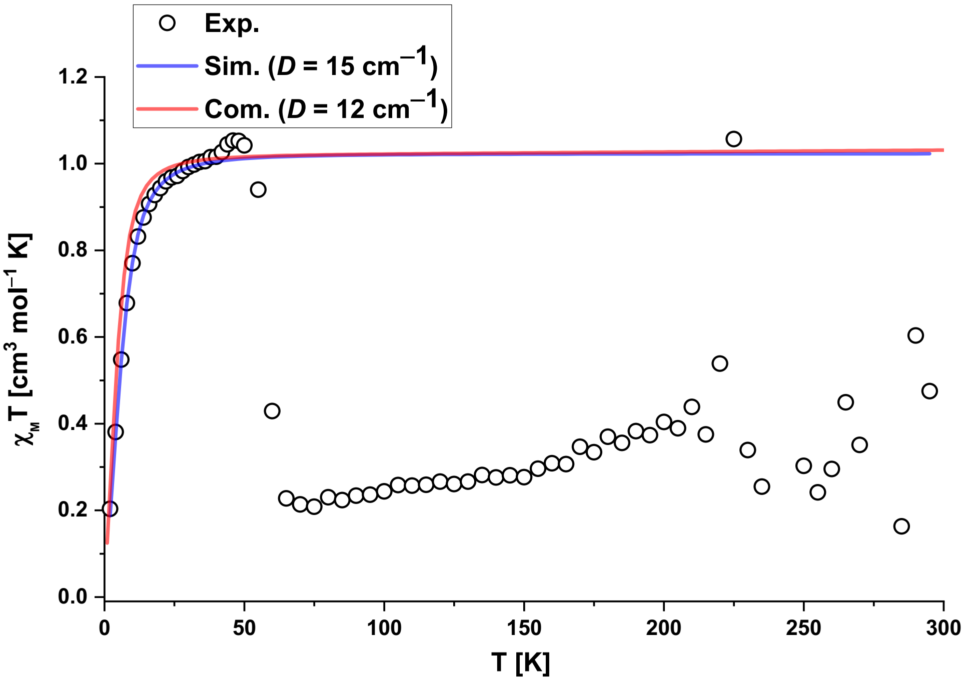

Figure S53: $\chi_M T$ vs *T* data (circles) of in situ formed **3**^{Pd,P} obtained by SQUID magnetometry with simulated (red line) and computed (blue line) values. Simulation parameters: g = 2.02 (fixed) and *D* = 15 cm^{-1}.

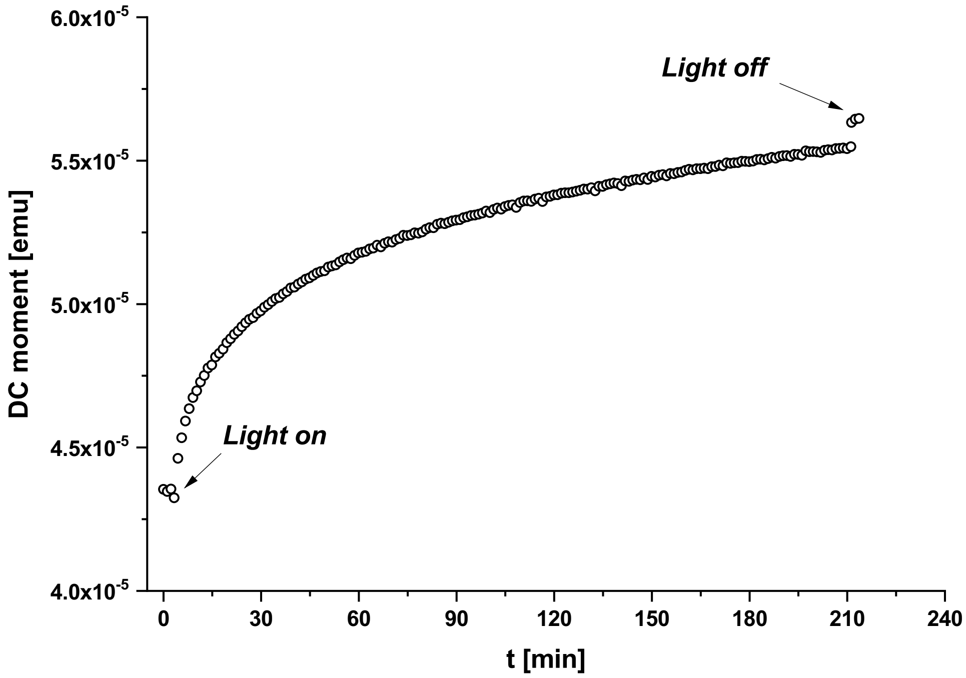

Figure S54: Increase of the DC moment upon photolysis of [Pd(AsCO)(PNP)] (**1**^{Pd,As}) at λ_{exc} = 570 nm and T = 5 K under an applied magnetic field of 500 mT.

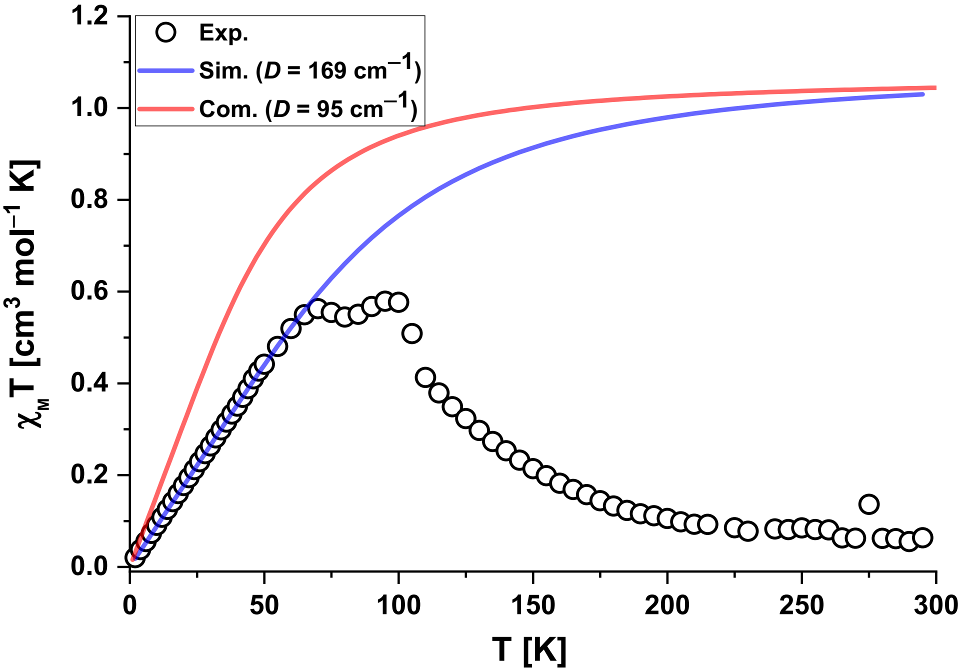

Figure S55: $\chi_M T$ vs T data (circles) of in situ formed **3**^{Pd,As} obtained by SQUID magnetometry with simulated (red line) and computed (blue line) values. Simulation parameters: g = 2.07 (fixed) and D = 169 cm^{-1}.

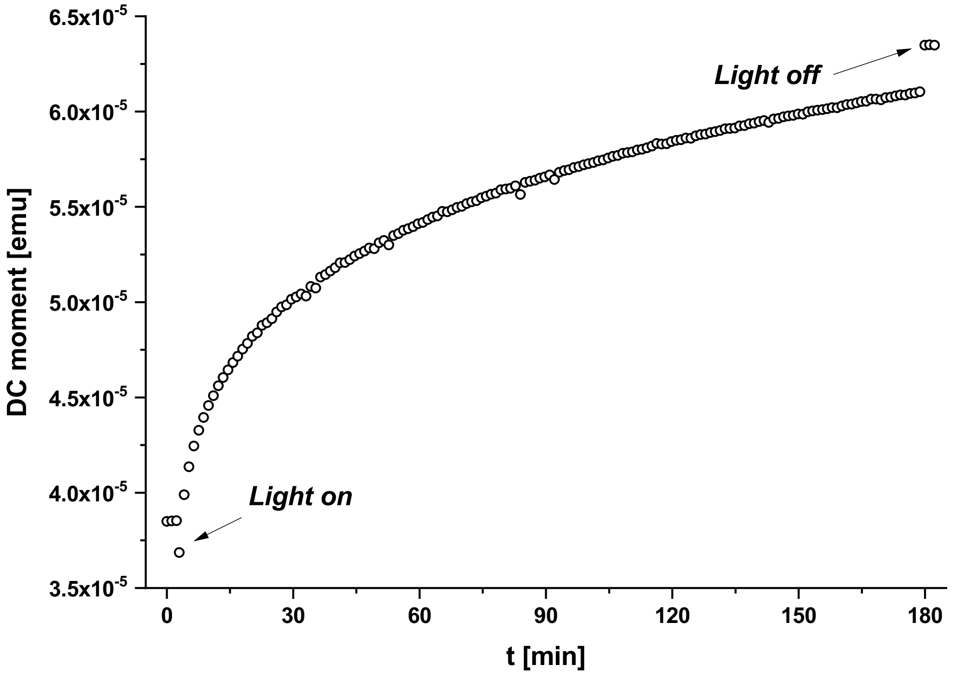

Figure S56: Increase of the DC moment upon photolysis of [Pt(PCO)(PNP)] (**1**$^{\mathbf{Pt,P}}$) at λ_{exc} = 482 nm and T = 5 K under an applied magnetic field of 500 mT.

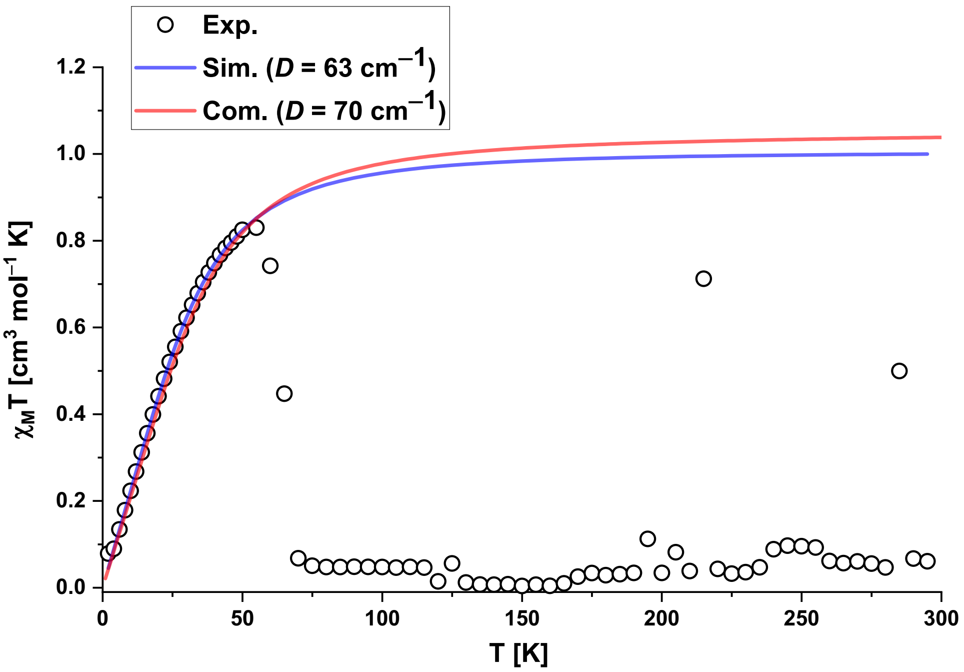

Figure S57: $\chi_M T$ vs T data (circles) of in situ formed **3**$^{\mathbf{Pt,P}}$ obtained by SQUID magnetometry with simulated (red line) and computed (blue line) values. Simulation parameters: g = 2.01 (fixed) and D = 63 cm^{-1}.

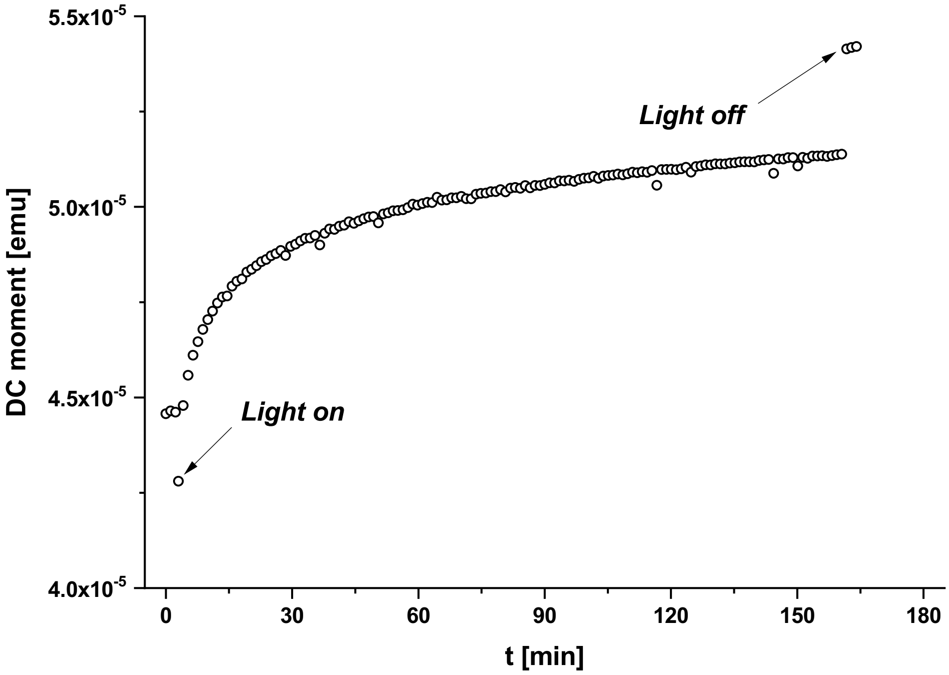

Figure S58: Increase of the DC moment upon photolysis of [Pt(AsCO)(PNP)] ($\mathbf{1}^{\mathbf{Pt,As}}$) at λ_{exc} = 517 nm and T = 5 K under an applied magnetic field of 500 mT.

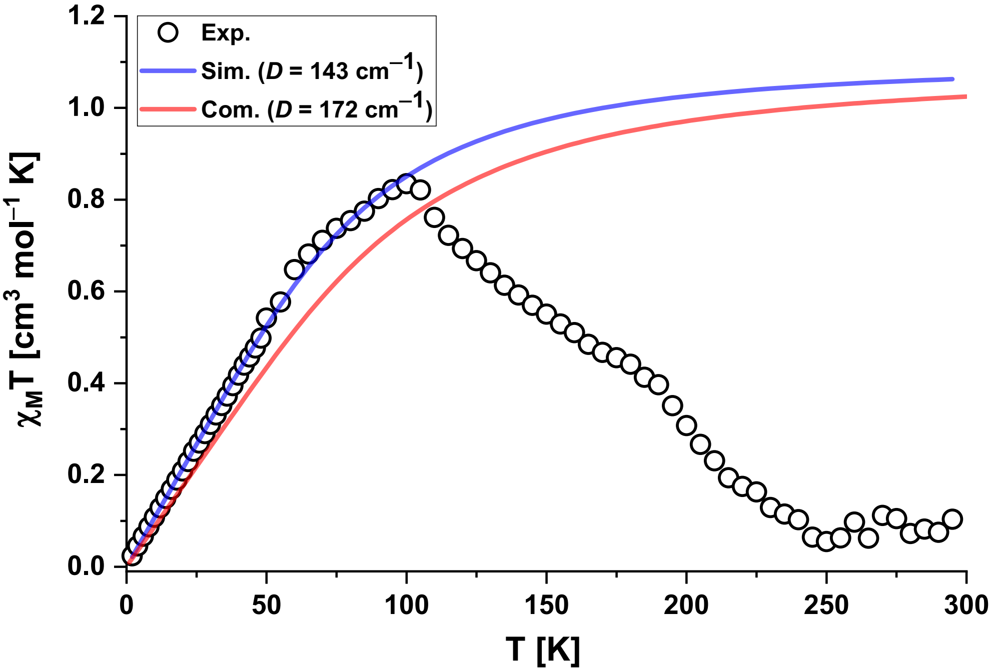

Figure S59: $\chi_M T$ vs T data (circles) of in situ ($\mathbf{3}^{\mathbf{Pt,As}}$ obtained by SQUID magnetometry with simulated (red line) and computed (blue line) values. Simulation parameters: g = 2.09 (fixed) and D = 143 cm^{-1}.

THz-EPR Spectroscopy

Sample preparation and measurement conditions

Samples for mid-infrared reaction monitoring were prepared by grinding some grains of [M(AsCO)(PNP)] (**1**^{M,As}) (M = Pd, Pt) with 120mg KBr in an agate mortar and pressing to a pellet of 10 mm diameter at about 1500 psi under N_2 atmosphere. The sample was transferred in a bath of liquid N_2, into the cryostat of the spectrometer. Samples for THz-EPR spectroscopy measurements were prepared identically to the MIR samples but with generally more sample material and high-density polyethylene (HDPE) powder as matrix (see Table S2).

Table S2: Sample preparation for THz-EPR measurements.

Sample	Sample mass / mg	HD-PE mass / mg
[(PNP)Pd-AsCO] (**1**^{Pd,As})	45.0	150
[(PNP)Pt-AsCO] (**1**^{Pt,As}) (1)	40.6	150
[(PNP)Pt-AsCO (**1**^{Pt,As}) (2)	28.2	90

Mid-infrared reaction monitoring was performed at the THz beamline at BESSY II using a Bruker IFS 125 FTIR spectrometer with the OPUS 8.5 software (see Figure S60 (A)).[12] The spectrometer was equipped with a liquid nitrogen cooled HgCdTe detector (MCT-12.5-2, InfraRed associates), an KBr beamsplitter and a helium gas flow cryostat (Opitstat, Bruker). The temperature was regulated via an ITC 503S (Oxford Instruments). The unpolarized MIR beam passes the sample placed in the cryostat through the optical windows. Optical excitation was achieved by coupling a light source through 2 mm thick quartz (Suprasil) windows into the spectrometer and guiding the beam perpendicular to the MIR beam, while the sample was rotated at 45° ± 5° to match both, the laser and the MIR beam. The samples were photolyzed with a HeNe laser (532 nm, 10 mW, LD532-10-24(20x80)-PL-C1500, Pictronic GmbH). Spectra were recorded in a range from 1 to 6000 cm^{-1} with a resolution of 2 cm^{-1} in transmission mode. The signal from the detector was pre-amplified (MCT-1000H, InfraRed associates) before it was fed back into the spectrometer interface for fast Fourier transformation. Transmission was calculated by dividing the raw spectrum of the respective sample by the raw spectrum of the empty cryostat.

Frequency-domain Fourier-transform electron paramagnetic resonance (FD-FT THz-EPR) spectroscopy measurements were performed at the THz beamline at BESSY II using a Bruker IFS 125 FTIR spectrometer with the OPUS 8.5 software equipped with a 6 µm Mylar beamsplitter, a superconducting 12 T magnet (J4777, Cryogenic, Inc.), and a Si-bolometer detector (HDL-5, Infrared Labs) cooled to 4.2 K with liquid He (see Figure S60 (B)).[12] The FIR beam was linearly polarized by a grid polarizer and passes the sample placed in the variable temperature insert (VTI) of the magnet in Voigt geometry with the B_1 magnetic field perpendicular to the B_0 field through 0.75 mm thick diamond windows. For optical excitation, an HeNe laser (532 nm, 10 mW, LD532-10-24(20x80) -PL-C1500, Pictronic GmbH) was coupled through a quartz window into the spectrometer and guided parallel to the FIR beam. Spectra were recorded in a range from 1 to 650 cm^{-1} with a resolution of 2 cm^{-1} in transmission mode. The signal from the detector was filtered (625 cm^{-1} LPF for **1**^{Pd,As} and 1000 cm^{-1} LPF for **1**^{Pt,As}) before it was fed back into the spectrometer interface for fast Fourier transformation. The relative transmission was calculated as magnetic-field-division-spectra (MDS) by dividing two raw spectra recorded at different magnetic-field strengths. Spectral simulations were performed using the THz-EPR functions[13] of the *EasySpin* toolbox[11] for MATLAB R2021b. The MDS were calculated from the simulated absorbance spectra $A(\tilde{\nu})$ by $T(\tilde{\nu}) = 10^{A(\tilde{\nu},B_j)-A(\tilde{\nu},B_i)}$. The spectra were simulated and scaled globally for best fitting the experimental data. The errors were estimated by individually varying the simulation parameters and visually inspecting the agreement of the simulation with the experimental data.

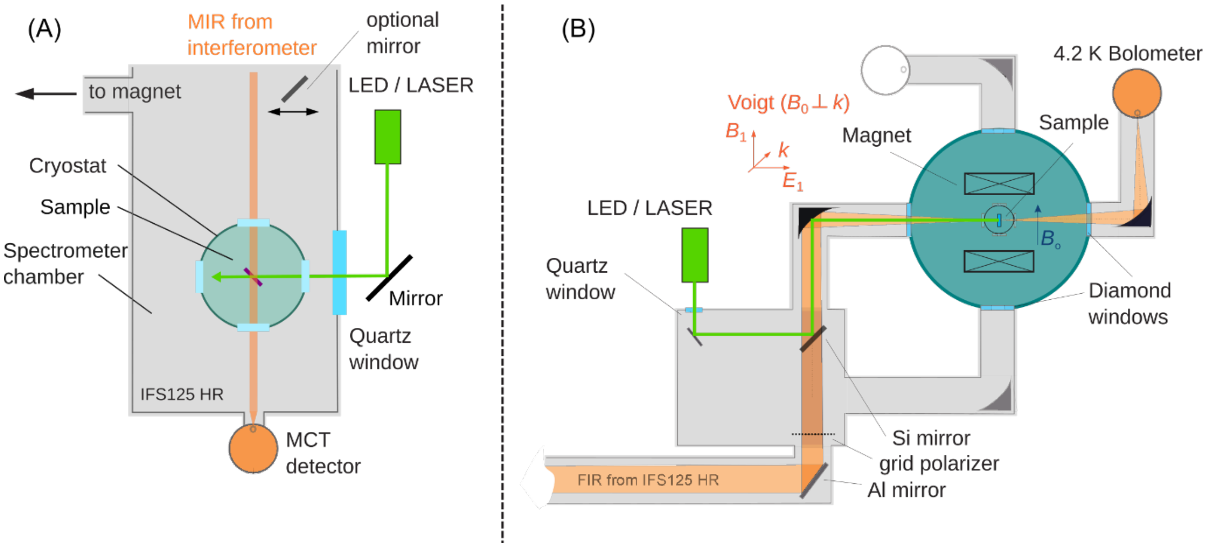

Figure S60: Schematic of the IR (a) and THz-EPR setup (b) including optical illumination.

Mid-IR reaction monitoring of the photoreactions

Prior to THz-EPR examination of the photoproducts, the photolysis conditions were optimized by mid-IR monitoring of the photochemical reaction progress, i.e., the decay of the AsCO stretching vibration (around 1860 cm^{-1}) and concomitant rise of the band for free CO (around 2100 cm^{-1}) under photolytic conditions (Figures S61a/b and S62a/b). The kinetic traces of the relative transmissions (T(t)/T(t=0)) at the spectral positions of the bound (Figures S61c and S62c) and free CO (Figures S61d and S62d) vibrations indicate partial conversion of precursor $\mathbf{1}^{Pd,As}$ after 200 min and rapid, quantitative conversion of $\mathbf{1}^{Pt,As}$ within about 30 min. The AsCO kinetic traces could be modelled with a bi-exponential decay function, $f(t) = a_0 - a_1 \exp(-k_1 t) - a_2 \exp(-k_2 t)$, using the parameters listed in Table S3 (red-dashed lines in Figures S61c and S62c). After photolysis, no changes of the IR spectra were observed over the the course of 150 minutes, confirming thermal stability under the conditions of the THz-EPR measurements (see below).

Table S3: Assignment of the AsCO stretching bands and rate constants *k* and fractions *a* of the corresponding exponential functions used to fit of the time-dependent transmission data during photodissociation (see text for details).

Sample	IR band / cm^{-1}	a_1	k_1 / min^{-1}	a_2	k_2 / min^{-1}
PdAsCO ($\mathbf{1}^{Pt,As}$)	1855	0.679(3)	−0.0099(2)	0.321(5)	−0.101(3)
PtAsCO ($\mathbf{1}^{Pt,As}$)	1858	0.86(2)	−1.81(6)	0.139(6)	−0.231(9)

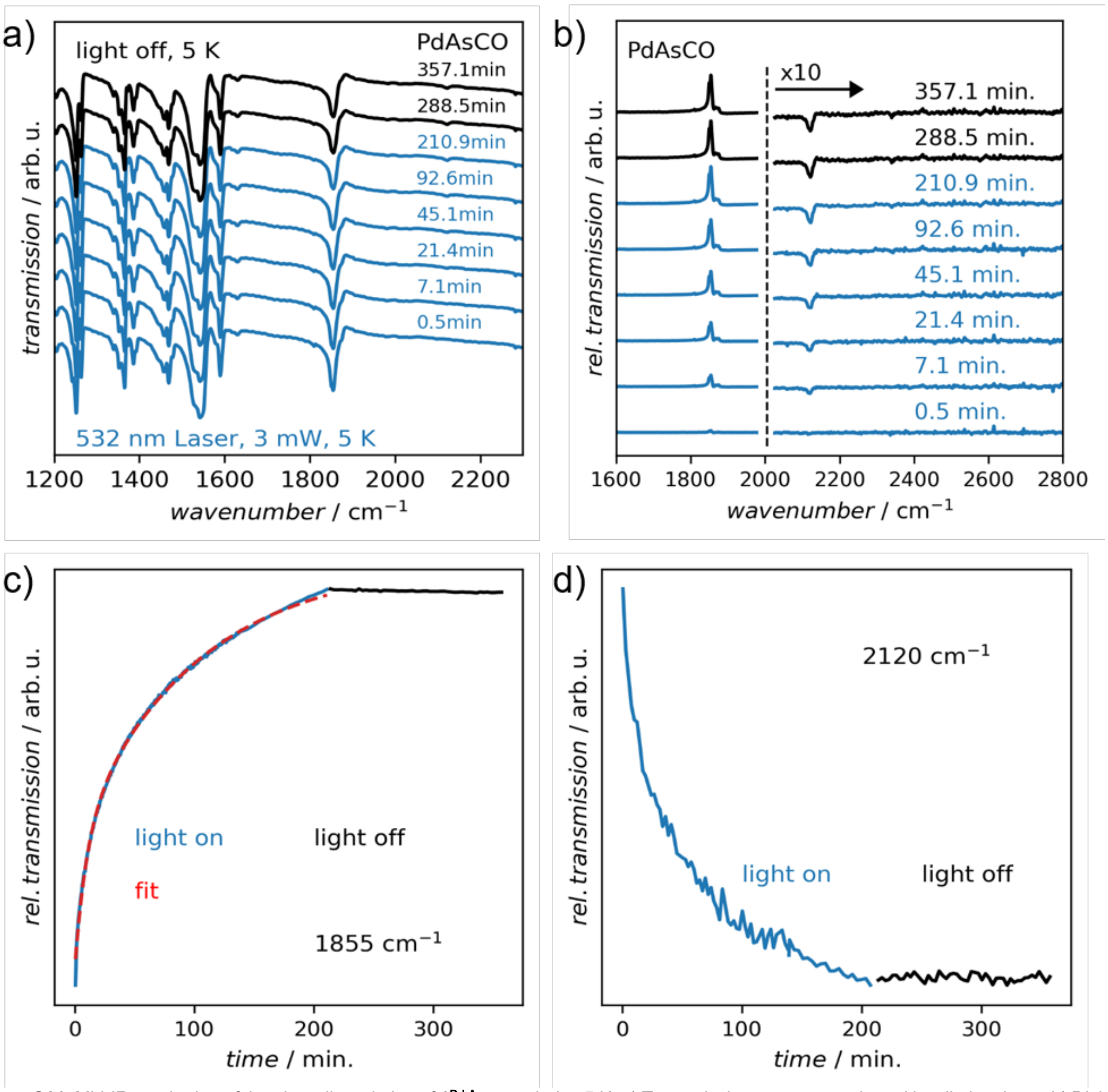

FigureS61: Mid-IR monitoring of the photodissociation of $\mathbf{1}^{Pd,As}$ recorded at 5 K. a) Transmission spectra at selected irradiation times. b) Division spectra referenced at the spectrum before irradiation. c) Relative transmission at the spectral position of the bound CO (1855 cm^{-1}) against photolysis time (blue) and against time after end of photolysis (black); the dashed red curve shows a fit on the experimental data during irradiation. d) Relative transmission at the spectral position of the free CO (2120 cm^{-1}) against photolysis time (blue) and against time stopping photolysis (black).

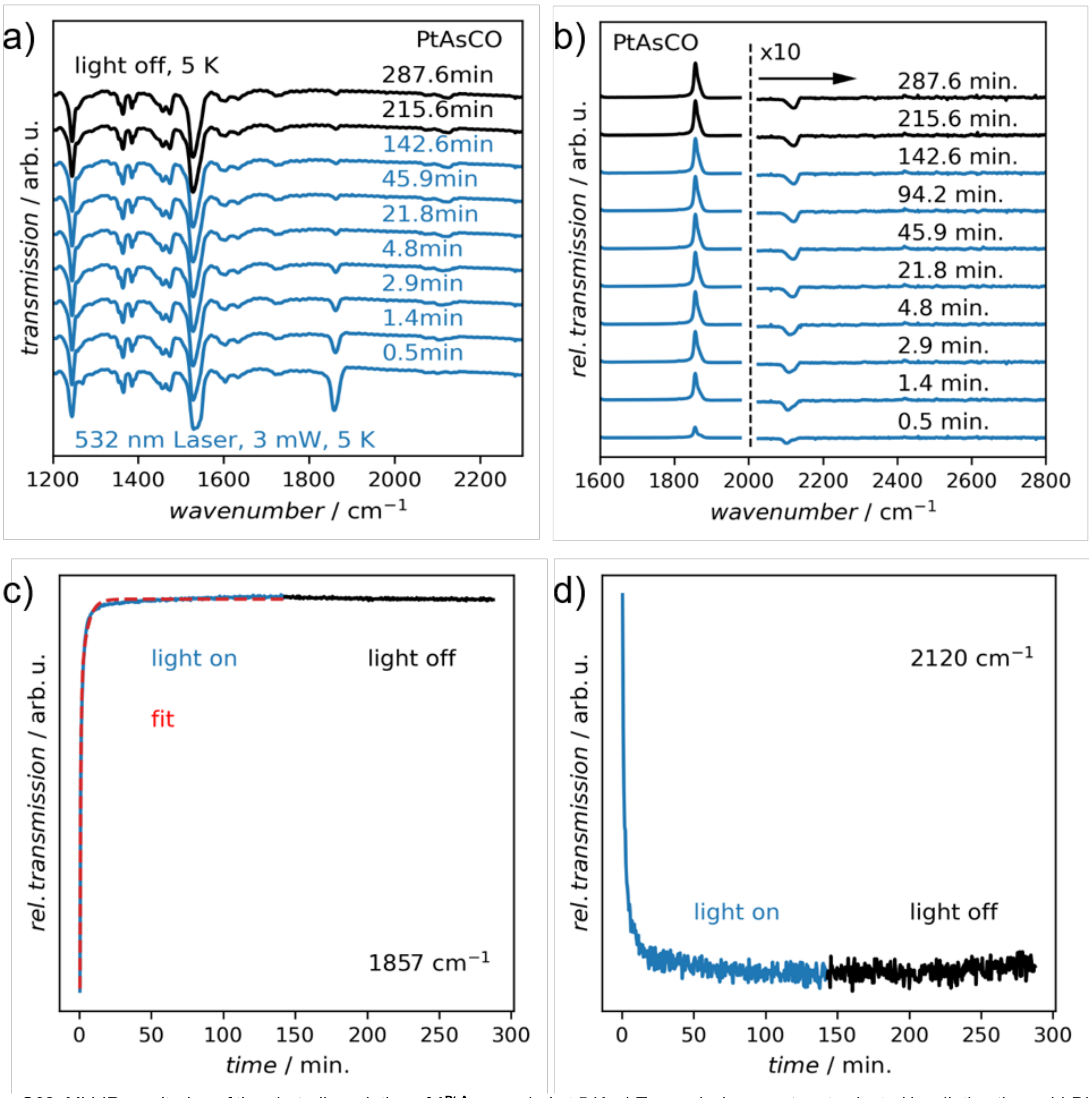

Figure S62: Mid-IR monitoring of the photodissociation of **1**^{Pt,As} recorded at 5 K. a) Transmission spectra at selected irradiation times. b) Division spectra referenced at the spectrum before irradiation. c) Relative transmission at the spectral position of the bound CO (1857 cm^{-1}) against photolysis time (blue) and against time after end of photolysis (black); the dashed red curve shows a fit on the experimental data during irradiation. d) Relative transmission at the spectral position of the free CO (2120 cm^{-1}) against photolysis time (blue) and against time stopping photolysis (black).

THz-EPR measurements

THz-EPR measurements were carried out in the range of 30 to 650 cm^{-1}. Guided by the examination kinetics derived above, the precursor samples were first photolyzed with continuous recording of spectra at zero magnetic field until no further spectral changes were observed (Figures S63a and S64a). The light-generated changes as a function of the illumination time referenced to the spectrum before illumination (t = 0) are depicted in Figure S63a (**1**^{Pd,As}) and S64a (**1**^{Pt,As}) as blue traces. The numerous and complex changes in the FIR spectra can be attributed to phonons that change their energies during the dissociation of CO, which are superimposed with the EPR signals of the triplet photoproducts. For deconvolution of EPR signals, magnetic field dependent spectra (MDS) from 0 to 10 T were recorded after the end of photolysis. (Figures S63b (**1**^{Pd,As}) and S64b (**1**^{Pt,As})).
For **1**^{Pd,As}, the MDS clearly show a signal around 150 – 160 cm^{-1} that changes systematically with the magnetic field with the typical shape of an EPR line of a triplet state with a large axial ZFS tensor. With increasing magnetic field, the absorption band broadens due to splitting of the M_S = ± 1 levels by the field dependent electron Zeeman interaction (Figure S63c and S64c) and division of a spectrum at lower field by another at higher field results in the observed up-down-up pattern. The signal is assigned to the EPR signal of triplet **3**^{Pd,As} and appears in the photolysis time-dependent zero-field spectra as a growing absorption over the illumination time (dotted line in Figure S63a). All other signals in the magnetic-field-division spectra do not show systematic dependence on the magnetic field and can be thus attributed to strong phonon peaks. The magnetic parameters were derived by numerical simulations upon fitting to spin Hamiltonian for a triplet (S =1) ground state:

$$\hat{H} = D\left(\hat{S}_z^2 - \frac{1}{3}S^2\right) + E\left(\hat{S}_x^2 - \hat{S}_y^2\right) + \mu_B B_0 g \hat{S}.$$

D and E: axial and rhombic ZFS parameters; μ_B: Bohr magneton; B_0: the magnetic field; g: g-tensor

The simulated spectra are shown as red traces in Figure S63b and the corresponding best simulation parameters listed in Table S4. The simulation results support the interpretation of the SQUID data, i.e., almost negligible rhombicity (|E| < 0.5 cm^{-1}) and no signs for

g-anisotropy within the spectral resolution. We note in passing that the observed line broadening could also be attributed to moderate g-anisotropy. However, to avoid over interpretation, g was set to 2 with an uncertainty of 0.1 estimated from the signal center of the observed magnetic field dependence and the linewidth.

The THz-EPR spectra of **3**^{Pt,As} (Figure S64) were investigated analogously. However, the measurements did not show magnetic features of similar strength and was further complicated by poorer signal-to-noise ratio. Assignment of the EPR transitions was therefore guided by the results from SQUID magnetometry (D = 143 cm^{-1}) to narrow down the spectral window for simulations. The assigned feature around 150 cm^{-1} shows the characteristic pattern for a triplet state with axial ZFS and magnetic field dependence that could be sufficiently simulated (red traces in Figure S64b, Table S4). The assignment is supported by two independent measurements with different sample concentrations. We note that in the MDS of **3**^{Pt,As} stronger signals around 175 and 234 cm^{-1} were detected close to strong phonons or regions of low intensity in the raw spectrum. However, they could be excluded as magnetic resonance signals, as the field dependence does not show the behavior expected for a triplet state.

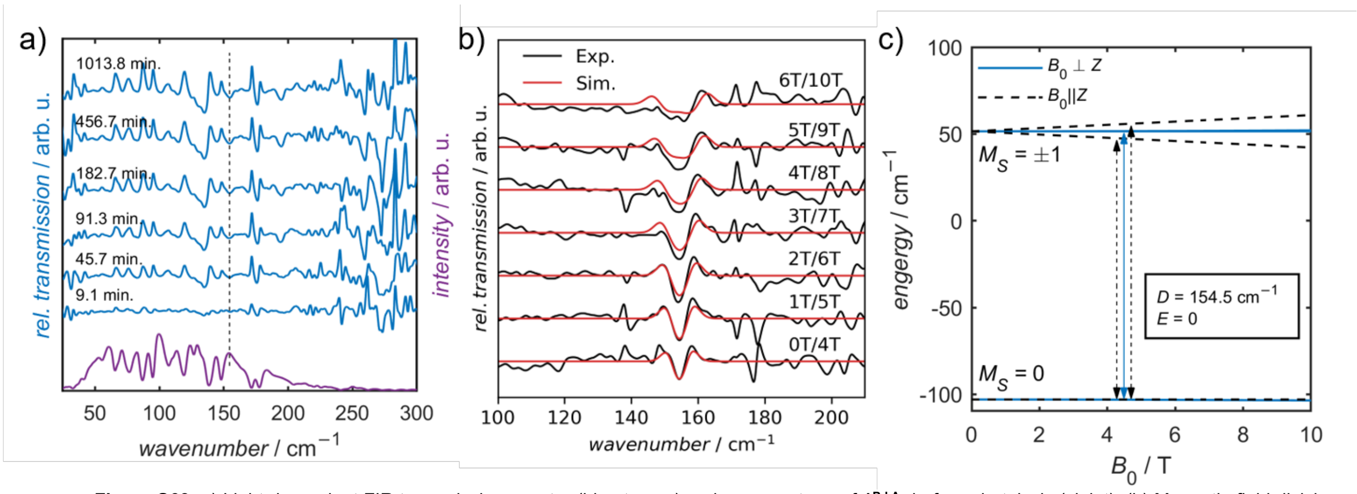

Figure S63: a) Light-dependent FIR transmission spectra (blue traces) and raw spectrum of **1**^{Pd,As} before photolysis (violet). (b) Magnetic-field division THz-EPR spectra of **3**^{Pd,As} (black lines) and numerical simulations (red lines). Simulation parameters: D = 154.5(3) cm^{-1}, $|E|$ = 0 cm^{-1}, g = 2.0(1), linewidth = 5.0(5) cm^{-1}. The magnetic field strengths that were used to calculate the MDS are indicated next to the spectra. c) Calculated magnetic field dependent triplet spin energy levels (Breit-Rabi diagram) using the spin Hamiltonian parameters of **3**^{Pd,As}. Energy levels are plotted for B_0 parallel and perpendicular to the Z-axis of the ZFS tensor. Allowed transitions are indicated as vertical arrows for an arbitrary magnetic field.

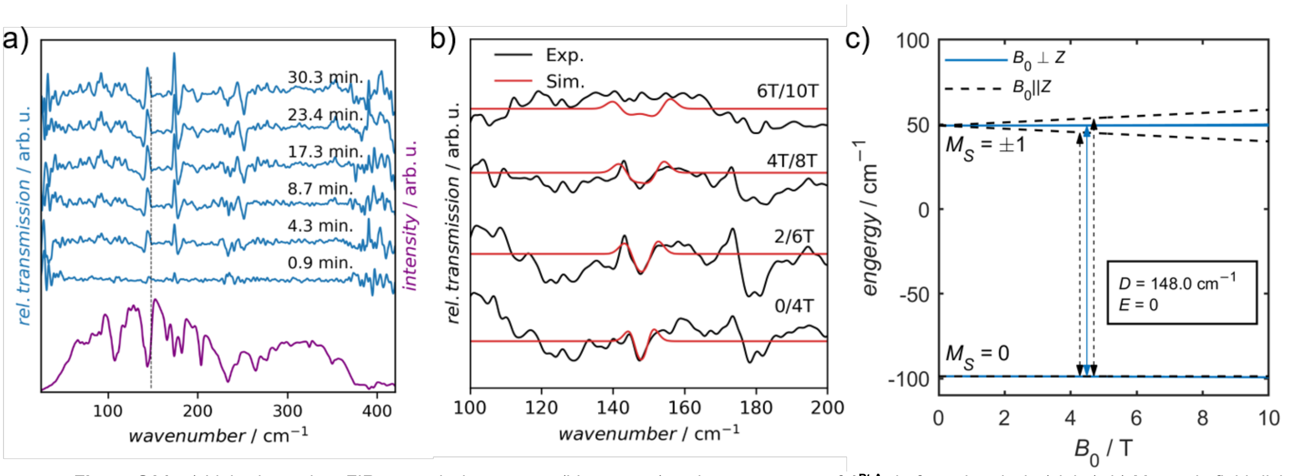

Figure S64: a) Light-dependent FIR transmission spectra (blue traces) and raw spectrum of **1**^{Pt,As} before photolysis (violet). b) Magnetic-field division THz-EPR spectra of **3**^{Pt,As} (black lines) and numerical simulations (red lines). Simulation parameters: D = 148.0(5) cm^{-1}, $|E|$ < 0 cm^{-1}, g = 2.0(4), and a linewidth of 4(1) cm^{-1}.The magnetic field strengths that were used to calculate the MDS are indicated next to the spectra. c) Calculated magnetic field dependent triplet spin energy levels (Breit-Rabi diagram) using the spin Hamiltonian parameters of **3**^{Pt,As}. Energy levels are plotted for B_0 parallel and perpendicular to the Z-axis of the ZFS tensor. Allowed transitions are indicated as vertical arrows for an arbitrary magnetic field.

Table S4: Comparison of the zero-field splitting parameters derived by FD-FT THz-EPR, SQUID magnetometry and quantumchemical computations.

Sample	FD-FT THz-EPR			SQUID		CASSCF (18,11)	
	D **/ cm^{-1}**	***E*** **/ cm^{-1}**	**LW / cm^{-1}**	***D*** **/ cm^{-1}**	***E*** **/ cm^{-1}**	***D*** **/ cm^{-1}**	***E*** **/ cm^{-1}**
**3**^{Pd,As}	154.5(3)	< 0.5	5.0(5)	169	0	95	1
**3**^{Pt,As}	148.0(5)	< 1	4(1)	143	0	172	2

In situ UV-vis studies

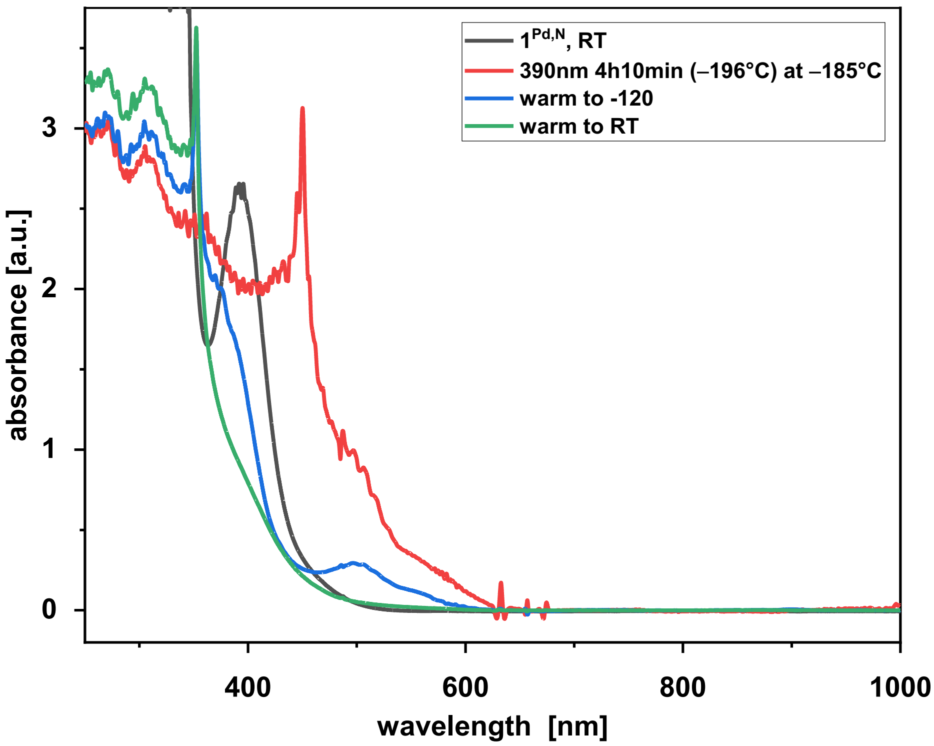

Figure S65: UV-vis spectra of $\mathbf{1}^{Pd,N}$ in 2-methyl-THF at room temperature (black), at –185 °C after photolysis (λ = 370 nm) for 250 min at –196 °C (red), and after warming to –120 °C (blue) and to room temperature (green).

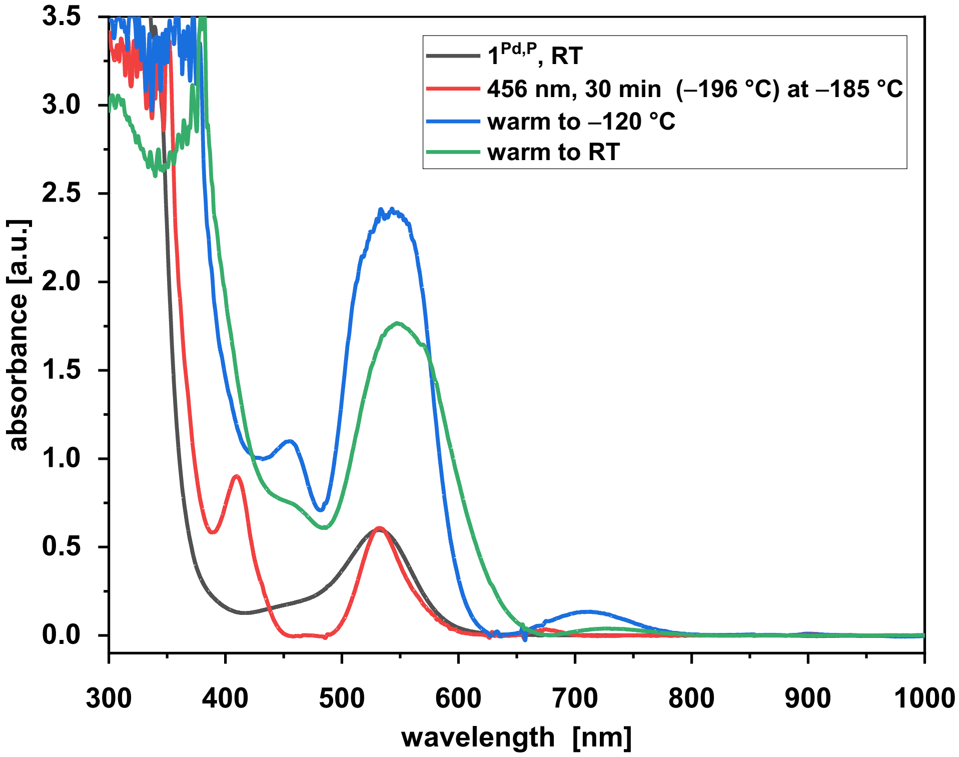

Figure S66: UV-vis spectra of $\mathbf{1}^{Pd,P}$ in 2-methyl-THF at room temperature (black), at –185 °C after photolysis (λ = 456 nm) for 30 min at –196 °C (red), and after warming to –120 °C (blue) and to room temperature (green).

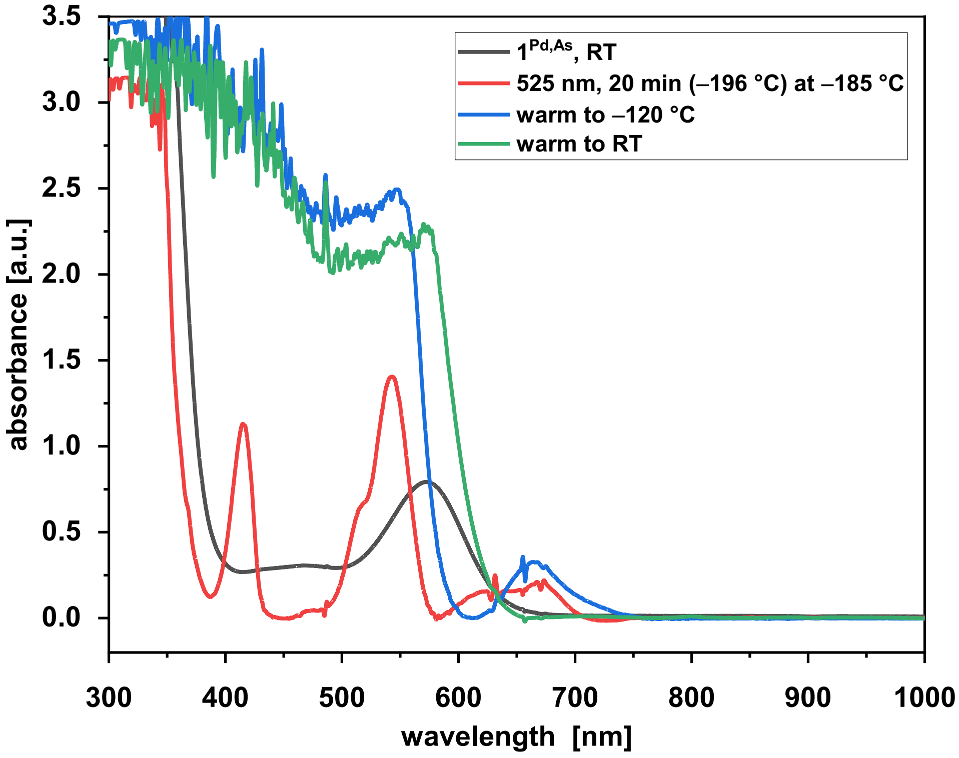

Figure S67: UV-vis spectra of $\mathbf{1}^{Pd,As}$ in 2-methyl-THF at room temperature (black), at –185 °C after photolysis (λ = 525 nm) for 20 min at –196 °C (red), and after warming to –120 °C (blue) and to room temperature (green).

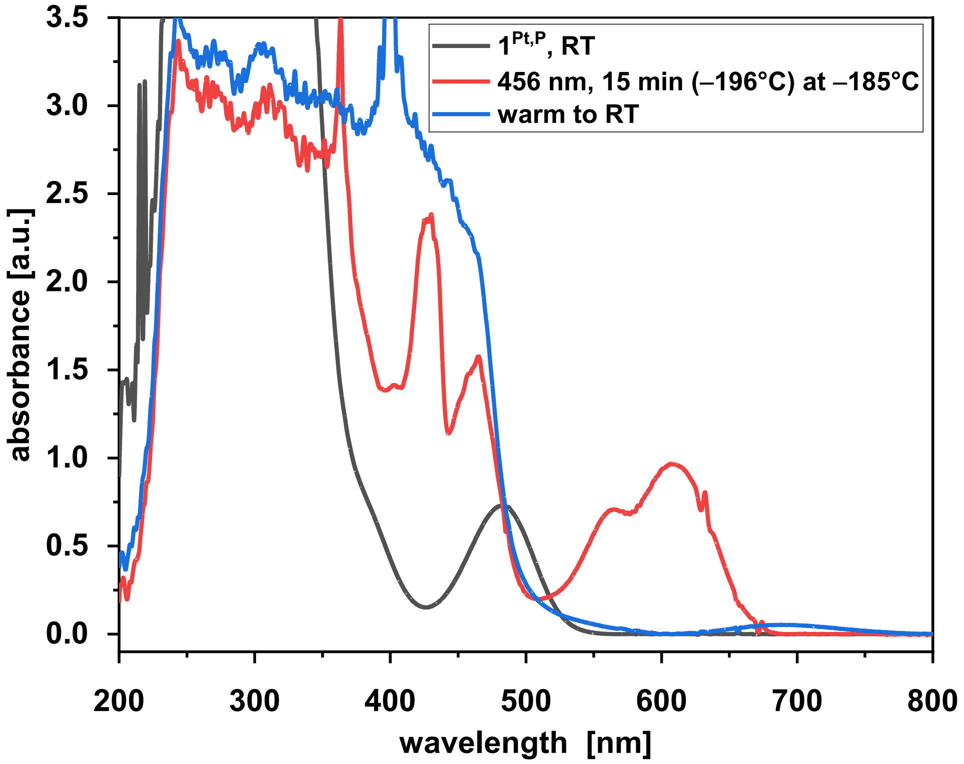

Figure S68: UV-vis spectra of $\mathbf{1}^{Pt,P}$ in 2-methyl-THF at room temperature (black), at –185 °C after photolysis (λ = 456 nm) for 15 min at –196 °C (red), and after warming to room temperature (blue).

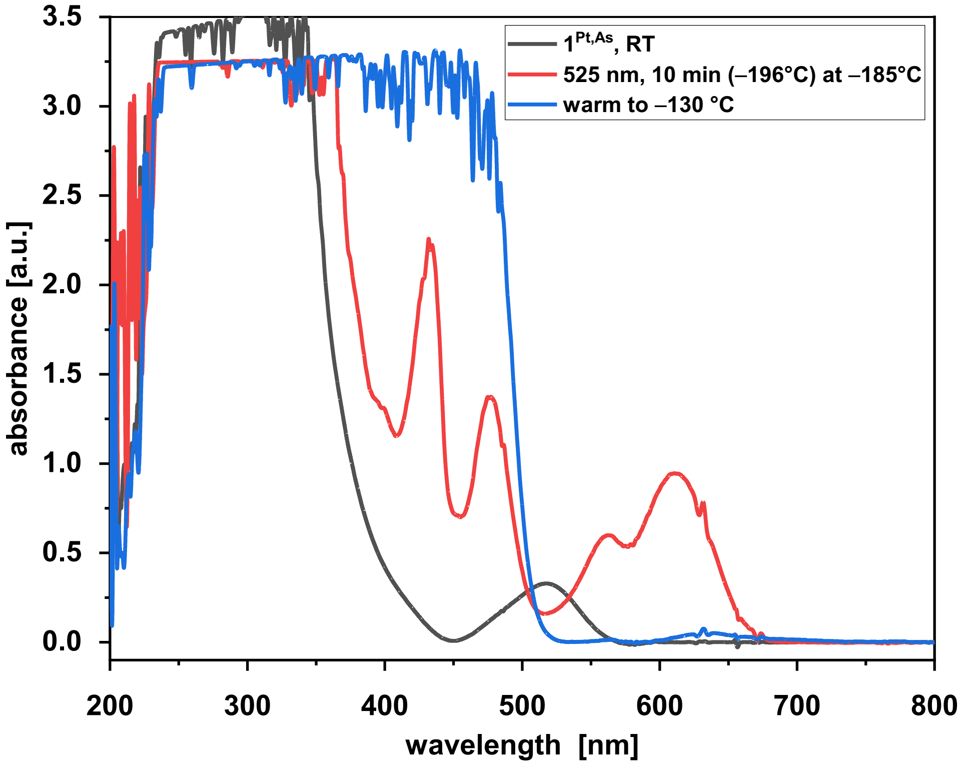

Figure S69: UV-vis spectra of $\mathbf{1^{Pt,As}}$ in 2-methyl-THF at room temperature (black), at –185 °C after photolysis (λ = 525 nm) for 10 min at –196 °C (red), and after warming to –130 °C (blue).

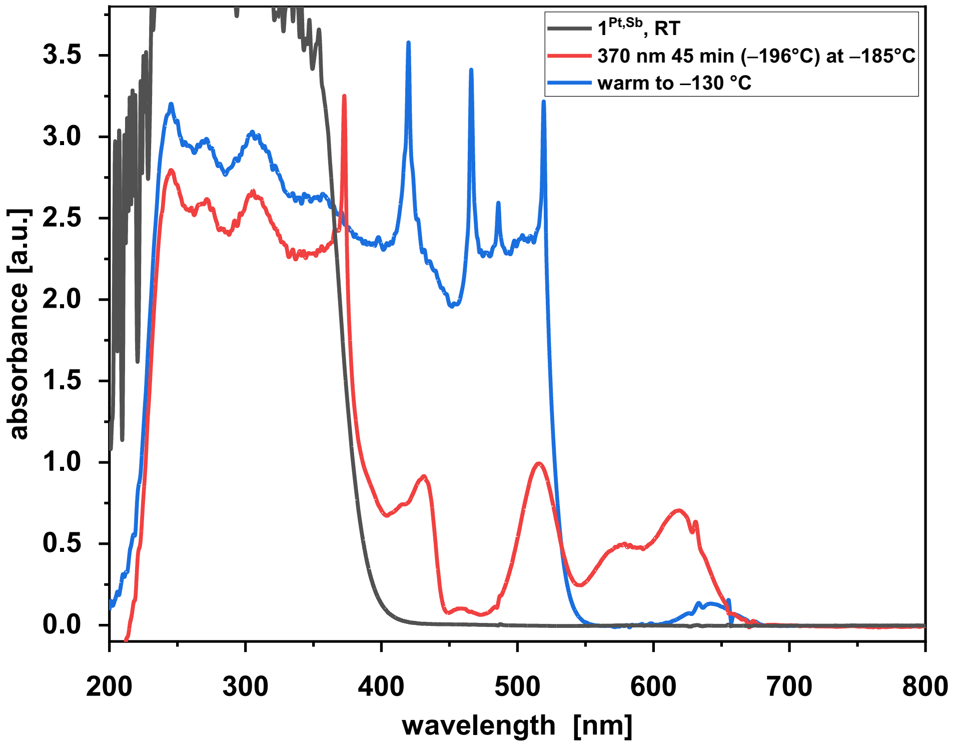

Figure S70: UV-vis spectra of $\mathbf{1^{Pt,Sb}}$ in 2-methyl-THF at room temperature (black), at –185 °C after photolysis (λ = 370 nm) for 45 min at –196 °C (red), and after warming to –130 °C (blue).

Computational Details

Molecular geometry optimizations and Hessian calculations were carried out with the Gaussian 16 program[14] assuming gas phase conditions. The PBE0 hybrid density functional[15,16] and the def2-SVP basis set[17] including a quasi-relativistic pseudopotential[18,19] (ECP60MWB for Pt; ECP28MWB for Pd and Sb) were employed together with D3BJ empirical dispersion corrections with Becke Johnson damping[20–24], abbreviated PBE0-D/def2-SVP. Zero-point vibrational energies and thermostatistic contributions to Gibbs free energies at 298.15 K were obtained within the ideal gas, rigid-rotator, and harmonic oscillator approximations. For improved energies, subsequent single point calculations with the larger def2-TZVPP basis set,[17] abbreviated PBE0-D/def2-TZVPP, were performed; wavefunctions at this level were used for natural bond order analysis (NBO) with the NBO 6.0 program[25]. To investigate the Raman intensities of the Pn–Pn stretching modes in the dipnictenes $\mathbf{2}^{\mathbf{M,Pn}}$, reoptimizations and subsequent frequency calculations were performed based on the PBE0 optimized structures employing the BP86[26,27] functional in combination with the def2-TZVP basis set[17] and density fitting using the *auto* fitting basis set.

Scalar relativistic and spin-orbit eigenstates for all metallopnictinidenes were calculated at the DFT triplet ground-state geometries with the Orca 4.2.1 program[28,29] by means of state-averaged CASSCF/NEVPT2[30–32] calculations followed by a quasi-degenerate perturbation theory treatment (QDPT) via a full spin-orbit mean field approximation (SOMF).[33] CASSCF wavefunctions were optimized within the ZORA approximation[34–37] employing the all-electron scalar-relativistic ZORA-def2-TZVP basis set and the segmented all-electron relativistically recontracted SARC-ZORA-TZVPP basis set[38,39] for palladium, platinum, antimony, and bismuth. The RIJK algorithm[40] was used for fitting of Coulomb and exchange integrals with the automatically generated auxiliary basis set (AutoAux).[41] The active space for all metallopnictinidenes comprises the five metal centered valence d-orbitals, the p_σ and p_π-orbitals of the pnictogen ligand, two metal PNP σ-orbitals and a PNP π-orbital, giving rise to a CAS(18,11) expansion (Figure S82). According to ab initio ligand-field theory arguments based on the formal oxidation state of the metal center, the CASSCF orbitals were optimized averaging over 100 states (5 quintet, 45 triplet, and 50 singlet roots) arising from the d^6 configuration of a palladium(IV) or platinum(IV) center.[42] Without anticipating the results of the computational analysis, this ensures coverage of both the larger $d^6(M^{IV})$ as well as the smaller $d^8(M^{II})$ configuration spaces (the latter counting 25 states – 10 triplets and 15 singlets), as was already demonstrated in previous studies.[1,2,43] Final energies are obtained in NEVPT2 calculations, employing the strongly-contracted NEVPT2 variant. The energies that enter the QDPT treatment via the SOMF operator are thus corrected to second order.

TD-DFT calculations were performed at the DFT ground-state geometries with the Orca 5.0.4 package[44] using the B3LYP-functional[45,46] and D3BJ empirical dispersion correction. In analogy to the CASSCF calculations, the scalar-relativistic ZORA approximation was used together with the all-electron scalar-relativistic ZORA-def2-TZVP basis set and the segmented all-electron relativistically recontracted SARC-ZORA-TZVPP basis set for palladium, platinum, antimony and bismuth. The Coulomb fitting SARC/J basis set was employed as an auxiliary basis set. The conductor-like polarizable continuum model (CPCM)[47] was included to implicitly simulate the solvents that were used in the UV-vis experiments: 2-Methyltetrahydrofuran (dielectric constant $\varepsilon = 6.97$ and refractive index $r = 1.407$) for all metallopnictinidenes $\mathbf{3}^{\mathbf{M,Pn}}$ and the Bi dipnictenes $\mathbf{2}^{\mathbf{M,Bi}}$; tetrahydrofuran ($\varepsilon = 7.25$ and $r = 1.407$) in the case of the remaining dipnictenes $\mathbf{2}^{\mathbf{M,Pn}}$ (Pn ≠ Bi) and all precursors $\mathbf{1}^{\mathbf{M,Pn}}$. In each TD-DFT calculation, the first 100 excited states were calculated. Tight SCF convergence criteria were employed. Simulated spectra were obtained with the *orca_mapspc* utility assuming a homogeneous line broadening of 1500 cm^{-1}.

For further scrutiny, additional TD-DFT calculations were performed for $\mathbf{3}^{\mathbf{Pt,As}}$ using the BLYP GGA-functional[48] (0% HF-exchange), the TPSSh hybrid functional[49] (10% HF-exchange), and the CAM-B3LYP range-separated functional[50] (19% (short range)/65% (long range) HF-exchange). As detailed below, however, B3LYP results match the experimental data best and was thus used for all TD-DFT simulations. In the case of the dipnictenes $\mathbf{2}^{\mathbf{M,Pn}}$, however, difficulties were encountered in predicting the UV-vis spectra using TD-DFT. Firstly, the varying orientations of the [M-PNP] planes in the dipnictenes result in different excitation patterns. In Sb and Bi dipnictenes featuring two coplanar [M-PNP] fragments, the π*(Pn)⟶π*(Pn) and π(PNP)⟶π*(Pn) excitations are symmetry forbidden. Both excitations are allowed for the P and As dipnictenes bearing orthogonal [M-PNP] fragments, thus obscuring direct comparison of the UV-vis spectra of the two structural motifs. In fact, the calculated π*(Pn) ⟶ π* (Pn) excitations (660–710 nm/14100–15100 cm^{-1}) for the P and As dipnictenes are in good agreement with the experimentally determined UV-vis absorption bands in this spectral range. In contrast, the experimental spectra of the Sb and Bi platinum dipnictenes do not exhibit any absorption bands in this spectral range, in line with the corresponding TD-DFT results. This might imply that the As dipnictenes feature orthogonal [M-PNP] fragments in solution, despite bearing coplanar [M-PNP] fragments in the solid state. As a second complication, some B3LYP calculated excitations for dipnictenes $\mathbf{2}^{\mathbf{M,Pn}}$ tend to be blue-shifted in comparison to the experimental absorption bands. For further validation of our approach we chose the experimental UV-vis spectra of aryldipnictenes $\mathbf{2}^{\mathbf{Ar,Pn}}$ (Ar: C_6H_3-2,6-Mes_2; Pn = P, As, Sb, Bi) put forth by Power et al.[51] as a relevant testing ground. As detailed in Table S9 below, the ZORA-B3LYP approach provides excellent agreement with both, the experimental data and earlier TD-DFT results by Vilarrubias.[52] This indicates that the erroneous blue-shift observed is a consequence of a poor description of PNP/Pn_2 and Pn_2/M charge-transfer excitations, whereas excitations within the Pn_2 fragments are far more accurate.

Electronic structures of the precursors $1^{M,Pn}$

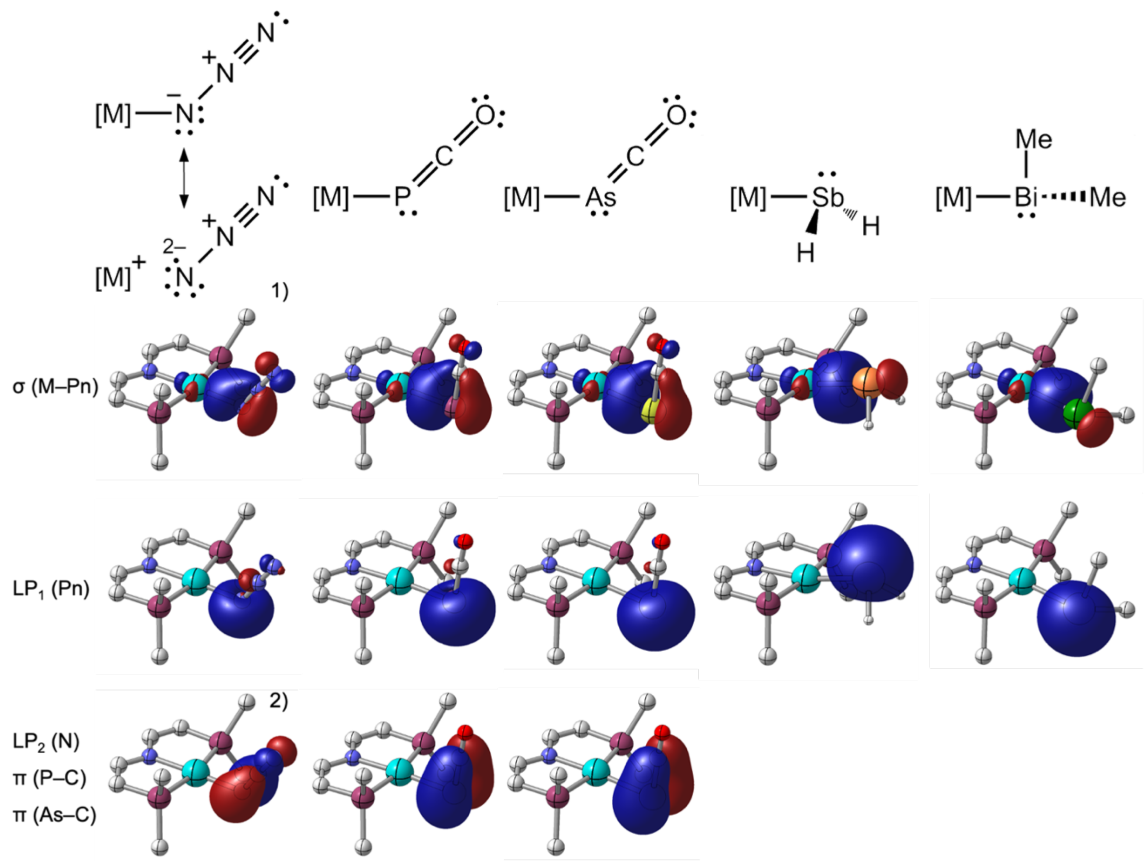

Figure S71: Leading Lewis structures of precursor complexes $\mathbf{1}^{M,Pn}$ resulting from NBO analysis and NLMOs of the M–Pn σ-bonds, the Pn lone pairs and Pn–X π-bonds (shown for M = Pd; results for M = Pt are equivalent; isosurfaces at ±0.05 $a_0^{-3/2}$; methyl groups and hydrogen atoms not shown). 1) In the leading Lewis structure (98.57% Lewis character) of $\mathbf{1}^{Pt,N}$, another N lone pair is present instead of a Pt–N σ-bond, which is present in an alternative representation with marginally less Lewis character (98.56%). However, the respective NLMOs do not differ visually. In contrast, the leading Lewis structure (98.42%) of $\mathbf{1}^{Pt,N}$ features a covalent Pd–N bond while the representation including an N lone pair has a slightly less Lewis character (98.39%). 2) In the case of $\mathbf{1}^{M,N}$, the NLMO shown is characterized as a lone pair localized at the vicinal N, while those shown for $\mathbf{1}^{M,P}$ and $\mathbf{1}^{M,As}$ are characterized as Pn–Pn π-bonds, as indicated in the Lewis structures.

Table S5: M–Pn bond lengths $d_{M\text{–}Pn}$ and NBO analyses of M–Pn σ-bonds, Pn lone pairs and Pn–X π-bonds in precursor complexes $\mathbf{1}^{M,Pn}$: Total Lewis character, charges at M, Pn and X according to natural population analysis (NPA), Wiberg bond indices (WBI), contributions of M and Pn to the NLMOs, and sp^x-hybridization *h* at Pn atoms with p-character. For comments 1) and 2) see the caption of Figure S71.

		$\mathbf{1}^{Pd,N}$	$\mathbf{1}^{Pd,P}$	$\mathbf{1}^{Pd,As}$	$\mathbf{1}^{Pd,Sb}$	$\mathbf{1}^{Pd,Bi}$	$\mathbf{1}^{Pt,N}$	$\mathbf{1}^{Pt,P}$	$\mathbf{1}^{Pt,As}$	$\mathbf{1}^{Pt,Sb}$	$\mathbf{1}^{Pt,Bi}$
$d_{M\text{–}Pn}$ / Å		2.031	2.382	2.487	2.611	2.737	2.043	2.390	2.498	2.632	2.770
Lewis Character / %		98.42	98.56	98.63	98.89	99.04	98.57	98.70	98.78	99.00	99.12
NPA charges											
M		0.43	0.28	0.26	0.17	0.16	0.39	0.23	0.21	0.12	0.09
Pn		–0.54	–0.14	–0.12	0.03	0.54	–0.55	–0.14	–0.13	0.03	0.55
N^b (N_3)		0.17					0.18				
N^c (N_3)		–0.26					–0.25				
C (PnCO)			0.22	0.24				0.24	0.26		
O (PnCO)			–0.52	–0.51				–0.52	–0.51		
H^a (SbH_2)					–0.13					–0.13	
H^b (SbH_2)					–0.13					–0.13	
C^a ($BiMe_2$)						–0.93					–0.98
C^b ($BiMe_2$)						–0.94					–0.99
Pn lone pairs											
LP_1(Pn)	Pn / %	91.71	94.55	95.63	98.71	98.52	90.68	94.20	95.44	98.92	98.73
	h (Pn)	$sp^{0.50}$ (33.20)	$sp^{0.29}$ (22.43)	$sp^{0.21}$ (17.30)	$sp^{0.46}$ (31.30)	$sp^{0.22}$ (17.76)	$sp^{0.53}$ (34.71)	$sp^{0.33}$ (24.58)	$sp^{0.24}$ (19.20)	$sp^{0.51}$ (33.55)	$sp^{0.24}$ (19.62)
LP_2(Pn)	Pn / %	71.45					71.63				
	h (Pn)	p (99.76)					p (99.77)				
M–Pn											
	WBI	0.37	0.43	0.43	0.56	0.56	0.42	0.50	0.50	0.62	0.62
σ(M–Pn)	M / %	20.20	34.04	35.57	43.78	48.61	11.72	35.16	36.78	44.29	48.15
	Pn / %	63.85	51.45	50.53	51.41	46.25	68.93	52.63	51.43	51.79	47.29
	h (Pn)	$sp^{21.19}$ (95.31)	$sp^{15.40}$ (93.18)	$sp^{17.91}$ (94.18)	$sp^{8.62}$ (89.36)	$p^{14.89}$ (93.58)	$sp^{16.58}$ (94.13)	$sp^{10.58}$ (90.70)	$sp^{12.43}$ (92.05)	$sp^{6.95}$ (87.17)	$sp^{11.63}$ (91.95)
Pn–X		N–N 2)	P–C	P–C			N–N 2)	P–C	P–C		
	WBI	1.69	1.84	1.74			1.67	1.82	1.72		
π(Pn–X)	Pn / %	—	61.25	63.02			—	60.87	62.68		
	X / %	—	37.30	35.26			—	37.80	35.71		

Computed UV-vis spectra of Precursors $1^{M,Pn}$

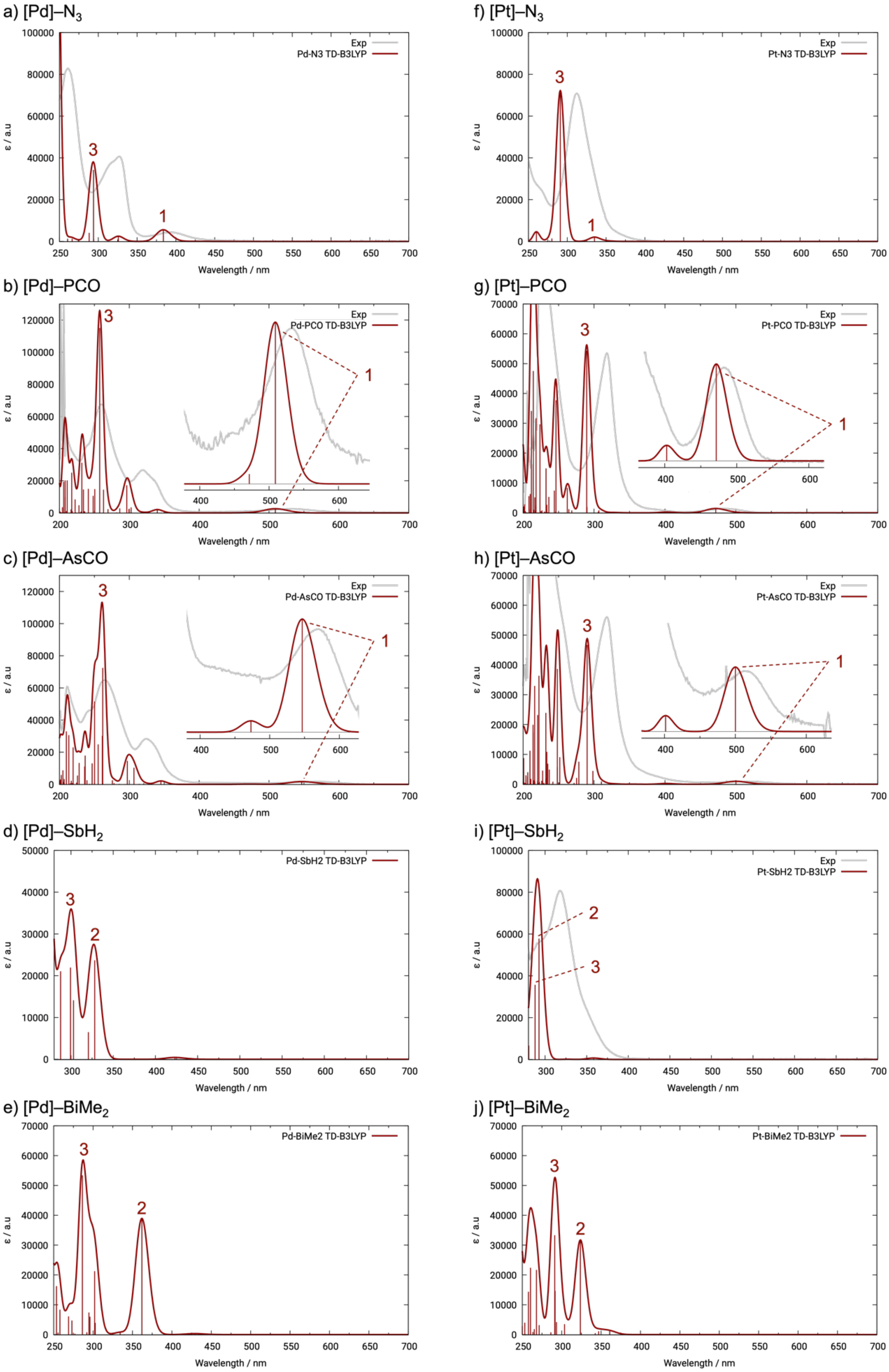

Figure S72: Experimental (gray) and TD-DFT computed (red) UV-vis spectra of precursor complexes $\mathbf{1}^{M,Pn}$. Further details are provided in Figure S73 and Table S6.

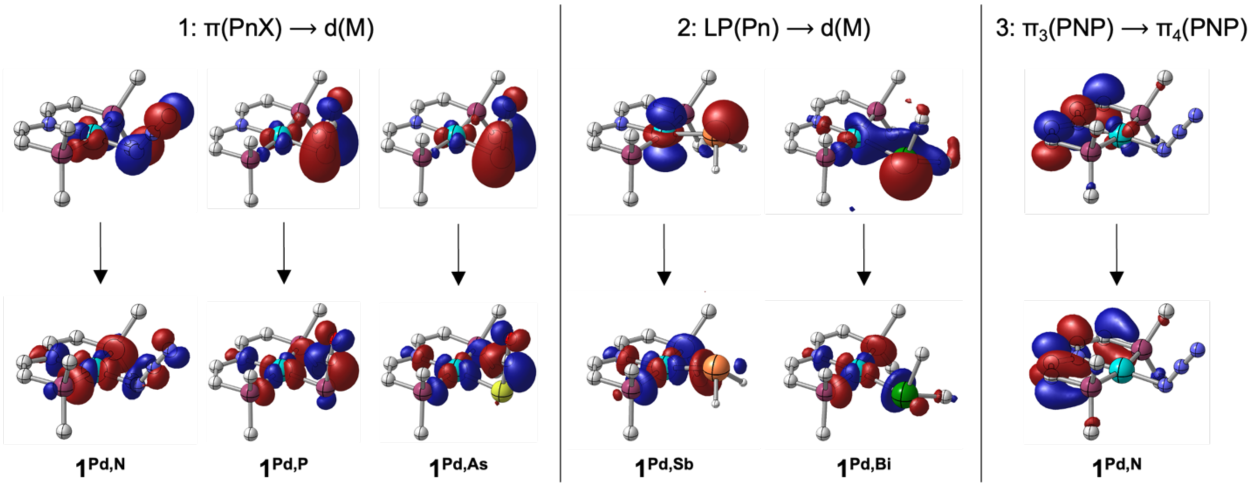

Figure S73: Leading NTOs of selected excitations of precursor complexes **1**$^{\mathbf{M,Pn}}$. Excitation 1 exists only for **1**$^{\mathbf{M,N}}$, **1**$^{\mathbf{M,P}}$ and **1**$^{\mathbf{M,As}}$; excitation 2 exists only for **1**$^{\mathbf{M,Sb}}$ and **1**$^{\mathbf{M,Bi}}$; excitation 3 exists for all precursors (excitations shown for M = Pd; results for M = Pt equivalent; isosurfaces at ±0.05 $a_0^{-3/2}$; methyl groups and hydrogen atoms not shown for clarity).

Table S6: TD-DFT computed absolute energies of selected excitations of precursor complexes **1**$^{\mathbf{M,Pn}}$ in cm^{-1}, oscillator strengths in parentheses.

	Transition	**1**$^{\mathbf{Pd,N}}$		**1**$^{\mathbf{Pd,P}}$		**1**$^{\mathbf{Pd,As}}$		**1**$^{\mathbf{Pd,Sb}}$		**1**$^{\mathbf{Pd,Bi}}$	
1	π (PnX) ⟶ $d_{z^2-y^2}$ (M)	26100	(0.0386)	19600	(0.0180)	18300	(0.0120)	—	—	—	—
2	LP (Pn) ⟶ $d_{z^2-y^2}$ (M)	—	—	—	—	—	—	30500	(0.1638)	27600	(0.2692)
3	π_3 (PNP) ⟶ π_4 (PNP)	34000	(0.2371)	33700	(0.1189)	33700	(0.1013)	33400	(0.1518)	34900	(0.3691)

	Transition	**1**$^{\mathbf{Pt,N}}$		**1**$^{\mathbf{Pt,P}}$		**1**$^{\mathbf{Pt,As}}$		**1**$^{\mathbf{Pt,Sb}}$		**1**$^{\mathbf{Pt,Bi}}$	
1	π (PnX) ⟶ $d_{z^2-y^2}$ (M)	29800	(0.0142)	21200	(0.0099)	20000	(0.0067)	—	—	—	—
2	LP (Pn) ⟶ $d_{z^2-y^2}$ (M)	—	—	—	—	—	—	34700	(0.2468)	30900	(0.2192)
3	π_3 (PNP) ⟶ π_4 (PNP)	34400	(0.4980)	34500	(0.3755)	34500	(0.3229)	34200	(0.3997)	34300	(0.2304)

Electronic structures of dipnictenes $\mathbf{2}^{M,Pn}$

Table S7: Free energy ΔG_D of pnictinidene dimerization in kcal mol^{-1}, calculated Pn–Pn stretching vibrations $\tilde{\nu}_{Pn–Pn}$ in cm^{-1} with Raman intensities and structural parameters of dipnictenes $\mathbf{2}^{M,Pn}$ (M = Pd, Pt; Pn = P, As, Sb, Bi). Bond lengths *d* in Å, bond angles ∡ and dihedral angles *ω* that define the relative orientation of the two [M-PNP] fragments to the Pn_2 fragment in degrees.

		$\mathbf{2}^{Pd,P}$	$\mathbf{2}^{Pd,As}$	$\mathbf{2}^{Pd,Sb}$	$\mathbf{2}^{Pd,Bi}$	$\mathbf{2}^{Pt,P}$	$\mathbf{2}^{Pt,As}$	$\mathbf{2}^{Pt,Sb}$	$\mathbf{2}^{Pt,Bi}$
ΔG_D / kcal mol^{-1}		–60.6	–46.9	–39.8	–35.6	–57.6	–44.7	–38.2	–34.0
$\tilde{\nu}_{Pn–Pn}$ / cm^{-1}		571	318	206	150	561	314	204	149
Raman Int. $\tilde{\nu}_{Pn–Pn}$ / a.u.		163	15	17	11	29	29	12	16
Structural parameters	*d* (Pn^a–Pn^b) / Å	2.027	2.237	2.638	2.789	2.037	2.246	2.644	2.794
	d (M^a–Pn^a) / Å	2.295	2.394	2.624	2.707	2.310	2.413	2.640	2.730
	d (M^b–Pn^b) / Å	2.328	2.432	2.624	2.707	2.330	2.439	2.640	2.730
	∡ (M^a–Pn^a–Pn^b) / °	106	103	108	105	107	104	108	105
	∡ (M^b–Pn^b–Pn^a) / °	116	116	108	105	116	116	108	105
	ω (Pn^a–M^a–Pn^a–Pn^b) / °	91	92	90	90	91	92	90	90
	ω (Pn^b–M^b–Pn^b–Pn^a) / °	5	0	90	90	4	0	90	90

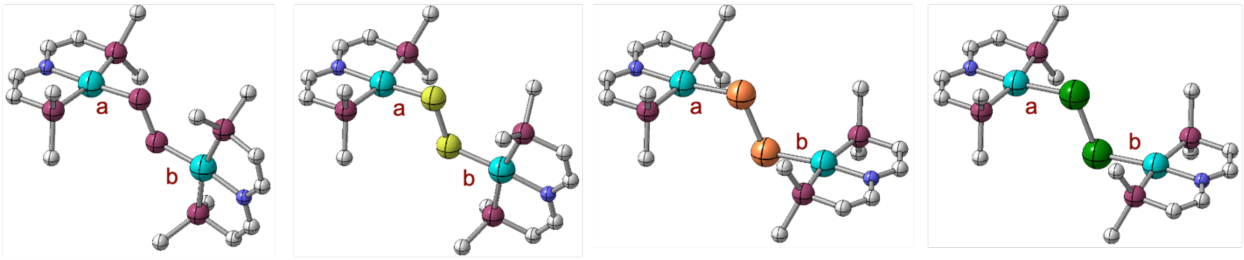

$\mathbf{2}^{Pd,P}$ $\mathbf{2}^{Pd,As}$ $\mathbf{2}^{Pd,Sb}$ $\mathbf{2}^{Pd,Bi}$

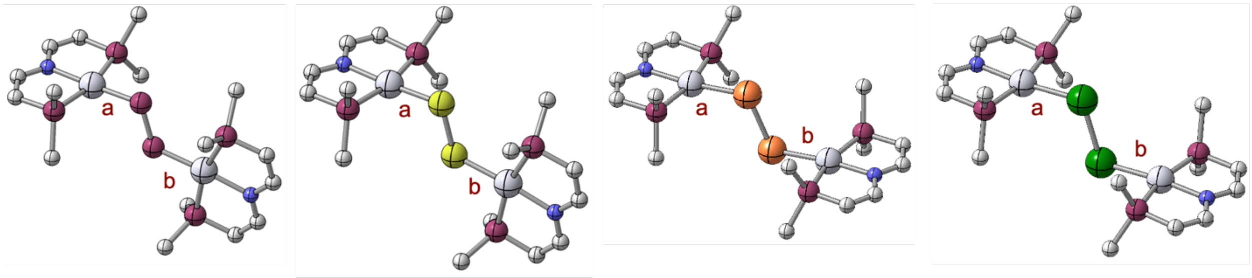

$\mathbf{2}^{Pt,P}$ $\mathbf{2}^{Pt,As}$ $\mathbf{2}^{Pt,Sb}$ $\mathbf{2}^{Pt,Bi}$

Figure S74: Structures of dipnictenes $\mathbf{2}^{M,Pn}$ (M = Pd, Pt; Pn = P, As, Sb, Bi; a and b designate the individual [Pn-M-PNP] fragments referred to in Table S7; methyl groups and hydrogen atoms not shown). Two distinct structural motifs were found: For P and As complexes, calculated minimum structures feature two orthogonal [M-PNP] fragments. In case of Sb and Bi, the [M-PNP] fragments are coplanar. For P, Sb and Bi, this is in agreement with the experimentally determined crystal structures. However, the crystal structures of the As complexes feature two coplanar [M-PNP] fragments, in contrast to an orthogonal alignment determined theoretically.

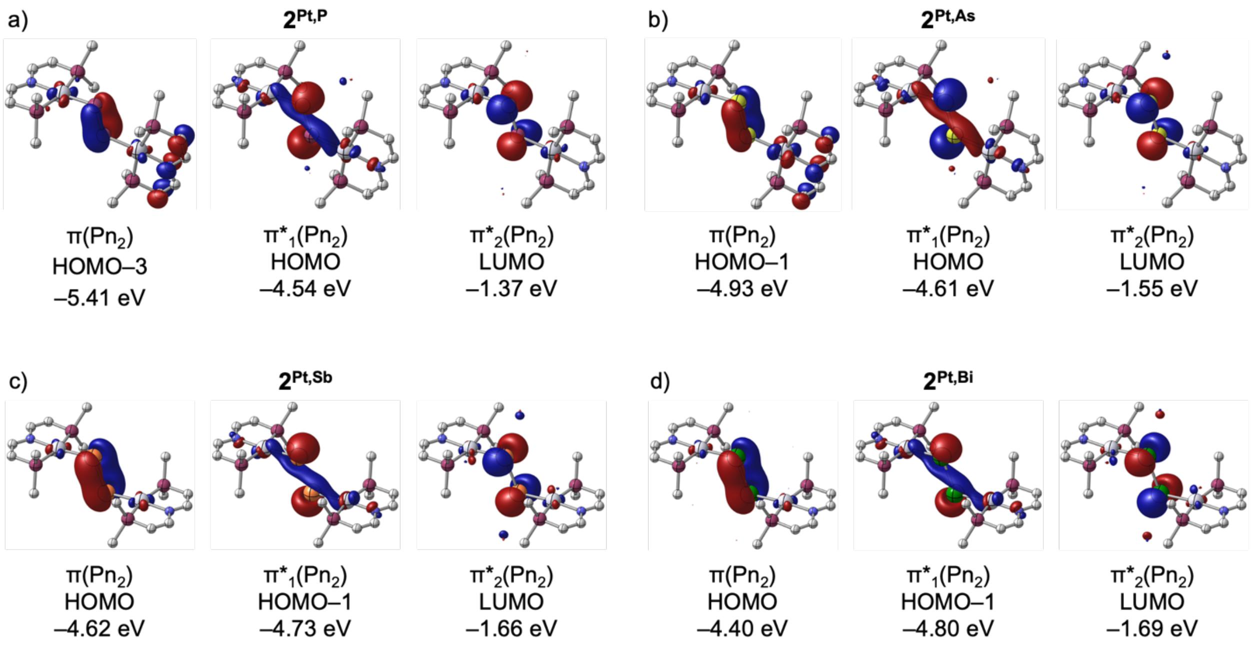

e)

	$\mathbf{2}^{Pd,P}$	$\mathbf{2}^{Pd,As}$	$\mathbf{2}^{Pd,Sb}$	$\mathbf{2}^{Pd,Bi}$
$\pi(Pn_2)$	–5.57 (HOMO–3)	–5.34 (HOMO–3)	–4.79 (HOMO–1)	–4.56 (HOMO)
$\pi^*_1(Pn_2)$	–4.56 (HOMO)	–4.63 (HOMO)	–4.78 (HOMO)	–4.85 (HOMO–1)
$\pi^*_2(Pn_2)$	–1.51 (LUMO)	–1.69 (LUMO)	–1.82 (LUMO)	–1.84 (LUMO)

Figure S75: Selected molecular orbitals of platinum dipnictenes a) $\mathbf{2}^{Pt,P}$, b) $\mathbf{2}^{Pt,As}$, c) $\mathbf{2}^{Pt,Sb}$ and d) $\mathbf{2}^{Pt,Bi}$ (orbital energies in eV; isosurfaces at ±0.05 $a_0^{-3/2}$; methyl groups and hydrogen atoms not shown). e) Selected orbital energies in palladium dipnictenes $\mathbf{2}^{Pd,P}$ (Pn= P, As, Sb, Bi) in eV.

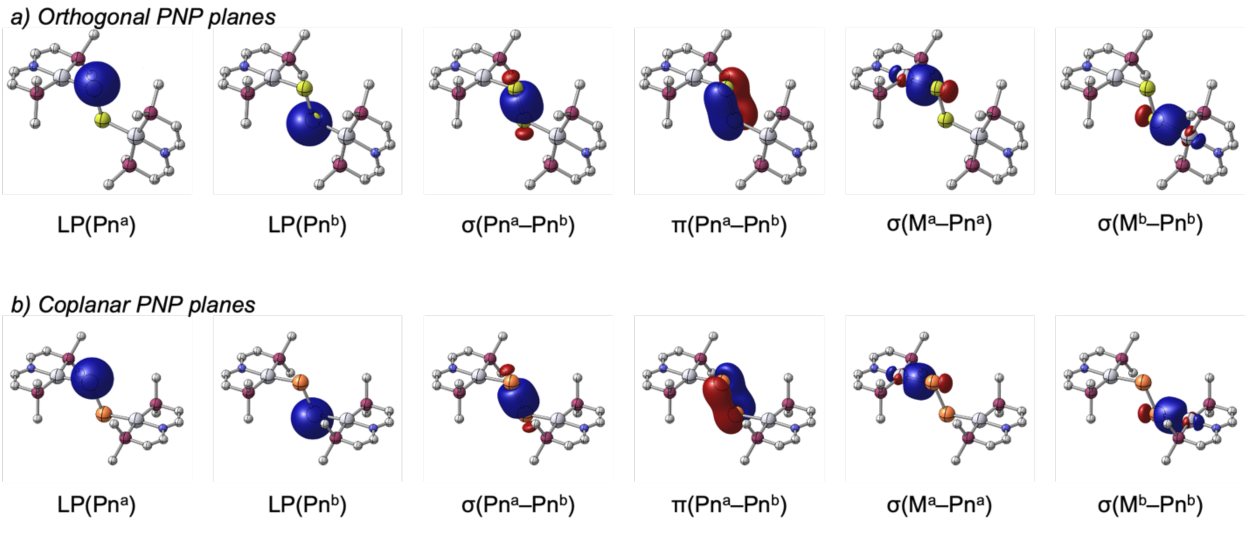

Figure S76: NLMOs of Pn lone pairs (LP), Pn–Pn σ- and π-bonds, and M–Pn bonds in the two distinct structural motifs of dipnictenes $\mathbf{2}^{M,Pn}$: a) $\mathbf{2}^{Pt,As}$ featuring orthogonal [M-PNP] fragments, b) $\mathbf{2}^{Pt,Sb}$ featuring coplanar [M-PNP] fragments (NLMOs of other dipnictenes $\mathbf{2}^{M,Pn}$ equivalent; isosurfaces at ±0.05 $a_0^{-3/2}$; methyl groups and hydrogen atoms not shown).

Table S8: NBO analyses of Pn lone pairs, Pn–Pn σ- and π-bonds, and Pn–M σ-bonds in dipnictenes $\mathbf{2}^{M,Pn}$ (M = Pd, Pt; Pn = N, P, As, Sb, Bi): Total Lewis character, NPA charges of M and Pn, Wiberg bond indices (WBI), contributions of M and Pn to the NLMOs, and sp^x-hybridization *h* at Pn with p-character.

		$\mathbf{2}^{Pd,P}$	$\mathbf{2}^{Pd,As}$	$\mathbf{2}^{Pd,Sb}$	$\mathbf{2}^{Pd,Bi}$	$\mathbf{2}^{Pt,P}$	$\mathbf{2}^{Pt,As}$	$\mathbf{2}^{Pt,Sb}$	$\mathbf{2}^{Pt,Bi}$
Lewis Character / %		98.69	98.79	98.84	98.96	98.84	98.92	98.96	99.06
NPA charges	Pn^a	–0.23	–0.20	–0.22	–0.19	–0.25	–0.22	–0.22	–0.18
	Pn^b	–0.34	–0.33	–0.22	–0.19	–0.36	–0.34	–0.22	–0.18
	M^a	0.22	0.21	0.19	0.18	0.17	0.16	0.12	0.12
	M^b	0.23	0.22	0.19	0.18	0.18	0.17	0.12	0.12
Lone pairs (Pn)									
LP(Pn^a)	Pn^a / %	97.16	97.55	98.12	98.55	97.37	97.77	98.46	98.86
	h (Pn^a)	$sp^{0.46}$ (31.42)	$sp^{0.33}$ (24.70)	$sp^{0.25}$ (19.78)	$sp^{0.15}$ (13.11)	$sp^{0.53}$ (34.55)	$sp^{0.38}$ (27.32)	$sp^{0.28}$ (21.71)	$sp^{0.17}$ (14.47)
LP(Pn^b)	Pn^b / %	96.96	97.58	98.12	98.55	97.51	98.05	98.46	98.86
	h (Pn^b)	$sp^{0.44}$ (30.54)	$sp^{0.31}$ (23.80)	$sp^{0.25}$ (19.78)	$sp^{0.15}$ (13.11)	$sp^{0.51}$ (33.82)	$sp^{0.36}$ (26.55)	$sp^{0.28}$ (21.71)	$sp^{0.17}$ (14.47)
Pn^a–Pn^b									
	WBI	1.89	1.84	1.82	1.81	1.83	1.79	1.79	1.79
σ(Pn^a–Pn^b)	Pn^a / %	49.39	50.04	49.35	49.35	49.48	50.02	49.25	49.23
	h (Pn^a)	$sp^{4.11}$ (79.62)	$sp^{5.12}$ (83.25)	$sp^{6.82}$ (86.83)	$sp^{10.94}$ (91.40)	$sp^{3.99}$ (80.41)	$sp^{5.67}$ (83.45)	$sp^{6.86}$ (86.89)	$sp^{11.03}$ (91.46)
	Pn^b / %	49.47	48.65	49.35	49.35	49.17	48.50	49.25	49.23
	h (Pn^b)	$sp^{4.43}$ (80.71)	$sp^{5.55}$ (84.27)	$sp^{6.82}$ (86.83)	$sp^{10.94}$ (91.40)	$sp^{4.34}$ (79.17)	$sp^{5.67}$ (84.55)	$sp^{6.86}$ (86.89)	$sp^{11.03}$ (91.46)
π(Pn^a–Pn^b)	Pn^a / %	46.88	45.66	48.67	48.44	46.77	45.65	48.63	48.42
	h (Pn^a)	p (96.19)	p (99.16)	p (99.53)	p (99.68)	p (96.78)	p (99.01)	p (99.53)	p (99.68)
	Pn^b / %	51.86	52.66	48.67	48.44	51.71	52.42	48.63	48.42
	h (Pn^b)	p (96.53)	p (99.24)	p (99.53)	p (99.68)	p (97.06)	p (99.09)	p (99.53)	p (99.68)
M–Pn									
σ(M^a–Pn^a)	WBI	0.54	0.54	0.53	0.53	0.63	0.62	0.60	0.60
	M^a/ %	41.36	42.50	43.51	45.07	40.74	42.62	44.66	45.88
	Pn^a / %	52.55	51.70	50.22	48.49	54.91	52.99	450.38	48.93
	h (Pn^a)	$sp^{7.90}$ (88.00)	$sp^{9.43}$ (89.93)	$sp^{10.90}$ (91.16)	$sp^{17.04}$ (94.17)	$sp^{5.62}$ (84.28)	$sp^{7.10}$ (87.22)	$sp^{8.62}$ (89.14)	$sp^{13.39}$ (92.75)
σ(M^b–Pn^b)	WBI	0.54	0.54	0.53	0.53	0.63	0.62	0.60	0.60
	M^b / %	39.95	41.06	43.51	45.07	33.13	41.69	44.66	45.88
	Pn^b / %	54.14	53.37	50.23	48.49	62.49	54.18	50.38	48.93
	h (Pn^b)	$sp^{7.60}$ (87.76)	$sp^{8.97}$ (89.59)	$sp^{10.90}$ (91.16)	$sp^{17.04}$ (94.17)	$sp^{1.86}$ (64.91)	$sp^{6.73}$ (86.69)	$sp^{8.62}$ (89.14)	$sp^{13.39}$ (92.75)

Computed UV-vis spectra of Dipnictenes $2^{M,Pn}$

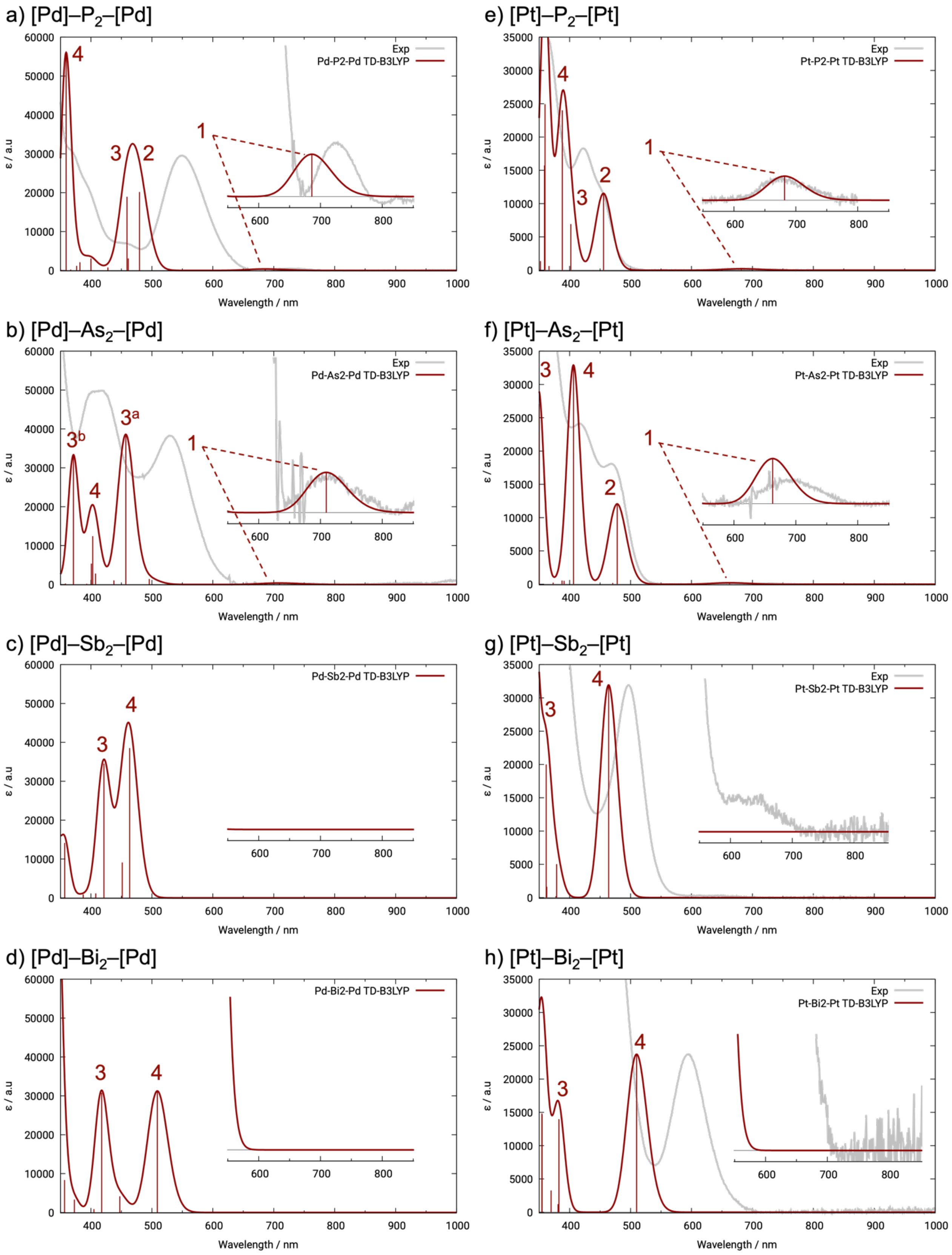

Figure S77: Experimental (gray) and TD-DFT computed (red) UV-vis spectra of dipnictenes $2^{M,Pn}$ (M = Pd, Pt; Pn = P, As, Sb, Bi). Further details are provided in Figure S78 and Table S9.

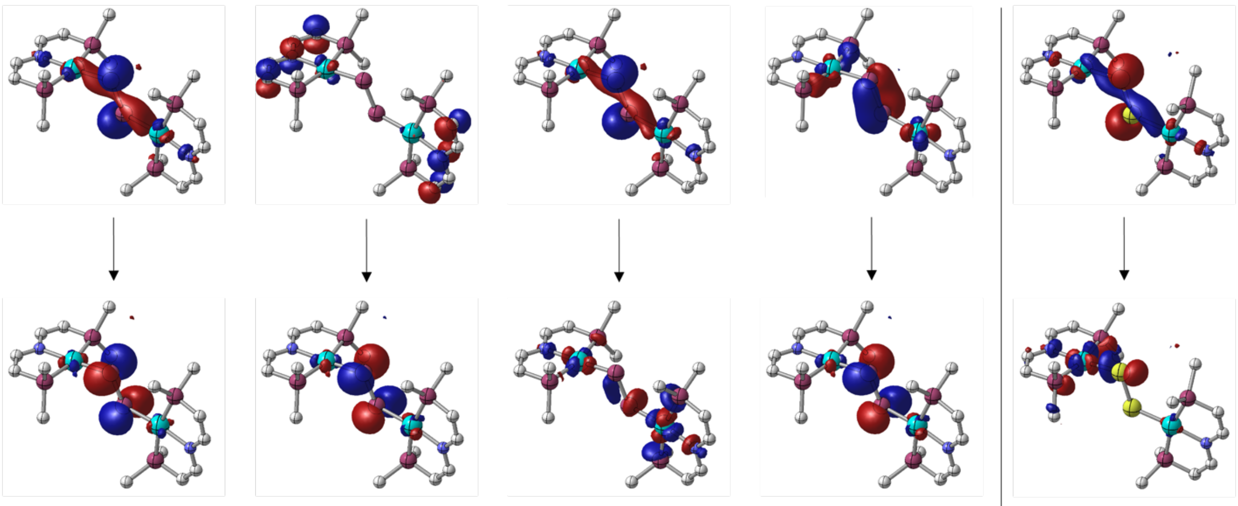

1	2	3	4	3[b]
$\pi^*(Pn_2) \longrightarrow \pi^*(Pn_2)$	$\pi(PNP) \longrightarrow \pi^*(Pn_2)$	$\pi^*(Pn_2) \longrightarrow d(M_2)$	$\pi(Pn_2) \longrightarrow \pi(Pn_2)$	$\pi^*(Pn_2) \longrightarrow d(M_2)$

Figure S78: Leading NTOs of selected excitations 1–4 of dipnictene $\mathbf{2}^{Pd,P}$ (excitations in other dipnictenes $\mathbf{2}^{M,Pn}$ equivalent; isosurfaces at ±0.05 $a_0^{-3/2}$; methyl groups and hydrogen atoms not shown). In the case of $\mathbf{2}^{Pd,As}$ an additional excitation 3[b] is identified with similar character to excitation 3.

Table S9: TD-DFT computed energies of selected excitations of dipnictenes $\mathbf{2}^{M,Pn}$ (M = Pd, Pt; Pn = P, As, Sb, Bi) in cm^{-1}, oscillator strengths in parentheses. In the case of Sb and Bi complexes (coplanar [M-PNP] fragments), transitions 1 and 2 are symmetry forbidden.

Transition		$\mathbf{2}^{Pd,P}$		$\mathbf{2}^{Pd,As}$		$\mathbf{2}^{Pd,Sb}$		$\mathbf{2}^{Pd,Bi}$	
1	$\pi^*(Pn_2) \longrightarrow \pi^*(Pn_2)$	14600	(0.0026)	14100	(0.0025)	15300	*forbidden*	15500	*forbidden*
2	$\pi(PNP) \longrightarrow \pi^*(Pn_2)$	21700	(0.0211)	—	—	19100	*forbidden*	18500	*forbidden*
3	$\pi^*(Pn_2) \longrightarrow d(M_2)$	20800	(0.1390)	a) 21900<br>b) 26900	(0.2643)<br>(0.2292)	23700	(0.2395)	23900	(0.2130)
4	$\pi(Pn_2) \longrightarrow \pi^*(Pn_2)$	27800	(0.3843)	24800	(0.0860)	21600	(0.2663)	19600	(0.2161)

Transition		$\mathbf{2}^{Pt,P}$		$\mathbf{2}^{Pt,As}$		$\mathbf{2}^{Pt,Sb}$		$\mathbf{2}^{Pt,Bi}$	
1	$\pi^*(Pn_2) \longrightarrow \pi^*(Pn_2)$	14700	(0.0015)	15100	(0.0015)	16200	*forbidden*	16400	*forbidden*
2	$\pi(PNP) \longrightarrow \pi^*(Pn_2)$	21900	(0.0796)	20900	(0.0822)	20200	*forbidden*	19700	*forbidden*
3	$\pi^*(Pn_2) \longrightarrow d(M_2)$	24900	(0.0475)	28600	(0.1871)	27600	(0.1377)	26200	(0.0966)
4	$\pi(Pn_2) \longrightarrow \pi^*(Pn_2)$	25800	(0.1662)	24600	(0.2259)	21600	(0.2208)	19600	(0.1640)

Computed UV-vis spectra of Diaryldipnictenes $2^{Ar,Pn}$ (Ar: C_6H_3-2,6-Mes_2; Pn: P, As, Sb, Bi)

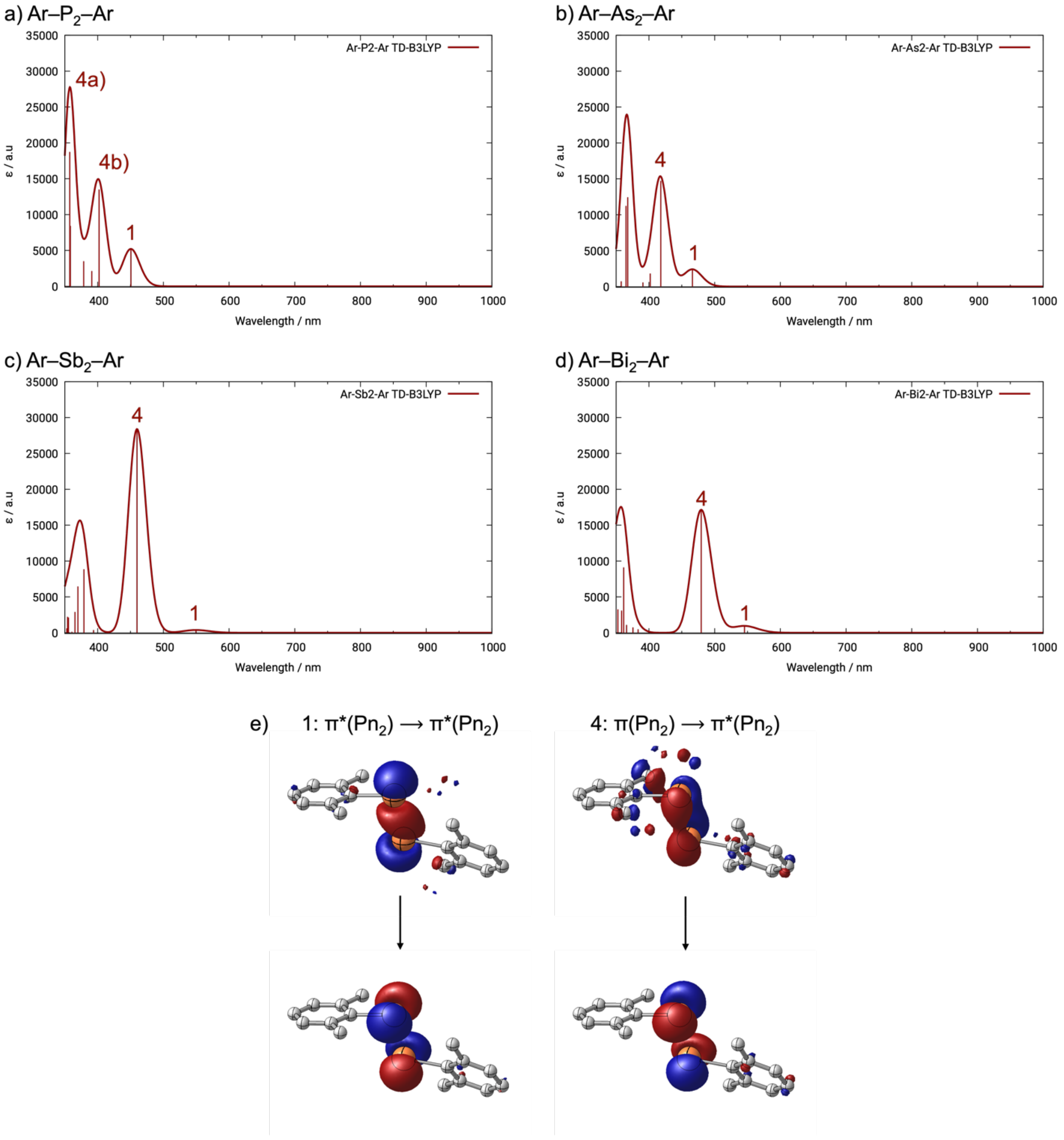

Figure S79: a) – d) TD-DFT computed UV-vis spectra of Aryldipnictenes $\mathbf{2}^{Ar,Pn}$ (Ar: C_6H_3-2,6-Mes_2; Pn = P, As, Sb, Bi). e) Leading NTOs of excitations 1 and 4 of $\mathbf{2}^{Ar,Sb}$ (numbering of the excitations follows the designation of the excitations of the dimers $\mathbf{2}^{M,Pn}$, Figure S78; excitations in other aryldipnictenes $\mathbf{2}^{Ar,Pn}$ equivalent; isosurfaces at ±0.05 $a_0^{-3/2}$; methyl groups and hydrogen atoms not shown). Excitation energies are shown in Table S10.

Table S10: TD-DFT computed energies of selected excitations of organic aryldipnictenes $\mathbf{2}^{Ar,Pn}$ (Ar: C_6H_3-2,6-Mes_2; Pn = P, As, Sb, Bi) in cm^{-1}, oscillator strengths in parentheses. For comparison, experimental values by Power et al.[51] and computed values by Vilarrubias[52] are given in gray.

Transition		Ar–P₂–Ar		Ar–As₂–Ar		Ar–Sb₂–Ar		Ar–Bi₂–Ar	
1	$n^+ (Pn_2) \longrightarrow \pi^* (Pn_2)$	22200	(0.0360)	21500	(0.0166)	18200	(0.0027)	18300	(0.0066)
	Experiment	22000		21600		—		—	
	Computed	21200	(0.0029)	20400	(0.0000)	18700	(0.0000)	18200	(0.0029)
4	$\pi (Pn_2) \longrightarrow \pi^* (Pn_2)$	a) 24900<br>b) 27900	(0.0934)<br>(0.1295)	23900	(0.1017)	21800	(0.1963)	20800	(0.1185)
	Experiment	27000		25000		22200		19800	
	Computed	26700	(0.0436)	25200	(0.0638)	21500	(0.0715)	19600	(0.0576)

Electronic Structure of Dipnictenyl radical cations $[2^{M,Pn}]^+$

Table S11: Ionization energies *IE* of dipnictenes $2^{M,Pn}$ (M = Pd, Pt; Pn = P, As, Sb, Bi) in eV, calculated Pn–Pn stretching vibrations $\tilde{\nu}_{Pn-Pn}$ in cm^{-1} with Raman intensities, NPA spin populations at M and Pn, and structural parameters of $[2^{M,Pn}]^+$. Bond lengths d_{M-Pn} in Å, angles ∡ and dihedrals *ω* that define the relative orientation of the two [M-PNP] fragments to the Pn_2 fragment in degrees.

		$[2^{Pd,P}]^+$	$[2^{Pd,As}]^+$	$[2^{Pd,Sb}]^+$	$[2^{Pd,Bi}]^+$	$[2^{Pt,P}]^+$	$[2^{Pt,As}]^+$	$[2^{Pt,Sb}]^+$	$[2^{Pt,Bi}]^+$
IE / eV		4.9	5.1	5.3	5.4	4.9	5.1	5.2	5.3
$\tilde{\nu}_{Pn-Pn}$ / cm^{-1}		614	335	199/204	118	598	322/332	197/206	97/113
Raman Int. $\tilde{\nu}_{Pn-Pn}$ / a.u.		522	1006	108/19	489	697	423/155	128/59	471/307
NPA spin populations	M^a / %	0.04	0.04	0.03	0.02	0.04	0.03	0.02	0.02
	M^b / %	0.04	0.04	0.04	0.02	0.04	0.05	0.04	0.02
	Pn^a / %	0.25	0.18	0.02	0.16	0.28	0.21	0.10	0.22
	Pn^b / %	0.51	0.60	0.77	0.67	0.52	0.56	0.72	0.63
Structural parameters	*d* (Pn^a–Pn^b) / Å	1.977	2.190	2.602	2.826	1.988	2.203	2.624	2.853
	d (M^a–Pn^a) / Å	2.264	2.375	2.562	2.633	2.269	2.384	2.575	2.654
	d (M^b–Pn^b) / Å	2.277	2.383	2.572	2.677	2.272	2.382	2.581	2.693
	∡ (M^a–Pn^a–Pn^b) / °	118	111	104	106	118	112	105	105
	∡ (M^b–Pn^b–Pn^a) / °	133	135	138	123	132	134	134	122
	ω (Pn^a–M^a–Pn^a–Pn^b) / °	88	91	86	77	87	90	83	79
	ω (Pn^b–M^b–Pn^b–Pn^a) / °	9	1	10	36	10	3	18	35

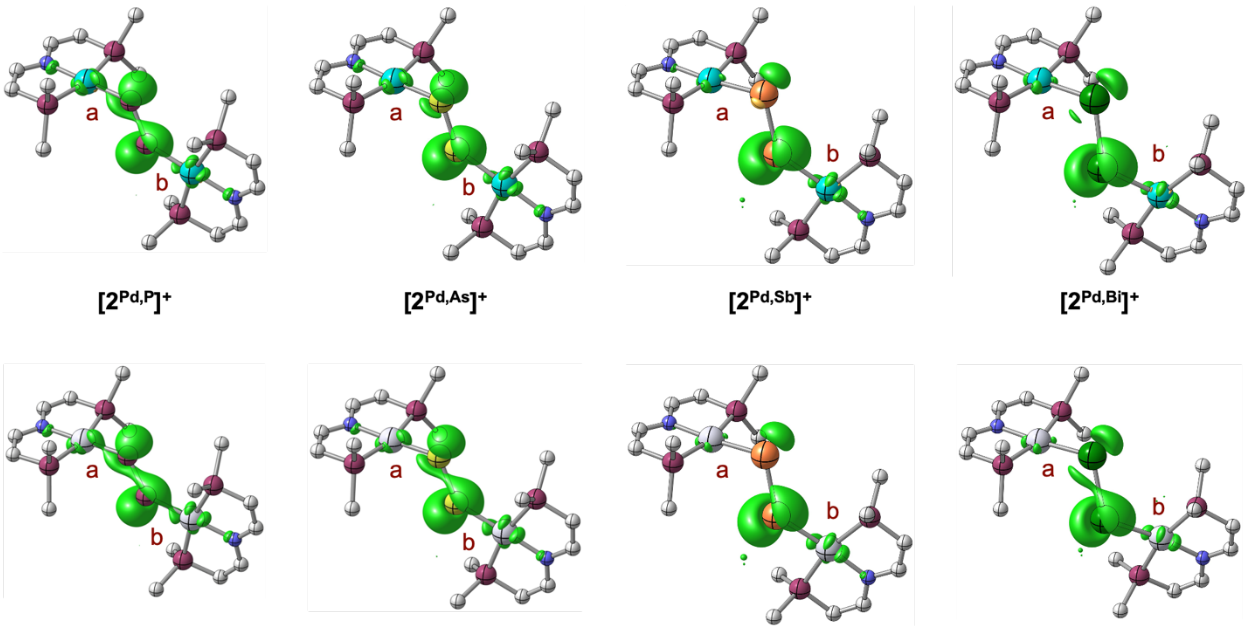

Figure S80: Spin densities of dipnictenyl radical cations $[2^{M,Pt}]^+$ (M = Pd, Pt; Pn = P, As, Sb, Bi; a and b designate the individual [Pn-M-PNP] fragments referred to in Table S11; methyl groups and hydrogen atoms not shown). Respective NPA spin populations of M and Pn atoms are given in Table S11.

Electronic Structure of Metallopnictinidenes $\mathbf{3}^{M,Pn}$

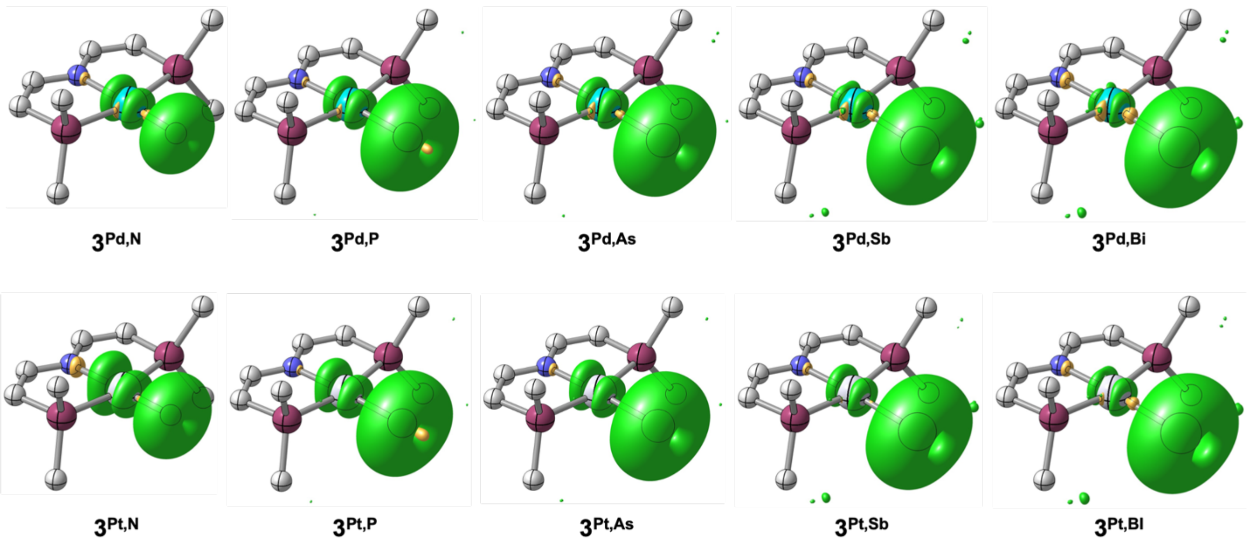

Figure S81: Spin densities of metallopnictinidenes $\mathbf{3}^{M,Pn}$ (M = Pd, Pt; Pn = N, P, As, Sb, Bi; isosurfaces at ±0.005 a_0^{-3}; methyl groups and hydrogen atoms not shown).

Table S12: M–Pn bond lengths $d_{M\text{–}Pn}$ in Å and results of the NBO analysis of the M–Pn bond in metallopnictinidenes $\mathbf{3}^{M,Pn}$ (M = Pd, Pt; Pn = N, P, As, Sb, Bi): Total Lewis character, Wiberg bond indices (WBI), NPA charges and spin populations at M and Pn, contributions of M and Pn to the NLMOs.

		$\mathbf{3}^{Pd,N}$	$\mathbf{3}^{Pd,P}$	$\mathbf{3}^{Pd,As}$	$\mathbf{3}^{Pd,Sb}$	$\mathbf{3}^{Pd,Bi}$	$\mathbf{3}^{Pt,N}$	$\mathbf{3}^{Pt,P}$	$\mathbf{3}^{Pt,As}$	$\mathbf{3}^{Pt,Sb}$	$\mathbf{3}^{Pt,Bi}$
Total Lewis character / %		98.73	98.73	98.81	98.88	98.99	98.86	98.87	98.94	98.99	99.09
$d_{M\text{–}Pn}$ / Å		1.891	2.263	2.378	2.583	2.676	1.894	2.275	2.393	2.603	2.700
WBI (M–Pn)		0.80	0.71	0.68	0.63	0.62	0.94	0.81	0.77	0.71	0.68
NPA charges	M	0.39	0.17	0.16	0.12	0.12	0.38	0.13	0.12	0.07	0.07
	Pn	–0.46	–0.25	–0.22	–0.14	–0.12	–0.52	–0.27	–0.23	–0.15	–0.12
NPA spin populations	M	0.07	0.04	0.02	0.00	–0.02	0.13	0.07	0.05	0.02	0.00
	Pn	1.91	1.88	1.90	1.93	1.95	1.81	1.83	1.86	1.90	1.92
σ(M–Pn)	M / %	64.02	51.98	51.99	49.96	50.07	63.17	51.60	51.18	49.18	49.18
	Pn / %	33.65	44.06	43.98	45.17	44.97	35.06	45.30	45.19	46.74	46.59
s(Pn)	M / %	0.54	0.47	0.45	0.47	0.42	0.30	0.25	0.23	0.25	0.22
	Pn / %	99.10	99.03	99.10	99.11	99.26	99.33	99.27	99.34	99.35	99.47
p_x(Pn)	M / %	0.10	0.11	0.08	0.14	0.11	0.31	0.11	0.12	0.12	0.10
	Pn / %	98.56	97.68	97.50	97.59	97.66	97.58	96.87	96.82	97.15	97.26
p_y(Pn)	M / %	0.06	0.04	0.03	0.03	0.02	0.18	0.04	0.03	0.02	0.02
	Pn / %	99.42	98.23	97.94	97.33	97.23	99.25	98.29	98.07	97.50	97.36

Table S13: Experimental M–Pn bond lengths $d_{M\text{–}Pn}$ of precursor complexes $\mathbf{1}^{M,Pn}$ and metallopnictinidenes $\mathbf{3}^{M,Pn}$ (both from *in crystallo* experiments using synchrotron radiation, see crystallographic details); PBE0 optimized M–Pn bond lengths d_{PBE0} of $\mathbf{1}^{M,Pn}$ and $\mathbf{3}^{M,Pn}$; estimated M–Pn single ($d_{M\text{–}Pn}$) and double bond lengths ($d_{M=Pn}$) based on Pyykkö's[53] covalent radii. All values in Å.

	$\mathbf{3}^{Pd,P}$	$\mathbf{3}^{Pd,As}$	$\mathbf{3}^{Pt,P}$	$\mathbf{3}^{Pt,As}$
$d_{M\text{–}Pn}$ ($\mathbf{1}^{M,Pn}$)	2.3665(6)	2.4616(8)	2.3574(16)	2.446(2)
$d_{M\text{–}Pn}$ ($\mathbf{3}^{M,Pn}$)	2.31(5)	2.349(13)	2.25(4)	2.36(3)
d_{PBE0} ($\mathbf{1}^{M,Pn}$)	2.38	2.49	2.39	2.50
d_{PBE0} ($\mathbf{3}^{M,Pn}$)	2.26	2.38	2.28	2.39
$d_{M\text{–}Pn}$ (Pyykkö)	2.31	2.41	2.34	2.44
$d_{M=Pn}$ (Pyykkö)	2.19	2.31	2.14	2.26

NEVPT2/CASSCF Computations on Metallopnictinidenes $3^{M,Pn}$

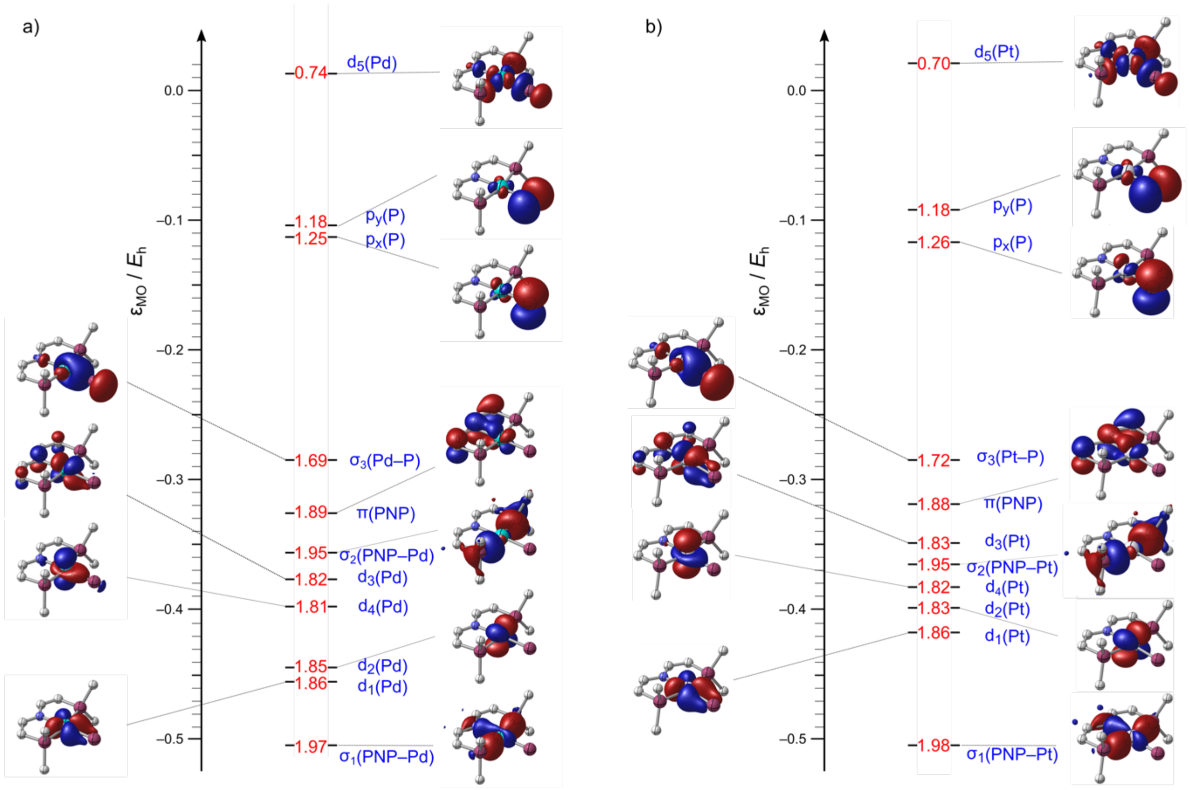

Figure S82: Orbital correlation diagram based on CASSCF(18,11) natural orbitals (orbital occupation numbers in red; state-averaging over 50 singlet, 45 triplet and 5 quintet states), for $\mathbf{3}^{\mathbf{Pd,P}}$ (a) and $\mathbf{3}^{\mathbf{Pt,P}}$ (b, triplet ground-state geometry; orbitals of other metallopnictinidenes $\mathbf{3}^{\mathbf{M,Pn}}$ equivalent; orbital isosurfaces at ±0.05 $a_0^{-3/2}$; methyl groups and hydrogen atoms not shown).

Table S14: CASSCF(18,11) natural orbital occupations and orbital energies in E_h for palladium pnictinidenes $\mathbf{3}^{\mathbf{Pd,Pn}}$ (Pn = N, P, As, Sb, Bi).

	$\mathbf{3}^{\mathbf{Pd,N}}$	$\mathbf{3}^{\mathbf{Pd,P}}$	$\mathbf{3}^{\mathbf{Pd,As}}$	$\mathbf{3}^{\mathbf{Pd,Sb}}$	$\mathbf{3}^{\mathbf{Pd,Bi}}$
d_1(M)	1.83 / –0.45	1.86 / –0.46	1.85 / –0.45	1.86 / –0.44	1.86 / –0.44
d_2(M)	1.84 / –0.44	1.85 / –0.45	1.85 / –0.44	1.86 / –0.44	1.86 / –0.44
d_3(M)	1.82 / –0.42	1.82 / –0.38	1.82 / –0.38	1.83 / –0.37	1.83 / –0.37
d_4(M)	1.80 / –0.41	1.81 / –0.40	1.82 / –0.39	1.83 / –0.39	1.83 / –0.40
d_5(M)	0.78 / 0.04	0.74 / 0.02	0.76 / 0.01	0.77 / 0.00	0.78 / –0.00
p_y(M)	1.19 / –0.11	1.18 / –0.10	1.15 / –0.10	1.12 / –0.10	1.12 / –0.09
p_x(M)	1.26 / –0.14	1.25 / –0.13	1.24 / –0.12	1.22 / –0.12	1.20 / –0.11
σ_1(PNP–M)	1.97 / –0.50	1.97 / –0.51	1.97 / –0.50	1.97 / –0.49	1.97 / –0.49
σ_2(PNP–M)	1.95 / –0.36	1.95 / –0.36	1.95 / –0.36	1.95 / –0.36	1.95 / –0.36
σ_3(M–Pn)	1.65 / –0.29	1.69 / –0.28	1.70 / –0.28	1.71 / –0.28	1.70 / –0.27
π_1(PNP)	1.91 / –0.29	1.89 / –0.33	1.89 / –0.33	1.89 / –0.33	1.89 / –0.32

Table S15: CASSCF(18,11) natural orbital occupations and orbital energies in E_h for platinum pnictinidenes **3**^{Pt,Pn} (Pn = N, P, As, Sb, Bi).

	**3**^{Pt,N}	**3**^{Pt,P}	**3**^{Pt,As}	**3**^{Pt,Sb}	**3**^{Pt,Bi}
d_1(M)	1.85 / –0.42	1.86 / –0.42	1.86 / –0.42	1.86 / –0.42	1.86 / –0.41
d_2(M)	1.83 / –0.40	1.83 / –0.40	1.84 / –0.41	1.84 / –0.41	1.85 / –0.40
d_3(M)	1.83 / –0.38	1.83 / –0.35	1.82 / –0.35	1.82 / –0.36	1.81 / –0.35
d_4(M)	1.81 / –0.38	1.82 / –0.38	1.80 / –0.37	1.81 / –0.36	1.82 / –0.36
d_5(M)	0.73 / 0.09	0.70 / 0.06	0.69 / 0.05	0.71 / 0.04	0.73 / 0.03
p_y(M)	1.20 / –0.09	1.18 / –0.09	1.19 / –0.09	1.19 / –0.10	1.15 / –0.09
p_x(M)	1.26 / –0.12	1.26 / –0.12	1.26 / –0.12	1.24 / –0.11	1.23 / –0.11
σ_1(PNP–M)	1.98 / –0.51	1.98 / –0.52	1.98 / –0.52	1.98 / –0.53	1.98 / –0.52
σ_2(PNP–M)	1.94 / –0.36	1.95 / –0.37	1.95 / –0.37	1.95 / –0.37	1.95 / –0.37
σ_3(M–Pn)	1.69 / –0.30	1.72 / –0.28	1.71 / –0.28	1.71 / –0.27	1.71 / –0.26
π_1(PNP)	1.90 / –0.30	1.88 / –0.32	1.89 / –0.32	1.89 / –0.32	1.90 / –0.32

Table S16: CASSCF(18,11)/NEVPT2-QDPT energy stabilization by spin-orbit-coupling of the ground state of metallopnictinidenes **3**^{M,Pn} (M = Pd, Pt; Pn = N, P, As, Sb, Bi; ΔE(SOC) in cm^{-1}) and zero-field-splitting parameters (D in cm^{-1}).

	**3**^{Pd,N}	**3**^{Pd,P}	**3**^{Pd,As}	**3**^{Pd,Sb}	**3**^{Pd,Bi}	**3**^{Pt,N}	**3**^{Pt,P}	**3**^{Pt,As}	**3**^{Pt,Sb}	**3**^{Pt,Bi}
ΔE(SOC) / cm^{-1}	–105	–106	–216	–671	–3635	–883	–838	–980	–1402	–4114
D / cm^{-1}	6.4	12	95	425	2240	80	70	172	481	2161
E / |D|	0.041	0.051	0.011	0.001	0.002	0.072	0.063	0.012	0.009	0.001

Table S17: CASSCF(18,11)/NEVPT2 transition energies and leading configuration weights for the lowest three states of $\mathbf{3}^{\mathbf{M,Pn}}$ (M = Pd, Pt; Pn = N, P, As, Sb, Bi). Individual further configurations do not contribute significantly to the ground state wave function.

compound	state	root	mult	weight	configuration	Δ*E* / kcal mol^{-1}
$\mathbf{3}^{\mathbf{Pd,N}}$	0	0	3	89.0%	[22222222110]	0.0
	1	0	1	63.9%	[22222222200]	23.0
				22.4%	[22222222020]	
	2	1	1	85.7%	[22222222110]	25.3
$\mathbf{3}^{\mathbf{Pd,P}}$	0	0	3	89.8%	[22222222110]	0.0
	1	0	1	73.7%	[22222222200]	13.7
				15.0%	[22222222020]	
	2	1	1	88.3%	[22222222110]	17.0
$\mathbf{3}^{\mathbf{Pd,As}}$	0	0	3	93.4%	[22222222110]	0.0
	1	0	1	75.6%	[22222222200]	14.3
				16.4%	[22222222020]	
	2	1	1	91.8%	[22222222110]	17.1
$\mathbf{3}^{\mathbf{Pd,Sb}}$	0	0	3	88.1%	[22222222110]	
	1	0	1	71.4%	[22222222200]	13.7
				16.3%	[22222222020]	
	2	1	1	87.5%	[22222222110]	15.7
$\mathbf{3}^{\mathbf{Pd,Bi}}$	0	0	3	86.0%	[22222222110]	0.0
	1	0	1	68.6%	[22222222200]	13.9
				17.2%	[22222222020]	
	2	1	1	85.7%	[22222222110]	15.7
$\mathbf{3}^{\mathbf{Pt,N}}$	0	0	3	91.9%	[22222222110]	0.0
	1	0	1	66.9%	[22222222200]	20.7
				23.2%	[22222222020]	
	2	1	1	89.7%	[22222222110]	22.9
$\mathbf{3}^{\mathbf{Pt,P}}$	0	0	3	89.2%	[22222222110]	0.0
	1	0	1	72.2%	[22222222200]	12.8
				16.2%	[22222222020]	
	2	1	1	88.0%	[22222222110]	16.1
$\mathbf{3}^{\mathbf{Pt,As}}$	0	0	3	92.7%	[22222222110]	0.0
	1	0	1	74.2%	[22222222200]	13.5
				17.3%	[22222222020]	
	2	1	1	91.4%	[22222222110]	16.4
$\mathbf{3}^{\mathbf{Pt,Sb}}$	0	0	3	91.7%	[22222222110]	0.0
	1	0	1	71.4%	[22222222200]	13.3
				19.3%	[22222222020]	
	2	1	1	90.6%	[22222222110]	15.2
$\mathbf{3}^{\mathbf{Pt,Bi}}$	0	0	3	91.0%	[22222222110]	0.0
	1	0	1	69.9%	[22222222200]	13.6
				20.3%	[22222222020]	
	2	1	1	90.0%	[22222222110]	15.3

Computed UV-vis spectra of Metallopnictinidenes $3^{M,Pn}$

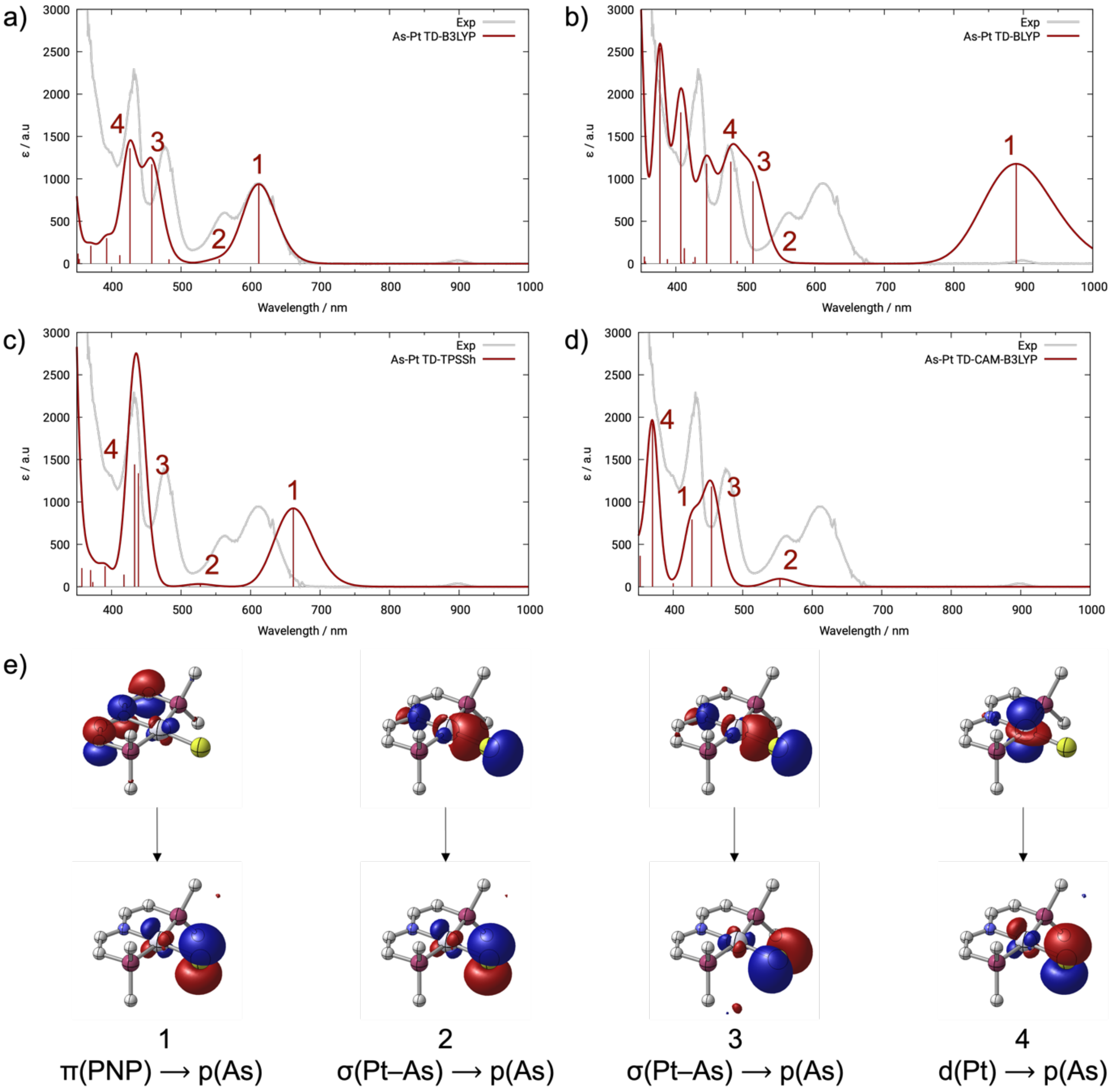

Figure S83: TD-DFT computed UV-vis spectra of metallopnictinidenes $\mathbf{3}^{\mathbf{Pt,As}}$ employing density functionals, B3YLP (a), BLYP (b), TPSSh (c), and CAM-B3LYP (d). e) Leading NTOs of selected excitations 1–4 of $\mathbf{3}^{\mathbf{Pt,As}}$ (isosurfaces at ±0.05 $a_0^{-3/2}$; methyl groups and hydrogen atoms not shown). Excitation energies are shown in Table S18.

Table S18: TD-DFT computed energies of selected excitations of $\mathbf{3}^{\mathbf{Pt,As}}$ in cm^{-1} employing density functionals B3YLP, BLYP, TPSSh and CAM-B3LYP.

Transition		B3LYP	BLYP	TPSSh	CAM-B3LYP	Exp.
1	π(PNP) ⟶ p(As)	16300	11200	15100	23400	16400
2	σ(Pt–As) ⟶ p(As)	18000	17500	18900	18000	17800
3	σ(Pt–As) ⟶ p(As)	21800	19600	22800	22000	21000
4	d(Pt) ⟶ p(As)	23400	20900	23100	27000	23100

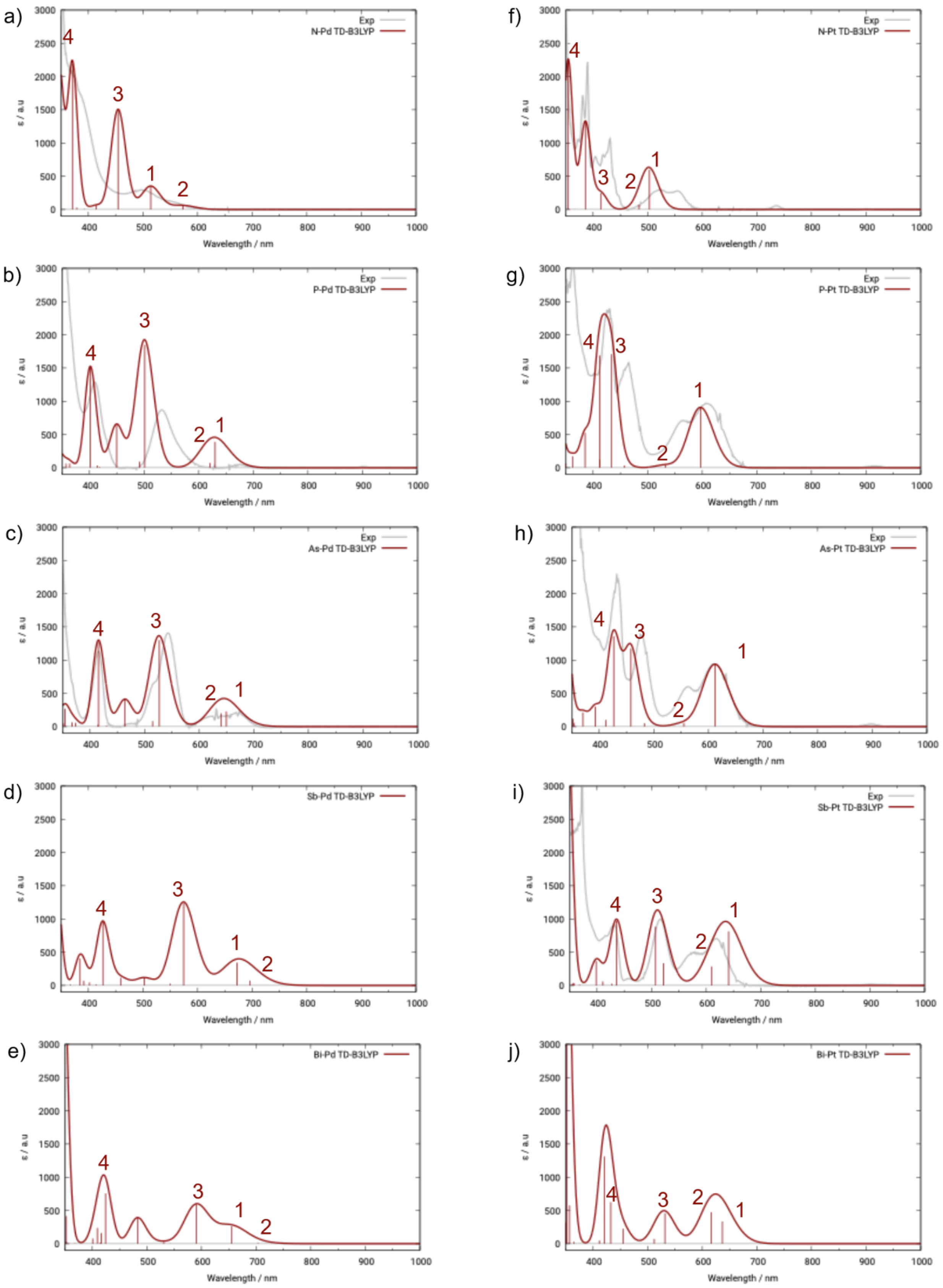

Figure S84: Experimental (gray) and TD-DFT computed (red) UV-vis spectra of metallopnictinidenes $\mathbf{3}^{\mathbf{M,Pn}}$ (M = Pd, Pt; Pn = N, P, As, Sb, Bi). Further details are provided in Figure S83 and Table S19.

Table S19: TD-DFT computed energies of selected excitations of metallopnictinidenes $\mathbf{3}^{M,Pn}$ (M = Pd, Pt; Pn = N, P, As, Sb, Bi) in cm^{-1}, oscillator strengths in parentheses.

	Transition	$\mathbf{3}^{Pd,N}$		$\mathbf{3}^{Pd,P}$		$\mathbf{3}^{Pd,As}$		$\mathbf{3}^{Pd,Sb}$		$\mathbf{3}^{Pd,Bi}$	
1	π(PNP) ⟶ p(Pn)	19400	(0.0024)	15900	(0.0027)	15400	(0.0016)	14900	(0.0024)	15300	(0.0018)
2	σ(Pd–Pn) ⟶ p(Pn)	17500	(0.0004)	16100	(0.0005)	15600	(0.0014)	14400	(0.0005)	14200	(0.0001)
3	σ(Pd–Pn) ⟶ p(Pn)	22000	(0.0104)	20000	(0.0128)	19000	(0.0090)	17400	(0.0086)	16900	(0.0041)
4	d(Pd) ⟶ p(Pn)	28800	(0.0143)	24900	(0.0104)	24100	(0.0088)	23400	(0.0066)	23600	(0.0052)

	Transition	$\mathbf{3}^{Pt,N}$		$\mathbf{3}^{Pt,P}$		$\mathbf{3}^{Pt,As}$		$\mathbf{3}^{Pt,Sb}$		$\mathbf{3}^{Pt,Bi}$	
1	π(PNP) ⟶ p(Pn)	19900	(0.0041)	16800	(0.0063)	16300	(0.0065)	15600	(0.0056)	15700	(0.0023)
2	σ(Pt–Pn) ⟶ p(Pn)	20700	(0.0005)	18800	(0.0003)	18000	(0.0004)	16400	(0.0020)	16200	(0.0033)
3	σ(Pt–Pn) ⟶ p(Pn)	25900	(0.0017)	23000	(0.0118)	21800	(0.0081)	19700	(0.0061)	18800	(0.0032)
4	d(Pt) ⟶ p(Pn)	28200	(0.0156)	24300	(0.0117)	23400	(0.0094)	22900	(0.0067)	23100	(0.0043)

Table S20: TD-DFT computed molecular orbital energies of p_y(Pn), p_x(Pn) and π_1(PNP) of pnictinidenes $\mathbf{3}^{M,Pn}$ (M = Pd, Pt; Pn = N, P, As, Sb, Bi) and orbital energy differences Δ*E* relative to π_1(PNP) in eV.

	$\mathbf{3}^{Pd,N}$	$\mathbf{3}^{Pd,P}$	$\mathbf{3}^{Pd,As}$	$\mathbf{3}^{Pd,Sb}$	$\mathbf{3}^{Pd,Bi}$	$\mathbf{3}^{Pt,N}$	$\mathbf{3}^{Pt,P}$	$\mathbf{3}^{Pt,As}$	$\mathbf{3}^{Pt,Sb}$	$\mathbf{3}^{Pt,Bi}$
p_y (Pn)	–1.1	–1.8	–1.9	–2.0	–2.0	–0.9	–1.6	–1.7	–1.9	–1.9
p_x (Pn)	–1.8	–2.3	–2.3	–2.4	–2.3	–1.8	–2.2	–2.3	–2.4	–2.3
π_1 (PNP)	–5.0	–5.0	–4.9	–4.9	–4.8	–5.1	–5.0	–5.0	–5.0	–4.9
$\Delta E(p_y - \pi_1)$	3.9	3.2	3.0	2.9	2.9	4.2	3.4	3.2	3.0	3.0
$\Delta E(p_x - \pi_1)$	3.2	2.7	2.6	2.5	2.5	3.3	2.8	2.7	2.6	2.6

Detailed computational results

Table S21: Total Energies and thermal contributions to Gibbs free energies and ⟨S^2⟩ expectation values of species studied.

Compound				E_{total}[a] / a.u.	E_{total}[b] / a.u.	$\Delta G_{contrib}$[c]	⟨S^2⟩	G / a.u.
Precursor complexes ($1^{M,Pn}$)								
[(PNP)Pd-N_3] (**$1^{Pd,N}$**)		^1A	C_1	-1813.548315	-1814.901916	0.516436		-1814.385480
[(PNP)Pd-PCO] (**$1^{Pd,P}$**)		^1A	C_1	-2103.896738	-2105.310378	0.510715		-2104.799663
[(PNP)Pd-AsCO] (**$1^{Pd,As}$**)		^1A	C_1	-3998.107753	-3999.681364	0.508702		-3999.172662
[(PNP)Pd-SbH_2] (**$1^{Pd,Sb}$**)		^1A	C_1	-1891.102808	-1892.279268	0.518060		-1891.761208
[(PNP)Pd- $BiMe_2$] (**$1^{Pd,Bi}$**)		^1A	C_1	-1943.946931	-1945.212790	0.571358		-1944.641432
[(PNP)Pt-N_3] (**$1^{Pt,N}$**)		^1A	C_1	-1805.043440	1806.378750	0.516177		-1805.862573
[(PNP)Pt-PCO] (**$1^{Pt,P}$**)		^1A	C_1	-2095.391726	-2096.787894	0.511836		-2096.276058
[(PNP)Pt-AsCO] (**$1^{Pt,As}$**)		^1A	C_1	-3989.601925	-3991.157501	0.509013		-3990.648488
[(PNP)Pt-SbH_2] (**$1^{Pt,Sb}$**)		^1A	C_1	-1882.596851	-1883.756286	0.518112		-1883.238174
[(PNP)Pt-$BiMe_2$] (**$1^{Pt,Bi}$**)		^1A	C_1	-1935.439978	-1936.688055	0.571763		-1936.116292
Metallopnictinidenes ($3^{M,Pn}$)								
	T	$^3A''$	C_s	-1704.175777	-1705.405924	0.505669	2.0191	-1704.900255
[(PNP)Pd-N] (**$3^{Pd,N}$**)	CSS	^1A	C_1	-1704.130233	-1705.363997	0.507163		-1704.856834
	BSS	^1A	C_1	-1704.158717	-1705.390292	0.506566	0.9797	-1704.883726
	T	$^3A''$	C_s	-1990.724839	-1992.004345	0.504452	2.0100	-1991.499893
[(PNP)Pd-P] (**$3^{Pd,P}$**)	CSS	^1A	C_1	-1990.699650	-1991.981787	0.505980		-1991.475807
	BSS	^1A	C_1	-1990.715864	-1991.996544	0.505720	0.9448	-1991.490824
	T	$^3A''$	C_s	-3884.958709	-3886.400026	0.503240	2.0097	-3885.896786
[(PNP)Pd-As] (**$3^{Pd,As}$**)	CSS	^1A	C_1	-3884.934218	-3886.377753	0.504127		-3885.873626
	BSS	^1A	C_1	-3884.949215	-3886.391418	0.504189	0.9395	-3885.887229
	T	$^3A''$	C_s	-1889.902802	-1891.075110	0.502566	2.0107	-1890.572544
[(PNP)Pd-Sb] (**$3^{Pd,Sb}$**)	CSS	^1A	C_1	-1889.880201	-1891.054843	0.503872		-1890.550971
	BSS	^1A	C_1	-1889.893589	-1891.066808	0.503033	0.9386	-1890.563775
	T	$^3A''$	C_s	-1864.290409	-1865.462302	0.501581	2.0138	-1864.960721
[(PNP)Pd-Bi] (**$3^{Pd,Bi}$**)	CSS	^1A	C_1	-1864.267977	-1865.442435	0.502886		-1864.939549
	BSS	^1A	C_1	-1864.280643	-1865.453507	0.501807	0.9376	-1864.95170
	T	^3A	C_s	-1695.678455	-1696.891852	0.505517	2.0162	-1696.386335
	CSS	^1A	C_1	-1695.637006	-1696.855518	0.507056		-1696.348462
[(PNP)Pt-N] (**$3^{Pt,N}$**)	BSS	^1A	C_1	-1695.662632	-1696.877398	0.506618	0.9747	-1696.37078
	T	^3A	C_s	-1695.678455	-1696.891852	0.505517	2.0162	-1696.386335
	CSS	^1A	C_1	-1695.637006	-1696.855518	0.507056		-1696.348462
	BSS	^1A	C_1	-1695.662632	-1696.877398	0.506618	0.9747	-1696.37078
	T	$^3A''$	C_s	-1982.223658	-1983.486880	0.504763	2.0095	-1982.982117
[(PNP)Pt-P] (**$3^{Pt,P}$**)	CSS	^1A	C_1	-1982.198826	-1983.465938	0.506392		-1982.959546
	BSS	^1A	C_1	-1982.214819	-1983.479403	0.506026	0.9423	-1982.973377
	T	$^3A''$	C_s	-3876.455797	-3877.880198	0.503806	2.0079	-3877.376392
[(PNP)Pt-As] (**$3^{Pt,As}$**)	CSS	^1A	C_1	-3876.430614	-3877.858279	0.502489		-3877.355790
	BSS	^1A	C_1	-3876.446190	-3877.871760	0.504481	0.9450	-3877.367279
	T	$^3A''$	C_s	-1881.397388	-1882.552814	0.503187	2.0079	-1882.049627
[(PNP)Pt-Sb] (**$3^{Pt,Sb}$**)	CSS	^1A	C_1	-1881.374137	-1882.532924	0.503890		-1882.029034
	BSS	^1A	C_1	-1881.388104	-1882.544654	0.503386	0.9430	-1882.041268
	T	$^3A''$	C_s	-1855.783339	-1856.938263	0.502321	2.0091	-1856.435942
[(PNP)Pt-Bi] (**$3^{Pt,Bi}$**)	CSS	^1A	C_1	-1855.760181	-1856.918612	0.503129		-1856.415483
	BSS	^1A	C_1	-1855.773538	-1856.929608	0.502551	0.9433	-1856.427057
Dipnictenes ($2^{M,Pn}$)								
[(μ-P_2){(PNP)Pd}$_2$] (**$2^{Pd,P}$**)		^1A	C_1	-3981.578965	-3984.137132	1.040830		-3983.096302
[(μ-As_2){(PNP)Pd}$_2$] (**$2^{Pd,As}$**)		^1A	C_1	-7770.023303	-7772.904032	1.035775		-7771.868257
[(μ-Sb_2){(PNP)Pd}$_2$] (**$2^{Pd,Sb}$**)		^1A	C_1	-3779.896643	-3782.241569	1.03307		-3781.208499
[(μ-Bi_2){(PNP)Pd}$_2$] (**$2^{Pd,Sb}$**)		^1A	C_1	-3728.661919	-3731.006336	1.028235		-3729.978101
[(μ-P_2){(PNP)Pt}$_2$] (**$2^{Pt,P}$**)		^1A	C_1	-3964.573099	-3967.097346	1.041246		-3966.056100
[(μ-As_2){(PNP)Pt}$_2$] (**$2^{Pt,As}$**)		^1A	C_1	-7753.014461	-7755.860706	1.036696		-7754.824010
[(μ-Sb_2){(PNP)Pt}$_2$] (**$2^{Pd,Sb}$**)		^1A	C_1	-3762.883593	-3765.193925	1.033784		-3764.160141
[(μ-Bi_2){(PNP)Pt}$_2$] (**$2^{Pd,Sb}$**)		^1A	C_1	-3711.645732	-3713.955391	1.029263		-3712.926128
Dipnictenyl radical cations ([**$2^{M,Pn}$**]$^+$)								
[(μ-P_2){(PNP)Pd}$_2$]$^+$ ([**$2^{Pd,P}$**]$^+$)		^2A	C_1	-3981.393557	-3983.953186	1.038764	0.7608	-3982.914422
[(μ-As_2){(PNP)Pd}$_2$]$^+$ ([**$2^{Pd,As}$**]$^+$)		^2A	C_1	-7769.834743	-7772.715533	1.035745	0.7633	-7771.679788
[(μ-Sb_2){(PNP)Pd}$_2$]$^+$ ([**$2^{Pd,Sb}$**]$^+$)		^2A	C_1	-3779.699245	-3782.043673	1.031294	0.7940	-3781.012379
[(μ-Bi_2){(PNP)Pd}$_2$]$^+$ ([**$2^{Pd,Bi}$**]$^+$)		^2A	C_1	-3728.464468	-3730.808091	1.028865	0.7777	-3729.779226
[(μ-P_2){(PNP)Pt}$_2$]$^+$ ([**$2^{Pt,P}$**]$^+$)		^2A	C_1	-3964.389725	-3966.916151	1.040240	0.7600	-3965.875911
[(μ-As_2){(PNP)Pt}$_2$]$^+$ ([**$2^{Pt,As}$**]$^+$)		^2A	C_1	-7752.827677	-7755.674409	1.036512	0.7623	-7754.637897
[(μ-Sb_2){(PNP)Pt}$_2$]$^+$ ([**$2^{Pt,Sb}$**]$^+$)		^2A	C_1	-3762.688898	-3764.999336	1.031539	0.7799	-3763.967797
[(μ-Bi_2){(PNP)Pt}$_2$]$^+$ ([**$2^{Pt,Bi}$**]$^+$)		^2A	C_1	-3711.452051	-3713.761319	1.030059	0.7725	-3712.73126

[a]PBE0-D3B/def2-SVP energies, [b]PBE0-D3BJ/def2-TZVPP energies, [c]Contributions to Gibbs free energy at 298 K.

Crystallographic Details

CCDC 2383559 ([Pd(PCO)(PNP)], $\mathbf{1}^{\mathbf{Pd,P}}$), CCDC 2383512 ([Pd(AsCO)(PNP)], $\mathbf{1}^{\mathbf{Pd,As}}$), CCDC 2383555 ([Pt(AsCO)(PNP)], $\mathbf{1}^{\mathbf{Pt,As}}$), CCDC 2387991 ([Pt(BiMe$_2$)(PNP)], $\mathbf{1}^{\mathbf{Pt,Bi}}$), CCDC 2383521 ([(*μ*-P$_2$){(PNP)Pd}$_2$], $\mathbf{2}^{\mathbf{Pd,P}}$),CCDC 2383513 ([(*μ*-As$_2$){Pd(PNP)}$_2$] $\mathbf{2}^{\mathbf{Pd,As}}$), CCDC 2383556 ([(*μ*-As$_2$){Pt(PNP)}$_2$] $\mathbf{2}^{\mathbf{Pt,As}}$), CCDC 2383519 ([(*μ*-Sb$_2$){Pt(PNP)}$_2$] $\mathbf{2}^{\mathbf{Pt,Sb}}$), CCDC 2387995 ([(*μ*-Bi$_2$){Pt(PNP)}$_2$] $\mathbf{2}^{\mathbf{Pt,Bi}}$), CCDC 2381476 ([Pd(P)(PNP)], $\mathbf{3}^{\mathbf{Pd,P}}$), CCDC 2381475 ([Pd(As)(PNP)], $\mathbf{3}^{\mathbf{Pd,As}}$), CCDC 2381478 ([Pt(P)(PNP)], $\mathbf{3}^{\mathbf{Pt,P}}$), CCDC 2381477 ([Pt(As)(PNP)], $\mathbf{3}^{\mathbf{Pt,As}}$) contain the supplementary crystallographic data for this paper. These data can be obtained free of charge from https://www.ccdc.cam.ac.uk/structures/ (or from Cambridge Crystallographic Data Centre, 12 Union Road, Cambridge, CB2 1EZ, UK. Fax: +44-1223-336-033; e-mail: deposit@ccdc.cam.ac.uk).

Suitable single crystals for X-ray structure determination were selected from the mother liquor under an inert gas atmosphere and transferred in protective perfluoro polyether oil on a microscope slide. The selected and mounted crystals were transferred to the cold gas stream on the diffractometer. The diffraction data were obtained at 100 K on a Bruker D8 three-circle diffractometer, equipped with a PHOTON 100 CMOS or PHOTON III detector (for all other crystals structures besides [(PNP)M-Pn], ($\mathbf{3}^{\mathbf{M,Pn}}$) (M = Pd, Pt; Pn = P, As), and an INCOATEC microfocus source with Quazar mirror optics (Mo-Kα radiation, λ= 0.71073 Å).

The data obtained were integrated with SAINT and a semi-empirical absorption correction from equivalents with SADABS was applied. The structures were solved and refined using the Bruker SHELX 2014 and 2018 software package.[54–57] All non-hydrogen atoms were refined with anisotropic displacement parameters. All C-H hydrogen atoms were refined isotropically on calculated positions by using a riding model with their Uiso values constrained to 1.5 Ueq of their pivot atoms for terminal sp^3 carbon atoms and 1.2 times for all other atoms.

For the *in crystallo* experiments, the X-ray crystal structure of [(PNP)M-Pn], ($\mathbf{3}^{\mathbf{M,Pn}}$) (M = Pd, Pt; Pn = P, As), was collected using synchrotron radiation (λ = 0.41328 Å) at ChemMatCARS located at the Advanced Photon Source (APS) housed at Argonne National Laboratory (ANL). Crystals of [(PNP)M-PnCO], ($\mathbf{1}^{\mathbf{M,Pn}}$) (M = Pd, Pt; Pn = P, As), suitable for X-ray diffraction were mounted on a glass fiber. The data was collected at 100 K (Oxford Cryosystems N_2 cold stream) using a vertically mounted Bruker D8 three-circle platform goniometer equipped with a PILATUS3 X CdTe 1M detector. Data was collected as a series of φ and/or ω scans. Data were integrated using SAINT and scaled with a multi-scan absorption correction using SADABS. Structures were solved by intrinsic phasing using SHELXT and refined against F2 on all data by full matrix least squares with SHELXL. All non-hydrogen atoms were refined anisotropically. H atoms were placed at idealized positions and refined using a riding model. Disorder was modeled using two parts and the thermal ellipsoids in the disordered molecules were restrained using the SHELXL instruction SIMU.

In crystallo photolysis was pursued to acquire solid-state structural data of metallopnicitinidenes [(PNP)M-Pn], ($\mathbf{3}^{\mathbf{M,Pn}}$) (M = Pd, Pt; Pn = P, As). In these experiments, photolysis of single crystal samples of each of the PCO and AsCO complexes were irradiated (λ = 365 nm) at 100 K and structural data was acquired before and after irradiation using synchrotron radiation (λ = 0.41328 Å).

The structures of phosphaethynolate complexes [(PNP)M-PCO], ($\mathbf{1}^{\mathbf{M,P}}$) (M = Pd, Pt) were determined from crystals in which 41% chemical conversion to the corresponding metallophosphinidene [(PNP)M-P], ($\mathbf{3}^{\mathbf{M,P}}$) (M = Pd, Pt) was achieved. Further irradiation led to significant loss of crystallinity and increase in uncertainty in the determination of metrical parameters.: Following photolysis, the extrusion of CO is accompanied by the contraction of the Pd-P distance from 2.3665(6) Å to 2.31(5) Å and from 2.3574(16) Å to 2.25(4) Å for Pt–P, respectively.

Refinement of diffraction data following *in crystallo* photolysis of arsaethynolate complexes [(PNP)M-AsCO], ($\mathbf{1}^{\mathbf{M,As}}$) (M = Pd, Pt), indicated 25% and 14% conversion to the corresponding metalloarsinidenes [(PNP)M-As], ($\mathbf{3}^{\mathbf{M,As}}$) (M = Pd, Pt), respectively. Following photolysis, the Pd–As distance contracted from 2.4616(8) Å to 2.349(13) Å while the Pt–As distance contracted from 2.446(2) Å to 2.36(3) Å. Due to a combination of relatively modest solid-state conversion and the potential for CO diffusion from crystalline samples, we were unable to locate gaseous carbon monoxide in the crystalline lattice.

X-ray Single-Crystal Structure Analysis of [Pd(PCO)(PNP)] ($1^{Pd,P}$)

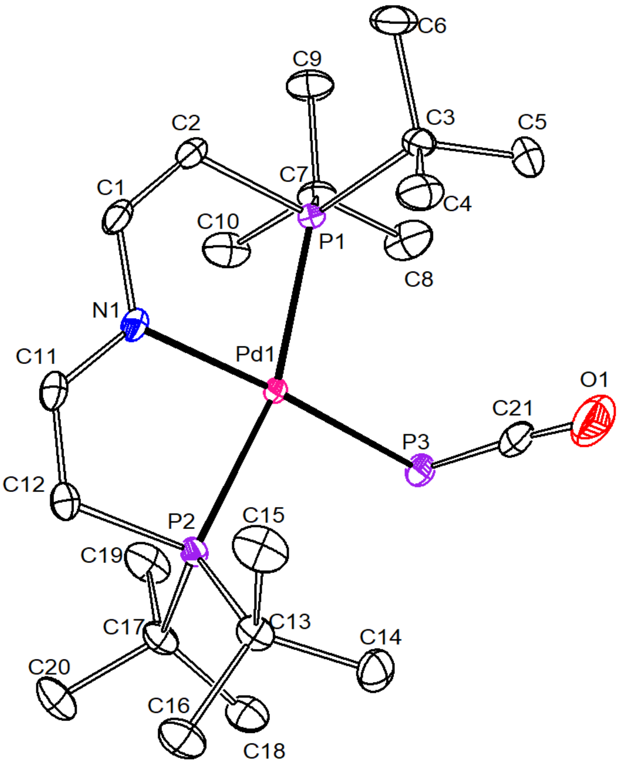

Figure S85: Thermal ellipsoid plot of $1^{Pd,P}$ with the anisotropic displacement parameters drawn at the 50% probability level. The asymmetric unit contains one complex molecule.

Table S22: Crystal data and structure refinement for $1^{Pd,P}$.

Identification code	JS_110521_2_MO	(JS-7-114)
Empirical formula	$C_{21} H_{40} N O P_3 Pd$	
Formula weight	521.85	
Temperature	100(2) K	
Wavelength	0.71073 Å	
Crystal system	Orthorhombic	
Space group	Pbca	
Unit cell dimensions	a = 15.1443(6) Å	a= 90°
	b = 13.9684(6) Å	b= 90°
	c = 23.4566(10) Å	g = 90°
Volume	4962.0(4) Å3	
Z	8	
Density (calculated)	1.397 Mg/m3	
Absorption coefficient	0.953 mm-1	
F(000)	2176	
Crystal size	0.382 x 0.364 x 0.321 mm3	
Crystal shape and color	Block,	clear intense red
Theta range for data collection	2.165 to 36.354°.	
Index ranges	-25<=h<=25, -23<=k<=23, -39<=l<=39	
Reflections collected	299364	
Independent reflections	12041 [R(int) = 0.0349]	
Completeness to theta = 25.242°	100.0 %	
Refinement method	Full-matrix least-squares on F2	
Data / restraints / parameters	12041 / 0 / 256	
Goodness-of-fit on F2	1.107	
Final R indices [I>2sigma(I)]	R1 = 0.0178,	wR2 = 0.0421
R indices (all data)	R1 = 0.0198,	wR2 = 0.0431
Largest diff. peak and hole	0.534 and -0.703 eÅ-3	

Table S23: Bond lengths [Å] and angles [°] for **1**^{Pd,P}.

Pd(1)-N(1)	2.0406(6)	C(12)-P(2)-C(13)	104.62(4)
Pd(1)-P(2)	2.3347(2)	C(12)-P(2)-C(17)	105.46(4)
Pd(1)-P(1)	2.3399(2)	C(13)-P(2)-C(17)	112.55(4)
Pd(1)-P(3)	2.3636(2)	C(12)-P(2)-Pd(1)	99.50(3)
P(1)-C(2)	1.7869(8)	C(13)-P(2)-Pd(1)	117.05(3)
P(1)-C(7)	1.8702(8)	C(17)-P(2)-Pd(1)	115.32(3)
P(1)-C(3)	1.8784(8)	C(21)-P(3)-Pd(1)	99.63(3)
P(2)-C(12)	1.7859(8)	C(1)-N(1)-C(11)	120.69(7)
P(2)-C(13)	1.8727(8)	C(1)-N(1)-Pd(1)	119.62(5)
P(2)-C(17)	1.8766(8)	C(11)-N(1)-Pd(1)	119.62(5)
P(3)-C(21)	1.6337(10)	C(2)-C(1)-N(1)	122.79(7)
O(1)-C(21)	1.1753(13)	C(1)-C(2)-P(1)	115.68(6)
N(1)-C(1)	1.3619(10)	C(5)-C(3)-C(4)	109.87(8)
N(1)-C(11)	1.3643(11)	C(5)-C(3)-C(6)	109.60(8)
C(1)-C(2)	1.3486(12)	C(4)-C(3)-C(6)	108.20(8)
C(3)-C(5)	1.5264(14)	C(5)-C(3)-P(1)	112.89(7)
C(3)-C(4)	1.5377(13)	C(4)-C(3)-P(1)	104.27(6)
C(3)-C(6)	1.5381(12)	C(6)-C(3)-P(1)	111.79(6)
C(7)-C(8)	1.5316(13)	C(8)-C(7)-C(9)	109.51(8)
C(7)-C(9)	1.5339(12)	C(8)-C(7)-C(10)	108.54(8)
C(7)-C(10)	1.5365(14)	C(9)-C(7)-C(10)	109.39(8)
C(11)-C(12)	1.3489(12)	C(8)-C(7)-P(1)	112.85(7)
C(13)-C(14)	1.5305(12)	C(9)-C(7)-P(1)	111.77(6)
C(13)-C(16)	1.5336(12)	C(10)-C(7)-P(1)	104.59(6)
C(13)-C(15)	1.5357(12)	C(12)-C(11)-N(1)	122.61(7)
C(17)-C(18)	1.5281(13)	C(11)-C(12)-P(2)	115.68(6)
C(17)-C(20)	1.5372(12)	C(14)-C(13)-C(16)	109.64(7)
C(17)-C(19)	1.5383(12)	C(14)-C(13)-C(15)	107.86(8)
N(1)-Pd(1)-P(2)	82.57(2)	C(16)-C(13)-C(15)	109.49(7)
N(1)-Pd(1)-P(1)	82.54(2)	C(14)-C(13)-P(2)	112.34(5)
P(2)-Pd(1)-P(1)	165.035(7)	C(16)-C(13)-P(2)	111.88(6)
N(1)-Pd(1)-P(3)	173.83(2)	C(15)-C(13)-P(2)	105.44(6)
P(2)-Pd(1)-P(3)	95.458(8)	C(18)-C(17)-C(20)	109.67(7)
P(1)-Pd(1)-P(3)	99.216(8)	C(18)-C(17)-C(19)	109.25(8)
C(2)-P(1)-C(7)	105.02(4)	C(20)-C(17)-C(19)	107.83(7)
C(2)-P(1)-C(3)	103.65(4)	C(18)-C(17)-P(2)	112.25(6)
C(7)-P(1)-C(3)	113.97(4)	C(20)-C(17)-P(2)	112.39(6)
C(2)-P(1)-Pd(1)	99.34(3)	C(19)-C(17)-P(2)	105.24(6)
C(7)-P(1)-Pd(1)	115.62(3)	O(1)-C(21)-P(3)	175.81(8)
C(3)-P(1)-Pd(1)	116.42(3)		

Symmetry transformations used to generate equivalent atoms:

Table S24: Torsion angles [°] for **1**^{Pd,P}.

C(11)-N(1)-C(1)-C(2)	-178.05(9)	C(1)-N(1)-C(11)-C(12)	176.14(9)
Pd(1)-N(1)-C(1)-C(2)	-1.19(12)	Pd(1)-N(1)-C(11)-C(12)	-0.72(13)
N(1)-C(1)-C(2)-P(1)	-0.49(12)	N(1)-C(11)-C(12)-P(2)	1.17(14)
C(7)-P(1)-C(2)-C(1)	121.41(7)	C(13)-P(2)-C(12)-C(11)	120.40(8)
C(3)-P(1)-C(2)-C(1)	-118.72(7)	C(17)-P(2)-C(12)-C(11)	-120.70(8)
Pd(1)-P(1)-C(2)-C(1)	1.54(7)	Pd(1)-P(2)-C(12)-C(11)	-0.95(8)
C(2)-P(1)-C(3)-C(5)	-161.11(7)	C(12)-P(2)-C(13)-C(14)	-172.58(6)
C(7)-P(1)-C(3)-C(5)	-47.54(7)	C(17)-P(2)-C(13)-C(14)	73.43(7)
Pd(1)-P(1)-C(3)-C(5)	91.00(7)	Pd(1)-P(2)-C(13)-C(14)	-63.62(6)
C(2)-P(1)-C(3)-C(4)	79.67(6)	C(12)-P(2)-C(13)-C(16)	63.59(7)
C(7)-P(1)-C(3)-C(4)	-166.76(6)	C(17)-P(2)-C(13)-C(16)	-50.40(7)
Pd(1)-P(1)-C(3)-C(4)	-28.22(7)	Pd(1)-P(2)-C(13)-C(16)	172.55(5)
C(2)-P(1)-C(3)-C(6)	-36.99(8)	C(12)-P(2)-C(13)-C(15)	-55.35(7)
C(7)-P(1)-C(3)-C(6)	76.57(8)	C(17)-P(2)-C(13)-C(15)	-169.35(6)
Pd(1)-P(1)-C(3)-C(6)	-144.88(6)	Pd(1)-P(2)-C(13)-C(15)	53.61(7)
C(2)-P(1)-C(7)-C(8)	179.52(7)	C(12)-P(2)-C(17)-C(18)	-159.14(6)
C(3)-P(1)-C(7)-C(8)	66.77(8)	C(13)-P(2)-C(17)-C(18)	-45.66(7)
Pd(1)-P(1)-C(7)-C(8)	-72.12(7)	Pd(1)-P(2)-C(17)-C(18)	92.17(6)

C(2)-P(1)-C(7)-C(9)	55.56(8)	C(12)-P(2)-C(17)-C(20)	-34.97(7)
C(3)-P(1)-C(7)-C(9)	-57.19(8)	C(13)-P(2)-C(17)-C(20)	78.52(7)
Pd(1)-P(1)-C(7)-C(9)	163.92(6)	Pd(1)-P(2)-C(17)-C(20)	-143.65(6)
C(2)-P(1)-C(7)-C(10)	-62.69(6)	C(12)-P(2)-C(17)-C(19)	82.13(7)
C(3)-P(1)-C(7)-C(10)	-175.44(6)	C(13)-P(2)-C(17)-C(19)	-164.39(6)
Pd(1)-P(1)-C(7)-C(10)	45.68(6)	Pd(1)-P(2)-C(17)-C(19)	-26.56(7)

Symmetry transformations used to generate equivalent atoms:

X-ray Single-Crystal Structure Analysis of [Pd(AsCO)(PNP)] ($1^{Pd,As}$)

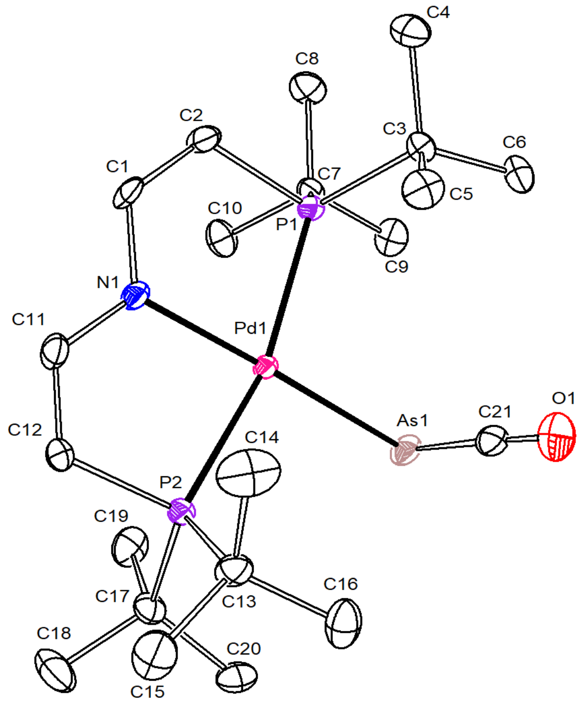

Figure S86: Thermal ellipsoid plot of $\mathbf{1}^{Pd,As}$ with the anisotropic displacement parameters drawn at the 50% probability level. The asymmetric unit contains one complex molecule. The reflection 16 1 8 was removed from the refinement using OMIT command.

Table S25: Crystal data and structure refinement for $\mathbf{1}^{Pd,As}$.

Identification code	mn1_22	(MN04-8)
Empirical formula	$C_{21} H_{40} As N O P_2 Pd$	
Formula weight	565.80	
Temperature	100(2) K	
Wavelength	0.71073 Å	
Crystal system	Orthorhombic	
Space group	Pna21	
Unit cell dimensions	a = 18.606(3) Å	α= 90°.
	b = 16.074(3) Å	β= 90°.
	c = 8.4418(11) Å	γ = 90°.
Volume	2524.7(7) Å^3	
Z	4	
Density (calculated)	1.489 Mg/m^3	
Absorption coefficient	2.173 mm^{-1}	
F(000)	1160	
Crystal size	0.241 x 0.066 x 0.039 mm^3	
Theta range for data collection	2.189 to 27.928°.	
Index ranges	-24<=h<=24, -21<=k<=21, -11<=l<=10	
Reflections collected	42699	
Independent reflections	6000 [R(int) = 0.0639]	
Completeness to theta = 25.242°	100.0 %	
Absorption correction	Semi-empirical from equivalents	
Max. and min. transmission	0.92 and 0.81	
Refinement method	Full-matrix least-squares on F^2	
Data / restraints / parameters	6000 / 1 / 256	
Goodness-of-fit on F2	1.055	
Final R indices [I>2sigma(I)]	R1 = 0.0267, wR2 = 0.0447	
R indices (all data)	R1 = 0.0347, wR2 = 0.0474	
Absolute structure parameter	0.012(6)	
Extinction coefficient	n/a	
Largest diff. peak and hole	0.410 and -0.585 e.Å^{-3}	

Table S26: Bond lengths [Å] and angles [°] for **1**^{Pd,As}.

Pd(1)-N(1)	2.051(3)	C(3)-P(1)-Pd(1)	116.02(14)
Pd(1)-P(1)	2.3343(11)	C(7)-P(1)-Pd(1)	115.94(13)
Pd(1)-P(2)	2.3503(11)	C(12)-P(2)-C(17)	104.90(19)
Pd(1)-As(1)	2.4664(6)	C(12)-P(2)-C(13)	104.63(19)
As(1)-C(21)	1.766(5)	C(17)-P(2)-C(13)	113.3(2)
P(1)-C(2)	1.794(4)	C(12)-P(2)-Pd(1)	99.09(14)
P(1)-C(3)	1.882(4)	C(17)-P(2)-Pd(1)	117.75(14)
P(1)-C(7)	1.883(4)	C(13)-P(2)-Pd(1)	114.56(14)
P(2)-C(12)	1.782(4)	C(1)-C(2)-P(1)	116.2(3)
P(2)-C(17)	1.878(4)	C(11)-N(1)-C(1)	121.5(4)
P(2)-C(13)	1.881(4)	C(11)-N(1)-Pd(1)	119.6(3)
C(2)-C(1)	1.347(6)	C(1)-N(1)-Pd(1)	118.9(3)
O(1)-C(21)	1.172(6)	C(2)-C(1)-N(1)	122.7(4)
N(1)-C(11)	1.359(5)	C(5)-C(3)-C(4)	108.1(3)
N(1)-C(1)	1.369(5)	C(5)-C(3)-C(6)	109.4(4)
C(3)-C(5)	1.532(6)	C(4)-C(3)-C(6)	109.5(4)
C(3)-C(4)	1.538(6)	C(5)-C(3)-P(1)	105.0(3)
C(3)-C(6)	1.540(6)	C(4)-C(3)-P(1)	112.1(3)
C(15)-C(13)	1.538(6)	C(6)-C(3)-P(1)	112.5(3)
C(7)-C(9)	1.531(6)	C(9)-C(7)-C(10)	108.2(3)
C(7)-C(10)	1.534(5)	C(9)-C(7)-C(8)	108.7(3)
C(7)-C(8)	1.539(6)	C(10)-C(7)-C(8)	108.8(3)
C(16)-C(13)	1.536(6)	C(9)-C(7)-P(1)	114.1(3)
C(17)-C(20)	1.531(6)	C(10)-C(7)-P(1)	104.8(3)
C(17)-C(18)	1.534(6)	C(8)-C(7)-P(1)	112.1(3)
C(17)-C(19)	1.543(6)	C(20)-C(17)-C(18)	109.9(4)
C(11)-C(12)	1.344(5)	C(20)-C(17)-C(19)	109.2(4)
C(13)-C(14)	1.528(7)	C(18)-C(17)-C(19)	108.1(4)
N(1)-Pd(1)-P(1)	83.14(10)	C(18)-C(17)-P(2)	113.3(3)
N(1)-Pd(1)-P(2)	82.17(10)	C(19)-C(17)-P(2)	104.2(3)
P(1)-Pd(1)-P(2)	165.23(4)	C(12)-C(11)-N(1)	122.6(4)
N(1)-Pd(1)-As(1)	173.09(10)	C(11)-C(12)-P(2)	116.3(3)
P(1)-Pd(1)-As(1)	96.33(3)	C(14)-C(13)-C(16)	108.4(4)
P(2)-Pd(1)-As(1)	98.44(3)	C(14)-C(13)-C(15)	109.6(4)
C(21)-As(1)-Pd(1)	95.87(15)	C(16)-C(13)-C(15)	108.8(4)
C(2)-P(1)-C(3)	104.95(19)	C(14)-C(13)-P(2)	105.0(3)
C(2)-P(1)-C(7)	103.96(19)	C(16)-C(13)-P(2)	112.8(3)
C(3)-P(1)-C(7)	114.16(18)	C(15)-C(13)-P(2)	112.0(3)
C(2)-P(1)-Pd(1)	98.95(14)	O(1)-C(21)-As(1)	176.7(4)

Symmetry transformations used to generate equivalent atoms:

Table S27: Torsion angles [°] for **1**^{Pd,As}.

C(3)-P(1)-C(2)-C(1)	-122.9(4)	C(12)-P(2)-C(17)-C(20)	-167.0(3)
C(7)-P(1)-C(2)-C(1)	116.9(4)	C(13)-P(2)-C(17)-C(20)	-53.5(4)
Pd(1)-P(1)-C(2)-C(1)	-2.8(4)	Pd(1)-P(2)-C(17)-C(20)	84.0(3)
P(1)-C(2)-C(1)-N(1)	2.2(6)	C(12)-P(2)-C(17)-C(18)	-42.1(4)
C(11)-N(1)-C(1)-C(2)	-179.0(4)	C(13)-P(2)-C(17)-C(18)	71.4(4)
Pd(1)-N(1)-C(1)-C(2)	0.0(6)	Pd(1)-P(2)-C(17)-C(18)	-151.1(3)
C(2)-P(1)-C(3)-C(5)	78.7(3)	C(12)-P(2)-C(17)-C(19)	75.1(3)
C(7)-P(1)-C(3)-C(5)	-168.2(3)	C(13)-P(2)-C(17)-C(19)	-171.4(3)
Pd(1)-P(1)-C(3)-C(5)	-29.4(3)	Pd(1)-P(2)-C(17)-C(19)	-33.8(3)
C(2)-P(1)-C(3)-C(4)	-38.5(4)	C(1)-N(1)-C(11)-C(12)	-178.8(4)
C(7)-P(1)-C(3)-C(4)	74.7(3)	Pd(1)-N(1)-C(11)-C(12)	2.3(6)
Pd(1)-P(1)-C(3)-C(4)	-146.5(3)	N(1)-C(11)-C(12)-P(2)	2.1(6)
C(2)-P(1)-C(3)-C(6)	-162.5(3)	C(17)-P(2)-C(12)-C(11)	-126.4(4)
C(7)-P(1)-C(3)-C(6)	-49.3(4)	C(13)-P(2)-C(12)-C(11)	114.1(4)
Pd(1)-P(1)-C(3)-C(6)	89.5(3)	Pd(1)-P(2)-C(12)-C(11)	-4.4(4)
C(2)-P(1)-C(7)-C(9)	178.0(3)	C(12)-P(2)-C(13)-C(14)	-61.2(4)
C(3)-P(1)-C(7)-C(9)	64.3(3)	C(17)-P(2)-C(13)-C(14)	-174.9(3)
Pd(1)-P(1)-C(7)-C(9)	-74.6(3)	Pd(1)-P(2)-C(13)-C(14)	46.2(3)
C(2)-P(1)-C(7)-C(10)	-63.8(3)	C(12)-P(2)-C(13)-C(16)	-179.1(3)
C(3)-P(1)-C(7)-C(10)	-177.5(3)	C(17)-P(2)-C(13)-C(16)	67.2(4)

Pd(1)-P(1)-C(7)-C(10)	43.6(3)	Pd(1)-P(2)-C(13)-C(16)	-71.7(3)
C(2)-P(1)-C(7)-C(8)	54.0(3)	C(12)-P(2)-C(13)-C(15)	57.7(4)
C(3)-P(1)-C(7)-C(8)	-59.8(3)	C(17)-P(2)-C(13)-C(15)	-56.0(4)
Pd(1)-P(1)-C(7)-C(8)	161.4(2)	Pd(1)-P(2)-C(13)-C(15)	165.0(3)

Symmetry transformations used to generate equivalent atoms:

X-ray Single-Crystal Structure Analysis of [Pt(AsCO)(PNP)] ($1^{Pt,As}$)

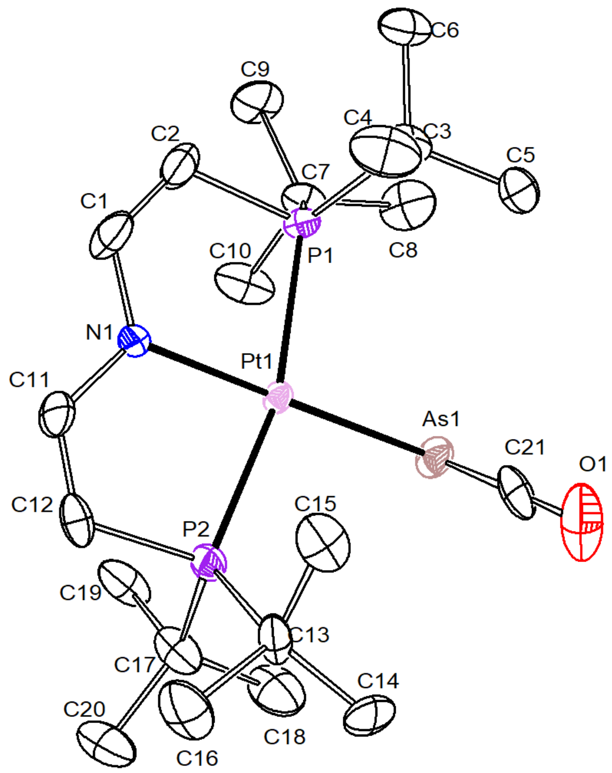

Figure S87: Thermal ellipsoid plot of $\mathbf{1}^{Pt,As}$ with the anisotropic displacement parameters drawn at the 50% probability level. The asymmetric unit contains one complex molecule.

Table S28: Crystal data and structure refinement for $\mathbf{1}^{Pt,As}$.

Identification code	MN_120122_MO	(MN03_17)
Empirical formula	C_{21} H_{40} As N O P_2 Pt	
Formula weight	654.49	
Temperature	100(2) K	
Wavelength	0.71073 Å	
Crystal system	Orthorhombic	
Space group	Pbca	
Unit cell dimensions	a = 15.2319(18) Å	α= 90°
	b = 14.0047(17) Å	β= 90°
	c = 23.452(3) Å	γ = 90°
Volume	5002.7(10) Å^3	
Z	8	
Density (calculated)	1.738 Mg/m^3	
Absorption coefficient	7.062 mm^{-1}	
F(000)	2576	
Crystal size	0.309 x 0.276 x 0.037 mm3	
Crystal shape and color	Plate,	clear intense pink
Theta range for data collection	2.158 to 25.514°.	
Index ranges	-18<=h<=18, -16<=k<=16, -28<=l<=28	
Reflections collected	37824	
Independent reflections	4594 [R(int) = 0.0298]	
Completeness to theta = 25.242°	98.9 %	
Refinement method	Full-matrix least-squares on F2	
Data / restraints / parameters	4594 / 0 / 256	
Goodness-of-fit on F2	1.081	
Final R indices [I>2sigma(I)]	R1 = 0.0522,	wR2 = 0.1059
R indices (all data)	R1 = 0.0556,	wR2 = 0.1084
Extinction coefficient	n/a	
Largest diff. peak and hole	2.455 and -1.559 eÅ^{-3}	

Table S29: Bond lengths [Å] and angles [°] for **1**^{Pt,As}.

Pt(1)-N(1)	2.043(7)	C(3)-P(1)-Pt(1)	116.5(3)
Pt(1)-P(1)	2.314(2)	C(12)-P(2)-C(17)	104.8(5)
Pt(1)-P(2)	2.321(2)	C(12)-P(2)-C(13)	104.1(5)
Pt(1)-As(1)	2.4550(10)	C(17)-P(2)-C(13)	113.2(5)
As(1)-C(21)	1.739(11)	C(12)-P(2)-Pt(1)	99.3(4)
P(1)-C(2)	1.794(10)	C(17)-P(2)-Pt(1)	115.6(3)
P(1)-C(7)	1.871(9)	C(13)-P(2)-Pt(1)	117.1(3)
P(1)-C(3)	1.876(9)	C(1)-N(1)-C(11)	121.7(8)
P(2)-C(12)	1.784(10)	C(1)-N(1)-Pt(1)	119.3(7)
P(2)-C(17)	1.880(11)	C(11)-N(1)-Pt(1)	118.7(6)
P(2)-C(13)	1.891(10)	C(2)-C(1)-N(1)	123.8(9)
O(1)-C(21)	1.185(14)	C(1)-C(2)-P(1)	114.5(8)
N(1)-C(1)	1.346(13)	C(4)-C(3)-C(6)	109.8(9)
N(1)-C(11)	1.382(12)	C(4)-C(3)-C(5)	108.0(9)
C(1)-C(2)	1.338(16)	C(6)-C(3)-C(5)	109.4(9)
C(3)-C(4)	1.528(15)	C(4)-C(3)-P(1)	106.2(7)
C(3)-C(6)	1.533(14)	C(6)-C(3)-P(1)	111.4(7)
C(3)-C(5)	1.533(14)	C(5)-C(3)-P(1)	111.8(7)
C(7)-C(10)	1.526(14)	C(10)-C(7)-C(9)	108.8(9)
C(7)-C(9)	1.527(13)	C(10)-C(7)-C(8)	107.7(9)
C(7)-C(8)	1.533(15)	C(9)-C(7)-C(8)	108.7(9)
C(11)-C(12)	1.314(15)	C(10)-C(7)-P(1)	106.5(7)
C(13)-C(14)	1.520(15)	C(9)-C(7)-P(1)	112.7(7)
C(13)-C(16)	1.535(14)	C(8)-C(7)-P(1)	112.2(7)
C(13)-C(15)	1.536(16)	C(12)-C(11)-N(1)	122.6(9)
C(17)-C(19)	1.499(16)	C(11)-C(12)-P(2)	116.7(8)
C(17)-C(20)	1.527(14)	C(14)-C(13)-C(16)	109.8(9)
C(17)-C(18)	1.540(16)	C(14)-C(13)-C(15)	110.5(9)
N(1)-Pt(1)-P(1)	82.4(2)	C(16)-C(13)-C(15)	108.0(9)
N(1)-Pt(1)-P(2)	82.7(2)	C(14)-C(13)-P(2)	113.2(8)
P(1)-Pt(1)-P(2)	165.04(8)	C(16)-C(13)-P(2)	111.4(7)
N(1)-Pt(1)-As(1)	173.8(2)	C(15)-C(13)-P(2)	103.7(7)
P(1)-Pt(1)-As(1)	95.13(6)	C(19)-C(17)-C(20)	109.2(10)
P(2)-Pt(1)-As(1)	99.50(7)	C(19)-C(17)-C(18)	108.1(10)
C(21)-As(1)-Pt(1)	99.0(3)	C(20)-C(17)-C(18)	109.7(10)
C(2)-P(1)-C(7)	105.4(5)	C(19)-C(17)-P(2)	105.4(8)
C(2)-P(1)-C(3)	103.7(5)	C(20)-C(17)-P(2)	111.8(7)
C(7)-P(1)-C(3)	112.6(4)	C(18)-C(17)-P(2)	112.3(8)
C(2)-P(1)-Pt(1)	99.9(4)	O(1)-C(21)-As(1)	174.4(10)
C(7)-P(1)-Pt(1)	116.3(3)		

Symmetry transformations used to generate equivalent atoms:

Table S30: Torsion angles [°] for **1**^{Pt,As}.

C(11)-N(1)-C(1)-C(2)	-175.9(11)	C(1)-N(1)-C(11)-C(12)	177.8(11)
Pt(1)-N(1)-C(1)-C(2)	-1.7(15)	Pt(1)-N(1)-C(11)-C(12)	3.6(15)
N(1)-C(1)-C(2)-P(1)	0.9(16)	N(1)-C(11)-C(12)-P(2)	-2.0(16)
C(7)-P(1)-C(2)-C(1)	121.1(9)	C(17)-P(2)-C(12)-C(11)	-119.9(9)
C(3)-P(1)-C(2)-C(1)	-120.4(10)	C(13)-P(2)-C(12)-C(11)	121.0(10)
Pt(1)-P(1)-C(2)-C(1)	0.1(10)	Pt(1)-P(2)-C(12)-C(11)	-0.1(10)
C(2)-P(1)-C(3)-C(4)	55.7(9)	C(12)-P(2)-C(13)-C(14)	160.1(8)
C(7)-P(1)-C(3)-C(4)	169.0(8)	C(17)-P(2)-C(13)-C(14)	46.9(9)
Pt(1)-P(1)-C(3)-C(4)	-52.9(8)	Pt(1)-P(2)-C(13)-C(14)	-91.5(8)
C(2)-P(1)-C(3)-C(6)	-63.9(8)	C(12)-P(2)-C(13)-C(16)	35.9(10)
C(7)-P(1)-C(3)-C(6)	49.5(9)	C(17)-P(2)-C(13)-C(16)	-77.3(10)
Pt(1)-P(1)-C(3)-C(6)	-172.5(6)	Pt(1)-P(2)-C(13)-C(16)	144.3(7)
C(2)-P(1)-C(3)-C(5)	173.3(7)	C(12)-P(2)-C(13)-C(15)	-80.0(8)
C(7)-P(1)-C(3)-C(5)	-73.3(8)	C(17)-P(2)-C(13)-C(15)	166.8(7)
Pt(1)-P(1)-C(3)-C(5)	64.7(8)	Pt(1)-P(2)-C(13)-C(15)	28.4(8)
C(2)-P(1)-C(7)-C(10)	-83.8(8)	C(12)-P(2)-C(17)-C(19)	62.2(8)
C(3)-P(1)-C(7)-C(10)	163.9(7)	C(13)-P(2)-C(17)-C(19)	175.0(7)
Pt(1)-P(1)-C(7)-C(10)	25.8(8)	Pt(1)-P(2)-C(17)-C(19)	-45.9(8)
C(2)-P(1)-C(7)-C(9)	35.4(9)	C(12)-P(2)-C(17)-C(20)	-56.4(10)

C(3)-P(1)-C(7)-C(9)	-76.9(9)	C(13)-P(2)-C(17)-C(20)	56.4(10)
Pt(1)-P(1)-C(7)-C(9)	145.0(7)	Pt(1)-P(2)-C(17)-C(20)	-164.5(8)
C(2)-P(1)-C(7)-C(8)	158.5(8)	C(12)-P(2)-C(17)-C(18)	179.7(8)
C(3)-P(1)-C(7)-C(8)	46.2(9)	C(13)-P(2)-C(17)-C(18)	-67.5(9)
Pt(1)-P(1)-C(7)-C(8)	-91.9(8)	Pt(1)-P(2)-C(17)-C(18)	71.6(9)

Symmetry transformations used to generate equivalent atoms:

X-ray Single-Crystal Structure Analysis of [Pt(BiMe$_2$)(PNP)] (1^{Pt,Bi})

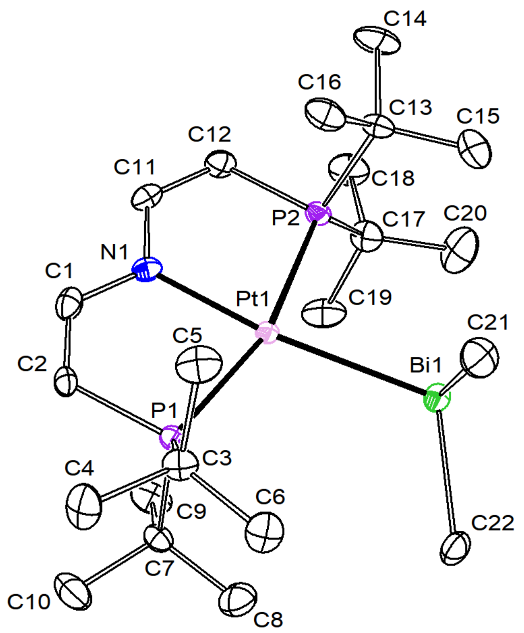

Figure S88: Thermal ellipsoid plot of **1^{Pt,Bi}** with the anisotropic displacement parameters drawn at the 50% probability level. The asymmetric unit contains one complex molecule. The reflections 0 1 0, 0 -1 1, -1 0 1 and -1 1 1 were removed from the refinement using OMIT command.

Table S31: Crystal data and structure refinement for **1^{Pt,Bi}**.

Identification code	mo_MN_240129_MN_05	
Empirical formula	$C_{22} H_{46}$ Bi N P_2 Pt	
Formula weight	790.61	
Temperature	100(2) K	
Wavelength	0.71073 Å	
Crystal system	Triclinic	
Space group	P-1	
Unit cell dimensions	a = 9.1002(2) Å	α= 107.1390(10)°.
	b = 12.0516(3) Å	β= 97.7300(10)°.
	c = 14.1785(4) Å	γ = 111.0660(10)°.
Volume	1335.27(6) Å^3	
Z	2	
Density (calculated)	1.966 Mg/m^3	
Absorption coefficient	11.941 mm^{-1}	
F(000)	752	
Crystal size	0.140 x 0.097 x 0.076 mm^3	
Theta range for data collection	2.43 to 27.55°.	
Index ranges	-11<=h<=11, -15<=k<=15, -18<=l<=18	
Reflections collected	63856	
Independent reflections	6150 [R(int) = 0.0874]	
Completeness to theta = 25.24°	99.7 %	
Absorption correction	Semi-empirical from equivalents	
Max. and min. transmission	0.745600 and 0.591400	
Refinement method	Full-matrix least-squares on F^2	
Data / restraints / parameters	6150 / 0 / 258	
Goodness-of-fit on F^2	1.041	
Final R indices [I>2sigma(I)]	R1 = 0.0254, wR2 = 0.0518	
R indices (all data)	R1 = 0.0361, wR2 = 0.0568	

Table S32: Bond lengths [Å] and angles [°] for **1**[Pt,Bi].

Bi(1)-C(21)	2.279(6)	C(2)-P(1)-Pt(1)	100.58(17)
Bi(1)-C(22)	2.295(5)	C(7)-P(1)-Pt(1)	114.40(16)
Bi(1)-Pt(1)	2.7323(3)	C(3)-P(1)-Pt(1)	117.91(18)
Pt(1)-N(1)	2.093(4)	C(12)-P(2)-C(17)	103.8(3)
Pt(1)-P(1)	2.3046(12)	C(12)-P(2)-C(13)	103.1(2)
Pt(1)-P(2)	2.3232(13)	C(17)-P(2)-C(13)	113.7(3)
P(1)-C(2)	1.791(5)	C(12)-P(2)-Pt(1)	100.61(18)
P(1)-C(7)	1.883(5)	C(17)-P(2)-Pt(1)	117.42(18)
P(1)-C(3)	1.884(5)	C(13)-P(2)-Pt(1)	115.20(17)
P(2)-C(12)	1.777(5)	C(1)-N(1)-C(11)	120.8(5)
P(2)-C(17)	1.880(6)	C(1)-N(1)-Pt(1)	119.5(3)
P(2)-C(13)	1.883(5)	C(11)-N(1)-Pt(1)	119.7(3)
N(1)-C(1)	1.354(7)	C(2)-C(1)-N(1)	121.9(5)
N(1)-C(11)	1.365(6)	C(1)-C(2)-P(1)	116.1(4)
C(1)-C(2)	1.336(7)	C(6)-C(3)-C(4)	109.6(4)
C(3)-C(6)	1.522(7)	C(6)-C(3)-C(5)	109.3(5)
C(3)-C(4)	1.535(7)	C(4)-C(3)-C(5)	108.5(5)
C(3)-C(5)	1.537(8)	C(6)-C(3)-P(1)	112.4(4)
C(7)-C(8)	1.517(8)	C(4)-C(3)-P(1)	113.0(4)
C(7)-C(10)	1.524(7)	C(5)-C(3)-P(1)	103.8(3)
C(7)-C(9)	1.541(7)	C(8)-C(7)-C(10)	109.8(5)
C(11)-C(12)	1.353(7)	C(8)-C(7)-C(9)	107.8(5)
C(13)-C(15)	1.514(8)	C(10)-C(7)-C(9)	108.4(5)
C(13)-C(16)	1.523(8)	C(8)-C(7)-P(1)	113.2(4)
C(13)-C(14)	1.525(7)	C(10)-C(7)-P(1)	113.0(4)
C(17)-C(20)	1.522(8)	C(9)-C(7)-P(1)	104.4(3)
C(17)-C(19)	1.534(8)	C(12)-C(11)-N(1)	121.3(5)
C(17)-C(18)	1.544(8)	C(11)-C(12)-P(2)	116.6(4)
C(21)-Bi(1)-C(22)	91.7(2)	C(15)-C(13)-C(16)	108.8(5)
C(21)-Bi(1)-Pt(1)	99.69(15)	C(15)-C(13)-C(14)	108.5(5)
C(22)-Bi(1)-Pt(1)	117.53(14)	C(16)-C(13)-C(14)	109.1(5)
N(1)-Pt(1)-P(1)	81.58(12)	C(15)-C(13)-P(2)	113.7(4)
N(1)-Pt(1)-P(2)	81.53(12)	C(16)-C(13)-P(2)	104.3(4)
P(1)-Pt(1)-P(2)	163.07(5)	C(14)-C(13)-P(2)	112.1(4)
N(1)-Pt(1)-Bi(1)	172.47(11)	C(20)-C(17)-C(19)	109.4(5)
P(1)-Pt(1)-Bi(1)	105.94(3)	C(20)-C(17)-C(18)	107.9(5)
P(2)-Pt(1)-Bi(1)	90.95(3)	C(19)-C(17)-C(18)	108.0(5)
C(2)-P(1)-C(7)	102.9(2)	C(20)-C(17)-P(2)	114.2(4)
C(2)-P(1)-C(3)	103.1(2)	C(19)-C(17)-P(2)	104.6(4)
C(7)-P(1)-C(3)	114.7(2)	C(18)-C(17)-P(2)	112.6(4)

Table S33: Torsion angles [°] for **1**[Pt,Bi].

C(11)-N(1)-C(1)-C(2)	176.3(5)	C(1)-N(1)-C(11)-C(12)	179.0(5)
Pt(1)-N(1)-C(1)-C(2)	-2.6(7)	Pt(1)-N(1)-C(11)-C(12)	-2.2(7)
N(1)-C(1)-C(2)-P(1)	-1.7(7)	N(1)-C(11)-C(12)-P(2)	-2.5(7)
C(7)-P(1)-C(2)-C(1)	-114.0(4)	C(17)-P(2)-C(12)-C(11)	126.9(4)
C(3)-P(1)-C(2)-C(1)	126.4(4)	C(13)-P(2)-C(12)-C(11)	-114.3(4)
Pt(1)-P(1)-C(2)-C(1)	4.3(4)	Pt(1)-P(2)-C(12)-C(11)	5.0(4)
C(2)-P(1)-C(3)-C(6)	170.0(4)	C(12)-P(2)-C(13)-C(15)	-173.0(4)
C(7)-P(1)-C(3)-C(6)	58.9(5)	C(17)-P(2)-C(13)-C(15)	-61.3(5)
Pt(1)-P(1)-C(3)-C(6)	-80.4(4)	Pt(1)-P(2)-C(13)-C(15)	78.4(5)
C(2)-P(1)-C(3)-C(4)	45.2(5)	C(12)-P(2)-C(13)-C(16)	68.6(4)
C(7)-P(1)-C(3)-C(4)	-65.8(5)	C(17)-P(2)-C(13)-C(16)	-179.7(4)
Pt(1)-P(1)-C(3)-C(4)	154.9(3)	Pt(1)-P(2)-C(13)-C(16)	-40.0(4)
C(2)-P(1)-C(3)-C(5)	-72.1(4)	C(12)-P(2)-C(13)-C(14)	-49.4(5)
C(7)-P(1)-C(3)-C(5)	176.9(3)	C(17)-P(2)-C(13)-C(14)	62.3(5)
Pt(1)-P(1)-C(3)-C(5)	37.6(4)	Pt(1)-P(2)-C(13)-C(14)	-158.0(4)
C(2)-P(1)-C(7)-C(8)	-176.8(4)	C(12)-P(2)-C(17)-C(20)	159.9(5)
C(3)-P(1)-C(7)-C(8)	-65.6(4)	C(13)-P(2)-C(17)-C(20)	48.6(5)
Pt(1)-P(1)-C(7)-C(8)	75.1(4)	Pt(1)-P(2)-C(17)-C(20)	-90.2(5)
C(2)-P(1)-C(7)-C(10)	-51.2(5)	C(12)-P(2)-C(17)-C(19)	-80.6(4)
C(3)-P(1)-C(7)-C(10)	59.9(5)	C(13)-P(2)-C(17)-C(19)	168.2(4)
Pt(1)-P(1)-C(7)-C(10)	-159.3(4)	Pt(1)-P(2)-C(17)-C(19)	29.4(4)
C(2)-P(1)-C(7)-C(9)	66.3(4)	C(12)-P(2)-C(17)-C(18)	36.4(5)
C(3)-P(1)-C(7)-C(9)	177.4(4)	C(13)-P(2)-C(17)-C(18)	-74.9(5)
Pt(1)-P(1)-C(7)-C(9)	-41.8(4)	Pt(1)-P(2)-C(17)-C(18)	146.3(4)

Symmetry transformations used to generate equivalent atoms:

X-ray Single-Crystal Structure Analysis of [(μ-P_2){Pd(PNP)}$_2$] ($2^{Pd,P}$)

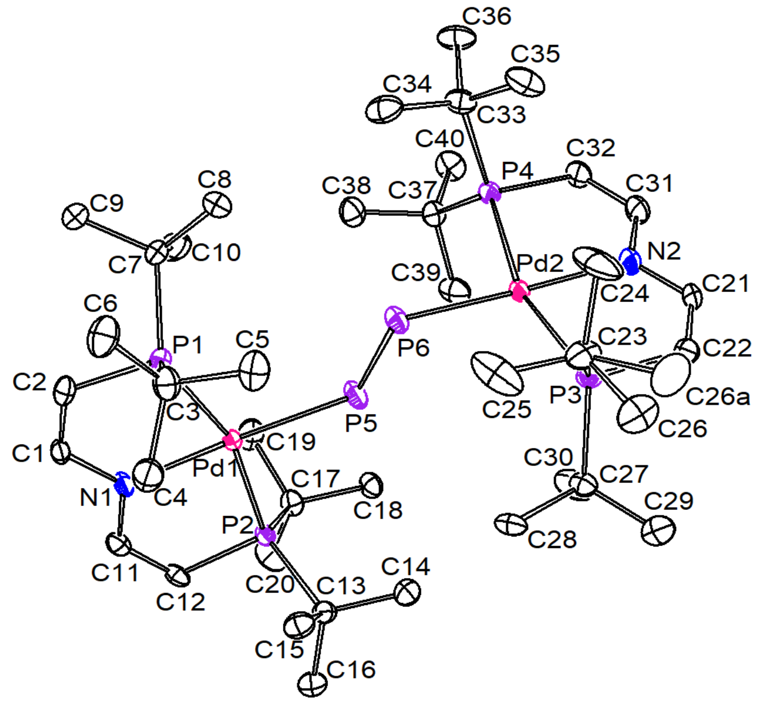

Figure S89: Thermal ellipsoid plot of $2^{Pd,P}$ with the anisotropic displacement parameters drawn at the 50% probability level. The asymmetric unit contains one disordered complex molecule. The disordered complex molecule was refined with population of 0.63 (7) on the main domain using some restraints and constraints (RIGU, SIMU, SADI). The reflections 1 1 0 and 1 0 1 are removed from the refinement using OMIT commands.

Table S34: Crystal data and structure refinement for $2^{Pd,P}$.

Identification code	mo_MN_251022_2_MO_0m_a	
Empirical formula	$C_{40}H_{80}N_2P_6Pd_2$	
Formula weight	987.68	
Temperature	100(2) K	
Wavelength	0.71073 Å	
Crystal system	Monoclinic	
Space group	$P2_1/n$	
Unit cell dimensions	a = 18.9998(12) Å	α= 90°
	b = 13.4430(10) Å	β= 110.336(2)°
	c = 19.7276(12) Å	γ = 90°
Volume	4724.7(5) Å^3	
Z	4	
Density (calculated)	1.389 Mg/m^3	
Absorption coefficient	0.993 mm^{-1}	
F(000)	2064	
Crystal size	0.068 x 0.106 x 0.117 mm^3	
Crystal shape and color	Plate,	clear pale red / black
Theta range for data collection	1.873 to 26.397°	
Index ranges	-23<=h<=23, -16<=k<=16, -21<=l<=24	
Reflections collected	99876	
Independent reflections	9666 [R(int) = 0.0776]	
Completeness to theta = 25.242°	99.9 %	
Refinement method	Full-matrix least-squares on F^2	
Data / restraints / parameters	9666 / 19 / 486	
Goodness-of-fit on F^2	1.172	
Final R indices [I>2sigma(I)]	R1 = 0.0502,	wR2 = 0.0940
R indices (all data)	R1 = 0.0657,	wR2 = 0.1039
Largest diff. peak and hole	0.640 and -0.684 eÅ^{-3}	

Table S35: Bond lengths [Å] and angles [°] for **$2^{Pd,P}$**.

Pd(1)-N(1)	2.107(4)	C(1)-N(1)-C(11)	121.4(4)
Pd(1)-P(5)	2.3303(13)	C(1)-N(1)-Pd(1)	119.7(3)
Pd(1)-P(1)	2.3511(12)	C(11)-N(1)-Pd(1)	119.0(3)
Pd(1)-P(2)	2.3608(12)	C(2)-P(1)-C(7)	102.8(2)
C(1)-C(2)	1.351(7)	C(2)-P(1)-C(3)	103.3(2)
C(1)-N(1)	1.358(6)	C(7)-P(1)-C(3)	112.3(2)
N(1)-C(11)	1.359(6)	C(2)-P(1)-Pd(1)	99.58(16)
P(1)-C(2)	1.788(5)	C(7)-P(1)-Pd(1)	120.62(16)
P(1)-C(7)	1.877(5)	C(3)-P(1)-Pd(1)	114.81(17)
P(1)-C(3)	1.880(5)	N(2)-Pd(2)-P(6)	177.14(13)
Pd(2)-N(2)	2.103(4)	N(2)-Pd(2)-P(4)	81.40(12)
Pd(2)-P(6)	2.3005(12)	P(6)-Pd(2)-P(4)	99.11(5)
Pd(2)-P(4)	2.3410(12)	N(2)-Pd(2)-P(3)	82.03(12)
Pd(2)-P(3)	2.3467(12)	P(6)-Pd(2)-P(3)	97.20(5)
N(2)-C(31)	1.356(6)	P(4)-Pd(2)-P(3)	162.71(4)
N(2)-C(21)	1.357(6)	C(1)-C(2)-P(1)	117.2(4)
P(2)-C(12)	1.784(5)	C(31)-N(2)-C(21)	121.8(4)
P(2)-C(13)	1.874(5)	C(31)-N(2)-Pd(2)	119.4(3)
P(2)-C(17)	1.887(5)	C(21)-N(2)-Pd(2)	118.8(3)
P(4)-C(32)	1.779(5)	C(12)-P(2)-C(13)	104.9(2)
P(4)-C(37)	1.870(5)	C(12)-P(2)-C(17)	103.9(2)
P(4)-C(33)	1.888(5)	C(13)-P(2)-C(17)	114.2(2)
C(4)-C(3)	1.537(7)	C(12)-P(2)-Pd(1)	99.71(15)
P(3)-C(22)	1.794(5)	C(13)-P(2)-Pd(1)	115.30(15)
P(3)-C(27)	1.871(5)	C(17)-P(2)-Pd(1)	116.11(16)
P(3)-C(23)	1.880(5)	C(32)-P(4)-C(37)	103.8(2)
C(3)-C(6)	1.526(7)	C(32)-P(4)-C(33)	104.7(2)
C(3)-C(5)	1.535(7)	C(37)-P(4)-C(33)	113.0(2)
P(5)-P(6)	2.0155(18)	C(32)-P(4)-Pd(2)	100.03(16)
C(9)-C(7)	1.535(6)	C(37)-P(4)-Pd(2)	119.47(16)
C(8)-C(7)	1.529(7)	C(33)-P(4)-Pd(2)	113.21(15)
C(7)-C(10)	1.535(7)	C(22)-P(3)-C(27)	103.7(2)
C(11)-C(12)	1.336(7)	C(22)-P(3)-C(23)	105.6(2)
C(14)-C(13)	1.526(6)	C(27)-P(3)-C(23)	112.4(2)
C(13)-C(15)	1.527(6)	C(22)-P(3)-Pd(2)	99.33(17)
C(13)-C(16)	1.545(6)	C(27)-P(3)-Pd(2)	119.64(16)
C(18)-C(17)	1.534(6)	C(23)-P(3)-Pd(2)	113.61(17)
C(17)-C(19)	1.532(7)	C(6)-C(3)-C(5)	110.4(4)
C(17)-C(20)	1.535(7)	C(6)-C(3)-C(4)	108.4(4)
C(21)-C(22)	1.339(7)	C(5)-C(3)-C(4)	107.0(4)
C(23)-C(24)	1.503(7)	C(6)-C(3)-P(1)	113.6(4)
C(23)-C(25)	1.532(8)	C(5)-C(3)-P(1)	111.6(3)
C(23)-C(26)	1.548(9)	C(4)-C(3)-P(1)	105.4(4)
C(23)-C(26A)	1.561(14)	P(6)-P(5)-Pd(1)	119.95(7)
C(27)-C(28)	1.524(6)	P(5)-P(6)-Pd(2)	107.00(6)
C(27)-C(29)	1.525(7)	C(8)-C(7)-C(9)	108.1(4)
C(27)-C(30)	1.539(7)	C(8)-C(7)-C(10)	109.8(4)
C(31)-C(32)	1.353(7)	C(9)-C(7)-C(10)	107.7(4)
C(33)-C(34)	1.528(7)	C(8)-C(7)-P(1)	112.7(3)
C(33)-C(36)	1.533(6)	C(9)-C(7)-P(1)	112.8(3)
C(33)-C(35)	1.534(7)	C(10)-C(7)-P(1)	105.6(3)
C(37)-C(39)	1.527(7)	C(12)-C(11)-N(1)	123.0(4)
C(37)-C(38)	1.533(7)	C(14)-C(13)-C(15)	108.9(4)
C(37)-C(40)	1.536(7)	C(14)-C(13)-C(16)	109.0(4)
N(1)-Pd(1)-P(5)	168.69(11)	C(15)-C(13)-C(16)	109.1(4)
N(1)-Pd(1)-P(1)	81.41(11)	C(14)-C(13)-P(2)	112.6(3)
P(5)-Pd(1)-P(1)	107.48(4)	C(15)-C(13)-P(2)	104.8(3)
N(1)-Pd(1)-P(2)	81.33(11)	C(16)-C(13)-P(2)	112.5(3)
P(5)-Pd(1)-P(2)	90.02(4)	C(11)-C(12)-P(2)	116.8(4)
P(1)-Pd(1)-P(2)	162.48(4)	C(19)-C(17)-C(18)	107.6(4)
C(2)-C(1)-N(1)	121.6(4)	C(19)-C(17)-C(20)	109.8(4)
C(18)-C(17)-C(20)	109.7(4)	C(28)-C(27)-P(3)	112.3(3)
C(19)-C(17)-P(2)	104.2(3)	C(29)-C(27)-P(3)	113.0(4)

C(18)-C(17)-P(2)	113.8(3)	C(30)-C(27)-P(3)	104.2(3)
C(20)-C(17)-P(2)	111.5(3)	C(32)-C(31)-N(2)	121.8(4)
C(22)-C(21)-N(2)	122.7(4)	C(31)-C(32)-P(4)	116.8(4)
C(21)-C(22)-P(3)	117.1(4)	C(34)-C(33)-C(36)	110.3(4)
C(24)-C(23)-C(25)	107.9(5)	C(34)-C(33)-C(35)	108.9(4)
C(24)-C(23)-C(26)	118.0(7)	C(36)-C(33)-C(35)	108.2(4)
C(25)-C(23)-C(26)	99.4(7)	C(34)-C(33)-P(4)	111.5(3)
C(24)-C(23)-C(26A)	91.9(11)	C(36)-C(33)-P(4)	112.4(3)
C(25)-C(23)-C(26A)	127.3(11)	C(35)-C(33)-P(4)	105.3(4)
C(24)-C(23)-P(3)	105.1(4)	C(39)-C(37)-C(38)	108.6(4)
C(25)-C(23)-P(3)	112.7(4)	C(39)-C(37)-C(40)	108.5(4)
C(26)-C(23)-P(3)	113.7(5)	C(38)-C(37)-C(40)	109.8(4)
C(26A)-C(23)-P(3)	107.9(8)	C(39)-C(37)-P(4)	104.5(3)
C(28)-C(27)-C(29)	109.4(4)	C(38)-C(37)-P(4)	111.2(3)
C(28)-C(27)-C(30)	108.9(4)	C(40)-C(37)-P(4)	114.1(3)
C(29)-C(27)-C(30)	108.9(5)		

Symmetry transformations used to generate equivalent atoms:

Table S36: Torsion angles [°] for **2^{Pd,P}**.

C(2)-C(1)-N(1)-C(11)	177.3(4)	C(12)-P(2)-C(17)-C(19)	78.7(4)
C(2)-C(1)-N(1)-Pd(1)	-2.9(6)	C(13)-P(2)-C(17)-C(19)	-167.6(3)
N(1)-C(1)-C(2)-P(1)	-3.5(6)	Pd(1)-P(2)-C(17)-C(19)	-29.7(4)
C(7)-P(1)-C(2)-C(1)	131.4(4)	C(12)-P(2)-C(17)-C(18)	-164.4(3)
C(3)-P(1)-C(2)-C(1)	-111.7(4)	C(13)-P(2)-C(17)-C(18)	-50.7(4)
Pd(1)-P(1)-C(2)-C(1)	6.8(4)	Pd(1)-P(2)-C(17)-C(18)	87.2(3)
C(2)-P(1)-C(3)-C(6)	-62.1(4)	C(12)-P(2)-C(17)-C(20)	-39.7(4)
C(7)-P(1)-C(3)-C(6)	47.9(4)	C(13)-P(2)-C(17)-C(20)	73.9(4)
Pd(1)-P(1)-C(3)-C(6)	-169.4(3)	Pd(1)-P(2)-C(17)-C(20)	-148.1(3)
C(2)-P(1)-C(3)-C(5)	172.3(4)	C(31)-N(2)-C(21)-C(22)	-179.9(5)
C(7)-P(1)-C(3)-C(5)	-77.7(4)	Pd(2)-N(2)-C(21)-C(22)	0.2(7)
Pd(1)-P(1)-C(3)-C(5)	65.0(4)	N(2)-C(21)-C(22)-P(3)	1.0(8)
C(2)-P(1)-C(3)-C(4)	56.5(4)	C(27)-P(3)-C(22)-C(21)	122.4(4)
C(7)-P(1)-C(3)-C(4)	166.5(3)	C(23)-P(3)-C(22)-C(21)	-119.3(5)
Pd(1)-P(1)-C(3)-C(4)	-50.8(4)	Pd(2)-P(3)-C(22)-C(21)	-1.4(5)
C(2)-P(1)-C(7)-C(8)	155.2(4)	C(22)-P(3)-C(23)-C(24)	61.8(5)
C(3)-P(1)-C(7)-C(8)	44.9(4)	C(27)-P(3)-C(23)-C(24)	174.2(4)
Pd(1)-P(1)-C(7)-C(8)	-95.4(4)	Pd(2)-P(3)-C(23)-C(24)	-46.0(5)
C(2)-P(1)-C(7)-C(9)	32.5(4)	C(22)-P(3)-C(23)-C(25)	179.0(4)
C(3)-P(1)-C(7)-C(9)	-77.8(4)	C(27)-P(3)-C(23)-C(25)	-68.6(5)
Pd(1)-P(1)-C(7)-C(9)	141.9(3)	Pd(2)-P(3)-C(23)-C(25)	71.2(4)
C(2)-P(1)-C(7)-C(10)	-84.9(4)	C(22)-P(3)-C(23)-C(26)	-68.8(7)
C(3)-P(1)-C(7)-C(10)	164.8(3)	C(27)-P(3)-C(23)-C(26)	43.7(7)
Pd(1)-P(1)-C(7)-C(10)	24.5(4)	Pd(2)-P(3)-C(23)-C(26)	-176.5(7)
C(1)-N(1)-C(11)-C(12)	179.3(4)	C(22)-P(3)-C(23)-C(26A)	-35.2(12)
Pd(1)-N(1)-C(11)-C(12)	-0.5(6)	C(27)-P(3)-C(23)-C(26A)	77.2(12)
C(12)-P(2)-C(13)-C(14)	167.6(3)	Pd(2)-P(3)-C(23)-C(26A)	-143.0(12)
C(17)-P(2)-C(13)-C(14)	54.4(4)	C(22)-P(3)-C(27)-C(28)	166.6(4)
Pd(1)-P(2)-C(13)-C(14)	-83.9(3)	C(23)-P(3)-C(27)-C(28)	53.1(4)
C(12)-P(2)-C(13)-C(15)	-74.3(3)	Pd(2)-P(3)-C(27)-C(28)	-84.0(3)
C(17)-P(2)-C(13)-C(15)	172.6(3)	C(22)-P(3)-C(27)-C(29)	42.4(4)
Pd(1)-P(2)-C(13)-C(15)	34.3(3)	C(23)-P(3)-C(27)-C(29)	-71.2(4)
C(12)-P(2)-C(13)-C(16)	44.0(4)	Pd(2)-P(3)-C(27)-C(29)	151.7(3)
C(17)-P(2)-C(13)-C(16)	-69.1(4)	C(22)-P(3)-C(27)-C(30)	-75.7(4)
Pd(1)-P(2)-C(13)-C(16)	152.6(3)	C(23)-P(3)-C(27)-C(30)	170.8(4)
N(1)-C(11)-C(12)-P(2)	-2.8(6)	Pd(2)-P(3)-C(27)-C(30)	33.7(4)
C(13)-P(2)-C(12)-C(11)	123.6(4)	C(21)-N(2)-C(31)-C(32)	-176.7(5)
C(17)-P(2)-C(12)-C(11)	-116.2(4)	Pd(2)-N(2)-C(31)-C(32)	3.2(7)
Pd(1)-P(2)-C(12)-C(11)	4.0(4)	N(2)-C(31)-C(32)-P(4)	3.1(7)
C(37)-P(4)-C(32)-C(31)	-130.4(4)	Pd(2)-P(4)-C(33)-C(35)	51.8(4)
C(33)-P(4)-C(32)-C(31)	110.9(4)	C(32)-P(4)-C(37)-C(39)	77.7(4)
Pd(2)-P(4)-C(32)-C(31)	-6.5(5)	C(33)-P(4)-C(37)-C(39)	-169.4(3)
C(32)-P(4)-C(33)-C(34)	-174.1(4)	Pd(2)-P(4)-C(37)-C(39)	-32.4(4)
C(37)-P(4)-C(33)-C(34)	73.6(4)	C(32)-P(4)-C(37)-C(38)	-165.3(3)

Pd(2)-P(4)-C(33)-C(34)	-66.2(4)	C(33)-P(4)-C(37)-C(38)	-52.5(4)
C(32)-P(4)-C(33)-C(36)	61.4(4)	Pd(2)-P(4)-C(37)-C(38)	84.5(3)
C(37)-P(4)-C(33)-C(36)	-50.9(4)	C(32)-P(4)-C(37)-C(40)	-40.5(4)
Pd(2)-P(4)-C(33)-C(36)	169.4(3)	C(33)-P(4)-C(37)-C(40)	72.3(4)
C(32)-P(4)-C(33)-C(35)	-56.2(4)	Pd(2)-P(4)-C(37)-C(40)	-150.7(3)
C(37)-P(4)-C(33)-C(35)	-168.4(3)		

Symmetry transformations used to generate equivalent atoms:

X-ray Single-Crystal Structure Analysis of [(μ-As$_2$){Pd(PNP)}$_2$] (2^{Pd,As})

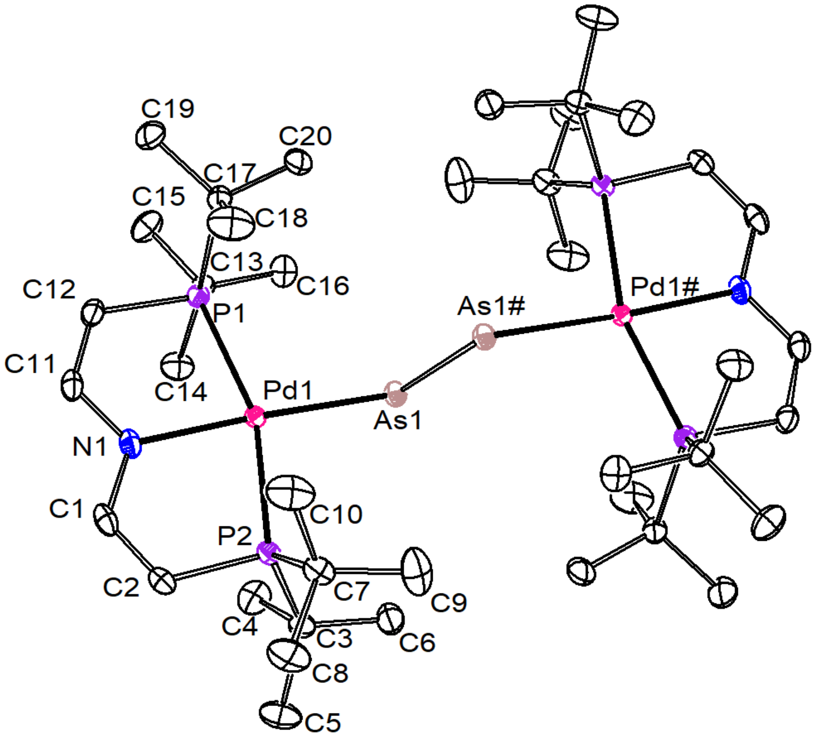

Figure S90: Thermal ellipsoid plot of **2^{Pd,As}**with the anisotropic displacement parameters drawn at the 50% probability level. The asymmetric unit contains only a half complex molecule.

Table S37: Crystal data and structure refinement for **2^{Pd,As}**.

Identification code	mo_MN_221026_2_MN_0m_a	
Empirical formula	C$_{40}$ H$_{80}$ As$_2$ N$_2$ P$_4$ Pd$_2$	
Formula weight	1075.58	
Temperature	100(2) K	
Wavelength	0.71073 Å	
Crystal system	Triclinic	
Space group	P-1	
Unit cell dimensions	a = 8.7508(7) Å	α= 79.043(3)°
	b = 11.9548(9) Å	β= 83.570(3)°
	c = 12.2091(8) Å	γ = 70.253(3)°
Volume	1178.64(15) Å^3	
Z	1	
Density (calculated)	1.515 Mg/m^3	
Absorption coefficient	2.320 mm^{-1}	
F(000)	552	
Crystal size	0.102 x 0.098 x 0.059 mm^3	
Crystal shape and color	Plate,	clear intense red/black
Theta range for data collection	2.29 to 29.19°.	
Index ranges	-11<=h<=12, -16<=k<=16, -16<=l<=16	
Reflections collected	71054	
Independent reflections	6369 [R(int) = 0.1068]	
Completeness to theta = 29.19°	99.9 %	
Absorption correction	Semi-empirical from equivalents	
Max. and min. transmission	0.66 and 0.41	
Refinement method	Full-matrix least-squares on F^2	
Data / restraints / parameters	6369 / 0 / 238	
Goodness-of-fit on F^2	1.054	
Final R indices [I>2sigma(I)]	R1 = 0.0393,	wR2 = 0.0692
R indices (all data)	R1 = 0.0560,	wR2 = 0.0745
Largest diff. peak and hole	1.578 and -0.857 eÅ^{-3}	

Pd(1)-N(1)	2.098(3)
Pd(1)-P(2)	2.3358(9)
Pd(1)-P(1)	2.3405(9)
Pd(1)-As(1)	2.4728(4)
As(1)-As(1)#1	2.2698(7)
P(1)-C(12)	1.782(3)
P(1)-C(17)	1.871(3)
P(1)-C(13)	1.881(3)
N(1)-C(1)	1.356(4)
N(1)-C(11)	1.363(4)
C(1)-C(2)	1.348(5)
P(2)-C(2)	1.782(3)
P(2)-C(3)	1.878(4)
P(2)-C(7)	1.879(3)
C(3)-C(6)	1.513(5)
C(3)-C(5)	1.530(5)
C(3)-C(4)	1.533(5)
C(11)-C(12)	1.341(5)
C(10)-C(7)	1.522(5)
C(17)-C(20)	1.516(5)
C(17)-C(19)	1.526(5)
C(17)-C(18)	1.529(5)
C(16)-C(13)	1.530(5)
C(15)-C(13)	1.528(5)
C(7)-C(9)	1.522(5)
C(7)-C(8)	1.524(5)
C(14)-C(13)	1.535(5)
N(1)-Pd(1)-P(2)	80.98(9)
N(1)-Pd(1)-P(1)	80.64(9)
P(2)-Pd(1)-P(1)	161.49(3)
N(1)-Pd(1)-As(1)	171.91(8)
P(2)-Pd(1)-As(1)	98.72(2)
P(1)-Pd(1)-As(1)	99.16(2)
As(1)#1-As(1)-Pd(1)	115.08(2)
C(12)-P(1)-C(17)	102.97(16)
C(12)-P(1)-C(13)	103.64(16)
C(17)-P(1)-C(13)	114.56(16)
C(12)-P(1)-Pd(1)	100.99(12)
C(17)-P(1)-Pd(1)	118.02(11)
C(13)-P(1)-Pd(1)	113.71(11)
C(1)-N(1)-C(11)	119.4(3)
C(1)-N(1)-Pd(1)	120.2(2)
C(11)-N(1)-Pd(1)	120.3(2)
C(2)-C(1)-N(1)	121.7(3)
C(2)-P(2)-C(3)	103.36(16)
C(2)-P(2)-C(7)	102.55(16)
C(3)-P(2)-C(7)	115.40(16)
C(2)-P(2)-Pd(1)	100.75(12)
C(3)-P(2)-Pd(1)	115.93(12)
C(7)-P(2)-Pd(1)	115.60(12)
C(1)-C(2)-P(2)	116.3(3)
C(6)-C(3)-C(5)	109.2(3)
C(6)-C(3)-C(4)	108.5(3)
C(5)-C(3)-C(4)	108.8(3)
C(6)-C(3)-P(2)	112.9(2)
C(5)-C(3)-P(2)	113.2(3)
C(4)-C(3)-P(2)	103.9(2)
C(12)-C(11)-N(1)	121.7(3)
C(20)-C(17)-C(19)	109.2(3)
C(20)-C(17)-C(18)	108.9(3)
C(19)-C(17)-C(18)	109.4(3)
C(20)-C(17)-P(1)	111.0(2)
C(19)-C(17)-P(1)	114.0(2)
C(18)-C(17)-P(1)	104.2(2)
C(10)-C(7)-C(9)	109.0(3)
C(10)-C(7)-C(8)	109.7(3)
C(9)-C(7)-C(8)	108.5(3)
C(10)-C(7)-P(2)	104.5(3)
C(9)-C(7)-P(2)	112.2(2)
C(8)-C(7)-P(2)	112.9(2)
C(15)-C(13)-C(16)	109.6(3)
C(15)-C(13)-C(14)	108.9(3)
C(16)-C(13)-C(14)	108.6(3)
C(15)-C(13)-P(1)	112.3(2)
C(16)-C(13)-P(1)	113.2(2)
C(14)-C(13)-P(1)	104.0(2)
C(11)-C(12)-P(1)	116.2(3)

Symmetry transformations used to generate equivalent atoms:
#1 -x+1,-y+1,-z+1

Table S38: Torsion angles [°] for **2**^{Pd,As}.

C(11)-N(1)-C(1)-C(2)	-179.7(3)
Pd(1)-N(1)-C(1)-C(2)	-1.5(5)
N(1)-C(1)-C(2)-P(2)	2.1(5)
C(3)-P(2)-C(2)-C(1)	-121.8(3)
C(7)-P(2)-C(2)-C(1)	117.9(3)
Pd(1)-P(2)-C(2)-C(1)	-1.6(3)
C(2)-P(2)-C(3)-C(6)	-174.4(3)
C(7)-P(2)-C(3)-C(6)	-63.3(3)
Pd(1)-P(2)-C(3)-C(6)	76.4(3)
C(2)-P(2)-C(3)-C(5)	-49.7(3)
C(7)-P(2)-C(3)-C(5)	61.4(3)
Pd(1)-P(2)-C(3)-C(5)	-158.9(2)
C(2)-P(2)-C(3)-C(4)	68.2(3)
C(7)-P(2)-C(3)-C(4)	179.3(2)
Pd(1)-P(2)-C(3)-C(4)	-41.0(3)
C(1)-N(1)-C(11)-C(12)	-177.8(3)
Pd(1)-N(1)-C(11)-C(12)	4.0(5)
C(12)-P(1)-C(17)-C(20)	-163.1(3)
C(17)-P(1)-C(13)-C(15)	-57.7(3)
Pd(1)-P(1)-C(13)-C(15)	162.4(2)
C(13)-P(1)-C(17)-C(20)	-51.3(3)
Pd(1)-P(1)-C(17)-C(20)	86.8(3)
C(12)-P(1)-C(17)-C(19)	-39.3(3)
C(13)-P(1)-C(17)-C(19)	72.5(3)
Pd(1)-P(1)-C(17)-C(19)	-149.4(2)
C(12)-P(1)-C(17)-C(18)	79.8(3)
C(13)-P(1)-C(17)-C(18)	-168.4(2)
Pd(1)-P(1)-C(17)-C(18)	-30.3(3)
C(2)-P(2)-C(7)-C(10)	-76.0(3)
C(3)-P(2)-C(7)-C(10)	172.4(2)
Pd(1)-P(2)-C(7)-C(10)	32.5(3)
C(2)-P(2)-C(7)-C(9)	166.0(3)
C(3)-P(2)-C(7)-C(9)	54.4(3)
Pd(1)-P(2)-C(7)-C(9)	-85.4(3)
C(2)-P(2)-C(7)-C(8)	43.0(3)
C(3)-P(2)-C(7)-C(8)	-68.5(3)
Pd(1)-P(2)-C(7)-C(8)	151.6(2)
C(12)-P(1)-C(13)-C(15)	53.7(3)
C(17)-P(1)-C(13)-C(14)	-175.3(2)
Pd(1)-P(1)-C(13)-C(14)	44.8(2)

C(12)-P(1)-C(13)-C(16)	178.5(3)	N(1)-C(11)-C(12)-P(1)	-1.1(5)
C(17)-P(1)-C(13)-C(16)	67.1(3)	C(17)-P(1)-C(12)-C(11)	-124.1(3)
Pd(1)-P(1)-C(13)-C(16)	-72.8(3)	C(13)-P(1)-C(12)-C(11)	116.3(3)
C(12)-P(1)-C(13)-C(14)	-63.9(3)	Pd(1)-P(1)-C(12)-C(11)	-1.7(3)

Symmetry transformations used to generate equivalent atoms:
#1 -x+1,-y+1,-z+1

X-ray Single-Crystal Structure Analysis of [(μ-As_2){Pt(PNP)}$_2$] ($2^{Pt,As}$)

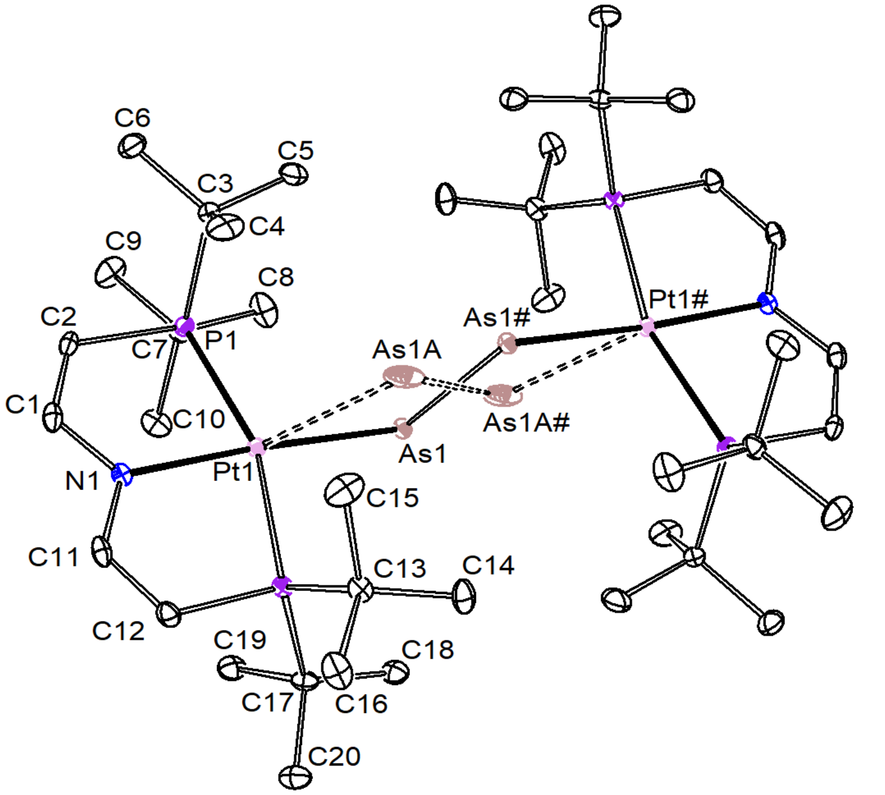

Figure S91: Thermal ellipsoid plot of $\mathbf{2}^{\mathbf{Pt,As}}$ with the anisotropic displacement parameters drawn at the 50% probability level. The asymmetric unit contains a half disordered complex molecule. The disordered As atom was refined with a population of 0.958(3) on the main domain.

Table S39: Crystal data and structure refinement for $\mathbf{2}^{\mathbf{Pt,As}}$.

Identification code	MN_110322_2_MO	(MN3-30)
Empirical formula	$C_{40}H_{80}As_2N_2P_4Pt_2$	
Formula weight	1252.96	
Temperature	100(2) K	
Wavelength	0.71073 Å	
Crystal system	Triclinic	
Space group	P-1	
Unit cell dimensions	a = 8.7520(6) Å	α= 79.108(4)°
	b = 11.9315(9) Å	β= 83.672(4)°
	c = 12.2126(9) Å	γ = 70.153(4)°
Volume	1176.47(15) Å^3	
Z	1	
Density (calculated)	1.768 Mg/m^3	
Absorption coefficient	7.499 mm^{-1}	
F(000)	616	
Crystal shape and color	Plate,	clear light green
Crystal size	0.241 x 0.079 x 0.060 mm^3	
Theta range for data collection	2.293 to 25.402°.	
Index ranges	-10<=h<=10, -14<=k<=14, -14<=l<=14	
Reflections collected	38367	
Independent reflections	4317 [R(int) = 0.0372]	
Completeness to theta = 25.242°	99.9 %	
Refinement method	Full-matrix least-squares on F^2	
Data / restraints / parameters	4317 / 0 / 248	
Goodness-of-fit on F^2	1.079	
Final R indices [I>2sigma(I)]	R1 = 0.0264,	wR2 = 0.0585
R indices (all data)	R1 = 0.0281,	wR2 = 0.0598
Largest diff. peak and hole	2.328 and -1.081 eÅ^{-3}	

Table S40: Bond lengths [Å] and angles [°] for $\mathbf{2}^{Pt,As}$.

Pt(1)-N(1)	2.102(4)	C(3)-P(1)-Pt(1)	115.70(16)
Pt(1)-P(1)	2.3186(12)	C(7)-P(1)-Pt(1)	115.35(17)
Pt(1)-P(2)	2.3220(12)	C(12)-P(2)-C(13)	103.0(2)
Pt(1)-As(1A)	2.436(17)	C(12)-P(2)-C(17)	103.6(2)
Pt(1)-As(1)	2.4668(5)	C(13)-P(2)-C(17)	114.3(2)
P(1)-C(2)	1.798(5)	C(12)-P(2)-Pt(1)	101.45(17)
P(1)-C(3)	1.874(5)	C(13)-P(2)-Pt(1)	117.87(16)
P(1)-C(7)	1.881(5)	C(17)-P(2)-Pt(1)	113.76(16)
P(2)-C(12)	1.787(5)	C(11)-N(1)-C(1)	119.5(4)
P(2)-C(13)	1.872(5)	C(11)-N(1)-Pt(1)	120.3(3)
P(2)-C(17)	1.874(5)	C(1)-N(1)-Pt(1)	120.1(3)
N(1)-C(11)	1.359(6)	C(2)-C(1)-N(1)	122.2(5)
N(1)-C(1)	1.360(7)	C(1)-C(2)-P(1)	115.3(4)
C(1)-C(2)	1.343(7)	C(5)-C(3)-C(6)	108.7(5)
C(3)-C(5)	1.518(7)	C(5)-C(3)-C(4)	109.0(5)
C(3)-C(6)	1.524(7)	C(6)-C(3)-C(4)	109.5(5)
C(3)-C(4)	1.526(8)	C(5)-C(3)-P(1)	112.0(4)
C(7)-C(8)	1.524(7)	C(6)-C(3)-P(1)	113.0(4)
C(7)-C(10)	1.531(7)	C(4)-C(3)-P(1)	104.5(3)
C(7)-C(9)	1.542(7)	C(8)-C(7)-C(10)	108.8(5)
C(11)-C(12)	1.339(7)	C(8)-C(7)-C(9)	108.8(5)
C(13)-C(14)	1.519(7)	C(10)-C(7)-C(9)	109.0(4)
C(13)-C(16)	1.529(7)	C(8)-C(7)-P(1)	112.5(3)
C(13)-C(15)	1.538(7)	C(10)-C(7)-P(1)	104.7(3)
C(17)-C(18)	1.525(7)	C(9)-C(7)-P(1)	112.9(4)
C(17)-C(20)	1.527(7)	C(12)-C(11)-N(1)	121.8(4)
C(17)-C(19)	1.538(7)	C(11)-C(12)-P(2)	115.7(4)
As(1)-As(1)#1	2.2888(9)	C(14)-C(13)-C(16)	109.1(4)
As(1A)-As(1A)#1	2.32(4)	C(14)-C(13)-C(15)	108.5(5)
N(1)-Pt(1)-P(1)	80.94(11)	C(16)-C(13)-C(15)	109.4(4)
N(1)-Pt(1)-P(2)	80.61(11)	C(14)-C(13)-P(2)	111.1(3)
P(1)-Pt(1)-P(2)	161.43(4)	C(16)-C(13)-P(2)	114.4(3)
N(1)-Pt(1)-As(1A)	149.1(5)	C(15)-C(13)-P(2)	104.1(3)
P(1)-Pt(1)-As(1A)	98.1(3)	C(18)-C(17)-C(20)	109.3(4)
P(2)-Pt(1)-As(1A)	98.8(3)	C(18)-C(17)-C(19)	108.3(4)
N(1)-Pt(1)-As(1)	172.37(11)	C(20)-C(17)-C(19)	108.5(4)
P(1)-Pt(1)-As(1)	98.88(3)	C(18)-C(17)-P(2)	113.6(3)
P(2)-Pt(1)-As(1)	99.13(3)	C(20)-C(17)-P(2)	112.6(3)
C(2)-P(1)-C(3)	102.6(2)	C(19)-C(17)-P(2)	104.3(3)
C(2)-P(1)-C(7)	103.2(2)	As(1)#1-As(1)-Pt(1)	115.55(3)
C(3)-P(1)-C(7)	115.5(2)	As(1A)#1-As(1A)-Pt(1)	116.8(10)
C(2)-P(1)-Pt(1)	101.46(17)		

Symmetry transformations used to generate equivalent atoms:
#1 -x+1,-y+1,-z+1

Table S41: Torsion angles [°] for $\mathbf{2}^{Pt,As}$.

C(11)-N(1)-C(1)-C(2)	-180.0(5)	Pt(1)-P(1)-C(3)-C(4)	-32.5(4)
Pt(1)-N(1)-C(1)-C(2)	-0.1(6)	C(2)-P(1)-C(7)-C(8)	174.9(4)
N(1)-C(1)-C(2)-P(1)	-1.0(7)	C(3)-P(1)-C(7)-C(8)	63.8(5)
C(3)-P(1)-C(2)-C(1)	-118.5(4)	Pt(1)-P(1)-C(7)-C(8)	-75.5(4)
C(7)-P(1)-C(2)-C(1)	121.1(4)	C(2)-P(1)-C(7)-C(10)	-67.2(4)
Pt(1)-P(1)-C(2)-C(1)	1.4(4)	C(3)-P(1)-C(7)-C(10)	-178.3(3)
C(2)-P(1)-C(3)-C(5)	-165.2(4)	Pt(1)-P(1)-C(7)-C(10)	42.5(4)
C(7)-P(1)-C(3)-C(5)	-53.8(5)	C(2)-P(1)-C(7)-C(9)	51.2(4)
Pt(1)-P(1)-C(3)-C(5)	85.3(4)	C(3)-P(1)-C(7)-C(9)	-59.8(4)
C(2)-P(1)-C(3)-C(6)	-42.1(4)	Pt(1)-P(1)-C(7)-C(9)	160.9(3)
C(7)-P(1)-C(3)-C(6)	69.4(4)	C(1)-N(1)-C(11)-C(12)	177.1(5)
Pt(1)-P(1)-C(3)-C(6)	-151.5(3)	Pt(1)-N(1)-C(11)-C(12)	-2.8(6)
C(2)-P(1)-C(3)-C(4)	77.0(4)	N(1)-C(11)-C(12)-P(2)	0.6(7)
C(7)-P(1)-C(3)-C(4)	-171.6(4)	C(13)-P(2)-C(12)-C(11)	123.9(4)
C(17)-P(2)-C(12)-C(11)	-116.7(4)	Pt(1)-P(2)-C(13)-C(15)	30.3(4)
Pt(1)-P(2)-C(12)-C(11)	1.5(4)	C(12)-P(2)-C(17)-C(18)	-179.2(4)

C(12)-P(2)-C(13)-C(14)	163.0(4)	C(13)-P(2)-C(17)-C(18)	-67.9(4)
C(17)-P(2)-C(13)-C(14)	51.3(4)	Pt(1)-P(2)-C(17)-C(18)	71.5(4)
Pt(1)-P(2)-C(13)-C(14)	-86.4(4)	C(12)-P(2)-C(17)-C(20)	-54.3(4)
C(12)-P(2)-C(13)-C(16)	38.9(4)	C(13)-P(2)-C(17)-C(20)	57.0(4)
C(17)-P(2)-C(13)-C(16)	-72.8(4)	Pt(1)-P(2)-C(17)-C(20)	-163.6(3)
Pt(1)-P(2)-C(13)-C(16)	149.5(3)	C(12)-P(2)-C(17)-C(19)	63.2(4)
C(12)-P(2)-C(13)-C(15)	-80.4(4)	C(13)-P(2)-C(17)-C(19)	174.5(3)
C(17)-P(2)-C(13)-C(15)	167.9(4)	Pt(1)-P(2)-C(17)-C(19)	-46.1(4)

Symmetry transformations used to generate equivalent atoms:
#1 -x+1,-y+1,-z+1

X-ray Single-Crystal Structure Analysis of [(μ-Sb_2){Pt(PNP)}$_2$] ($2^{Pt,Sb}$)

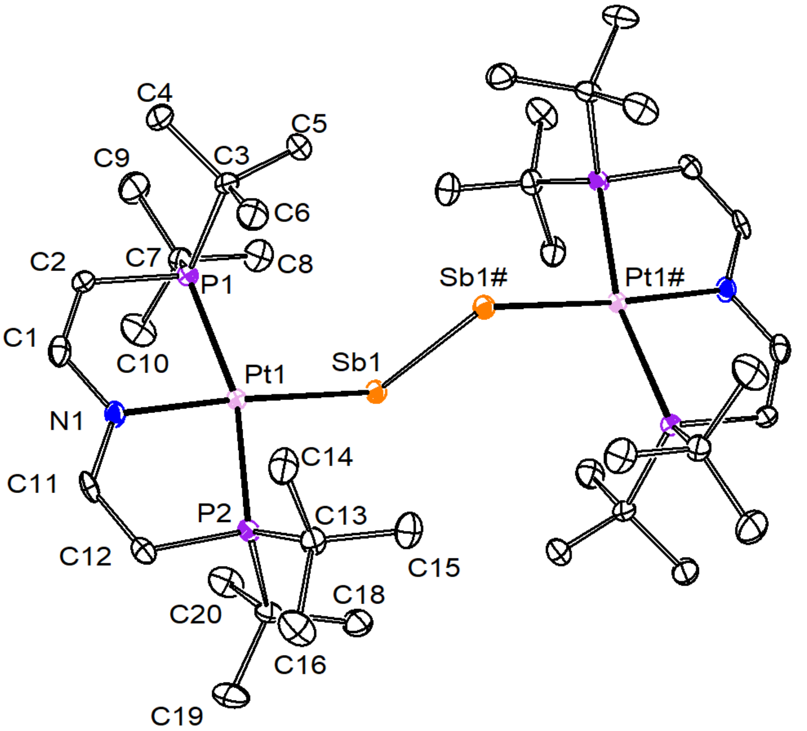

Figure S92: Thermal ellipsoid plot of $2^{Pt,Sb}$ with the anisotropic displacement parameters drawn at the 50% probability level. The asymmetric unit contains one half complex molecule.

Table S42: Crystal data and structure refinement for ($2^{Pt,Sb}$).

Identification code	MN_230417_MN_01_a	
Empirical formula	$C_{40} H_{80} N_2 P_4 Pt_2 Sb_2$	
Formula weight	1346.62	
Temperature	100(2) K	
Wavelength	0.71073 Å	
Crystal system	Monoclinic	
Space group	P 1 21/n 1	
Unit cell dimensions	a = 14.5017(5) Å	α= 90°.
	b = 12.0578(4) Å	β= 111.2260(10)°.
	c = 14.6939(5) Å	γ = 90°.
Volume	2395.05(14) $Å^3$	
Z	2	
Density (calculated)	1.867 Mg/m^3	
Absorption coefficient	7.100 mm^{-1}	
F(000)	1304	
Crystal size	0.084 x 0.047 x 0.039 mm^3	
Crystal shape and color	plate,	clear intense orange-yellow
Theta range for data collection	2.25 to 28.31°.	
Index ranges	-19<=h<=19, -16<=k<=16, -19<=l<=19	
Reflections collected	114038	
Independent reflections	5944 [R(int) = 0.1377]	
Completeness to theta = 28.31°	99.9 %	
Absorption correction	Semi-empirical from equivalents	
Max. and min. transmission	0.77 and 0.69	
Refinement method	Full-matrix least-squares on F^2	
Data / restraints / parameters	5944 / 132 / 238	
Goodness-of-fit on F^2	0.883	
Final R indices [I>2sigma(I)]	R1 = 0.0325, wR2 = 0.0630	
R indices (all data)	R1 = 0.0520, wR2 = 0.0724	
Largest diff. peak and hole	0.915 and -1.451 $eÅ^{-3}$	

Table S43: Bond lengths [Å] and angles [°] for ($2^{Pt,Sb}$).

Pt(1)-N(1)	2.086(4)	C(3)-P(1)-Pt(1)	115.34(17)
Pt(1)-P(1)	2.3121(14)	C(11)-N(1)-C(1)	120.7(5)
Pt(1)-P(2)	2.3143(14)	C(11)-N(1)-Pt(1)	119.9(4)
Pt(1)-Sb(1)	2.6066(4)	C(1)-N(1)-Pt(1)	119.4(4)
Sb(1)-Sb(1)#1	2.6638(7)	C(12)-P(2)-C(17)	103.6(2)
P(1)-C(2)	1.785(5)	C(12)-P(2)-C(13)	103.5(3)
P(1)-C(7)	1.875(6)	C(17)-P(2)-C(13)	114.2(2)
P(1)-C(3)	1.888(6)	C(12)-P(2)-Pt(1)	100.7(2)
N(1)-C(11)	1.353(7)	C(17)-P(2)-Pt(1)	117.06(17)
N(1)-C(1)	1.375(7)	C(13)-P(2)-Pt(1)	114.89(17)
P(2)-C(12)	1.786(6)	C(2)-C(1)-N(1)	121.7(5)
P(2)-C(17)	1.878(6)	C(1)-C(2)-P(1)	116.1(4)
P(2)-C(13)	1.879(6)	C(5)-C(3)-C(6)	109.8(5)
C(1)-C(2)	1.343(8)	C(5)-C(3)-C(4)	109.5(5)
C(3)-C(5)	1.516(7)	C(6)-C(3)-C(4)	107.9(5)
C(3)-C(6)	1.533(7)	C(5)-C(3)-P(1)	112.5(4)
C(3)-C(4)	1.535(7)	C(6)-C(3)-P(1)	104.8(4)
C(7)-C(9)	1.522(7)	C(4)-C(3)-P(1)	112.1(4)
C(7)-C(10)	1.529(7)	C(9)-C(7)-C(10)	109.0(5)
C(7)-C(8)	1.534(7)	C(9)-C(7)-C(8)	108.8(5)
C(11)-C(12)	1.345(8)	C(10)-C(7)-C(8)	108.1(5)
C(13)-C(14)	1.522(7)	C(9)-C(7)-P(1)	112.6(4)
C(13)-C(15)	1.529(7)	C(10)-C(7)-P(1)	105.0(4)
C(13)-C(16)	1.537(7)	C(8)-C(7)-P(1)	113.2(4)
C(17)-C(18)	1.529(7)	C(12)-C(11)-N(1)	121.9(5)
C(17)-C(20)	1.534(7)	C(11)-C(12)-P(2)	115.9(4)
C(17)-C(19)	1.535(7)	C(14)-C(13)-C(15)	107.9(5)
N(1)-Pt(1)-P(1)	81.76(13)	C(14)-C(13)-C(16)	109.3(5)
N(1)-Pt(1)-P(2)	81.53(13)	C(15)-C(13)-C(16)	109.1(5)
P(1)-Pt(1)-P(2)	163.22(5)	C(14)-C(13)-P(2)	104.7(4)
N(1)-Pt(1)-Sb(1)	172.08(13)	C(15)-C(13)-P(2)	113.4(4)
P(1)-Pt(1)-Sb(1)	99.22(4)	C(16)-C(13)-P(2)	112.2(4)
P(2)-Pt(1)-Sb(1)	97.55(4)	C(18)-C(17)-C(20)	109.1(5)
Pt(1)-Sb(1)-Sb(1)#1	111.32(2)	C(18)-C(17)-C(19)	109.3(5)
C(2)-P(1)-C(7)	102.8(2)	C(20)-C(17)-C(19)	108.0(5)
C(2)-P(1)-C(3)	103.5(2)	C(18)-C(17)-P(2)	113.0(4)
C(7)-P(1)-C(3)	113.4(2)	C(20)-C(17)-P(2)	104.7(4)
C(2)-P(1)-Pt(1)	100.97(19)	C(19)-C(17)-P(2)	112.5(4)
C(7)-P(1)-Pt(1)	117.76(18)		

Symmetry transformations used to generate equivalent atoms:
#1 -x+1,-y+1,-z+1

Table S44: Torsion angles [°] for **$2^{Pt,Sb}$**.

C(11)-N(1)-C(1)-C(2)	-179.7(5)	Pt(1)-P(1)-C(7)-C(9)	168.1(3)
Pt(1)-N(1)-C(1)-C(2)	0.2(7)	C(2)-P(1)-C(7)-C(10)	-60.2(4)
N(1)-C(1)-C(2)-P(1)	-0.5(7)	C(3)-P(1)-C(7)-C(10)	-171.3(4)
C(7)-P(1)-C(2)-C(1)	122.5(4)	Pt(1)-P(1)-C(7)-C(10)	49.6(4)
C(3)-P(1)-C(2)-C(1)	-119.2(4)	C(2)-P(1)-C(7)-C(8)	-177.9(4)
Pt(1)-P(1)-C(2)-C(1)	0.5(5)	C(3)-P(1)-C(7)-C(8)	71.0(5)
C(2)-P(1)-C(3)-C(5)	-156.1(4)	Pt(1)-P(1)-C(7)-C(8)	-68.0(4)
C(7)-P(1)-C(3)-C(5)	-45.5(5)	C(1)-N(1)-C(11)-C(12)	-179.3(5)
Pt(1)-P(1)-C(3)-C(5)	94.6(4)	Pt(1)-N(1)-C(11)-C(12)	0.8(7)
C(2)-P(1)-C(3)-C(6)	84.5(4)	N(1)-C(11)-C(12)-P(2)	1.0(7)
C(7)-P(1)-C(3)-C(6)	-164.9(4)	C(17)-P(2)-C(12)-C(11)	-123.4(4)
Pt(1)-P(1)-C(3)-C(6)	-24.8(4)	C(13)-P(2)-C(12)-C(11)	117.1(5)
C(2)-P(1)-C(3)-C(4)	-32.2(5)	Pt(1)-P(2)-C(12)-C(11)	-1.9(5)
C(7)-P(1)-C(3)-C(4)	78.4(5)	C(12)-P(2)-C(13)-C(14)	-65.6(4)
Pt(1)-P(1)-C(3)-C(4)	-141.5(4)	C(17)-P(2)-C(13)-C(14)	-177.4(4)
C(2)-P(1)-C(7)-C(9)	58.2(5)	Pt(1)-P(2)-C(13)-C(14)	43.2(4)
C(3)-P(1)-C(7)-C(9)	-52.9(5)	C(12)-P(2)-C(13)-C(15)	177.0(4)
C(17)-P(2)-C(13)-C(15)	65.2(5)	Pt(1)-P(2)-C(17)-C(18)	87.1(4)

Pt(1)-P(2)-C(13)-C(15)	-74.2(4)	C(12)-P(2)-C(17)-C(20)	78.2(4)
C(12)-P(2)-C(13)-C(16)	52.8(5)	C(13)-P(2)-C(17)-C(20)	-169.9(3)
C(17)-P(2)-C(13)-C(16)	-59.1(5)	Pt(1)-P(2)-C(17)-C(20)	-31.5(4)
Pt(1)-P(2)-C(13)-C(16)	161.6(4)	C(12)-P(2)-C(17)-C(19)	-38.8(5)
C(12)-P(2)-C(17)-C(18)	-163.1(4)	C(13)-P(2)-C(17)-C(19)	73.1(5)
C(13)-P(2)-C(17)-C(18)	-51.3(5)	Pt(1)-P(2)-C(17)-C(19)	-148.5(4)

Symmetry transformations used to generate equivalent atoms:
#1 -x+1,-y+1,-z+1

X-ray Single-Crystal Structure Analysis of [(*μ*-Bi_2){Pt(PNP)}$_2$] ($2^{Pt,Bi}$)

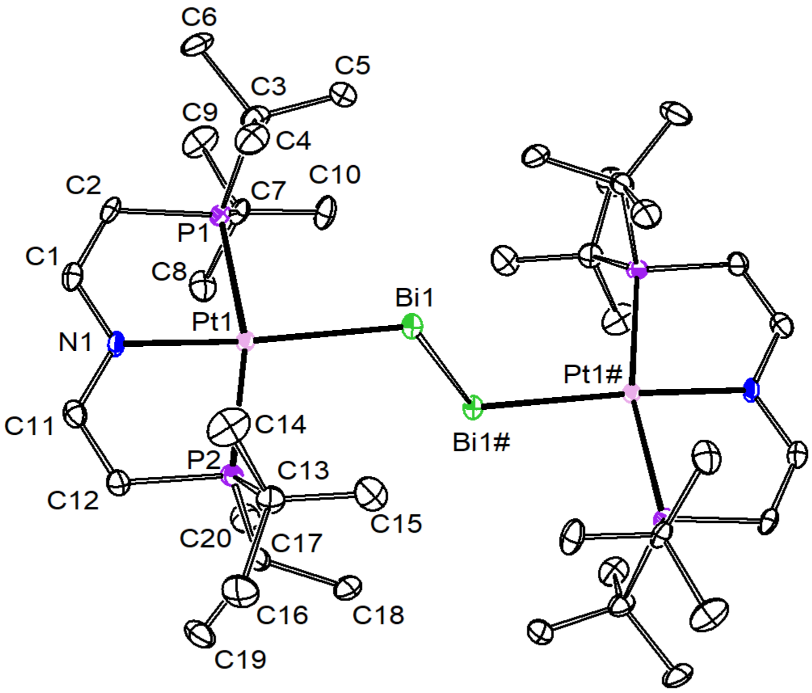

Figure S93: Thermal ellipsoid plot of $2^{Pt,Bi}$ with the anisotropic displacement parameters drawn at the 50% probability level. The asymmetric unit contains one half complex molecule. The reflection 0 1 1 was removed from the refinement using OMIT command.

Table S45: Crystal data and structure refinement for $2^{Pt,Bi}$.

Identification code	mo_MN_230823_05_MN_0m_a	
Empirical formula	C_{40} H_{80} Bi_2 N_2 P_4 Pt_2	
Formula weight	1521.08	
Temperature	102(2) K	
Wavelength	0.71073 Å	
Crystal system	Monoclinic	
Space group	P 1 21/n 1	
Unit cell dimensions	a = 14.6415(16) Å	α= 90°.
	b = 11.9910(12) Å	β= 111.711(4)°.
	c = 14.8209(14) Å	γ = 90°.
Volume	2417.5(4) Å^3	
Z	2	
Density (calculated)	2.090 Mg/m^3	
Absorption coefficient	13.186 mm^{-1}	
F(000)	1432	
Crystal size	0.076 x 0.060 x 0.053 mm^3	
Crystal shape and color	plate,	clear, intense green-red
Theta range for data collection	2.38 to 30.55°.	
Index ranges	-20<=h<=20, -17<=k<=17, -21<=l<=21	
Reflections collected	91584	
Independent reflections	7394 [R(int) = 0.0719]	
Completeness to theta = 30.55°	99.9 %	
Absorption correction	Semi-empirical from equivalents	
Max. and min. transmission	0.54 and 0.41	
Refinement method	Full-matrix least-squares on F^2	
Data / restraints / parameters	7394 / 132 / 238	
Goodness-of-fit on F^2	0.978	
Final R indices [I>2sigma(I)]	R1 = 0.0267, wR2 = 0.0703	
R indices (all data)	R1 = 0.0394, wR2 = 0.0832	
Largest diff. peak and hole	1.459 and -2.505 eÅ^{-3}	

Table S46: Bond lengths [Å] and angles [°] for $\mathbf{2}^{Pt,Bi}$.

Pt(1)-N(1)	2.075(4)	C(1)-N(1)-C(11)	120.8(4)
Pt(1)-P(2)	2.3062(12)	C(1)-N(1)-Pt(1)	119.7(3)
Pt(1)-P(1)	2.3066(12)	C(11)-N(1)-Pt(1)	119.5(3)
Pt(1)-Bi(1)	2.6992(3)	Pt(1)-Bi(1)-Bi(1)#1	108.735(13)
P(1)-C(2)	1.791(5)	C(12)-P(2)-C(13)	102.6(2)
P(1)-C(7)	1.883(5)	C(12)-P(2)-C(17)	103.8(2)
P(1)-C(3)	1.894(5)	C(13)-P(2)-C(17)	112.8(2)
N(1)-C(1)	1.362(6)	C(12)-P(2)-Pt(1)	100.73(17)
N(1)-C(11)	1.379(6)	C(13)-P(2)-Pt(1)	118.15(16)
Bi(1)-Bi(1)#1	2.8400(4)	C(17)-P(2)-Pt(1)	115.64(16)
P(2)-C(12)	1.789(5)	C(2)-C(1)-N(1)	121.7(4)
P(2)-C(13)	1.872(5)	C(1)-C(2)-P(1)	116.0(4)
P(2)-C(17)	1.881(5)	C(5)-C(3)-C(6)	109.3(4)
C(1)-C(2)	1.343(7)	C(5)-C(3)-C(4)	109.8(4)
C(3)-C(5)	1.526(7)	C(6)-C(3)-C(4)	108.4(4)
C(3)-C(6)	1.528(6)	C(5)-C(3)-P(1)	112.4(3)
C(3)-C(4)	1.536(7)	C(6)-C(3)-P(1)	112.2(3)
C(7)-C(10)	1.519(6)	C(4)-C(3)-P(1)	104.5(3)
C(7)-C(8)	1.524(6)	C(10)-C(7)-C(8)	108.4(4)
C(7)-C(9)	1.532(6)	C(10)-C(7)-C(9)	109.2(4)
C(11)-C(12)	1.346(7)	C(8)-C(7)-C(9)	109.2(4)
C(13)-C(16)	1.522(6)	C(10)-C(7)-P(1)	113.3(3)
C(13)-C(15)	1.531(7)	C(8)-C(7)-P(1)	104.4(3)
C(13)-C(14)	1.543(7)	C(9)-C(7)-P(1)	112.2(3)
C(17)-C(18)	1.527(7)	C(12)-C(11)-N(1)	121.4(5)
C(17)-C(19)	1.533(6)	C(11)-C(12)-P(2)	116.1(4)
C(17)-C(20)	1.545(7)	C(16)-C(13)-C(15)	108.6(4)
N(1)-Pt(1)-P(2)	82.21(12)	C(16)-C(13)-C(14)	109.3(5)
N(1)-Pt(1)-P(1)	82.03(12)	C(15)-C(13)-C(14)	108.4(5)
P(2)-Pt(1)-P(1)	164.15(4)	C(16)-C(13)-P(2)	112.2(4)
N(1)-Pt(1)-Bi(1)	172.53(12)	C(15)-C(13)-P(2)	112.9(3)
P(2)-Pt(1)-Bi(1)	98.96(3)	C(14)-C(13)-P(2)	105.2(3)
P(1)-Pt(1)-Bi(1)	96.88(3)	C(18)-C(17)-C(19)	109.4(4)
C(2)-P(1)-C(7)	103.6(2)	C(18)-C(17)-C(20)	109.1(4)
C(2)-P(1)-C(3)	103.7(2)	C(19)-C(17)-C(20)	108.2(4)
C(7)-P(1)-C(3)	113.6(2)	C(18)-C(17)-P(2)	112.8(3)
C(2)-P(1)-Pt(1)	100.42(17)	C(19)-C(17)-P(2)	112.3(3)
C(7)-P(1)-Pt(1)	115.10(15)	C(20)-C(17)-P(2)	104.8(3)
C(3)-P(1)-Pt(1)	117.58(16)		

Table S47: Torsion angles [°] for $\mathbf{2}^{Pt,Bi}$.

C(11)-N(1)-C(1)-C(2)	178.8(5)	C(3)-P(1)-C(7)-C(9)	-57.5(4)
Pt(1)-N(1)-C(1)-C(2)	1.8(6)	Pt(1)-P(1)-C(7)-C(9)	162.9(3)
N(1)-C(1)-C(2)-P(1)	0.8(6)	C(1)-N(1)-C(11)-C(12)	-179.0(5)
C(7)-P(1)-C(2)-C(1)	116.8(4)	Pt(1)-N(1)-C(11)-C(12)	-1.9(6)
C(3)-P(1)-C(2)-C(1)	-124.4(4)	N(1)-C(11)-C(12)-P(2)	0.3(7)
Pt(1)-P(1)-C(2)-C(1)	-2.5(4)	C(13)-P(2)-C(12)-C(11)	123.4(4)
C(2)-P(1)-C(3)-C(5)	-160.6(4)	C(17)-P(2)-C(12)-C(11)	-118.9(4)
C(7)-P(1)-C(3)-C(5)	-48.9(4)	Pt(1)-P(2)-C(12)-C(11)	1.1(4)
Pt(1)-P(1)-C(3)-C(5)	89.7(4)	C(12)-P(2)-C(13)-C(16)	58.8(4)
C(2)-P(1)-C(3)-C(6)	-36.9(4)	C(17)-P(2)-C(13)-C(16)	-52.3(4)
C(7)-P(1)-C(3)-C(6)	74.8(4)	Pt(1)-P(2)-C(13)-C(16)	168.4(3)
Pt(1)-P(1)-C(3)-C(6)	-146.7(3)	C(12)-P(2)-C(13)-C(15)	-178.0(4)
C(2)-P(1)-C(3)-C(4)	80.3(4)	C(17)-P(2)-C(13)-C(15)	70.9(4)
C(7)-P(1)-C(3)-C(4)	-168.0(3)	Pt(1)-P(2)-C(13)-C(15)	-68.4(4)
Pt(1)-P(1)-C(3)-C(4)	-29.4(4)	C(12)-P(2)-C(13)-C(14)	-59.9(4)
C(2)-P(1)-C(7)-C(10)	178.5(4)	C(17)-P(2)-C(13)-C(14)	-171.0(4)
C(3)-P(1)-C(7)-C(10)	66.7(4)	Pt(1)-P(2)-C(13)-C(14)	49.7(4)
Pt(1)-P(1)-C(7)-C(10)	-73.0(4)	C(12)-P(2)-C(17)-C(18)	-156.7(4)
C(2)-P(1)-C(7)-C(8)	-63.8(4)	C(13)-P(2)-C(17)-C(18)	-46.4(4)
C(3)-P(1)-C(7)-C(8)	-175.6(3)	Pt(1)-P(2)-C(17)-C(18)	94.0(4)
Pt(1)-P(1)-C(7)-C(8)	44.8(3)	C(12)-P(2)-C(17)-C(19)	-32.5(5)
C(2)-P(1)-C(7)-C(9)	54.3(4)	C(13)-P(2)-C(17)-C(19)	77.9(4)
Pt(1)-P(2)-C(17)-C(19)	-141.8(4)	C(13)-P(2)-C(17)-C(20)	-164.9(3)
C(12)-P(2)-C(17)-C(20)	84.7(4)	Pt(1)-P(2)-C(17)-C(20)	-24.6(4)

Symmetry transformations used to generate equivalent atoms: #1 -x+1,-y+1,-z+1

X-ray Single-Crystal Structure Analysis of [Pd(P)(PNP)] ($3^{Pd,P}$)

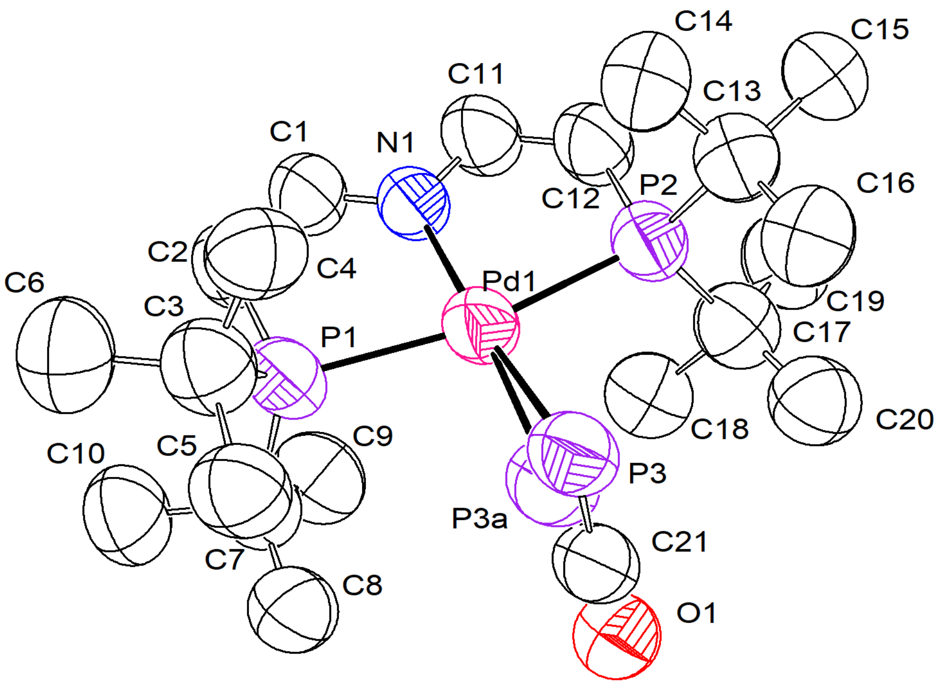

Figure S94: Thermal ellipsoid plot of $3^{Pd,P}$ with the anisotropic displacement parameters drawn at the 50% probability level. The asymmetric unit contains one disordered complex molecule. The disordered complex molecule was refined with population of 0.58(4) on the main domain using some restraints and constraints (SIMU, RIGU).

Table S48: Crystal data and structure refinement for $3^{Pd,P}$.

Identification code	run14	
Empirical formula	$C_{20.59}$ H_{40} N $O_{0.59}$ P_3 Pd	
Formula weight	510.29	
Temperature	100.00 K	
Wavelength	0.41328 Å	
Crystal system	Orthorhombic	
Space group	$Pna2_1$	
Unit cell dimensions	a = 18.721(8) Å	α= 90°.
	b = 15.986(7) Å	β= 90°.
	c = 8.502(4) Å	γ = 90°.
Volume	2544.5(18) $Å^3$	
Z	4	
Density (calculated)	1.332 Mg/m^3	
Absorption coefficient	1.026 mm^{-1}	
F(000)	1065	
Crystal size	0.07 x 0.06 x 0.02 mm^3	
Theta range for data collection	1.265 to 9.930°.	
Index ranges	-15<=h<=15, -13<=k<=13, -7<=l<=7	
Reflections collected	9892	
Independent reflections	1533 [R(int) = 0.0775]	
Completeness to theta = 9.930°	99.30 %	
Absorption correction	Semi-empirical from equivalents	
Max. and min. transmission	0.7434 and 0.5359	
Refinement method	Full-matrix least-squares on F^2	
Data / restraints / parameters	1533 / 407 / 254	
Goodness-of-fit on F^2	1.147	
Final R indices [I>2sigma(I)]	R1 = 0.0561, wR2 = 0.1351	
R indices (all data)	R1 = 0.0639, wR2 = 0.1438	
Absolute structure parameter	0.073(18)	
Largest diff. peak and hole	0.683and -0.297 $eÅ^-$	

Table S49: Bond lengths [Å] and angles [°] for $3^{Pd,P}$.

Pd(1)-P(1)	2.342(4)	C(13)-P(2)-Pd(1)	115.0(5)
Pd(1)-P(2)	2.331(4)	C(13)-P(2)-C(12)	106.5(7)
Pd(1)-P(3)	2.35(3)	C(17)-P(2)-Pd(1)	117.4(5)
Pd(1)-N(1)	2.033(10)	C(17)-P(2)-C(12)	102.8(7)
Pd(1)-P(3A)	2.31(5)	C(17)-P(2)-C(13)	112.8(7)
P(1)-C(2)	1.729(14)	C(21)-P(3)-Pd(1)	96.3(9)
P(1)-C(3)	1.832(16)	C(1)-N(1)-Pd(1)	119.8(10)
P(1)-C(7)	1.846(18)	C(11)-N(1)-Pd(1)	119.6(10)
P(2)-C(12)	1.754(16)	C(11)-N(1)-C(1)	120.6(13)
P(2)-C(13)	1.855(16)	H(1)-C(1)-N(1)	118.2(9)
P(2)-C(17)	1.880(17)	C(2)-C(1)-N(1)	123.6(14)
P(3)-C(21)	1.71(3)	C(2)-C(1)-H(1)	118.2(9)
O(1)-C(21)	1.15(3)	C(1)-C(2)-P(1)	115.2(11)
N(1)-C(1)	1.283(16)	H(2)-C(2)-P(1)	122.4(5)
N(1)-C(11)	1.364(17)	H(2)-C(2)-C(1)	122.4(9)
C(1)-C(2)	1.393(18)	H(11)-C(11)-N(1)	119.1(9)
C(11)-C(12)	1.39(2)	C(12)-C(11)-N(1)	121.8(15)
C(3)-C(5)	1.61(2)	C(12)-C(11)-H(11)	119.1(9)
C(3)-C(6)	1.54(2)	C(11)-C(12)-P(2)	115.5(12)
C(3)-C(4)	1.47(2)	H(12)-C(12)-P(2)	122.3(6)
C(7)-C(8)	1.408(19)	H(12)-C(12)-C(11)	122.3(9)
C(7)-C(10)	1.58(2)	C(5)-C(3)-P(1)	108.9(11)
C(7)-C(9)	1.50(2)	C(6)-C(3)-P(1)	116.4(12)
C(13)-C(14)	1.542(19)	C(6)-C(3)-C(5)	107.3(13)
C(13)-C(15)	1.62(2)	C(4)-C(3)-P(1)	108.0(11)
C(13)-C(16)	1.521(19)	C(4)-C(3)-C(5)	109.3(14)
C(17)-C(18)	1.60(2)	C(4)-C(3)-C(6)	106.9(15)
C(17)-C(19)	1.53(2)	C(8)-C(7)-P(1)	113.5(12)
C(17)-C(20)	1.47(2)	C(10)-C(7)-P(1)	109.8(12)
P(2)-Pd(1)-P(1)	164.66(14)	C(10)-C(7)-C(8)	107.2(14)
P(3)-Pd(1)-P(1)	101.1(3)	C(9)-C(7)-P(1)	103.8(12)
P(3)-Pd(1)-P(2)	94.2(3)	C(9)-C(7)-C(8)	112.3(16)
N(1)-Pd(1)-P(1)	82.0(3)	C(9)-C(7)-C(10)	110.2(14)
N(1)-Pd(1)-P(2)	82.7(3)	C(14)-C(13)-P(2)	106.0(10)
N(1)-Pd(1)-P(3)	170.3(4)	C(15)-C(13)-P(2)	108.4(10)
P(3A)-Pd(1)-P(1)	94.8(4)	C(15)-C(13)-C(14)	111.9(12)
P(3A)-Pd(1)-P(2)	100.3(4)	C(16)-C(13)-P(2)	113.4(11)
P(3A)-Pd(1)-P(3)	12.6(5)	C(16)-C(13)-C(14)	107.3(13)
P(3A)-Pd(1)-N(1)	176.4(6)	C(16)-C(13)-C(15)	109.8(12)
C(2)-P(1)-Pd(1)	99.1(5)	C(18)-C(17)-P(2)	102.2(11)
C(3)-P(1)-Pd(1)	116.1(6)	C(19)-C(17)-P(2)	114.7(12)
C(3)-P(1)-C(2)	103.5(8)	C(19)-C(17)-C(18)	103.0(13)
C(7)-P(1)-Pd(1)	111.8(5)	C(20)-C(17)-P(2)	111.5(11)
C(7)-P(1)-C(2)	105.9(7)	C(20)-C(17)-C(18)	109.0(14)
C(7)-P(1)-C(3)	117.5(8)	C(20)-C(17)-C(19)	115.2(15)
C(12)-P(2)-Pd(1)	100.1(6)	O(1)-C(21)-P(3)	175(2)

Symmetry transformations used to generate equivalent atoms:

Table S50: Torsion angles [°] for $3^{Pd,P}$.

P2-Pd1-P1-C2	-11.6(8)	N1-Pd1-P1-C7	107.1(6)
P2-Pd1-P1-C3	-121.6(8)	P1-Pd1-P2-C12	3.5(8)
P2-Pd1-P1-C7	99.6(8)	P1-Pd1-P2-C13	117.1(7)
P3-Pd1-P1-C2	166.5(6)	P1-Pd1-P2-C17	-106.7(8)
P3-Pd1-P1-C3	56.5(7)	P3-Pd1-P2-C12	-174.7(6)
P3-Pd1-P1-C7	-82.2(6)	P3-Pd1-P2-C13	-61.1(6)
N1-Pd1-P1-C2	-4.2(6)	P3-Pd1-P2-C17	75.2(7)
N1-Pd1-P1-C3	-114.2(7)	N1-Pd1-P2-C12	-3.9(6)
N1-Pd1-P2-C13	109.7(6)	C3-P1-C7-C10	58(1)
N1-Pd1-P2-C17	-114.1(7)	C3-P1-C7-C9	176(1)
P1-Pd1-P3-C21	90.8(9)	Pd1-P2-C12-C11	6(1)

P2-Pd1-P3-C21	-89.7(9)	C13-P2-C12-C11	-114(1)
N1-Pd1-P3-C21	-161(2)	C17-P2-C12-C11	127(1)
P1-Pd1-N1-C1	5(1)	Pd1-P2-C13-C14	-43(1)
P1-Pd1-N1-C11	-176(1)	Pd1-P2-C13-C15	-163.2(8)
P2-Pd1-N1-C1	-177(1)	Pd1-P2-C13-C16	74(1)
P2-Pd1-N1-C11	2(1)	C12-P2-C13-C14	67(1)
P3-Pd1-N1-C1	-105(2)	C12-P2-C13-C15	-53(1)
P3-Pd1-N1-C11	74(3)	C12-P2-C13-C16	-176(1)
Pd1-P1-C2-C1	4(1)	C17-P2-C13-C14	179(1)
C3-P1-C2-C1	124(1)	C17-P2-C13-C15	59(1)
C7-P1-C2-C1	-112(1)	C17-P2-C13-C16	-64(1)
Pd1-P1-C3-C5	-81(1)	Pd1-P2-C17-C18	32(1)
Pd1-P1-C3-C6	158(1)	Pd1-P2-C17-C19	143(1)
Pd1-P1-C3-C4	38(1)	Pd1-P2-C17-C20	-84(1)
C2-P1-C3-C5	172(1)	C12-P2-C17-C18	-77(1)
C2-P1-C3-C6	50(1)	C12-P2-C17-C19	34(1)
C2-P1-C3-C4	-70(1)	C12-P2-C17-C20	167(1)
C7-P1-C3-C5	55(1)	C13-P2-C17-C18	169(1)
C7-P1-C3-C6	-66(1)	C13-P2-C17-C19	-80(1)
C7-P1-C3-C4	174(1)	C13-P2-C17-C20	53(1)
Pd1-P1-C7-C8	76(1)	Pd1-P3-C21-O1	158(25)
Pd1-P1-C7-C10	-164.0(9)	Pd1-N1-C1-C2	-3(2)
Pd1-P1-C7-C9	-46(1)	C11-N1-C1-C2	177(1)
C2-P1-C7-C8	-177(1)	Pd1-N1-C11-C12	2(2)
C2-P1-C7-C10	-57(1)	N1-C1-C2-P1	-1(2)
C2-P1-C7-C9	61(1)	C1-N1-C11-C12	-179(1)
C3-P1-C7-C8	-62(1)		

Symmetry transformations used to generate equivalent atoms:

X-ray Single-Crystal Structure Analysis of [Pd(As)(PNP)] ($3^{Pd,As}$)

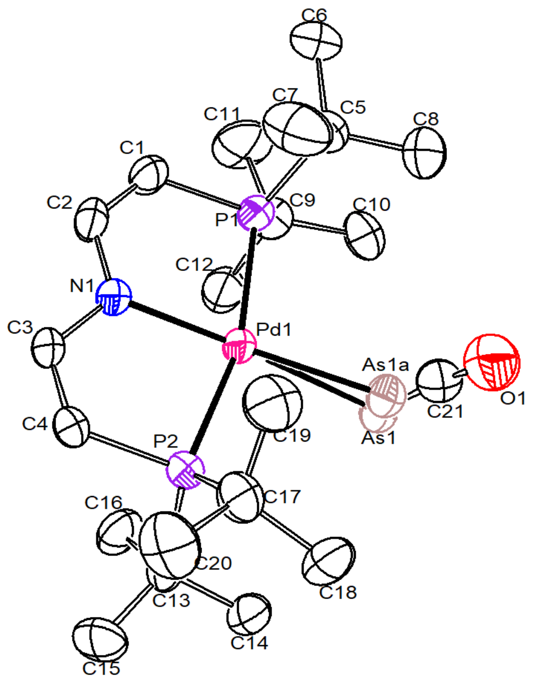

Figure S95: Thermal ellipsoid plot of $\mathbf{3^{Pd,As}}$ with the anisotropic displacement parameters drawn at the 50% probability level. The asymmetric unit contains one disordered complex molecule. The disordered complex molecule was refined with population of 0.85(2) on the main domain using some restraints and constraints (SIMU, RIGU, EADP). The Pd-As, Pd-As1, As1-C21 and C21-O1 atoms were calculated and constraint to 2.451, 2.357, 1.18 and 1.7 Å by using DFIX and isotropically refined.

Table S51: Crystal data and structure refinement for $\mathbf{3^{Pd,As}}$.

Identification code	run4_pnppdasco	
Empirical formula	$C_{20.85}H_{40}AsNO_{0.85}P_2Pd$	
Formula weight	561.53	
Temperature	100.0 K	
Wavelength	0.41328 Å	
Crystal system	Orthorhombic	
Space group	$Pna2_1$	
Unit cell dimensions	a = 18.750(3) Å	α= 90°.
	b = 16.143(3) Å	β= 90°.
	c = 8.5434(16) Å	γ = 90°.
Volume	2586.1(8) $Å^3$	
Z	4	
Density (calculated)	1.442 Mg/m^3	
Absorption coefficient	1.287 mm^{-1}	
F(000)	1151	
Crystal size	0.061 x 0.053 x 0.021 mm^3	
Theta range for data collection	1.263 to 11.924°.	
Index ranges	-18<=h<=18, -16<=k<=16, -8<=l<=8	
Reflections collected	18135	
Independent reflections	2726 [R(int) = 0.1177]	
Completeness to theta = 11.924°	99.9 %	
Absorption correction	Semi-empirical from equivalents	
Max. and min. transmission	0.7437 and 0.3391	
Refinement method	Full-matrix least-squares on F^2	
Data / restraints / parameters	2726 / 340 / 260	
Goodness-of-fit on F^2	1.111	
Final R indices [I>2sigma(I)]	R1 = 0.0488, wR2 = 0.1149	
R indices (all data)	R1 = 0.0594, wR2 = 0.1207	
Absolute structure parameter	0.010(15)	
Extinction coefficient	n/a	
Largest diff. peak and hole	0.939 and -0.427 $eÅ^{-3}$	

Table S52: Bond lengths [Å] and angles [°] for **3**^{Pd,As}.

Pd(1)-As(1)	2.463(4)	C(9)-P(1)-Pd(1)	117.5(6)
Pd(1)-As(1A)	2.349(13)	C(9)-P(1)-C(5)	114.7(10)
Pd(1)-P(1)	2.352(4)	C(4)-P(2)-Pd(1)	97.6(6)
Pd(1)-P(2)	2.348(5)	C(4)-P(2)-C(13)	105.0(8)
Pd(1)-N(1)	2.047(13)	C(4)-P(2)-C(17)	105.1(9)
As(1)-C(21)	1.710(12)	C(13)-P(2)-Pd(1)	116.1(6)
P(1)-C(1)	1.762(17)	C(17)-P(2)-Pd(1)	116.4(6)
P(1)-C(5)	1.87(2)	C(17)-P(2)-C(13)	113.8(8)
P(1)-C(9)	1.82(2)	C(2)-N(1)-Pd(1)	120.1(11)
P(2)-C(4)	1.807(17)	C(2)-N(1)-C(3)	120.8(14)
P(2)-C(13)	1.896(19)	C(3)-N(1)-Pd(1)	119.1(11)
P(2)-C(17)	1.85(2)	C(2)-C(1)-P(1)	117.5(14)
O(1)-C(21)	1.194(12)	C(1)-C(2)-N(1)	121.9(16)
N(1)-C(2)	1.35(2)	C(4)-C(3)-N(1)	120.0(15)
N(1)-C(3)	1.42(2)	C(3)-C(4)-P(2)	119.4(13)
C(1)-C(2)	1.32(2)	C(6)-C(5)-P(1)	112.2(15)
C(3)-C(4)	1.32(2)	C(7)-C(5)-P(1)	106.1(13)
C(5)-C(6)	1.56(3)	C(7)-C(5)-C(6)	109.7(18)
C(5)-C(7)	1.48(4)	C(7)-C(5)-C(8)	108(2)
C(5)-C(8)	1.51(2)	C(8)-C(5)-P(1)	114.0(14)
C(13)-C(14)	1.51(2)	C(8)-C(5)-C(6)	106.5(15)
C(13)-C(15)	1.53(2)	C(10)-C(9)-P(1)	113.1(13)
C(13)-C(16)	1.57(2)	C(11)-C(9)-P(1)	113.8(15)
C(17)-C(18)	1.54(2)	C(11)-C(9)-C(10)	109.2(17)
C(17)-C(19)	1.50(3)	C(12)-C(9)-P(1)	107.6(14)
C(17)-C(20)	1.53(2)	C(12)-C(9)-C(10)	105.9(16)
As(1A)-Pd(1)-P(1)	99.2(6)	C(12)-C(9)-C(11)	106.6(17)
P(1)-Pd(1)-As(1)	98.59(14)	C(14)-C(13)-P(2)	113.3(11)
P(2)-Pd(1)-As(1)	96.21(14)	C(14)-C(13)-C(15)	109.7(14)
P(2)-Pd(1)-As(1A)	95.2(6)	C(14)-C(13)-C(16)	108.0(16)
P(2)-Pd(1)-P(1)	165.20(16)	C(15)-C(13)-P(2)	113.0(15)
N(1)-Pd(1)-As(1)	172.5(5)	C(15)-C(13)-C(16)	107.5(14)
N(1)-Pd(1)-As(1A)	173.4(9)	C(16)-C(13)-P(2)	104.9(11)
N(1)-Pd(1)-P(1)	81.6(4)	C(18)-C(17)-P(2)	112.6(13)
N(1)-Pd(1)-P(2)	83.7(4)	C(19)-C(17)-P(2)	106.1(13)
C(21)-As(1)-Pd(1)	93.7(8)	C(19)-C(17)-C(18)	108.6(16)
C(1)-P(1)-Pd(1)	98.6(6)	C(19)-C(17)-C(20)	108.4(17)
C(1)-P(1)-C(5)	105.4(8)	C(20)-C(17)-P(2)	112.9(13)
C(1)-P(1)-C(9)	104.1(9)	C(20)-C(17)-C(18)	108.2(16)
C(5)-P(1)-Pd(1)	113.7(7)	O(1)-C(21)-As(1)	173(2)

Symmetry transformations used to generate equivalent atoms:

Table S53: Torsion angles [°] for **3**^{Pd,As}.

Pd(1)-P(1)-C(1)-C(2)	6.0(15)	C(2)-N(1)-C(3)-C(4)	-179.4(19)
Pd(1)-P(1)-C(5)-C(6)	-165.5(12)	C(3)-N(1)-C(2)-C(1)	-179.8(16)
Pd(1)-P(1)-C(5)-C(7)	-45.6(15)	C(4)-P(2)-C(13)-C(14)	-179.6(13)
Pd(1)-P(1)-C(5)-C(8)	73.4(17)	C(4)-P(2)-C(13)-C(15)	-53.9(14)
Pd(1)-P(1)-C(9)-C(10)	-81.8(14)	C(4)-P(2)-C(13)-C(16)	62.9(14)
Pd(1)-P(1)-C(9)-C(11)	152.7(13)	C(4)-P(2)-C(17)-C(18)	163.8(13)
Pd(1)-P(1)-C(9)-C(12)	34.8(14)	C(4)-P(2)-C(17)-C(19)	-77.6(14)
Pd(1)-P(2)-C(4)-C(3)	4.3(18)	C(4)-P(2)-C(17)-C(20)	40.9(16)
Pd(1)-P(2)-C(13)-C(14)	74.0(13)	C(5)-P(1)-C(1)-C(2)	-111.6(15)
Pd(1)-P(2)-C(13)-C(15)	-160.4(10)	C(5)-P(1)-C(9)-C(10)	55.9(15)
Pd(1)-P(2)-C(13)-C(16)	-43.6(12)	C(5)-P(1)-C(9)-C(11)	-69.6(16)
Pd(1)-P(2)-C(17)-C(18)	-89.6(13)	C(5)-P(1)-C(9)-C(12)	172.5(11)
Pd(1)-P(2)-C(17)-C(19)	29.0(15)	C(9)-P(1)-C(1)-C(2)	127.3(15)
Pd(1)-P(2)-C(17)-C(20)	147.6(12)	C(9)-P(1)-C(5)-C(6)	55.2(16)
Pd(1)-N(1)-C(2)-C(1)	-1(2)	C(9)-P(1)-C(5)-C(7)	175.1(14)
Pd(1)-N(1)-C(3)-C(4)	2(2)	C(9)-P(1)-C(5)-C(8)	-65.9(18)
P(1)-C(1)-C(2)-N(1)	-4(2)	C(13)-P(2)-C(4)-C(3)	-115.3(17)
N(1)-C(3)-C(4)-P(2)	-4(3)	C(13)-P(2)-C(17)-C(18)	49.5(15)
C(1)-P(1)-C(5)-C(6)	-58.6(17)	C(13)-P(2)-C(17)-C(19)	168.1(13)

C(1)-P(1)-C(5)-C(7)	61.2(16)	C(13)-P(2)-C(17)-C(20)	-73.4(16)
C(1)-P(1)-C(5)-C(8)	-179.7(15)	C(17)-P(2)-C(4)-C(3)	124.3(17)
C(1)-P(1)-C(9)-C(10)	170.5(13)	C(17)-P(2)-C(13)-C(14)	-65.2(15)
C(1)-P(1)-C(9)-C(11)	45.0(16)	C(17)-P(2)-C(13)-C(15)	60.4(13)
C(1)-P(1)-C(9)-C(12)	-72.9(13)	C(17)-P(2)-C(13)-C(16)	177.2(11)

Symmetry transformations used to generate equivalent atoms:

X-ray Single-Crystal Structure Analysis of [Pt(P)(PNP)] ($3^{Pt,P}$)

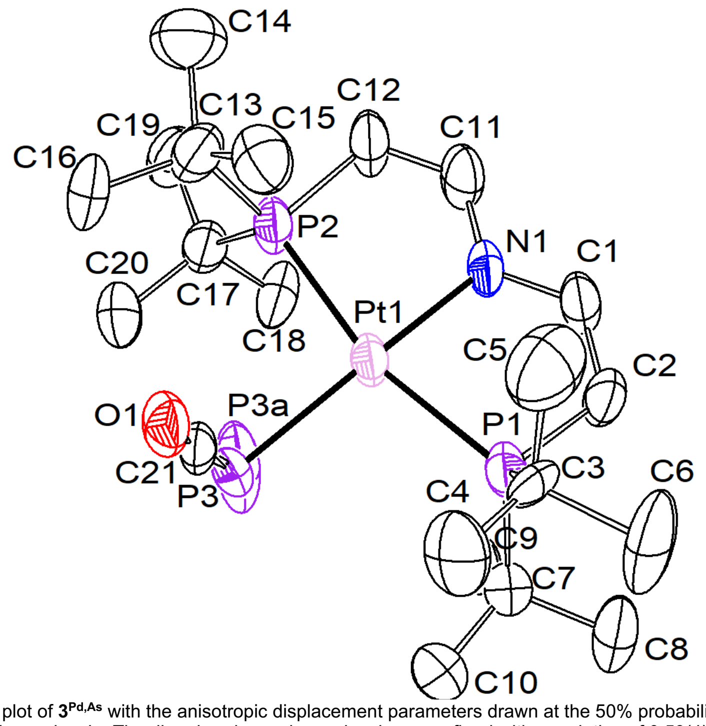

Figure S96: Thermal ellipsoid plot of $3^{Pd,As}$ with the anisotropic displacement parameters drawn at the 50% probability level. The asymmetric unit contains one disordered complex molecule. The disordered complex molecule was refined with population of 0.59(4) on the main domain using some restraints and constraints (SIMU, RIGU). The Pt-P3 and C21-O1 atoms were calculated and constraint to 2.41, and 1.19 Å by using DFIX.

Table S54: Crystal data and structure refinement for $3^{Pt,P}$.

Identification code	sspt7_2_run2	
Empirical formula	$C_{20.59}H_{40}NO_{0.59}P_3Pt$	
Formula weight	599.08	
Temperature	110(2) K	
Wavelength	0.41328 Å	
Crystal system	Orthorhombic	
Space group	$Pna2_1$	
Unit cell dimensions	a = 18.6215(15) Å	α= 90°.
	b = 15.8857(13) Å	β= 90°.
	c = 8.4095(7) Å	γ = 90°.
Volume	2487.7(4) Å^3	
Z	4	
Density (calculated)	1.611 Mg/m^3	
Absorption coefficient	1.390 mm^{-1}	
F(000)	1193	
Crystal size	0.050 x 0.050 x 0.050 mm^3	
Theta range for data collection	0.980 to 15.120°.	
Index ranges	-23<=h<=23, -19<=k<=19, -10<=l<=10	
Reflections collected	31923	
Independent reflections	5200 [R(int) = 0.0790]	
Completeness to theta = 14.357°	100.0 %	
Absorption correction	Semi-empirical from equivalents	
Max. and min. transmission	0.7439 and 0.5339	
Refinement method	Full-matrix least-squares on F^2	
Data / restraints / parameters	5200 / 23 / 266	
Goodness-of-fit on F^2	1.143	
Final R indices [I>2sigma(I)]	R1 = 0.0612, wR2 = 0.160	
R indices (all data)	R1 = 0.0789, wR2 = 0.1764	
Absolute structure parameter	0.011(15)	
Extinction coefficient	n/a	
Largest diff. peak and hole	4.855 and -1.983 e.Å^{-3}	

Table S55: Bond lengths [Å] and angles [°] for **$3^{Pt,P}$**.

Pt(1)-N(1)	2.066(15)
Pt(1)-P(3A)	2.25(4)
Pt(1)-P(2)	2.306(5)
Pt(1)-P(1)	2.318(5)
Pt(1)-P(3)	2.342(16)
P(1)-C(2)	1.74(2)
P(1)-C(3)	1.82(3)
P(1)-C(7)	1.86(2)
C(2)-C(1)	1.36(3)
P(3)-C(21)	1.56(3)
C(21)-O(1)	1.184(14)
C(3)-C(5)	1.43(3)
C(3)-C(6)	1.54(5)
C(3)-C(4)	1.59(3)
N(1)-C(1)	1.35(3)
N(1)-C(11)	1.36(3)
P(2)-C(12)	1.78(2)
P(2)-C(17)	1.84(2)
P(2)-C(13)	1.86(2)
C(7)-C(9)	1.45(3)
C(7)-C(10)	1.52(3)
C(7)-C(8)	1.53(3)
C(11)-C(12)	1.34(3)
C(13)-C(16)	1.50(3)
C(13)-C(14)	1.51(3)
C(13)-C(15)	1.57(3)
C(17)-C(20)	1.51(3)
C(17)-C(19)	1.52(3)
C(17)-C(18)	1.56(3)
N(1)-Pt(1)-P(3A)	173.5(14)
N(1)-Pt(1)-P(2)	82.6(5)
P(3A)-Pt(1)-P(2)	96.2(11)
N(1)-Pt(1)-P(1)	82.3(5)
P(3A)-Pt(1)-P(1)	99.0(11)
P(2)-Pt(1)-P(1)	164.80(17)
N(1)-Pt(1)-P(3)	174.4(7)
P(2)-Pt(1)-P(3)	96.7(5)
P(1)-Pt(1)-P(3)	98.5(5)
C(2)-P(1)-C(3)	107.5(11)
C(2)-P(1)-C(7)	104.0(10)
C(3)-P(1)-C(7)	115.4(12)
C(2)-P(1)-Pt(1)	99.6(7)
C(3)-P(1)-Pt(1)	111.8(7)
C(7)-P(1)-Pt(1)	116.5(8)
C(1)-C(2)-P(1)	117.2(14)
C(21)-P(3)-Pt(1)	102.9(13)
O(1)-C(21)-P(3)	176(2)
C(5)-C(3)-C(6)	114(3)
C(5)-C(3)-C(4)	105(2)
C(6)-C(3)-C(4)	105(3)
C(5)-C(3)-P(1)	116(2)
C(6)-C(3)-P(1)	106.6(17)
C(4)-C(3)-P(1)	109(2)
C(1)-N(1)-C(11)	122.0(17)
C(1)-N(1)-Pt(1)	118.9(14)
C(11)-N(1)-Pt(1)	119.1(12)
N(1)-C(1)-C(2)	121.5(19)
C(12)-P(2)-C(17)	103.7(10)
C(12)-P(2)-C(13)	105.3(12)
C(17)-P(2)-C(13)	113.1(10)
C(12)-P(2)-Pt(1)	99.6(8)
C(17)-P(2)-Pt(1)	116.6(8)
C(13)-P(2)-Pt(1)	115.9(7)
C(9)-C(7)-C(10)	111(2)
C(9)-C(7)-C(8)	108(2)
C(10)-C(7)-C(8)	108(2)
C(9)-C(7)-P(1)	106.0(17)
C(10)-C(7)-P(1)	110.5(18)
C(8)-C(7)-P(1)	113.4(19)
C(12)-C(11)-N(1)	121.8(19)
C(11)-C(12)-P(2)	116.8(18)
C(16)-C(13)-C(14)	107(2)
C(16)-C(13)-C(15)	109.3(19)
C(14)-C(13)-C(15)	108(2)
C(16)-C(13)-P(2)	114.8(18)
C(14)-C(13)-P(2)	112.6(18)
C(15)-C(13)-P(2)	105.1(14)
C(20)-C(17)-C(19)	109.2(18)
C(20)-C(17)-C(18)	106.8(19)
C(19)-C(17)-C(18)	108(2)
C(20)-C(17)-P(2)	114.4(18)
C(19)-C(17)-P(2)	113.0(15)
C(18)-C(17)-P(2)	105.0(13)

Symmetry transformations used to generate equivalent atoms:

Table S56: Torsion angles [°] for **$3^{Pt,P}$**.

C(3)-P(1)-C(2)-C(1)	-109(2)
C(7)-P(1)-C(2)-C(1)	128(2)
Pt(1)-P(1)-C(2)-C(1)	7(2)
C(2)-P(1)-C(3)-C(5)	-177(2)
C(7)-P(1)-C(3)-C(5)	-61(2)
Pt(1)-P(1)-C(3)-C(5)	75(2)
C(2)-P(1)-C(3)-C(6)	55(2)
C(7)-P(1)-C(3)-C(6)	170.7(19)
Pt(1)-P(1)-C(3)-C(6)	-53.1(19)
C(2)-P(1)-C(3)-C(4)	-58(2)
C(7)-P(1)-C(3)-C(4)	57(2)
Pt(1)-P(1)-C(3)-C(4)	-166.6(18)
C(11)-N(1)-C(1)-C(2)	180(2)
N(1)-C(11)-C(12)-P(2)	-2(4)
C(17)-P(2)-C(12)-C(11)	-117(2)
C(13)-P(2)-C(12)-C(11)	124(2)
Pt(1)-P(2)-C(12)-C(11)	3(2)
Pt(1)-N(1)-C(1)-C(2)	0(3)
P(1)-C(2)-C(1)-N(1)	-5(3)
C(2)-P(1)-C(7)-C(9)	-71.5(17)
C(3)-P(1)-C(7)-C(9)	171.0(15)
Pt(1)-P(1)-C(7)-C(9)	36.9(18)
C(2)-P(1)-C(7)-C(10)	168.7(17)
C(3)-P(1)-C(7)-C(10)	51(2)
Pt(1)-P(1)-C(7)-C(10)	-82.9(18)
C(2)-P(1)-C(7)-C(8)	47(2)
C(3)-P(1)-C(7)-C(8)	-70(2)
Pt(1)-P(1)-C(7)-C(8)	155.6(19)
C(1)-N(1)-C(11)-C(12)	180(2)
Pt(1)-N(1)-C(11)-C(12)	0(3)
C(17)-P(2)-C(13)-C(15)	168.4(15)
Pt(1)-P(2)-C(13)-C(15)	29.9(17)
C(12)-P(2)-C(17)-C(20)	-54.4(18)
C(13)-P(2)-C(17)-C(20)	59.1(18)

C(12)-P(2)-C(13)-C(16)	160.8(17)	Pt(1)-P(2)-C(17)-C(20)	-162.8(13)
C(17)-P(2)-C(13)-C(16)	48(2)	C(12)-P(2)-C(17)-C(19)	179.8(17)
Pt(1)-P(2)-C(13)-C(16)	-90.2(17)	C(13)-P(2)-C(17)-C(19)	-66.7(19)
C(12)-P(2)-C(13)-C(14)	38(2)	Pt(1)-P(2)-C(17)-C(19)	71.4(17)
C(17)-P(2)-C(13)-C(14)	-74(2)	C(12)-P(2)-C(17)-C(18)	62.3(18)
Pt(1)-P(2)-C(13)-C(14)	147.1(17)	C(13)-P(2)-C(17)-C(18)	175.8(16)
C(12)-P(2)-C(13)-C(15)	-79.0(17)	Pt(1)-P(2)-C(17)-C(18)	-46.1(17)

Symmetry transformations used to generate equivalent atoms:

X-ray Single-Crystal Structure Analysis of [Pt(As)(PNP)] ($3^{Pt,As}$)

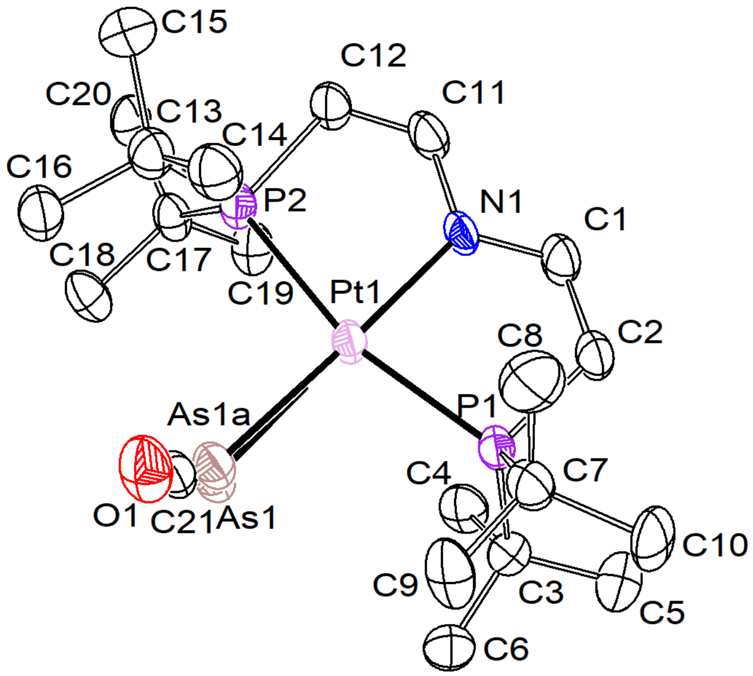

Figure S97: Thermal ellipsoid plot $3^{Pt,As}$ with the anisotropic displacement parameters drawn at the 50% probability level. The asymmetric unit contains one complex molecule. The structure picture results from 14 % of $1^{Pt,As}$ to $3^{Pt,As}$.For refinement restraints and constraints (SIMU, RIGU, EADP) have been used. The Pt1-As1 atoms were calculated and constraint to 2.46 Å by using DFIX and isotropically refined. The reflections 0 3 -5, 4 2 0, 10 0 -3, 10 1 -3, 10 2 -3, 11 1 -3, 17 2 -1 and 18 1 -1 are removed from the refinement using OMIT command.

Table S57: Crystal data and structure refinement for $3^{Pt,As}$.

Identification code	sven_ptasco_run4_2a	
Empirical formula	$C_{20.86}$ H_{40} As N $O_{0.86}$ P_2 Pt	
Formula weight	650.57	
Temperature	100.00 K	
Wavelength	0.41328 Å	
Crystal system	Orthorhombic	
Space group	$Pna2_1$	
Unit cell dimensions	a = 18.556(6) Å	α= 90°.
	b = 15.833(7) Å	β= 90°.
	c = 8.423(4) Å	γ = 90°.
Volume	2474.7(17) Å^3	
Z	4	
Density (calculated)	1.738 Mg/m^3	
Absorption coefficient	1.737 mm^{-1}	
F(000)	1274	
Crystal size	0.065 x 0.03 x 0.02 mm^3	
Theta range for data collection	0.983 to 14.222°.	
Index ranges	-21<=h<=21, -18<=k<=18, -9<=l<=9	
Reflections collected	28616	
Independent reflections	4308 [R(int) = 0.1614]	
Completeness to theta = 14.222°	99.4 %	
Absorption correction	Semi-empirical from equivalents	
Max. and min. transmission	0.7435 and 0.6784	
Refinement method	Full-matrix least-squares on F^2	
Data / restraints / parameters	4308 / 407 / 259	
Goodness-of-fit on F^2	1.075	
Final R indices [I>2sigma(I)]	R1 = 0.0504, wR2 = 0.1313	
R indices (all data)	R1 = 0.0562, wR2 = 0.1348	
Absolute structure parameter	0.07(3)	
Extinction coefficient	n/a	
Largest diff. peak and hole	2.184 and -1.573 e.Å^{-3}	

Table S58: Bond lengths [Å] and angles [°] for **3**^{Pt,As}.

Pt(1)-P(1)	2.308(4)
Pt(1)-P(2)	2.297(4)
Pt(1)-N(1)	2.039(13)
Pt(1)-As(1)	2.449(4)
Pt(1)-As(1A)	2.36(3)
P(1)-C(2)	1.783(17)
P(1)-C(7)	1.84(2)
P(1)-C(3)	1.854(18)
P(2)-C(12)	1.786(17)
P(2)-C(13)	1.875(18)
P(2)-C(17)	1.869(18)
N(1)-C(1)	1.33(2)
N(1)-C(11)	1.37(2)
C(1)-C(2)	1.33(3)
C(11)-C(12)	1.33(3)
C(7)-C(9)	1.51(2)
C(7)-C(8)	1.43(3)
C(7)-C(10)	1.57(2)
C(3)-C(6)	1.48(3)
C(3)-C(5)	1.51(3)
C(3)-C(4)	1.54(3)
C(13)-C(14)	1.48(3)
C(13)-C(15)	1.52(2)
C(13)-C(16)	1.54(2)
C(17)-C(18)	1.56(2)
C(17)-C(19)	1.52(2)
C(17)-C(20)	1.49(2)
As(1)-C(21)	1.75(2)
O(1)-C(21)	1.17(3)
P(1)-Pt(1)-As(1)	98.19(15)
P(1)-Pt(1)-As(1A)	99.6(8)
P(2)-Pt(1)-P(1)	165.14(15)
P(2)-Pt(1)-As(1)	96.66(15)
P(2)-Pt(1)-As(1A)	95.1(8)
N(1)-Pt(1)-P(1)	81.6(4)
N(1)-Pt(1)-P(2)	83.6(4)
N(1)-Pt(1)-As(1)	173.2(4)
N(1)-Pt(1)-As(1A)	176.8(7)
C(2)-P(1)-Pt(1)	99.5(6)
C(2)-P(1)-C(7)	104.3(8)
C(2)-P(1)-C(3)	105.4(9)
C(7)-P(1)-Pt(1)	112.3(7)
C(7)-P(1)-C(3)	115.1(10)
C(3)-P(1)-Pt(1)	117.7(6)
C(12)-P(2)-Pt(1)	99.3(6)
C(12)-P(2)-C(13)	104.3(9)
C(12)-P(2)-C(17)	104.5(8)
C(13)-P(2)-Pt(1)	116.2(6)
C(17)-P(2)-Pt(1)	115.2(6)
C(17)-P(2)-C(13)	114.5(8)
C(1)-N(1)-Pt(1)	120.7(12)
C(1)-N(1)-C(11)	121.6(15)
C(11)-N(1)-Pt(1)	117.6(11)
N(1)-C(1)-C(2)	122.3(16)
C(1)-C(2)-P(1)	115.5(13)
C(12)-C(11)-N(1)	123.5(16)
C(11)-C(12)-P(2)	115.6(14)
C(9)-C(7)-P(1)	112.6(14)
C(9)-C(7)-C(10)	106.7(16)
C(8)-C(7)-P(1)	107.4(14)
C(8)-C(7)-C(9)	112(2)
C(8)-C(7)-C(10)	108.5(18)
C(10)-C(7)-P(1)	109.4(14)
C(6)-C(3)-P(1)	112.6(14)
C(6)-C(3)-C(5)	110.3(17)
C(6)-C(3)-C(4)	108.1(16)
C(5)-C(3)-P(1)	112.9(15)
C(5)-C(3)-C(4)	108.2(17)
C(4)-C(3)-P(1)	104.4(13)
C(14)-C(13)-P(2)	104.7(13)
C(14)-C(13)-C(15)	109.1(16)
C(14)-C(13)-C(16)	110.8(16)
C(15)-C(13)-P(2)	112.5(13)
C(15)-C(13)-C(16)	107.8(16)
C(16)-C(13)-P(2)	111.8(12)
C(18)-C(17)-P(2)	113.1(12)
C(19)-C(17)-P(2)	104.1(11)
C(19)-C(17)-C(18)	109.3(16)
C(20)-C(17)-P(2)	111.4(13)
C(20)-C(17)-C(18)	108.8(14)
C(20)-C(17)-C(19)	110.1(14)
C(21)-As(1)-Pt(1)	98.2(7)
O(1)-C(21)-As(1)	179(2)

Symmetry transformations used to generate equivalent atoms:

Table S59: Torsion angles [°] for **3**^{Pt,As}.

Pt(1)-P(1)-C(2)-C(1)	4.8(17)
Pt(1)-P(1)-C(7)-C(9)	74.3(17)
Pt(1)-P(1)-C(7)-C(8)	-49.7(15)
Pt(1)-P(1)-C(7)-C(10)	-167.3(12)
Pt(1)-P(1)-C(3)-C(6)	-81.9(15)
Pt(1)-P(1)-C(3)-C(5)	152.3(13)
Pt(1)-P(1)-C(3)-C(4)	35.1(14)
Pt(1)-P(2)-C(12)-C(11)	4.9(17)
Pt(1)-P(2)-C(13)-C(14)	29.7(14)
Pt(1)-P(2)-C(13)-C(15)	148.1(12)
Pt(1)-P(2)-C(13)-C(16)	-90.4(13)
Pt(1)-P(2)-C(17)-C(18)	73.4(13)
Pt(1)-P(2)-C(17)-C(19)	-45.1(13)
Pt(1)-P(2)-C(17)-C(20)	-163.7(10)
Pt(1)-N(1)-C(1)-C(2)	-3(3)
C(12)-P(2)-C(17)-C(19)	62.8(13)
C(12)-P(2)-C(17)-C(20)	-55.8(13)
Pt(1)-N(1)-C(11)-C(12)	3(3)
N(1)-C(1)-C(2)-P(1)	-2(3)
N(1)-C(11)-C(12)-P(2)	-6(3)
C(1)-N(1)-C(11)-C(12)	180(2)
C(2)-P(1)-C(7)-C(9)	-178.9(15)
C(2)-P(1)-C(7)-C(8)	57.1(16)
C(2)-P(1)-C(7)-C(10)	-60.5(16)
C(2)-P(1)-C(3)-C(6)	168.3(14)
C(2)-P(1)-C(3)-C(5)	42.6(17)
C(2)-P(1)-C(3)-C(4)	-74.7(13)
C(11)-N(1)-C(1)-C(2)	-180(2)
C(12)-P(2)-C(13)-C(14)	-78.5(14)
C(12)-P(2)-C(13)-C(15)	39.9(15)
C(12)-P(2)-C(13)-C(16)	161.4(13)
C(12)-P(2)-C(17)-C(18)	-178.7(13)
C(3)-P(1)-C(7)-C(10)	54.4(16)
C(13)-P(2)-C(12)-C(11)	125.2(17)

C(7)-P(1)-C(2)-C(1)	-111.3(17)	C(13)-P(2)-C(17)-C(18)	-65.3(15)
C(7)-P(1)-C(3)-C(6)	54.0(16)	C(13)-P(2)-C(17)-C(19)	176.2(12)
C(7)-P(1)-C(3)-C(5)	-71.7(17)	C(13)-P(2)-C(17)-C(20)	57.6(14)
C(7)-P(1)-C(3)-C(4)	171.1(11)	C(17)-P(2)-C(12)-C(11)	-114.4(17)
C(3)-P(1)-C(2)-C(1)	127.2(17)	C(17)-P(2)-C(13)-C(14)	167.9(12)
C(3)-P(1)-C(7)-C(9)	-64.0(18)	C(17)-P(2)-C(13)-C(15)	-73.7(15)
C(3)-P(1)-C(7)-C(8)	172.0(14)	C(17)-P(2)-C(13)-C(16)	47.9(16)

Symmetry transformations used to generate equivalent atoms:

References

(1) Schmidt-Räntsch, T.; Verplancke, H.; Lienert, J. N.; Demeshko, S.; Otte, M.; Van Trieste, G. P.; Reid, K. A.; Reibenspies, J. H.; Powers, D. C.; Holthausen, M. C.; Schneider, S. Nitrogen Atom Transfer Catalysis by Metallonitrene C−H Insertion: Photocatalytic Amidation of Aldehydes. *Angew. Chem.- Int. Ed.* **2022**, *61* (9), e202115626.

(2) Sun, J.; Abbenseth, J.; Verplancke, H.; Diefenbach, M.; de Bruin, B.; Hunger, D.; Würtele, C.; van Slageren, J.; Holthausen, M. C.; Schneider, S. A Platinum(Ii) Metallonitrene with a Triplet Ground State. *Nat. Chem.* **2020**, *12* (11), 1054–1059. https://doi.org/10.1038/s41557-020-0522-4.

(3) Sun, J.; Verplancke, H.; Schweizer, J. I.; Diefenbach, M.; Würtele, C.; Otte, M.; Tkach, I.; Herwig, C.; Limberg, C.; Demeshko, S.; Holthausen, M. C.; Schneider, S. Stabilizing P≡P: P22–, P2·–, and P20 as Bridging Ligands. *Chem* **2021**, *7* (7), 1952–1962. https://doi.org/10.1016/J.CHEMPR.2021.06.006.

(4) Heift, D.; Benko, Z.; Grützmacher, H. Coulomb Repulsion versus Cycloaddition: Formation of Anionic Four-Membered Rings from Sodium Phosphaethynolate, Na(OCP). *Dalt. Trans.* **2014**, *43* (2), 831–840. https://doi.org/10.1039/c3dt52359d.

(5) Hinz, A.; Goicoechea, J. M. The 2-Arsaethynolate Anion: Synthesis and Reactivity Towards Heteroallenes. *Angew. Chem.- Int. Ed.* **2016**, *55* (30), 8536–8541. https://doi.org/10.1002/anie.201602310.

(6) Chávez, I.; Alvarez-Carena, A.; Molins, E.; Roig, A.; Maniukiewicz, W.; Arancibia, A.; Arancibia, V.; Brand, H.; Manuel Manríquez, J. Selective Oxidants for Organometallic Compounds Containing a Stabilising Anion of Highly Reactive Cations: (3,5(CF3)2C6H3)4B−)Cp2Fe+ and (3,5(CF3)2C6H3)4B−)Cp*2Fe+. *J. Organomet. Chem.* **2000**, *601* (1), 126–132. https://doi.org/10.1016/S0022-328X(00)00044-9.

(7) Hornung, B.; Bodi, A.; Pongor, C. I.; Gengeliczki, Z.; Baer, T.; Sztáray, B. Dissociative Photoionization of X(CH 3) 3 (X = N, P, As, Sb, Bi): Mechanism, Trends, and Accurate Energetics. *J. Phys. Chem. A* **2009**, *113* (28), 8091–8098. https://doi.org/10.1021/jp900920r.

(8) Dollberg, K.; Schneider, S.; Richter, R. M.; Dunaj, T.; von Hänisch, C. Synthesis and Application of Alkali Metal Antimonide—A New Approach to Antimony Chemistry. *Angew. Chem.- Int. Ed.* **2022**, *61* (52), e202213098. https://doi.org/10.1002/anie.202213098.

(9) Evans, D. F. The Determination of the Paramagnetic Susceptibility of Substances in Solution by Nuclear Magnetic Resonance. *J. Chem. Soc.* **1959**, *81*, 2003–2005. https://doi.org/10.1039/jr9590002003.

(10) Sur, S. K. Measurement of Magnetic Susceptibility and Magnetic Moment of Paramagnetic Molecules in Solution by High-Field Fourier Transform NMR Spectroscopy. *J. Magn. Reson.* **1989**, *82* (1), 169–173. https://doi.org/10.1016/0022-2364(89)90178-9.

(11) Stoll, S.; Schweiger, A. EasySpin, a Comprehensive Software Package for Spectral Simulation and Analysis in EPR. *J. Magn. Reson.* **2006**, *178* (1), 42–55. https://doi.org/10.1016/J.JMR.2005.08.013.

(12) Nehrkorn, J.; Holldack, K.; Bittl, R.; Schnegg, A. Recent Progress in Synchrotron-Based Frequency-Domain Fourier-Transform THz-EPR. *J. Magn. Reson.* **2017**, *280*, 10–19. https://doi.org/10.1016/J.JMR.2017.04.001.

(13) Nehrkorn, J.; Schnegg, A.; Holldack, K.; Stoll, S. General Magnetic Transition Dipole Moments for Electron Paramagnetic Resonance. *Phys. Rev. Lett.* **2015**, *114* (1), 010801. https://doi.org/10.1103/PHYSREVLETT.114.010801/FIGURES/2/MEDIUM.

(14) Frisch, M. J.; Trucks, G. W.; Schlegel, H. B.; Scuseria, G. E.; Robb, M. a.; Cheeseman, J. R.; Scalmani, G.; Barone, V.; Petersson, G. a.; Nakatsuji, H.; Li, X.; Caricato, M.; Marenich, a. V.; Bloino, J.; Janesko, B. G.; Gomperts, R.; Mennucci, B.; Hratchian, H. P.; Ortiz, J. V.; Izmaylov, a. F.; Sonnenberg, J. L.; Williams; Ding, F.; Lipparini, F.; Egidi, F.; Goings, J.; Peng, B.; Petrone, A.; Henderson, T.; Ranasinghe, D.; Zakrzewski, V. G.; Gao, J.; Rega, N.; Zheng, G.; Liang, W.; Hada, M.; Ehara, M.; Toyota, K.; Fukuda, R.; Hasegawa, J.; Ishida, M.; Nakajima, T.; Honda, Y.; Kitao, O.; Nakai, H.; Vreven, T.; Throssell, K.; Montgomery Jr., J. a.; Peralta, J. E.; Ogliaro, F.; Bearpark, M. J.; Heyd, J. J.; Brothers, E. N.; Kudin, K. N.; Staroverov, V. N.; Keith, T. a.; Kobayashi, R.; Normand, J.; Raghavachari, K.; Rendell, a. P.; Burant, J. C.; Iyengar, S. S.; Tomasi, J.; Cossi, M.; Millam, J. M.; Klene, M.; Adamo, C.; Cammi, R.; Ochterski, J. W.; Martin, R. L.; Morokuma, K.; Farkas, O.; Foresman, J. B.; Fox, D. J. *Gaussian 16, Revision B.01*; Gaussian Inc.: Wallingford, CT, 2016.

(15) Perdew, J. P.; Ernzerhof, M.; Burke, K. Rationale for Mixing Exact Exchange with Density Functional Approximations. *J. Chem. Phys.* **1996**, *105* (22), 9982–9985. https://doi.org/10.1063/1.472933.

(16) Adamo, C.; Barone, V. Toward Reliable Density Functional Methods without Adjustable Parameters: The PBE0 Model. *J. Chem. Phys.* **1999**, *110* (13), 6158–6170. https://doi.org/10.1063/1.478522.

(17) Weigend, F.; Ahlrichs, R. Balanced Basis Sets of Split Valence, Triple Zeta Valence and Quadruple Zeta Valence Quality for H to Rn: Design and Assessment of Accuracy. *Phys. Chem. Chem. Phys.* **2005**, *7* (18), 3297–3305. https://doi.org/10.1039/b508541a.

(18) Andrae, D.; Häußermann, U.; Dolg, M.; Stoll, H.; Preuß, H. Energy-Adjusted Ab Initio Pseudopotentials for the Second and Third Row Transition Elements. *Theor. Chim. Acta* **1990**, *77* (2), 123–141. https://doi.org/10.1007/BF01114537/METRICS.

(19) Metz, B.; Stoll, H.; Dolg, M. Small-Core Multiconfiguration-Dirac-Hartree-Fock-Adjusted Pseudopotentials for Post-d Main Group Elements: Application to PbH and PbO. *J. Chem. Phys.* **2000**, *113* (7), 2563–2569. https://doi.org/10.1063/1.1305880.

(20) Grimme, S.; Antony, J.; Ehrlich, S.; Krieg, H. A Consistent and Accurate Ab Initio Parametrization of Density Functional Dispersion Correction (DFT-D) for the 94 Elements H-Pu. *J. Chem. Phys.* **2010**, *132* (15). https://doi.org/10.1063/1.3382344.

(21) Becke, A. D.; Johnson, E. R. Exchange-Hole Dipole Moment and the Dispersion Interaction. *J. Chem. Phys.* **2005**, *122* (15), 154104. https://doi.org/10.1063/1.1884601/922477.

(22) Becke, A. D.; Johnson, E. R. A Density-Functional Model of the Dispersion Interaction. *J. Chem. Phys.* **2005**, *123* (15). https://doi.org/10.1063/1.2065267/347819.

(23) Johnson, E. R.; Becke, A. D. A Post-Hartree-Fock Model of Intermolecular Interactions: Inclusion of Higher-Order Corrections. *J. Chem. Phys.* **2006**, *124* (17). https://doi.org/10.1063/1.2190220/935697.

(24) Grimme, S.; Ehrlich, S.; Goerigk, L. Effect of the Damping Function in Dispersion Corrected Density Functional Theory. *J. Comput. Chem.* **2011**, *32* (7), 1456–1465. https://doi.org/10.1002/JCC.21759.

(25) Glendening, E. D.; Badenhoop, J., K.; Reed, A. E.; Carpenter, J. E.; Bohmann, J. A.; Morales, C. M.; Landis, C. R.; Weinhold, F. *NBO 6.0*; Theoretical

Chemistry Institute University of Wisconsin: Madison, WI, 2013.

(26) Perdew, J. P. Density-Functional Approximation for the Correlation Energy of the Inhomogeneous Electron Gas. *Phys. Rev. B* **1986**, *33* (12), 8822. https://doi.org/10.1103/PhysRevB.33.8822.

(27) Becke, A. D. Density-Functional Exchange-Energy Approximation with Correct Asymptotic Behavior. *Phys. Rev. A* **1988**, *38* (6), 3098–3100. https://doi.org/10.1103/PhysRevA.38.3098.

(28) Neese, F. Software Update: The ORCA Program System, Version 4.0. *Wiley Interdiscip. Rev. Comput. Mol. Sci.* **2018**, *8* (1), e1327. https://doi.org/10.1002/WCMS.1327.

(29) Neese, F.; Wennmohs, F.; Becker, U.; Riplinger, C. The ORCA Quantum Chemistry Program Package. *J. Chem. Phys.* **2020**, *152* (22), 224108. https://doi.org/10.1063/5.0004608/1061982.

(30) Angeli, C.; Cimiraglia, R.; Evangelisti, S.; Leininger, T.; Malrieu, J. P. Introduction of N-Electron Valence States for Multireference Perturbation Theory. *J. Chem. Phys.* **2001**, *114* (23), 10252. https://doi.org/10.1063/1.1361246.

(31) Angeli, C.; Cimiraglia, R.; Malrieu, J. P. N-Electron Valence State Perturbation Theory: A Spinless Formulation and an Efficient Implementation of the Strongly Contracted and of the Partially Contracted Variants. *J. Chem. Phys.* **2002**, *117* (20), 9138–9153. https://doi.org/10.1063/1.1515317.

(32) Angeli, C.; Cimiraglia, R.; Malrieu, J. P. N-Electron Valence State Perturbation Theory: A Fast Implementation of the Strongly Contracted Variant. *Chem. Phys. Lett.* **2001**, *350* (3–4), 297–305. https://doi.org/10.1016/S0009-2614(01)01303-3.

(33) Heß, B. A.; Marian, C. M.; Wahlgren, U.; Gropen, O. A Mean-Field Spin-Orbit Method Applicable to Correlated Wavefunctions. *Chem. Phys. Lett.* **1996**, *251* (5–6), 365–371. https://doi.org/10.1016/0009-2614(96)00119-4.

(34) Van Lenthe, E.; Baerends, E. J.; Snijders, J. G. Relativistic Regular Two-Component Hamiltonians. *J. Chem. Phys.* **1993**, *99* (6), 4597–4610. https://doi.org/10.1063/1.466059.

(35) Van Lenthe, E.; Baerends, E. J.; Snijders, J. G. Relativistic Total Energy Using Regular Approximations. *J. Chem. Phys.* **1994**, *101* (11), 9783–9792. https://doi.org/10.1063/1.467943.

(36) Van Lenthe, E.; Snijders, J. G.; Baerends, E. J. The Zero-Order Regular Approximation for Relativistic Effects: The Effect of Spin-Orbit Coupling in Closed Shell Molecules. *J. Chem. Phys.* **1996**, *105* (15), 6505–6516. https://doi.org/10.1063/1.472460.

(37) Van Wüllen, C. Molecular Density Functional Calculations in the Regular Relativistic Approximation: Method, Application to Coinage Metal Diatomics, Hydrides, Fluorides and Chlorides, and Comparison with First-Order Relativistic Calculations. *J. Chem. Phys.* **1998**, *109* (2), 392–399. https://doi.org/10.1063/1.476576.

(38) Pantazis, D. A.; Chen, X. Y.; Landis, C. R.; Neese, F. All-Electron Scalar Relativistic Basis Sets for Third-Row Transition Metal Atoms. *J. Chem. Theory Comput.* **2008**, *4* (6), 908–919. https://doi.org/10.1021/CT800047T/SUPPL_FILE/CT800047T-FILE003.TXT.

(39) Rolfes, J. D.; Neese, F.; Pantazis, D. A. All-Electron Scalar Relativistic Basis Sets for the Elements Rb–Xe. *J. Comput. Chem.* **2020**, *41* (20), 1842–1849. https://doi.org/10.1002/JCC.26355.

(40) Weigend, F. A Fully Direct RI-HF Algorithm: Implementation, Optimised Auxiliary Basis Sets, Demonstration of Accuracy and Efficiency. *Phys. Chem. Chem. Phys.* **2002**, *4* (18), 4285–4291. https://doi.org/10.1039/B204199P.

(41) Stoychev, G. L.; Auer, A. A.; Neese, F. Automatic Generation of Auxiliary Basis Sets. *J. Chem. Theory Comput.* **2017**, *13* (2), 554–562. https://doi.org/10.1021/ACS.JCTC.6B01041.

(42) Singh, S. K.; Eng, J.; Atanasov, M.; Neese, F. Covalency and Chemical Bonding in Transition Metal Complexes: An Ab Initio Based Ligand Field Perspective. *Coord. Chem. Rev.* **2017**, *344*, 2–25. https://doi.org/10.1016/J.CCR.2017.03.018.

(43) Verplancke, H.; Diefenbach, M.; Lienert, J. N.; Ugandi, M.; Kitsaras, M. P.; Roemelt, M.; Stopkowicz, S.; Holthausen, M. C. Another Torture Track for Quantum Chemistry: Reinvestigation of the Benzaldehyde Amidation by Nitrogen-Atom Transfer from Platinum(II) and Palladium(II) Metallonitrenes. *Isr. J. Chem.* **2023**, *63* (7–8), e202300060. https://doi.org/10.1002/IJCH.202300060.

(44) Neese, F. Software Update: The ORCA Program System—Version 5.0. *Wiley Interdiscip. Rev. Comput. Mol. Sci.* **2022**, *12* (5), e1606. https://doi.org/10.1002/WCMS.1606.

(45) Becke, A. D. Density-Functional Thermochemistry. III. The Role of Exact Exchange. *J. Chem. Phys.* **1993**, *98* (7), 5648–5652. https://doi.org/10.1063/1.464913.

(46) Becke, A. D. Density-Functional Thermochemistry. IV. A New Dynamical Correlation Functional and Implications for Exact-Exchange Mixing. *J. Chem. Phys.* **1996**, *104* (3), 1040–1046. https://doi.org/10.1063/1.470829.

(47) Cammi, R.; Mennucci, B.; Tomasi, J. Fast Evaluation of Geometries and Properties of Excited Molecules in Solution: A Tamm-Dancoff Model with Application to 4-Dimethylaminobenzonitrile. *J. Phys. Chem. A* **2000**, *104* (23), 5631–5637. https://doi.org/10.1021/JP000156L/ASSET/IMAGES/LARGE/JP000156LF00002.JPEG.

(48) Becke, A. D. Density-Functional Exchange-Energy Approximation with Correct Asymptotic Behavior. *Phys. Rev. A* **1988**, *38* (6), 3098. https://doi.org/10.1103/PhysRevA.38.3098.

(49) Tao, J.; Perdew, J. P.; Staroverov, V. N.; Scuseria, G. E. Climbing the Density Functional Ladder: Nonempirical Meta–Generalized Gradient Approximation Designed for Molecules and Solids. *Phys. Rev. Lett.* **2003**, *91* (14), 146401. https://doi.org/10.1103/PHYSREVLETT.91.146401/FIGURES/1/MEDIUM.

(50) Yanai, T.; Tew, D. P.; Handy, N. C. A New Hybrid Exchange–Correlation Functional Using the Coulomb-Attenuating Method (CAM-B3LYP). *Chem. Phys. Lett.* **2004**, *393* (1–3), 51–57. https://doi.org/10.1016/J.CPLETT.2004.06.011.

(51) Twamley, B.; Sofield, C. D.; Olmstead, M. M.; Power, P. P. Homologous Series of Heavier Element Dipnictenes 2,6- Ar2H3C6E=EC6H3-2,6-Ar2 (E = P, As, Sb, Bi; Ar = Mes = C6H2-2,4,6- Me3; or Trip = C6H2-2,4,6-(i)Pr3) Stabilized by m-Terphenyl Ligands. *J. Am. Chem. Soc.* **1999**, *121* (14), 3357–3367. https://doi.org/10.1021/JA983999N.

(52) Vilarrubias, P. The Dipnictenes R-E=E′-R′ (E=P, As, Sb, Bi) Revisited: A Tddft and Multi-Reference Study of Some Aspects of Its Electronic Spectroscopy. *Mol. Phys.* **2017**, *115* (20), 2597–2604. https://doi.org/10.1080/00268976.2017.1338366.

(53) Pyykkö, P. Additive Covalent Radii for Single-, Double-, and Triple-Bonded Molecules and Tetrahedrally Bonded Crystals: A Summary. *J. Phys. Chem. A* **2015**, *119* (11), 2326–2337. https://doi.org/10.1021/jp5065819.

(54) *APEX3 V2019.1-0 (SAINT/SADABS/SHELXT/SHELXL)*; Bruker AXS Inc.: Madison, WI, USA, 2019.

(55) Sheldrick, G. M. A Short History of SHELX. *Acta Crystallogr. Sect. A Found. Crystallogr.* **2008**, *64* (1), 112–122.

(56) Sheldrick, G. M. SHELXT–Integrated Space-Group and Crystal-Structure Determination. *Acta Crystallogr. Sect. A Found. Adv.* **2015**, *71* (1), 3–8.

(57) Sheldrick, G. M. Crystal Structure Refinement with SHELXL. *Acta Crystallogr. Sect. C Struct. Chem.* **2015**, *71* (1), 3–8.
